# Evolution and Functional Implications of the Tricarboxylic Acid Cycle as Revealed by Phylogenetic Analysis

**DOI:** 10.1093/gbe/evu221

**Published:** 2014-10-01

**Authors:** João Henrique Frota Cavalcanti, Alberto A. Esteves-Ferreira, Carla G.S. Quinhones, Italo A. Pereira-Lima, Adriano Nunes-Nesi, Alisdair R. Fernie, Wagner L. Araújo

**Affiliations:** ^1^Departamento de Biologia Vegetal, Universidade Federal de Viçosa, MG, Brazil; ^2^Max-Planck-Partner Group at the Departamento de Biologia Vegetal, Universidade Federal de Viçosa, MG, Brazil; ^3^Max-Planck-Institut für Molekulare Pflanzenphysiologie, Potsdam-Golm, Germany

**Keywords:** mitochondria, pathway evolution, plant respiration, phylogeny, TCA cycle

## Abstract

The tricarboxylic acid (TCA) cycle, a crucial component of respiratory metabolism, is composed of a set of eight enzymes present in the mitochondrial matrix. However, most of the TCA cycle enzymes are encoded in the nucleus in higher eukaryotes. In addition, evidence has accumulated demonstrating that nuclear genes were acquired from the mitochondrial genome during the course of evolution. For this reason, we here analyzed the evolutionary history of all TCA cycle enzymes in attempt to better understand the origin of these nuclear-encoded proteins. Our results indicate that prior to endosymbiotic events the TCA cycle seemed to operate only as isolated steps in both the host (eubacterial cell) and mitochondria (alphaproteobacteria). The origin of isoforms present in different cell compartments might be associated either with gene-transfer events which did not result in proper targeting of the protein to mitochondrion or with duplication events. Further in silico analyses allow us to suggest new insights into the possible roles of TCA cycle enzymes in different tissues. Finally, we performed coexpression analysis using mitochondrial TCA cycle genes revealing close connections among these genes most likely related to the higher efficiency of oxidative phosphorylation in this specialized organelle. Moreover, these analyses allowed us to identify further candidate genes which might be used for metabolic engineering purposes given the importance of the TCA cycle during development and/or stress situations.

## Introduction

Mitochondria, vital cytoplasmic organelles of eukaryotic cells, were identified over 50 years ago as being responsible for oxidative energy metabolism and the synthesis of the majority of respiratory adenosine-5′-triphosphate (ATP) in plants, animals, and fungi (Logan 2006; Araújo, Nunes-Nesi, et al. 2012). Although mitochondria contain their own genome and the machinery for its replication (Gray et al. 2001; Burger et al. 2003; Gray 2012), they are only semiautonomous. Indeed the majority of mitochondrial polypeptides is encoded in the nuclear genome, synthesized in the cytosol, and imported into the mitochondria posttranscriptionally (Unseld et al. 1997; Whelan and Glaser 1997; Tanudji et al. 2001; Duby and Boutry 2002; Eckers et al. 2012). Thus, it is assumed that eukaryotic cells arose through the capture of free-living bacteria by endosymbiosis and their gradual conversion into organelles (plastid and mitochondria).

Today, it is essentially beyond question that eukaryotes originated from an ancient unicellular bacterial-like cell by the engulfment of other free-living bacteria. In doing so, they acquired new functionalities through a cooperative relationship—a process termed endosymbiosis or endocytobiosis (Gray et al. 2001; Martin et al. 2002). Heterotrophic eukaryotes have, furthermore, evolved from an archaea-like ancestor through engagement of an alphaproteobacteria (related to Rickettsiales) in an event that marks the origin of mitochondria (Gray et al. 2001; Thrash et al. 2011). The evolution of phototrophic eukaryotes began with the acquisition of oxygenic photosynthesis by primary endosymbiosis in which a heterotrophic unicellular eukaryote acquired a cyanobacterium-like cell as a plastid (Hohmann-Marriott and Blankenship 2011). Phylogenetic studies have indicated that the primary endosymbiont was likely a close relative to filamentous heterocystous N_2_-fixing cyanobacteria (related to Nostocales; Deusch et al. 2008). Although some evidence suggests that primary endosymbiosis occurred only once in evolutionary history (McFadden and van Dooren 2004; Tirichine and Bowler 2011), ultrastructural, biochemical, and genetic analyses have revealed that secondary endosymbiosis arose multiple times (Kutschera and Niklas 2008; Nakayama and Ishida 2009). Given its extraordinary metabolic plasticity, it is not surprising that some of these characteristics of cyanobacteria seem to have been transferred into the heterotrophic eukaryote during this process leading to the evolution of algae and higher plants (Margulis 1970; Reyes-Prieto et al. 2007). Hence, many plant genes have originated from the cyanobacterial endosymbiont, including those coding for proteins involved in photosynthesis, respiration, and many other metabolic as well as regulatory functions (Martin et al. 2002; Kern et al. 2011).

Over the last decades, our exponentially increasing capacity for genome sequencing has generated vast amounts of sequence data of prokaryotic and eukaryotic providence. As such, novel opportunities to study the molecular evolution not only of individual enzymes but also of specific pathways consisting of several enzymatic reactions have now become possible. As mitochondria are organellar descendents of an alphaproteobacterial ancestor merged with a eukaryotic cell, it would be interesting to understand how mitochondrial pathways evolved and assembled during the course of the evolution. Although biochemical studies in the late 1960s have contributed much to our knowledge about the function of metabolic pathways and the regulation of metabolism, it was believed that cyanobacteria, like some other prokaryotes, harbored an incomplete tricarboxylic acid (TCA) cycle given that they lack the enzyme 2-oxoglutarate dehydrogenase (OGDH) (Pearce and Carr 1967; Smith et al. 1967). It was thought that the operation of the glyoxylate shunt or, alternatively, aspartate transaminase reactions functioned to complete this pathway (Steinhauser et al. 2012). Consequently, the presence of an incomplete TCA cycle had been used to explain various aspects of our current understanding of the physiology of cyanobacteria. However, compelling evidence has recently demonstrated that two other enzymes, namely 2-oxoglutarate decarboxylase (OGDC) and succinic semialdehyde dehydrogenase, are able to functionally substitute OGDH and succinyl-CoA ligase to generate reducing equivalents, thereby closing the cycle (Zhang and Bryant 2011). Remarkably, the presence of the OGDH complex in eukaryotic cells indicates that these genes were acquired by transfer events independent of the endosymbiotic process (Schnarrenberger and Martin 2002).

Recent advances have demonstrated that the TCA cycle intermediates play several key roles in cell physiology (for a review, see Araújo, Nunes-Nesi, et al. 2012) highlighting the plasticity of carbon metabolism in plants. Interestingly, many of the reactions in the TCA cycle can be bypassed by reactions resident in other subcellular compartments with only those catalyzed by succinyl-CoA ligase and succinate dehydrogenase (SDH) being unique to mitochondrion (Millar et al. 2011; Nunes-Nesi et al. 2013). Thus, all other enzymes are most likely encoded by paralogous genes and thus it is reasonable to assume that to accumulate so many functions within a cell, the number of TCA cycle genes increased in higher plants most likely due to multiple horizontal gene transfer and polyploidization events (Schnarrenberger and Martin 2002; Blanc et al. 2003). Accordingly, it has been assumed that genome reduction plays an important role during the endosymbiosis so that the mitochondrial genome shrinks relative to its bacteria ancestor (Gray et al. 2001). Furthermore, several genes belonging to the ancestral genome seem to have been transferred to the host genome by horizontal gene transfer (Dyall et al. 2004), whereas some of the remaining ones have been lost with their function replaced by host processes (Gabaldón and Huynen 2003). Thus, a selective set of metabolism pathways encoded by both mitochondrial genome and nuclear genome are most likely maintained in mitochondria to sustain its current functions.

We here investigated the evolutionary history of the TCA cycle based on phylogenetic analyses of all enzymes of the cycle and compared the trees obtained for individual enzymes searching for general patterns of phylogenetic similarities or discordance among them. We additionally analyzed the sequences of paralogous genes encoding TCA cycle enzymes and created a framework for coexpression analysis showing quantitative, temporal, and spatial differences among mitochondrial genes in both shoot and root tissues. Here, we present a comparative genomic study using robust phylogenetic analyses, including a vast number of taxa to validate an evolutionary model for the origin of TCA cycle genes in plants and to add novel insights into the TCA cycle relationship. Our results revealed tight connections among TCA cycle genes and that most of those genes originated after duplication events that occurred in plants. Additionally, we demonstrated that the emergence of different roles for TCA cycle genes, especially during suboptimal conditions in vascular plants, occurred through a process of neofunctionalization and/or subfunctionalization. These combined results are discussed in the context of the current models of the metabolic evolution and its connections providing clues to the understanding of the organization principles of mitochondria.

## Materials and Methods

### Data Mining for Nucleotides and Deduced Putative Protein Sequences of TCA Cycle Enzymes

Protein sequences were retrieved from GenBank through the pBLAST algorithm using all mitochondrial TCA cycle enzymes from *Arabidopsis thaliana* as query. Basic Local Alignment Search Tool (BLAST) searches were performed at National Center for Biotechnology Information nucleotide and protein database to search for sequences of TCA cycle enzymes in plants, mammals, and yeast. Additionally, data mining was performed in the cyanobacteria (CyanoBASE; http://genome.microbedb.jp/cyanobase, last accessed October 13, 2014) and *Escherichia coli* (http://genprotec.mbl.edu, last accessed October 13, 2014) genome databases in order to establish TCA cycle gene orthology to aid in understanding molecular evolution. When selecting the sequences we tried to include sequences from plants, animals, cyanobacteria, and fungi in addition to a representative sample of gene diversity and ancient family from eubacteria and archaebacteria. In some cases, homologs were not available from all sources. Sequences were aligned using the ClustalW software package (Higgins and Sharp 1988) using default parameters. Neighbor-joining trees (Saitou and Nei 1987) were constructed with MEGA5 software (Tamura et al. 2011) using midpoint rooting. Distances were calculated using pairwise deletion and Poisson correction for multiple hits, and bootstrap values were obtained with 1,000 pseudoreplicates.

Sequence data from this article can be found in the GenBank/EMBL databases under the accession numbers shown in supplementary table S1 and data sets S1–S4, Supplementary Material online.

### In *Silico Coexpression and Correlation Analysis of TCA Cycle Genes*

For gene expression analyses, the online tools of Genevestigator (http://www.genevestigator.com, last accessed October 13, 2014; Zimmermann et al. 2004) and e-Northens w.Expression browser (http://bar.utoronto.ca/affydb/cgi-bin/affy_db_exprss_browser_in.cgi, last accessed October 13, 2014; Toufighi et al. 2005) were used. The heat map was constructed using the obtained gene expression data sets with the software package TMEV (Saeed et al. 2003). Coexpression network analyses were performed using coefficient values calculated using the PRIMe coexpression database (Akiyama et al. 2008; http://prime.psc.riken.jp/?action=coexpression_index, last accessed October 13, 2014) calculated from publicly available microarray data. Connections between each gene were prepared by “interconection of sets” and “union of sets” searches. The criteria for interactome frameworks were performed at the cut-off value of 0.5 and 0.7, whereas criteria for correlation candidates were correlation values of 0.7, and *P* values < 0.05. Correlation networks were determined using Pearson’s correlation (*P* < 0.01). The output files which were formatted with “.net” file from PRIMe database were later used to drawn the networks using Pajek software (Batagelj and Mrvar 1998) (http://vlado.fmf.uni-lj.si/pub/networks/pajek/, last accessed October 13, 2014).

## Results and Discussion

**Fig. 1.—:**
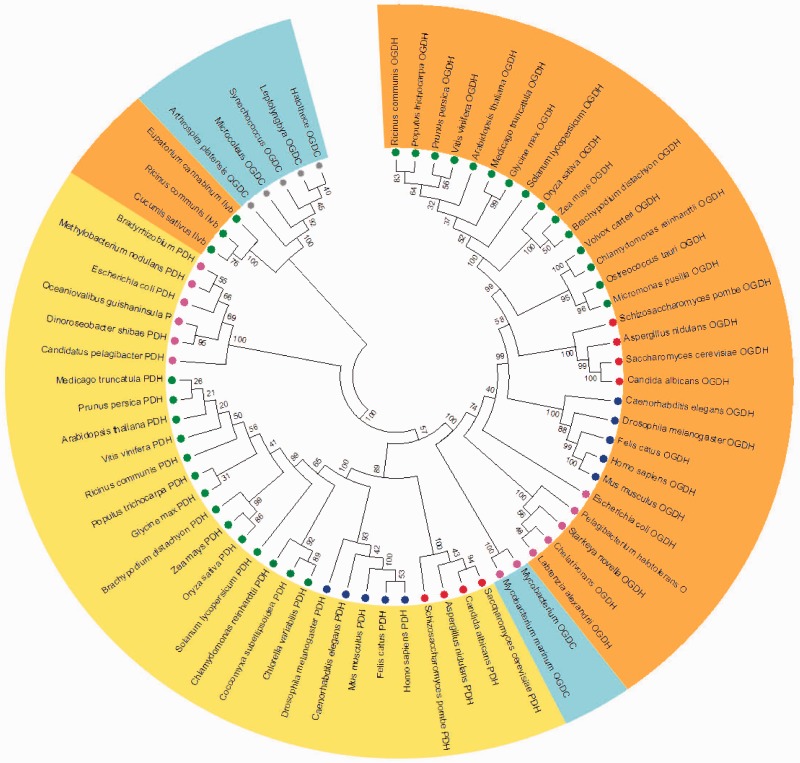
The distinct phylogeny of OGDH, PDH, OGDC, and OGDC. Phylogenetic tree was built using putative amino acids sequences from OGDH (orange background), PDH (yellow background), OGDC (blue background), and OGDC from cyanobacteria (green background) and acetolactate synthase. Sequences of putative proteins from plants are highlighted by green circle, yeast by red circle, animals by blue circle, algae by dark green circle, bacteria by pink circle, and cyanobacteria by gray cycle.

Due to the intrinsic complex structure of some TCA cycle enzymes consisting of multiple subunits (e.g., OGDH complex, succinyl-CoA ligase, and SDH), we analyzed each enzyme of the cycle individually by creating their respective phylogenetic trees attempting to infer the evolutionary history on an enzyme-by-enzyme basis. The only exception to this was the simultaneous phylogenetic analysis we conducted for the enzymes OGDH, pyruvate dehydrogenase (PDH), and OGDC (fig. 1). This construction was designed to facilitate the understanding of the evolutionary history of these enzymes of relatively similar function—indeed they share a common subunit. It has long been known that plant OGDH requires TPP, NAD^+^, and ADP (Bowman et al. 1976) and that the enzyme competes with PDH for intramitochondrial NAD^+^ and CoA (Dry and Wiskich 1987), the latter fact being of particular importance given that OGDH and PDH share a common subunit (E3). It is important to mention that range of studies have revealed that although OGDH is a key control point involved in the regulation of fluxes through the TCA cycle (Araújo, Nunes-Nesi, et al. 2012) the inhibition of PDH by light also reduces the TCA cycle flux (Randall et al. 1990; Tcherkez et al. 2011) allowing the elucidation of the precise physiological role of this enzymes. Although the evolution of these enzymes is somewhat complicated given that some organism used here present particular changes on the cycle structure associated with the absence of an OGDH and the presence of an alternative OGDC (Zhang and Bryant 2011), our results show that OGDC is phylogenetic closer to OGDH than to PDH clustering in the same branch as the former (fig. 1). Interestingly, in organisms as cyanobacteria there is no molecular evidence showing the presence of OGDH suggesting that TCA cycle was incomplete within this organism. However, an alternative to the lack of OGDH was recently found in cyanobacteria *Sycnechoccocus* sp which is able to synthesis succinate from 2-oxoglutarate (Zhang and Bryant 2011). Remarkable this alternative enzyme is the so-called OGDC which in *Sycnechoccocus* sp is encoded by SynPCC7002_A2770 gene requiring a second enzyme, SSADH, to synthesize succinate and complete the cycle (Zhang and Bryant 2011; Steinhauser et al. 2012). It should be mentioned that this OGDC reported in *Sycnechoccocus* sp is actually the acetolactate synthase gene which is phylogenetically distinct from both OGDH and OGDC from *Euglena gracilis* and *Mycobacterium* spp. (this group is branched between OGDH and PDH groups and it is highlighted by green background in fig. 1) even though this gene also encodes a TPP-dependent enzyme (fig. 1). Remarkably, the highly regulated production of 2-oxoglutarate by the action of the OGDH is involved in glucose oxidation through the TCA cycle occupying an amphibolic branch point in the cycle, where the energy-producing reaction of the 2-oxoglutarate degradation competes with glutamate synthesis through nitrogen incorporation into 2-oxoglutarate. As such, the importance of this step within the cycle is manifold: 1) OGDH is a key regulatory point allowing the flux of 2-oxoglutarate through TCA cycle (Zhang and Bryant 2011; Nunes-Nesi et al. 2013), 2) the synthesis of the essential amino acid lysine through the α-aminoadipate pathway requires 2-oxoglutarate (Kirma et al. 2012) and the synthesis of 2-oxoglutarate generates a branch linking this metabolite to other pathways such as amino acid biosynthesis (Araújo, Tohge, et al. 2012), and finally 3) the 2-oxoglutarate seems to be a connection between classical and alternative pathways of respiration in feeding electrons to the mitochondrial electron transport chain (Araújo et al. 2010).

Interestingly, we also observed the presence of one putative OGDC, most likely derived from cyanobacteria, in the chloroplasts of land plants but being demarcated as acetolactate synthase. It should be noted, however, that compelling evidence has shown that the endosymbiotic event of cyanobacteria by host cells culminated in plastid evolution in those plants (Keeling 2013). Notably, acetolactate synthase catalyzes the first enzymatic step in the synthesis of branched-chain amino acids (valine, leucine, and isoleucine) in plants (Chipman et al. 2005). Altogether this idea reinforces the hypothesis that the cyanobacterial TCA cycle performs a crucial function in producing precursor metabolites for biosynthetic reactions (Zhang and Bryant 2011) and that the usage of alternative pathways for respiration requires the presence of 2-oxoglutarate completing the cycle (Araújo et al. 2010). In addition, it is known that SSADH is active in mitochondrion of plant cells where it functions as an alternate succinate source, particularly under conditions of stress (Rocha et al. 2010; Sweetlove et al. 2010). Taken together, these results demonstrated a tight coordination of cell metabolism and indicate that during evolution the correct targeting of specific proteins allowed their subcellular compartmentation and specific functions in eukaryotes organisms.

### Citrate Synthase

**Fig. 2.—:**
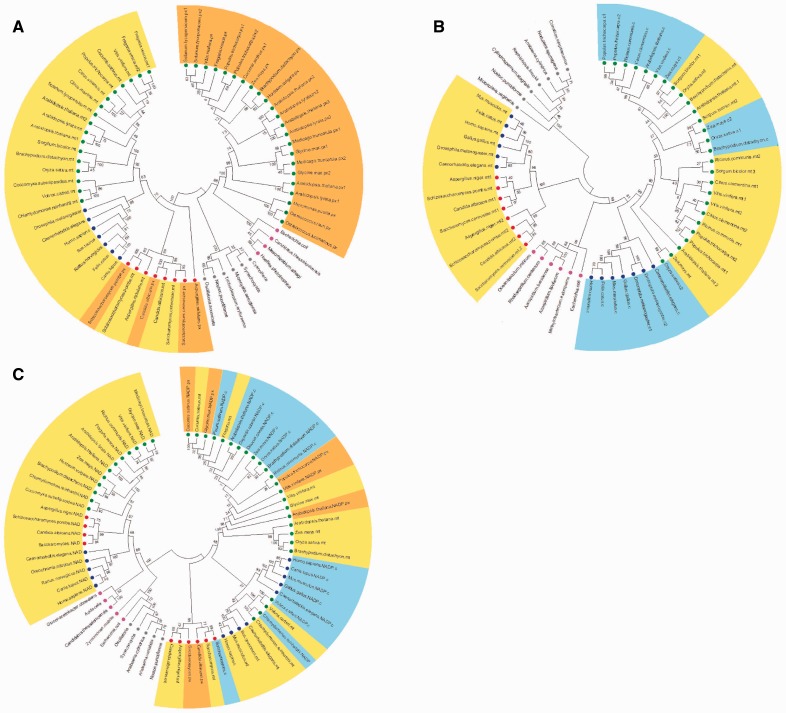
The phylogenetic tree of (*A*) CS, (*B*) aconitase, and (*C*) IDH. Trees were built using putative amino acids sequences from CS, aconitase, and IDH. Subcellular compartments are highlighted by background colors and in all cases figures follow the same pattern: Mitochondrial isoforms are highlighted by yellow background, peroxisomal isoforms by orange background, and cytosolic isoforms by blue background. Sequences of putative proteins from plants are highlighted by green circle, yeast by red circle, animals by blue circle, algae by dark green circle, bacteria by pink circle, and cyanobacteria by gray circle.

Citrate synthase (CS), which is often regarded as the first enzyme of the TCA cycle (Fernie et al. 2004), catalyzes the combination of oxaloacetate and acetyl CoA to produce citrate. This enzyme has been focus of many studies in plant–soil interactions suggesting that it is an important determinant of root citrate exudation (Kochian et al. 2004) and an important mediator of both phosphate uptake and aluminum tolerance (de la Fuente et al. 1997). The mitochondrial CS has also been suggested to play important roles in floral development (Landschutze et al. 1995) and as a source of carbon skeletons for nitrogen assimilation (Sienkiewicz-Porzucek et al. 2008). Although these studies have greatly enhanced our understanding of the role of CS in specific developmental and environmental interactions, they provide little information concerning the evolutionary history of isoforms of this enzyme. To enlighten this issue, a phylogenetic tree was constructed for CS isoenzymes (fig. 2*A*) and the sequences used clustered into two distinct groups related to mitochondrial or cytosolic isoforms. Interestingly, cytosolic isoforms of eukaryotic organisms are closer to CS of *E. coli*, which can be assumed as a sort of alpha proteobacteria related to be the mitochondrial ancestor, whereas mitochondrial isoforms were isolated in a distinct clade. This finding suggests that before the endosymbiotic event had occurred the eukaryotic ancestor already owned a CS gene and that CS family gene is clustered by horizontal gene transfer to mitochondria ancestor to host (eukaryotic cell) events.

### Aconitase

Aconitase catalyzes the reversible conversion of citrate into isocitrate through the formation of the intermediate product *cis*-aconitate. Two isoforms of this enzyme have been detected in land plants with the mitochondrial isoform being involved in the TCA cycle (Carrari et al. 2003), whereas the cytosolic participates in a range of processes such as citrate metabolism in the cytosol and the glyoxylate cycle (Hayashi et al. 1995). Interestingly in wild species tomato (*Solanum penelli*), the aconitase mutant allele (which corresponds to SlAco3b) is deficient in both cytosolic and mitochondrial aconitase proteins (Carrari et al. 2003), suggesting that at least in tomato this gene product is dual targeted. Therefore, we decided to analyze the evolutionary history of mitochondrial and cytosolic isoforms of a range of organisms (fig. 2*B*). As would perhaps be expected, regardless of the isoform plant aconitase was clustered together in our analyses. In contrast, the situation was quite different for animal aconitase, in which we could observe that the mitochondrial isoforms were quite different than the cytosolic isoforms, the latter being more similar to plant mitochondrial isoforms (fig. 2*B*). Noteworthy, although cyanobacterial aconitase formed a completely distinct and isolated cluster, yeast aconitase grouped with aconitase from animals. One possible explanation for this pattern is that cyanobacteria presents one aconitase type B (AcnB), which is functionally distinct from the others, whereas eukaryotic organisms and *Eschrichia coli* share cytosolic and mitochondrial aconitase type A (AcnA). Simultaneously, our results demonstrated that segregation between cytosolic and mitochondrial isoforms in eukaryotic organisms seems to have occurred during the course of the evolution and that it might be explained by independent horizontal gene transfer events.

Following the identification of the genes encoding aconitase, it has become apparent that although the number of genes encoding aconitase varies, particularly between plant species, with some having two and other three genes, the gene products are often dual targeted to both cytosol and mitochondria (Carrari et al. 2003; Arnaud et al. 2007; Morgan et al. 2013). It is important to mention that although this enzyme is highly sensitive to oxidative stress (Verniquet et al. 1991; Lehmann et al. 2009), the aconitase isoforms seem to differ in their relative sensitivity in which mitochondrial isoforms are likely more sensitive to oxidative stress than cytosolic isoforms in eukaryotic cells (Walden 2002). This fact coupled with the central point of this enzyme in the regulation of organic acid metabolism, aconitase seems to be a potential and suitable target for metabolic engineering applications. For instance, the recent combination of genetic and molecular approaches has demonstrated the crucial role of this enzyme in controlling organic acid content in ripe tomato fruit (Morgan et al. 2013), whereas in citrus fruit it was possible to manipulate the fruit acidity by changes in amino acid metabolism (Degu et al. 2011).

### Isocitrate Dehydrogenase

Isocitrate is oxidatively decarboxylated to 2-oxoglutarate by either NAD+ or NADP^+^-dependent isocitrate dehydrogenase (IDH), generating CO_2_ and NADH or NADPH, respectively (Lemaitre et al. 2007; Sienkiewicz-Porzucek et al. 2010; Sulpice et al. 2010). The function of this enzyme has been associated with the maintenance of the 2-oxoglutarate level and therefore the regulation of nitrogen assimilation (Stitt and Fernie 2003; Nunes-Nesi et al. 2010), as well as with the recycling of lysine during alternative respiratory pathways (Boex-Fontvieille et al. 2013) and in tolerance to biotic stress (Leterrier et al. 2012; Dghim et al. 2013). However, there is controversy concerning the evolutionary history of IDH regarding whether eukaryotic cells arisen of either a single progenitor gene or through independent duplications of an ancestor IDH gene within each kingdom (Schnarrenberger and Martin 2002; Hodges et al. 2003). Our results demonstrate that NAD^+^-dependent IDH is closer to alphaproteobacteria and cyanobacteria whereas NADP^+^-dependent IDH isoforms (e.g., peroxisome and cytosolic) cluster in other branches of our phylogenetic tree (fig. 2*C*). Thus our findings seem to be more related to the first hypothesis regarding IDH evolution (Schnarrenberger and Martin 2002) suggesting that the presence of at least one IDH gene ancestor for each kingdom is the most reasonable explanation for the different subcellular localization observed within this enzyme. This suggestion is further supported by the findings that for instance, IDH isoforms of plants and animals were grouped in isolated cluster branches of tree differently than what is shown here where NAD^+^-dependent IDH of animals and plants cluster in the same branch. On the other hand, peroxisome and cytosolic isoforms of IDH clustered in other branch and are much closer between them. One conspicuous feature concerning the role of IDH in human is a recurrent association with brain cancers and leukemia (Kranendijk et al. 2010; Tonjes et al. 2013) which has been associated with the accumulation of 2-hydroxyglutarate. The accumulation of this compound in dark-induced senescent mutant plants involved with alternative pathways of respiration (Araújo et al. 2010; Araújo, Ishizaki, et al. 2011; Engqvist et al. 2011) coupled with the recent association of this enzyme with lysine metabolism (Boex-Fontvieille et al. 2013) suggests that this enzyme might have yet further functions. Thus, returning to a more evolutionary perspective, altogether these data indicate that most likely IDH arises in two independent manners in eukaryotic cells: 1) Horizontal gene transfer of mitochondrion ancestor (NAD^+^-dependent) to host cells and 2) before endosymbiotic events, host cells have already obtained NADP^+^-dependent gene which further segregated in several isoforms. This dual idea is in good agreement with the several novel functions that have been associated with both cytosolic and mitochondrial isoforms of this surprisingly enzyme and highlights that further examination of this enzyme should provide significant insights into an integrated overview of the metabolic connections of this enzyme.

### Succinyl-CoA Ligase

Succinyl-CoA ligase catalyzes the reversible interconversion of succinyl-CoA to succinate using inorganic phosphate and dinucleotide to produce trinucleotide and CoA. It has been demonstrated that the inhibition of this enzyme in tomato plants leads to only minor changes in both respiratory and photosynthetic metabolism, most likely due to a compensatory upregulation of the GABA shunt (Studart-Guimarães et al. 2007). Characterization of the regulatory properties of this enzyme suggests that allosteric control regulating the flux through the TCA cycle would allow high cyclic flux in carbon rich times and reduced flux in times of carbon deficiency (Studart-Guimarães et al. 2007) suggesting that the succinyl-CoA enzyme may represent an adaptive mechanism in the attempt to maintain the rate of respiration under suboptimal conditions.

**Fig. 3.—:**
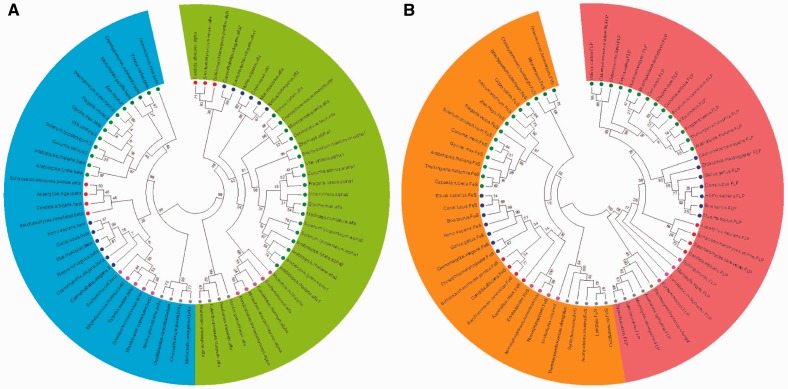
The phylogenetic tree of (*A*) succinl-CoA ligase and (*B*) SDH. Trees were built using putative amino acids sequences from (*A*) succinyl-CoA ligase highlighting the two subunits: Alpha (green background) and beta (blue background); and (*B*) SDH highlighting two subunits: Flavoprotein (red background) and iron-sulfur (orange background). Sequences of putative proteins from plants are highlighted by green circle, yeast by red circle, animals by blue circle, algae by dark green circle, bacteria by pink circle, and cyanobacteria by gray circle.

In yeast, the heterodimeric protein succinyl-CoA is encoded by two single-copy genes (Studart-Guimarães et al. 2005). Thus in an attempt to identify the pattern of evolution of this enzyme, we constructed phylogenetic trees using sequences ranging from yeast to mammalian proteins of both alpha and beta subunits encoded by different genes (fig. 3*A*) expecting that they had their own evolutionary history. One can easily note 1) a clear separation of alpha and beta subunits (different colors shown in fig. 3*A*) and 2) a wide segregation among kingdoms when analyzing succinyl-CoA ligase sequences which clearly suggest that eubacterial cells emerged from mitochondria ancestor and that succinyl-CoA ligase genes were most likely transferred to eubacterial genome. Another point to reinforce these suggestions is that although in higher plants there are two single copy genes encoding cytosolic and mitochondrial isoforms, these genes present high similarity between each other clustering in the same branch (e.g., *Solanum lycopersicum*) or in very close branches of sisters-species (e.g., alpha gene of *A. thaliana* and *Arabidopsis lyrata* are in the same cluster and the same happening with beta genes but in a different cluster) (fig. 3*A*).

### Succinate Dehydrogenase

SDH, also often referred to as complex II, has a dual function, being important in both the TCA cycle and the aerobic respiratory chain, through the catalysis of the oxidation of succinate to fumarate and the reduction of ubiquinone to ubiquinol, respectively (Affourtit et al. 2001; Araújo, Nunes-Nesi, et al. 2011). The conserved elements of this complex, a mere four polypeptides, comprise two peripheral membrane proteins—a flavoprotein (SDH1), and an iron-sulfur binding protein (SDH2)—as well as two integral membrane proteins (SDH3 and SDH4) (Rasmusson et al. 2008). However, the evolutionary history of these subunits is as yet unknown. For the sake of simplicity, multiple alignments of SDH sequences were carried out using only the flavoprotein and iron-sulfur subunits and the results obtained are shown in figure 3*B*. Although responsible for SDH function these subunits were relatively different and clustered in independent groups regardless the organisms evaluated here (fig. 3*B*). As it can be deduced by the data presented in figure 3*B* there are more similarity between plant and animal for each subunit than among cyanobacteria SDH subunits, indicating a mitochondrial origin for the eukaryotic gene. Although the proteins branch from the alpha proteobacterial homologs, it seems that the genes for SDH were most likely acquired from the mitochondrion ancestor. Altogether our data revealed a short divergence in terms of evolution and proximity of eukaryotic cells with the mitochondria ancestor. In good agreement with the view concerning the evolutionary history of SDH from mitochondria to nuclear genome, it has been demonstrated that at least some SDH genes were lost during horizontal gene transfer (Adams et al. 2002; Choi et al. 2006). Taken as a whole this feature might, at least partially, explain cross kingdom differences in the structural architecture of SDH (e.g., *A. thaliana* has 12 subunits; supplementary table S1, Supplementary Material online).

### Fumarase

Fumarase (or fumarate hydratase) catalyses the reversible hydration of fumarate to malate (Nunes-Nesi et al. 2007). Interestingly, although the mitochondrial fumarase seems to be an essential enzyme (Pracharoenwattana et al. 2010) characterization of a cytosolic fumarase mutant suggests that fumarate accumulation in the light is linked to nitrogen assimilation and increased starch in leaves of *A. thaliana* (Pracharoenwattana et al. 2010). Accordingly, fumarase activity has been shown to be high in guard cells of *Vicia faba* and *Pisum sativum* (Hampp et al. 1982; Outlaw 2003). Moreover and consistent with this observation, transgenic tomato plants with a reduced expression and activity of fumarase of up to 75% were characterized to have marginally elevated fumarate contents and that the reduced growth phenotype observed on a whole plant basis plants could be linked to impaired stomatal functioning (Nunes-Nesi et al. 2007), rather than a direct metabolic effect, resulting in CO_2_ limitation of photosynthesis. It was also observed that these transgenic plants had altered shoot to root resource partitioning (van der Merwe et al. 2009).

**Fig. 4.—:**
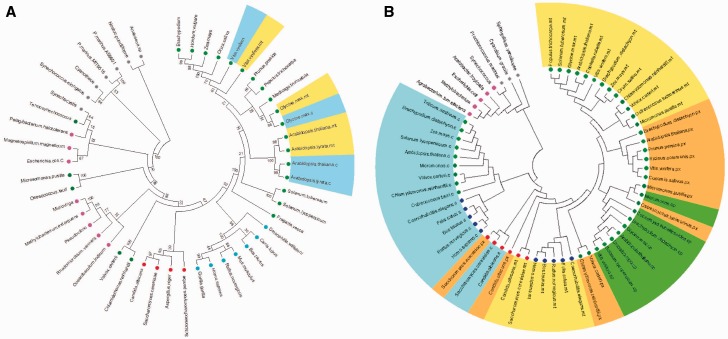
The phylogenetic tree of (*A*) fumarase and (*B*) MDH. Trees were built using putative amino acids sequences from (*A*) fumarase and (*B*) MDH. Subcellular compartments are highlighted as in figure 2: Mitochondrial isoforms are highlighted by yellow background, peroxissomal isoforms by orange background, and cytosolic isoforms by blue background. Sequences of putative proteins from plants are highlighted by green circle, yeast by red circle, animals by blue circle, algae by dark green circle, bacteria by pink circle, and cyanobacteria by gray circle.

Although the presence of a cytosolic fumarase has not yet been reported in Solanaceous species, the genome of both potato and tomato seems to encode these genes. In good agreement, data mining reveals that unlike most of other kingdoms, plants species harbor genes encoding both cytosolic and mitochondrial isoforms (fig. 4*A*). It should be mention that in some vertebrates, such as rat, the cytosolic enzyme is encoded by the same gene as the mitochondrial isoform which is generated by an alternative translation site (Suzuki et al. 1992). Additionally *Saccharomyces cerevisae* also harbors two enzymes located in the cytosol and mitochondrial due to different cleavage sites (Wu and Tzagoloff 1987). Nevertheless, fumarase gene of plants clustered in the Eudicots group suggesting the occurrence of a recent duplication event. In support to this point of view, paralogous genes are more similar to each other than to orthologous genes (e.g., *A. thaliana* mitochondrial and cytosolic genes cluster closer than the mitochondrial genes from *A. thaliana* and *Vitis vinifera*). Similar observations in terms of evolution of other mitochondrial genes families such as the *Arabidopsis* S-adenosylmethionine carrier SAMC1 (At4g39460) and SAMC2 (At1g34065) were also recently described (Palmieri et al. 2011) supporting our hypothesis that intraspecies paralogous were originate through recent duplication events within the Eudicot clade.

### Malate Dehydrogenase

The last step of the TCA cycle is catalyzed by malate dehydrogenase (MDH) and constitutes the reversible oxidation of malate to produce oxaloacetate (Nunes-Nesi et al. 2005). The mitochondrial isoform of this enzyme is important not only for NADH oxidation within the TCA cycle but is also responsible for the exchange of reducing equivalents between metabolic pathways in different cell compartments (Scheibe et al. 2005; Pracharoenwattana et al. 2007; Tomaz et al. 2010). Moreover, in *Arabidopsis* the number of nuclear genes that encode polypeptide components is 1, 3, 2, and 2 with localization in plastid, cytosol, mitochondria, and peroxisomes, respectively. Biochemical characterization of these subunits has revealed a broad connection of the MDH not only with respiration (Nunes-Nesi et al. 2005; Tomaz et al. 2010) but also with several other processes such as photorespiration, ß-oxidation of fatty acids, seed germination, and stress tolerance (Tesfaye et al. 2001; Pracharoenwattana et al. 2007; Cousins et al. 2008; Wang et al. 2010). Moreover, metabolic control coefficients of dark respiration have indicated that the control of leaf respiration is largely dominated by the enzyme MDH and that much of the control through the TCA cycle is shared between this enzyme, fumarase, and OGDH (Araújo, Nunes-Nesi, et al. 2012). Briefly, a central and complex role for this enzyme has been demonstrated suggesting its importance in the partitioning of carbon and energy in higher plants, providing new directions for bioengineering of plant growth rate and new insights into the molecular mechanisms linking respiration, photosynthesis, and photorespiration in plants (Cousins et al. 2008; van der Merwe et al. 2009; Tomaz et al. 2010).

Our data demonstrated that in order to play a range of role as described above it must be required that MDH has been spread across several subcellular compartments. Accordingly, we also had shown that MDH from cyanobacteria and bacteria clusters closer (fig. 4*B*). Moreover, our results indicate that cytosolic MDH most likely rises from horizontal gene transfer (fig. 4*B*). These findings are also in agreement with previous observation (Schnarrenberger and Martin 2002). Additionally, we also believe that other MDH isoforms had originated from cytosolic isoform (fig. 4*B*). This idea is highly interesting given that plastid isoforms clustered together in plants and green algae species suggesting that the plastid isoform of MDH arises after segregation between autotrophic and heterotrophic organisms (fig. 4*B*). Additionally, it seems highly possible that this plastid isoform (NADP^+^-dependent) was created through cytosolic (NAD^+^-dependent) isoform.

Collectively, our data indicated that the evolution of the TCA cycle was consistent with one step-by-step acquisition of each gene especially in free-living organisms which are the ancestor of mitochondrion. Accordingly, it is well known that paralogous genes often belong to the same species and cope with different evolutionary pressure arising either novel function of an existing gene (neofunctionalization) or in case of recent gene duplication similar function may still remain (subfunctionalization) (Force et al. 1999; Dani et al. 2014). Furthermore, paralogous sequence might provide useful insights into the way genome sequences evolve. For instance, although peroxisomal MDH is essential for ß-oxidation and seed germination (Pracharoenwattana et al. 2005) and has only a limited impact on photorespiration (Cousins et al. 2008), the mitochondrial MDH is important for both plant respiration and plays a key role on photorespiration regulating plant growth in *Arabidopsis* (Tomaz et al. 2010). In addition, overexpression of cytosolic MDH in alfalfa (*Medicago sativa*) increased aluminum tolerance through metal chelation in the soil (Tesfaye et al. 2001). These studies highlight a complex form of functional specialization between isoforms in different compartments and clearly show that changes in the amount of one specific isoform can have far reaching effects on plant growth and development. Surprisingly, although several studies have targeted on mitochondrial TCA cycle gene through reverse genetic approach or specific inhibitor (reviewed in (Araújo, Nunes-Nesi, et al. 2012; Nunes-Nesi et al. 2013) with a few exceptions our understanding of the role of other subcellular compartments isoforms has been mostly hampered (Pracharoenwattana et al. 2005, 2010; Sulpice et al. 2010) limiting our understanding of the complex evolution of this pathway. When considered together these results coupled with the ones we presented here strongly suggest that the TCA cycle most likely occurred in isolated steps in free-living organisms ancestor of mitochondrion (Schnarrenberger and Martin 2002; Gabaldón and Huynen 2003) and indicate that some enzymes (e.g., CS, MDH, and aconitase—discussed above) were acquired for eukaryotic cell by lateral gene transfer from several independent events using eubacteria donors.

### Interactions among Mitochondrial TCA Cycle Genes

The TCA cycle is composed by a set of eight enzymes presented in the mitochondrial matrix cycle coupling the product of the oxidation of pyruvate and malate (generated in the cytosol) to CO_2_ with the production of NADH by the respiratory chain (Fernie et al. 2004; Millar et al. 2011). It is known that genes encoding proteins that are involved in the same process, here meaning the TCA cycle, should be simultaneously expressed in time and space therefore here we choose coexpression data to aid in the discovery of patterns and novel players involved in this important process. Coexpression network analysis is commonly used to identify genes that have similar expression patterns and therefore represents an important tool to predict gene functionality using public transcriptome data sets. The availability of large amounts of gene expression data and the growing power of bioinformatics, coupled with availability of computational resources, opens new avenues to discover proteins involved in important processes, such as plant respiration.

**Fig. 5.—:**
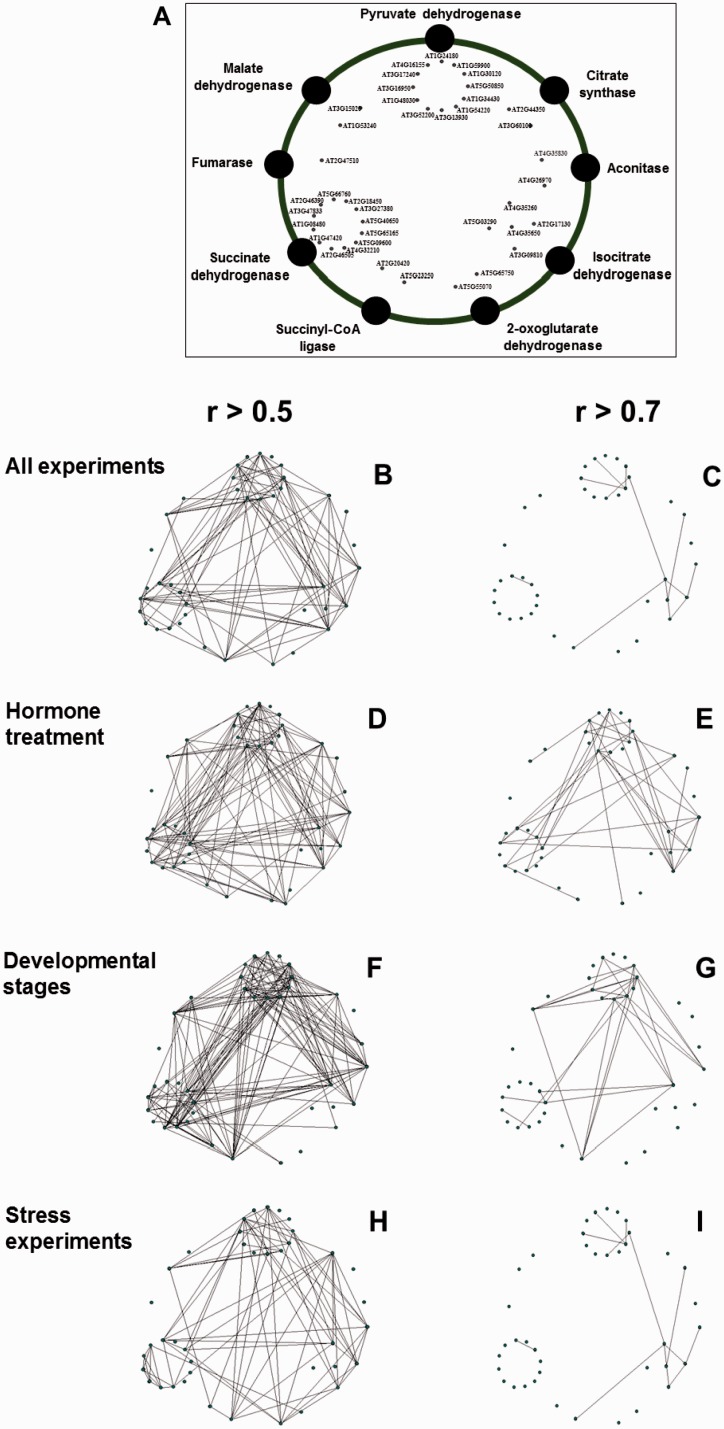
Coexpression analysis using mitochondrial genes of the TCA cycle from plant. (*A*) Framework for coregulation network analysis of mitochondrial isoforms of TCA cycle-related genes using the Pajek software. Transcriptional data mining was performed using coexpression PRIMe database (http://prime.psc.riken.jp/?action=coexpression_index, last accessed October 13, 2014) using as trap the genes listed in supplementary table S1, Supplementary Material online. Coresponse connection was performed using coefficient values calculated by a microarray data set of all experiments covering 1,388 microarray data (*B* and *C*); “hormone treatment” covering 326 data (*D* and *E*), “developmental stages” covering 237 data (*F* and *G*), and “stress experiments” covering 298 data (*H* and *I*). Analyses were performed with cut-off value of *r* > 0.5 for (*B)*, (*D*), (*F*), and (*H*) and of *r* > 0.7 for (*C*), (*E*), (*G*), and (*I*). Connections between each genes were prepared by “intersection of sets “search. The output files which were formatted with “.net” file from PRIMe database were later used to drawn the networks using Pajek software (Batagelj and Mrvar et al. 1998). Abbreviations and locus names of genes used are available in supplementary table S1, Supplementary Material online.

Our first in silico coexpression method was performed using all genes or a large group of genes that are computed by coexpression responses for different sets of microarrays data (Akiyama et al. 2008) using separate data sets of hormone (fig. 5*A* and *B*), developmental changes (fig. 5*C* and *D*), and stress responses (fig. 5*G* and *H*). Interestingly, both hormone treatment and developmental change data sets demonstrated a high and intense regulation within the TCA cycle genes when compared with stress experiment (fig. 5). This observation can be expected in the light of, for instance, recent results showing a strong association between hormone and energy metabolism (Araújo, Tohge, et al. 2012; Ribeiro et al. 2012a, 2012b) as well as evidence concerning that GA might be able to modify primary metabolism at the entry point of TCA cycle (Yazaki et al. 2003; Jan et al. 2006). Although the results highlighted above provide clear support to the role of energetic metabolism and particularly the TCA cycle as a central through in supplying ATP and other fundamental metabolites to support growth and development, our current understanding of the general role of hormones in the regulation of plant metabolism and growth is still limited and deeply deserving further investigation.

**Fig. 6.—:**
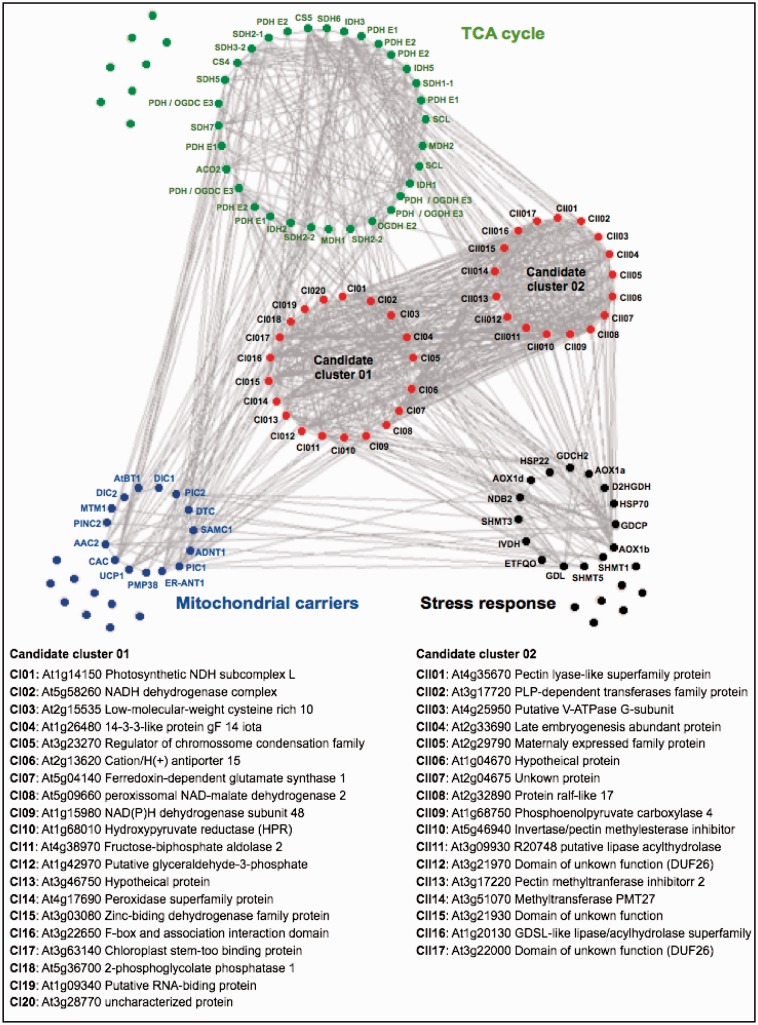
Coexpression analysis as a tool to identify candidate genes involved on mitochondrial metabolism. Framework for coregulation network analysis (*r* > 0.70) of 84 preselected known genes encoding 40 mitochondrial TCA cycle (green circle), 24 mitochondrial carriers (blue circle), and 22 stress response (black circle) genes using coexpression PRIMe database and Pajek software. The candidate genes were listed by a combinatorial method of “intersection of sets” and “interconnection of sets” using the PRIMe website (http://prime.psc.riken.jp/, last accessed October 13, 2014). Candidate genes were found by an “intersection of sets” search with a threshold value with a coefficient of *r* > 0.70 queried by intraconnection between all query genes. A coexpression network, including candidate genes (37 genes) and queried genes (84 genes), was reconstructed by a “union of sets” search with *r* > 0.70 using the PRIMe database. The output files that were formatted with a “.net” file from the PRIMe database and networks were drawn using Pajek software (http://vlado.fmf.uni-lj.si/pub/networks/pajek/, last accessed October 13, 2014). For a complete description of the gene names, see supplementary table S2, Supplementary Material online.

In order to further develop our analysis, we conducted an expanded guide-gene approach that used interconnections between sets of specific guide genes of related biological processes (fig. 6). For this end, we used three sets of “bait genes” known to be involved in plant TCA cycle (40 genes), mitochondrial carrier (24), and stress responses and photorespiratory genes (22) aiming to identify further candidate genes involved in the mechanism of regulation of plant respiration (supplementary table S2, Supplementary Material online). As would perhaps be expected, this coexpression revealed close connections between these candidate genes. Furthermore, we were able to identify two connected cluster of coexpressed genes that could be clearly separated. First, we identified genes in Candidate Cluster I (20 genes) which coexpressed with all processes making them logical candidates for further investigations. It should be mention that the some genes in the Candidate Cluster II (17 genes) correlated better with the TCA cycle and mitochondrial stress-related genes, whereas some specific genes within the Candidate Cluster I seem to highly coexpressed with mitochondrial carriers. One interesting feature of the network that has been generated is that among the genes present in the Candidate Cluster I we found several genes involved in photosynthesis or photorespiration which are located in other subcellular compartments suggesting a close association of mitochondrial related processes with other processes within the plant cell—as has been noted in many experimental studies (Carrari et al. 2003; Nunes-Nesi et al. 2005, 2007; Tcherkez et al. 2005; Sweetlove et al. 2006; Tomaz et al. 2010; Araújo, Nunes-Nesi, et al. 2011; Foyer et al. 2011; Timm et al. 2012; Boex-Fontvieille et al. 2013). Thus although mitochondrial genes such as NAD(P)H dehydrogenase (At5g58260) and genes involved in redox balance control (At5g04140; ferredoxin-dependent glutamate synthase 1) were present among our candidate genes, other uncharacterized genes classified as unknown or hypothetical were coexpressed suggesting that further investigation of mitochondrial metabolism might identify other as yet unknown connections within the TCA cycle. Further functional characterization of these genes will likely help us in identifying genes that control and regulate plant respiration as well as facilitating the discovery of novel gene functions with potential biotechnological applications.

### Physiological Importance of Mitochondrial TCA Cycle Genes under Suboptimal Conditions

**Fig. 7.—:**
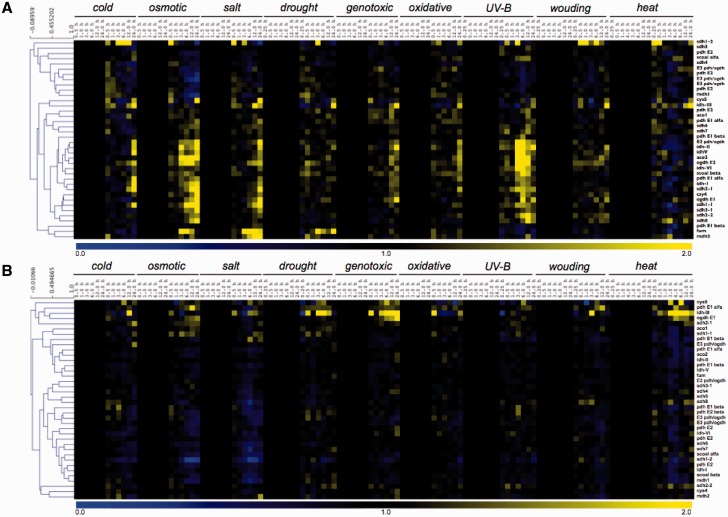
Coexpression analysis of genes encoding TCA cycle genes found in *A. thaliana* under a range of stress situations. Heatmap of gene expression data and clustering of the corresponding gene expression data set retrieved of The Bioarray Resource for Plant Biology (BAR, http://bar.utoronto.ca/welcome.htm, last accessed October 13, 2014) using e-Northerns w. Expression Browser platform was performed with MultiExperiment Viewer software (MeV software; Saaed et al. 2003) using “AtGenExpress—Stress Series” data set. The analysis was performed with 48 TCA cycle-related genes of *A. thaliana* using the expression in (*A*) shoot and (*B*) root tissues. For a complete description of the gene names, see supplementary table S1, Supplementary Material online.

Although plant respiration is mainly dependent on carbohydrate oxidation (Plaxton and Podesta 2006; Araújo, Nunes-Nesi, et al. 2012), it has been recently demonstrated that the oxidation of alternative substrates becomes considerably important during various stress conditions, which affects carbohydrates supply (Ishizaki et al. 2005, 2006; Engqvist et al. 2009; Araújo et al. 2010). Additionally, plant metabolism is highly reorganized under a range of different stress conditions including salt, cold, drought, and oxidative stress (Kaplan et al. 2004, 2007; Gong et al. 2005; Sanchez et al. 2008, 2012; Alet et al. 2012; Siahpoosh et al. 2012), allowing plants to continue to produce indispensable metabolites while preventing the accumulation of reactive oxygen species. Thus, we here postulate that the functionally translated portion of the genome plays an essential role in plant stress, and therefore extended bioinformatic studies will likely provide a finer picture of protein networks involved in metabolic pathways that are important for cellular detoxification and tolerance mechanisms. As such, a prominent topic in plant mitochondria research involves linking mitochondrial composition and function to environmental stress responses. In order to understand transcriptional changes of genes encoding TCA cycle proteins, we evaluated data from AtGenExpress concerning a wide range of stress including exposure to cold, osmotic, salt, drought, genotoxic, oxidative, UV-B, wounding, and heat stress displayed in a heat map documentation of the relative expression of 40 TCA cycle-related genes in both shoot and roots (fig. 7). Interestingly, the majority of the genes whose function has been experimentally investigated showed strong transcriptional regulation following stress situation, either in root or in shoot tissue. Notably, the expression profile observed in shoots under different stress conditions seems to be independent of the changes observed in roots (fig. 7*A* and *B*, respectively). For instance, when the expression profiles of all gene copies of IDH (with the exception of idh3, discussed below) were compared distinct patterns are apparent in roots and shoots. Although in roots only a moderate decrease in expression was observed after 1 and 3-h exposure to osmotic and salt stresses, respectively, in shoots gene expression is highly upregulated by cold, osmotic, salt, wounding, and heat. Differential patterns of expression between shoot and root were also observed for MDH 2, fumarase, and the majority of the SDH subunits under osmotic and UV-B stresses at least 1 h after stress application (fig. 7). Thus, as would be expected that given the profile previously observed for genes encoding mitochondrial carriers (Palmieri et al. 2011), the pattern of expression of the TCA cycle-related genes under the stress conditions appears to be more affected by environmental changes in shoots than in roots.

Our analysis also revealed a molecular plasticity in transcripts level of the TCA cycle genes between the tissues analyzed (fig. 7). As such, although most of the TCA cycle-related genes are upregulated in shoots (fig. 7*A*) these genes are normally downregulated in roots under a range of stresses (fig. 7*B*). Thus, in roots a small group of genes seem to be stress-induced involving six TCA cycle-related genes in the same cluster (fig. 7*B*). These genes are citrate synthase 5 (cs5), aconitase 1 (aco1), isocitrate dehydrogenase 3 (idh3), 2-oxoglutare dehydrogenase (ogdh) E1 and E2 subunits, and succinate dehydrogenase 1-1 (sdh1-1). Interestingly, from all IDH used here only NAD^+^-dependent idh-3, which is strictly associated with the TCA cycle (Nunes-Nesi et al. 2013), showed an augmentation in its expression (fig. 7*B*). In addition, the transcript of this IDH isoform clearly increased after exposure for at least 3 h to drought stress. Indeed, under abiotic stresses situation such as drought, oxidative, and salt stress augmentation in the transcript levels of ogdh and sdh1-1 as well as idh3 was observed in root tissues. Whether these genes have at least partial redundant functions, particularly under stress situations, or whether the similar pattern of expression due to any transcription factor is unknown.

In contrast to the situation observed in roots, the TCA cycle-related genes were highly upregulated under various stresses in shoots (fig. 7*A*). Notably, when comparing the data obtained within upregulated genes, we can observe that the mitochondrial fumarase gene is highly upregulated in response to both drought and salt stresses, which are stresses well known to impact photosynthesis due to, in several cases, stomatal closure (Deikman et al. 2012; Daszkowska-Golec and Szarejko 2013). In close agreement, and as discussed above, antisense inhibition of fumarase leads to decreased photosynthesis and biomass in tomato plants associated with impairment in stomatal function (Nunes-Nesi et al. 2007), whereas antisense inhibition of the iron-sulfur subunit of SDH in tomato plants culminated in higher photosynthesis rate as well as increased whole plant biomass (Araújo, Nunes-Nesi, et al. 2011). These results provided strong evidence to support that modulation of malate and fumarate concentration through genetic manipulation of the mitochondrial metabolism can greatly influence stomatal function and photosynthesis itself in an abscissic acid independent manner. Similarly, during salt stress, fumarase seems to be strongly coexpressed with the mitochondrial MDH 2 (fig. 7*A*). In good agreement with our putative data showing an increase of fumarase and MDH 2 genes during various stresses, it has been recently demonstrated that after 6 h of salt treatment there was a reduction and increase on MDH 2 transcripts and protein amount, respectively, whereas fumarase transcripts and proteins are both reduced (Jacoby et al. 2011). This increment of mitochondrial MDH protein abundance following 6 h of salt treatment suggests that MDH 2 displays rapid responses to stress. Accordingly, salt stress acts decreasing water potential of soil leading to physiological effects that are similar to those seen during drought stresses (Munns 2002); however, in this case it is reasonable to assume that malate has a dual role being 1) required for stomatal closure as discussed above or 2) used as substrate by MDH to form NADH and subsequently ATP synthesis by mitochondrial electron chain transfer in order to relieve cellular damages caused by salt stress.

Our data additionally demonstrated that UV-B stress leads to significant reductions in the expression of genes from the second half of the TCA cycle, such as fumarase and MDH simultaneously to increments on transcripts level of genes from the first half of the TCA cycle in shoot tissue (fig. 7*A*). This finding is in a good agreement with recent experimental evidence using UV-B stress under shoot tissue of *Arabidopsis* (Kusano et al. 2011). Accordingly, intermediates of the first half of the TCA cycle are deviated for the phenylpropanoid pathway reducing flux through the TCA cycle (Kusano et al. 2011) suggesting the operation of a modular TCA cycle in response to UV-B.

### Concluding Remarks and Outlook

Although it is clear that coexpression analysis can be used to associate genes with certain biological functions, the remaining question is how reliable these predictions are which means, do the genes identified by coexpression analysis truly function together? There are currently several examples available in which such an approach has been successfully used to identify genes not previously associated with a given biological question (for reviews see Usadel et al. 2009; Bordych et al. 2013, and references therein). We are aware that coexpression analysis, performed individually, is not sufficient to yield lists of candidate genes that are short enough to be investigated in vivo. Nevertheless, as shown in our work, this analysis can be assumed as a reasonable first step to provide initial suggestion of putative candidates. We have no doubts that the coexpression approach has opened new venues for plant researchers and it seems to be true also for the TCA cycle in plants. The coming years will see many more gene expression data sets from specific cell types and other species, which should dramatically accelerate precise hypothesis generation in plant biology (Usadel et al. 2009; Bordych et al. 2013) and particularly within the TCA cycle.

Altogether our coexpression analyses coupled with phylogenetic trees provided an evolutionary explanation for the modular operation of the TCA cycle according with physiological conditions. This is most likely due to the fact that prior endosymbiotic events some TCA enzymes were already present in eubacterial host and these enzymes seem to have being interconnected to other pathways during evolution, what explain, at least partially, the diversity of function for the isoforms of the TCA cycle enzymes. Notably, endosymbiotic process allowed that other enzyme closed the cycle at the same time that other pathways continued associated with TCA cycle and giving even more functions to this highly important and specialized pathway.

**Fig. 8.—:**
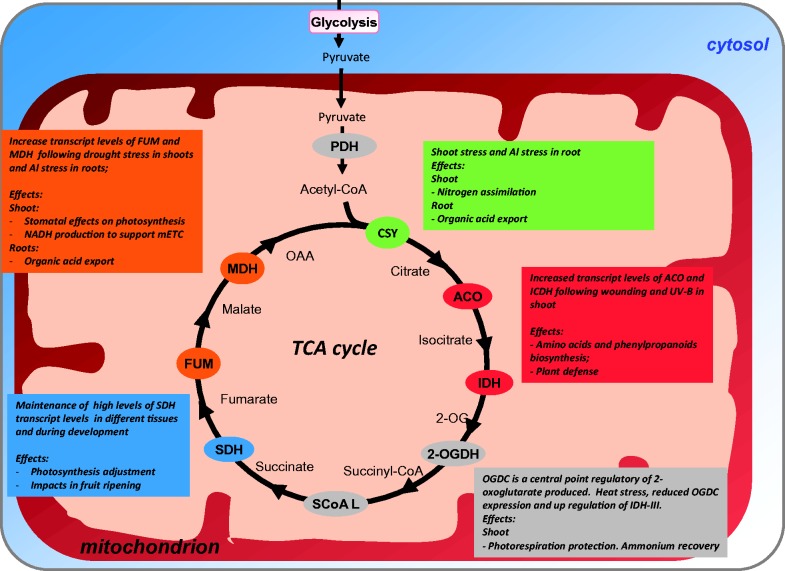
Schematic summary representation of tricarboxylic acid (TCA) cycle and its possible strategic for the metabolic engineering. By using bioinformatics approaches presented in figures 6 and 7 a number of possible research avenues for metabolic engineering with the TCA cycle enzymes are presented and discussed in the text. Abbreviations: citrate synthase (CS), aconitase (ACO), isocitrate dehydrogenase (ICDH), 2-oxoglutarate dehydrogenase complex (OGDC), succinyl-CoA ligase (SCoA L), succinate dehydrogenase (SDH), fumarase (FUM), and malate dehydrogenase (MDH).

Accordingly, increasing our understanding on the evolution, organization, and function of the TCA cycle members seems to be an interesting way to address endosymbiotic gene transfer. Our results demonstrated that this was most likely the case for several of the mitochondrial enzymes of the TCA cycle, which clearly created a range of biochemical associations of this important pathway with others running in different cell compartments (fig. 8). It will thus be interesting in future studies to evaluate these processes temporally and simultaneously, to dissect both the cascades that control them and the consequence of energy-related processes on the life cycle of the plant. Notwithstanding this fact, the results here also illustrate that mitochondrial metabolism and particularly the TCA cycle in shoots are more dramatically altered with respect to a range of stress. Further work is still required to fully establish the mechanisms involved in this response; however, it is clear that the diverse biochemical phenotypes that have been observed (discussed above) cannot be explained in terms of the operation of a classical TCA cycle, as suppression of any of the enzymes would be expected to reduce the cyclic flux to a greater or lesser extent and thus lead to similar consequences (Sweetlove et al. 2010). Similar conclusions have previously been drawn in microbial and mammalian systems (Tian et al. 2005; Singh et al. 2009; Lemire et al. 2010), allowing us to postulate that the different steps in the TCA cycle have functions other than maintenance of cyclic flux, and that the fine metabolic balancing between these functions is likely to depend on the physiological context in which the pathway is operating. That being said, the far greater alterations observed in TCA cycle-related genes render both the cycle itself and the mitochondrial metabolism as whole as important targets for metabolic engineering and/or breeding strategies for the generation of plants capable of performing well in the future global climate changes scenario (fig. 8).

## Supplementary Material

Supplementary data sets S1–S4 and tables S1 and S2 are available at *Genome Biology and Evolution* online (http://www.gbe.oxfordjournals.org/).

Supplementary Data
