# Supplementary material for: Evolution and Functional Implications of the Tricarboxylic Acid Cycle as Revealed by Phylogenetic Analysis
Source: Genome Biol Evol. 2014 Oct 1;6(10):2830–48. doi: 10.1093/gbe/evu221 (PMC4224347; doi:10.1093/gbe/evu221)
Supplement: Supplementary Data [file supp_6_10_2830__index.html]

Evolution and functional implications of the tricarboxylic acid cycle as revealed by phylogenetic analysis — Evolution and Functional Implications of the Tricarboxylic Acid Cycle as Revealed by Phylogenetic Analysis — Supplementary Data 

# Evolution and Functional Implications of the Tricarboxylic Acid Cycle as Revealed by Phylogenetic Analysis

## Supplementary Data

files

**Files in this Data Supplement:**

- Supplementary Data - pdf file
- Supplementary Data - doc file
- Supplementary Data - doc file
- Supplementary Data - doc file
- Supplementary Data - doc file
