# Supplementary material for: Evolution and Functional Implications of the Tricarboxylic Acid Cycle as Revealed by Phylogenetic Analysis
Source: Genome Biol Evol. 2014 Oct 1;6(10):2830–48. doi: 10.1093/gbe/evu221 (PMC4224347; doi:10.1093/gbe/evu221)
Supplement: Supplementary Data [file supp_evu221_STable1_2.pdf]

Supplemental Table S1. Number and locus of nuclear genes encoding TCA cycle related genes in *Arabidopsis thaliana* and their subcellular localization. Genes retrieved from TAIR, NCBI and KEGG database.

	Number of genes	Locus	Subcellular localization
**Pyruvate Dehydrogenase Complex**	14	AT1G01090	chloroplast
		AT1G24180	mitochondria
		AT1G59900	
		AT2G34590	chloroplast
		AT1G30120	mitochondria
		AT5G50850	
		AT1G34430	mitochondria
		AT1G54220	
		AT3G13930	
		AT3G52200	
		AT1G48030	mitochondria
		AT3G16950	
		AT3G17240	
		AT4G16155	
**Citrate Synthase**	5	AT3G58740	peroxissome
		AT3G58750	
		AT2G42790	
		AT2G44350	mitochondria
		AT3G60100	
**Aconitase**	3	AT2G05710	cytosol
		AT4G35830	mitochondria
		AT4G26970	
**Isocitrate Dehydrogenase**	7	AT4G35260	mitochondria
		AT2G17130	
		AT4G35650	
		AT3G09810	
		AT5G03290	
		AT1G65930	cytosol
		AT1G54340	peroxissome
**2-Oxoglutarate Dehydogenase Complex**	3	AT5G65750	mitochondria
		AT5G55070	
		**equal of E3 PDC**	
**Succinil-CoA Ligase**	3	AT5G08300	cytosol
		AT5G23250	mitochondria
		AT2G20420	
**Succinate Dehydrogenase**	12	AT5G66760	mitochondria
		AT2G18450	
		AT3G27380	
		AT5G40650	
		AT5G65165	
		AT5G09600	
		AT4G32210	
		AT2G46505	
		AT1G47420	
		AT1G08480	
		AT3G47833	
		AT2G46390	
**Fumarase**	2	AT5G50950	cytosol
		AT2G47510	mitochondria
		AT1G04410	cytosol
		AT5G43330	
		AT5G56720	

**Malate Dehydrogenase**	8	AT1G53240	mitochondria
		AT3G15020	
		AT2G22780	peroxissome
		AT5G09660	
		AT3G47520	chloroplast

Supplemental Table 3. List of candidate gene co-expressed involved in mitochondrial response associated with the mitochondrial TCA cycle, carriers and stress response genes.

Locus		Function
Cluster 0I		
CI01	AT1G14150	Photosynthetic NDH subcomplex L
CI02	AT5G58260	NADH dehydrogenase complex
CI03	AT2G15535	Low-molecular-weight cysteine rich 10
CI04	AT1G26480	14-3-3-like protein GF 14 iota
CI05	AT3G23270	Regulator of chromossome condensation family
CI06	AT2G13620	Cation/H(+) antiporter 15
CI07	AT5G04140	Ferredoxin-dependent glutamate synthase 1
CI08	AT5G09660	peroxissomal NAD-malate dehydrogenase 2
CI09	AT1G15980	NAD(P)H dehydrogenase subunit 48
CI10	AT1G68010	Hydroxypyruvate reductase (HPR)
CI11	AT4G38970	Fructose-biphosphate aldolase 2
CI12	AT1G42970	Putative glyceraldehyde-3-phosphate
CI13	AT3G46750	Hypotheical protein
CI14	AT4G17690	Peroxidase superfamily protein
CI15	AT3G03080	Zinc-biding dehydrogenase family protein
CI16	AT3G22650	F-box and association interaction domain
CI17	AT3G63140	Chloroplast stem-too binding protein
CI18	AT5G36700	2-phosphoglycolate phosphatase 1
CI19	AT1G09340	Putative RNA-biding protein
CI20	AT3G28770	uncharacterized protein
Cluster II		
CII01	AT4G35670	Pectin lyase-like superfamily protein
CII02	AT3G17720	PLP-dependent transferases family protein
CII03	AT4G25950	Putative V-ATPase G-subunit
CII04	AT2G33690	Late embryogenesis abundant protein
CII05	AT2G29790	Maternaly expressed family protein
CII06	AT1G04670	Hypotheical protein
CII07	AT2G04675	Unkown protein
CII08	AT2G32890	Protein ralf-like 17
CII09	AT1G68750	Phosphoenolpyruvate carboxylase 4
CII10	AT5G46940	Invertase/pectin methylesterase inhibitor
CII11	AT3G09930	R20748 putative lipase acylthydrolase
CII12	AT3G21970	Domain of unkown function (DUF26)
CII13	AT3G17220	Pectin methyltranferase inhibitorr 2
CII14	AT3G51070	Methyltransferase PMT27
CII15	AT3G21930	Domain of unkown function
CII16	AT1G20130	GDSL-like lipase/acylhydrolase superfamily
CII17	AT3G22000	Domain of unkown function (DUF26)
