# Supplementary material for: Evolution and Functional Implications of the Tricarboxylic Acid Cycle as Revealed by Phylogenetic Analysis
Source: Genome Biol Evol. 2014 Oct 1;6(10):2830–48. doi: 10.1093/gbe/evu221 (PMC4224347; doi:10.1093/gbe/evu221)
Supplement: Supplementary Data [file supp_evu221_Supplemental_DataSet_1.doc]

E1: 2-oxoglutarate dehydrogenase

>Arabidopsis thaliana (NM_125972.4)

MVWFRIGSSVAKLAIRRTLSQSRCGSYATRTRVLPCQTRCFHSTILKSKAESAAPVPRPVPLSKLTDSFLDGTSSVYLEELQRAWEADPNSVDESWDNFFRNFVGQASTSPGISGQTIQESMRLLLLVRAYQVNGHMKAKLDPLGLEKREIPEDLTPGLYGFTEADLDREFFLGVWRMSGFLSENRPVQTLRSILSRLEQAYCGTIGYEYMHIADRDKCNWLRDKIETPTPRQYNSERRMVIYDRLTWSTQFENFLATKWTTAKRFGLEGAESLIPGMKEMFDRSADLGVENIVIGMPHRGRLNVLGNVVRKPLRQIFSEFSGGTRPVDEVGLYTGTGDVKYHLGTSYDRPTRGGKHLHLSLVANPSHLEAVDPVVIGKTRAKQYYTKDENRTKNMGILIHGDGSFAGQGVVYETLHLSALPNYCTGGTVHIVVNNQVAFTTDPREGRSSQYCTDVAKALSAPIFHVNADDIEAVVHACELAAEWRQTFHSDVVVDLVCYRRFGHNEIDEPSFTQPKMYKVIRSHPSSLQIYQEKLLQSGQVTQEDIDKIQKKVSSILNEEYEASKDYIPQKRDWLASHWTGFKSPEQISRIRNTGVKPEILKNVGKAISTFPENFKPHRGVKRVYEQRAQMIESGEGIDWGLGEALAFATLVVEGNHVRLSGQDVERGTFSHRHSVLHDQETGEEYCPLDHLIKNQDPEMFTVSNSSLSEFGVLGFELGYSMENPNSLVIWEAQFGDFANGAQVMFDQFISSGEAKWLRQTGLVVLLPHGYDGQGPEHSSGRLERFLQMSDDNPYVIPEMDPTLRKQIQECNWQVVNVTTPANYFHVLRRQIHRDFRKPLIVMAPKNLLRHKQCVSNLSEFDDVKGHPGFDKQGTRFKRLIKDQSGHSDLEEGIRRLVLCSGKVYYELDEERKKSETKDVAICRVEQLCPFPYDLIQRELKRYPNAEIVWCQEEPMNMGGYQYIALRLCTAMKALQRGNFNDIKYVGRLPSAATATGFYQLHVKEQTDLVKKALQPDPITPVIP

>Ricinus communis (XP_002528465.1)

MAWFRAGASVARLAIRRTLSQSGSYTVRTRVVPSQNRYFHTTVFKSKAQAAPVPRPVPLSRLTDSFLDGTSSVYLEELQRAWEADPSSVDESWDNFFRNFVGQAATSPGISGQTIQESMRLLLLVRAYQVNGHMKAKLDPLGLEEREIPEDLDPALYGFAEADLDREFFLGVWRMSGFLSENRPVQTLRSILTRLEQAYCGSIGYEYMHIADRDKCNWLRDKIETPTPMQYNRQRREVILDRLIWSTQFENFLATKWTTAKRFGLEGGETLIPGMKEMFDRSADLGVESIVIGMPHRGRLNVLGNVVRKPLRQIFSEFSGGTKPVDEVGLYTGTGDVKYHLGTSYDRPTRGGKRIHLSLVANPSHLEAVDPVVVGKTRAKQYYSNDEDRIKNMGILIHGDGSFAGQGVVYETLHLSALPNYSTGGTIHIVVNNQVAFTTDPTAGRSSQYCTDVAKALNAPIFHVNGDDVEAVVHACELAAEWRQTFHSDVVVDLVCYRRFGHNEIDEPSFTQPKMYQVIRNHPSSLQIYKNKLLESGQVGEEDISRIQEKVITILNEEFLASKDYVPKRRDWLSSHWAGFKSPEQLSRIRNTGVQPEILKNVGKAITTIPDNFKPHRAVKKVYEQRAQMIETGEGIDWAVAEALAFATLLVEGNHVRLSGQDVERGTFSHRHSVIHDQETGEKYCPLDHVIMNQNEEMFTVSNSSLSEFGVLGFELGYSMESPNSLVIWEAQFGDFSNGAQVIFDQFLSSGESKWLRQTGLVVLLPHGYDGQGPEHSSARLERFLQMSDDNPCVIPEMEPTLRKQIQECNWQVVNVTTPANYFHVLRRQLHRDFRKPLIVMAPKNLLRHKDCKSNLSEFDDVQGHPGFDKQGTRFKRLIKDQNDHSDLEEGIRRLVLCSGKVYYELDEERKKIGAKDVAICRVEQLCPFPYDLIQRELKRYPNAEIVWCQEEPMNMGAYNYIAPRLCTAMKALERGSVEDIKYVGRAPSAATATGFYQVHVKEQSELVQKAMQPEPIHYPF

>Populus trichocarpa (XP_002315242.1)

MAWFRAGSGVARLAIRRTLSQGGSYATRSRVIPPQNRYFHSTVFKSKAQAAPVPRPVPLSKLTDNFLDGTSSVYLEELQRAWETDPNSVDESWDNFFKNFVGQAATSPGISGQTIQESMRLLLLVRAYQVNGHMKAKLDPLGLEEREIPDDLDPALYGFTDADLDREFFLGVWRMAGFLSENRPVQTLRAILTRLEQAYCGSIGYEYMHIADREKCNWLRDKIETPTSMQYNRQRREVILDRLIWSTQFENFLATKWTTAKRFGLEGGETLIPGMKEMFDRSADLGVESIVIGMPHRGRLNVLGNVVRKPLRQIFSEFSGGTKPVDEVGLYTGTGDVKYHLGTSYDRPTRGGKRIHLSLVANPSHLEAVDPVVVGKTRAKQYYSNDSDRTKNMGILIHGDGSFAGQGVVYETLHLSALPNYTTGGTIHIVVNNQVAFTTDPRAGRSSQYCTDVAKALNAPIFHVNGDDMEAVVHVCELAAEWRQTFHSDVVVDLVCYRRFGHNEIDEPSFTQPKMYQVIRNHPSALEIYKKKLLESGQVTEEDISRIQEKVLSILNEEFLASKDYVPKRRDWLSSHWTGFKSPEQLSRVRNTGVKPEILKNVGKAITTFPENFKPHRAVKKVYEQRLQMIETGEGIDWAVGEALAFATLLVEGNHVRLSGQDVERGTFSHRHSVVHDQETGEKYCPLDHVVMNQDEEMFTVSNSSLSEFGVLGFELGYSMESPNSLVIWEAQFGDFANGAQVIFDQFLSSGESKWLRQTGLVLLLPHGYDGQGPEHSSARLERFLQMSDDNPYVIPEMEPTLRKQIQECNWQVVNVTTPANYFHVLRRQIHRDFRKPLVVIAPKNLLRHKECKSNLSEFDDVQGHPGFDKQGTRFKRLIKDRNDHSDLEEGIRRLVLCSGKIYYELDEVRGKVEAKDIAICRVEQLCPFPYDLIQRELKRYPNAEIVWCQEEPMNMGAYNYIAPRLSTAMKALERGTVDDIKYVGRGPSAASATGFYQVHVKEQTELVQMAMQPEPIKFPN

>Prunus persica (EMJ22109.1)

MTWFRAGSSVAKLAIRRTLSHSGSYAGRRRVLPSQNRDFHTTLCKSKAQSAPVPRPVPLSRLTDSFLDGTSSVYLEGLQRAWEADPNSVDESWDNFFRNFVGQASTSPGISGQTIQESMRLLLLVRAYQVNGHMKAKLDPLGLEEREIPDDLDPALYGFTEADLDREFFLGVWRMAGFLSENRPVQTLRSILTRLEQAYCGTIGYEYMHIADRNRCNWLRDKIETPTPMQYNRQRREVILDRLIWSTQFENFLATKWTAAKRFGLEGGETLIPGMKEMFDRAADLGVESIVIGMSHRGRLNVLGNVVRKPLRQIFSEFSGGTKPVDEVGLYTGTGDVKYHLGTSYDRPTRGGNRIHLSLLANPSHLEAVDPVVVGKTRAKQYYSSDPDRTKNVGILIHGDGSFAGQGVVYETLHLSALPNYTTGGTIHIVVNNQVAFTTDPMSGRSSQYCTDVAKALNAPIFHVNADDMEAVVHVCELAAEWRQTFHSDVVVDLVCYRRFGHNEIDEPSFTQPKMYKVIRNHPSALTIYQNKLLESGQVTKEDIERIQNKVNSILNEEFLASKDYVPQRRDWLSSHWSGFKSPEQISRIRNTGVKPEILKSVGKAVTSLPETFKPHRAVKKNYEQRAQMIETGEGIDWAVAEALAFATLLVEGNHVRLSGQDVERGTFSHRHSVVHDQETGERYCPLDHIMANQDEEMFTVSNSSLSEFGVLGFELGYSMESPNALVIWEAQFGDFANGAQVIFDQFLSSGESKWLRQTGLVVLLPHGYDGQGPEHSSARLERFLQMSDDNPFVIPEMDPTLRKQIQECNWQVVNVTTPANYFHVLRRQLHREFRKPLIVMAPKNLLRHKECKSNLSEFDDVQGHPGFDKQGTRFKRLIKDQNDHSNLEEGIRRLVLCSGKLYYELDEERRKVEAKDVAICRVEQLCPFPYDLIQRELKRYPNAEIVWCQEEPMNMGAYSYIAPRLCSAMKSLGRGTIEDIKYVGRAPSAATATGFYQVHVKEQNEIVHKAVQPEPIEYHI

>Solanum lycopersicum (XP_004236757.1)

MAWFRAGSSVAKLAIRRAVSQGGSYVPRTRIIPSQSRYFHTTVVRPKAQAAPVPRPVPLSKLTDSFLDGTSSVYLEELQRAWEQDPSSVDESWDNFFRNFTGLAATSPGISGQTIQESMNLLLLVRAYQVNGHLKAKLDPLDLEERDIPDVLDPVSYGFTEADLDREFFLGVWRMAGFLSENRPVQTLRAILTRLEQAYCGSIGFEYMHISDRDKCNWLRERIETPTPREYNRERREVILDRLMWSTQFENFLATKWVAAKRFGLEGCETLIPGMKEMFDRSADLGVESIVIGMPHRGRLNVLGNVVRKPLRQIFSEFTGGTKPADGAGYVGTGDVKYHLGTSYDRPTRGGKRIHLSLVANPSHLEAVDPVVIGKTRAKQYYSNDVDRTKNMGILLHGDGSFAGQGVVYETLHLSALPNYTTGGTIHIVVNNQVAFTTDPKAGRSSQYCTDVAKALSAPIFHVNGDDVEGVVYACELAAEWRQTFHSDVVVDIVCYRRFGHNEIDEPSFTQPKMYQVIRNHPSSLEIYQNKLLQHGQVTKDDVEKIHNKINTILNEEFVASKDYVPQKRDWLSAFWSGFKSPAQLSRVRNTGVKPEILKDVGKAITSLPDDFKPHRAVKRIFDDRKKMIETGEGVDWAVGEALAFATLLVEGNHVRLSGQDVERGTFSHRHSVVHDQETGAKYCPLDHVMMNQNEEMFTVSNSSLSEFGVLGFELGYSMENPNSLVLWEAQFGDFANGAQVIFDQFLSSGEAKWLRQSGLVVLLPHGYDGQGPEHSSARLERFLQMSDDNPYVIPDMEPTLRKQIQECNLQVVNVTTPANYFHVLRRQIHRDFRKPLIVMSPKNLLRHKDCKSNLSEFDDVQGHPGFDKQGTRFKRLIKDQNDHSDLEEDIRRLVLCSGKVYYELDEERKKVEGKDVAICRVEQLCPFPYDLVQRELKRYPNAEIVWCQEEPMNMGAYHYIAPRLSTAMKALNRGNVDDIKYVGRAPSAATATGFYQVHVKEQTELVQKALQQDPISSPV

>Vitis vinifera (XP_002279332.2)

MVWFRVGSGVGSVAKHAIRRTLCQGGSYATRTRVLPSQNRYFHSTVLKSKAQAAPVPRPVPLSRLTDSFLDGTSSVYLEELQRAWEADPNSVDESWDNFFRNFVGQAATSPGISGQTIQESMRLLLLVRAYQVNGHMKAKLDPLGLEEREIPDDLDPALYGFTEADLDREFFLGVWRMAGFLSENRPVQTLRAILTRLEQAYCGSIGYEYMHIADRDKCNWLRDKIETPTPRQYNQQRREVILDRLIWSTQFENFLATKWTAAKRXXXXXXTGDVKYHLGTSYDRPTRGGRRIHLSLVANPSHLEAVDPVVVGKTRAKQYYSNDLDRTKNIGVLIHGDGSFAGQGVVYETLHLSALPNYTTGGTIHIVVNNQVAFTTDPRSGRSSQYCTDVAKALNAPIFHVNGDDMEAVVHVCELAAEWRQTFHSDVVVDVVCYRRFGHNEIDEPSFTQPKMYKVIRNHPSALEIYQKKLLELGQATQEDIDRVQNKVNTILNEEFLASKDYVPNRRDWLSAYWAGFKSPEQISRVRNTGVRPEILKNVGKAITTLPENFKAHRAVKKIFDLRAQMIETGEGIDWAVGEALAFATLLVEGNHVRLSGQDVERGTFSHRHSVIHDQETGERYCPLDHVIMNQNEEMFTVSNSSLSEFGVLGFELGYSMENPNALVMWEAQFGDFANGAQVIFDQFLSSGESKWLRQTGLVVLLPHGYDGQGPEHSSARLERFLQMSDDNPYVIPEMDPTLRKQIQECNWQIVNVTTPANYFHVLRRQIHREFRKPLIVMSPKNLLRHKDCKSNLSEFDDVQGHPGFDKQGTRFKRLIKDQNDHSSLEEGIRRLILCSGKVYYELDEQRKKVKANDVAICRVEQLCPFPYDLAQRELKRYPNAEIVWCQEEPMNMGAYNYILPRLCTAMKEVDRGTVEDVKYVGRAPSAATATGFSSLHTKEQTELVQKALQPEPINYPF

>Medicago truncatula (XP_003621819.1)

MAWFRAGASIAKNAIKRTISQNRSSSYLVSRSRILPSTHGRKFHTTVFKQQAAPVPRAVPLSRLTDSFLDGTSSVYLEELQRAWEADPNSVDESWDNFFRNFVGQASTSPGISGQTIQESMRLLLMVRAYQVNGHMKAKLDPLNLEARQIPDDLDPALYGFSEADLDREFFLGVWRMAGFLSENRPVQTLRSILTRLEQAYCGSIGFEYMHISDREKCNWLRDKIETPTPVQFNRERREAIFDRLAWSSLFENFLATKWTSAKRFGLEGGETLIPGMKEMFDRASDLGVESIVIGMAHRGRLNVLGNVVRKPLRQIFCEFSGGLSPEDEVGLYTGTGDVKYHLGTSYDRPTRGGKRIHLSLVANPSHLEAVDPVVVGKTRAKQYYSNDVDRTKNMGILLHGDGSFAGQGVVYETLHLSALPNYTTGGTIHIVLNNQVAFTTDPMSGRSSQYCTDVAKALDAPIFHVNGDDVEAVVHACELAAEWRQTFHSDVVVDLVCYRRFGHNEIDEPSFTQPKMYKVIRSHPSTLEIYQKKLLETGELTQDDIDKIHKKVTSILNEEFQASKDYIPKRRDWLSAYWLGFKSPEQLSRVRNTGVKPEILKTVGKAITTIPENFTPHKAVKRIYEQRAQMIETGEDIDWGFGEALAFATLLVEGNHVRLSGQDVERGTFSHRHSVVHDQTTGEKYCPLDNVILNQDEEMFTVSNRYAHISILSMSVFETICSSLSEFAVLGFELGYSMENPNSLIIWEAQFGDFANGAHVIFDNFLASGEAKWLRQTGLVVLLPHGYDGQGPEHSSARLERFLQMADDNPYIIPEMDPTLRKQIQECNLQIVNVTTPANFFHVLRRQIHREFRKPLIVMSPKNLLRSKSCRSNLSEFDDVQGHPGFDKQGTRFKRLIKDRNDHNTVEEGIRRLVLCSGKVYYELDDHRSKVDASDVAICRVEQLCPFPYDLVQRELKRYPNAEVVWCQEEPMNMGGYTYILPRLVSSMKAVGRGGFDDVKYVGRAPSAATATGFLKVHQKEQAEIAEKALQREPVNFPF

>Glycine max (XP_003518655.1)

MAWFRAGTSIAKHAIRRTLSKGGSTYLVSRARFLPSIPSSSSSPYSRSFHSTVFKEQAAPVPRAVPLSKLTDSFLDGTSSVYLEELQRAWEADPNSVDESWDNFFRNFVGQATTSPGISGQTIQESMRLLLLVRAYQVNGHMKAKLDPLNLEPRQISEDLDPALYGFTEADLDREFFLGVWRMAGFLSENRPVQTLRSILTRLEQAYCGSIGYEYMHIADRHKCNWLRDKIETPTPTQFNRERREAIFDRLAWSSLFENFLATKWTSAKRFGLEGGETLIPGMKEMFDRASDLGVESIVIGMAHRGRLNVLGNVVRKPLRQIFCEFSGGLQPEGEVGLYTGTGDVKYHLGTSYDRPTRGGKRIHLSLVANPSHLEAVNPLVIGKTRAKQYYANDVDRTKNMGVLIHGDGSFAGQGVVYETLHLSALPNYTTGGTIHIVFNNQVAFTTDPTSGRSSQYCTDVAKALDAPIFHVNGDDVEAVVHACELAAEWRQTFHSDVVVDLVCYRRFGHNEIDEPSFTQPKMYKVIRSHPSTLEIYEKNLLESGELTQEEIDRIHKKVTSILNEEFLASKEYIPKRRDWLSAYWLGFKSPEQLSRIRNTGVKPEILKTVGKAITTIPENFTPHRAVKRIYEQRAQMIETGEDIDWGFAEALAYATLLIEGNHVRLSGQDVERGTFSHRHAVVHDQTTGEKYCPLDHVIMNQDEEMFTVSNSSLSEFGVLGFELGYSMENPNSLIIWEAQFGDFANGAHVIFDNFLASGEAKWLRQTGLVVLLPHGYDGQGPEHSSARLERFLQMADDNPHVIPEMDPTLRKQIQECNLQIVNVTTPANFFHVLRRQVHRDFRKPLIVMSPKNLLRSKACRSNLSEFDDVQGHPGFDKQGTRFKRLIKDQNAHKDVEEGIRRLVLCSGKVYYELDEQRTKVDANDVAICRVEQLCPFPYDLVQRELKRYPNAEVVWCQEEPMNMGGYTYVLPRLISSMKAVNRGGYDDVKYIGRAPSAATATGFLKVHQKEQTEIAEKAVQQEPIDFPF

>Zea mays (NP_001169698.1)

MGLFRAASGLARLALRRNLSRAAASPFAGSGGAVPGAMPARYFHSTRPRRFAAPAPRAVPLSRLTDSFLDGTSSVYLEELQRAWEADPNSVDESWDNFFRNFVGQAAATSPGLSGQTIQESMRLLLLVRAYQVSGHLKAKLDPLGLEERPVPDVLDPGFYGFSEADLDREFFLGVWMMAGFLSENRPVQTLRSVLERLEQAYCGTIGYEYMHIPDREKCNWLRDRIETVNPREYTYDRRQVMLDRLIWSTQFENFLATKWTTAKRFGLEGAETLIPGMKEMFDRAADLGVESIVIGMPHRGRLNVLGNVVRKPLRQIFSEFSGGTKPVNEGEGLYTGTGDVKYHLGTSYDRPTRGGKHIHLSLVANPSHLEAVDPVVAGKTRAKQYYSNDRDRTKNLGVLLHGDGSFSGQGVVYETLHLSALENYTTGGTIHIVVNNQVAFTTDPRSGRSSQYCTDVAKALDAPIFHVNGDDLEAVVHVCELAAEWRQTFHSDVVVDIVCYRRFGHNEIDEPSFTQPKMYKVIRNHPSALEIYQRKLLESGKISKEDIDKLNKKVSTILNEEFQNSKDYVPNKRDWLSAYWTGFKSPEQISRIQNTGVKPEILKRVGEAMTTLPENFNPHRAVKKIFYQRRQMIETGEGIDWAVGEALAFATLIIEGNHVRLSGQDVERGTFSHRHSVLHDQETGEQYCPLDHLVMNQDEELFTVSNSSLSEFAVLGFELGYSMENPNSLVIWEAQFGDFSNGAQVIFDQFLSSGESKWLRQTGLVVCLPHGYDGQGPEHSSARLERFLQMSDDNPYVIPEMDPTLRKQIQQCNWQVVNVTTPANYFHVLRRQIHRDFRKPLIVMSPKNLLRHKDCKSNLSEFDDLAGHPGFDKQGTRFKRLIKDQNNHKDLEEGINRLVLCSGKVYYELDEERRKTERTDVAICRVEQLCPFPYDLIQRELKRYPNAEIVWCQEEPMNMGAYSYINPRLLTAMKALGRGGIEDIKYVGRAPSAATATGFYSVHVQEQTELVQKALQRDPINYPF

>Oryza sativa (EEC77180.1)

MGWFRAASGLARVALRRNLARAPANPFAGPAPRYFHSTRPRRFAAPVPRAVPLSRLTDSFLDGTSSVYLEELQRAWEADPTSVDESWDNFFRNFVGQAATSPGISGQTIQESMRLLLLVRAYQVSGHLKAKLDPLALEERPIPDVLDPAFYGFSEADLDREFFLGVWRMAGFLSENRPVQTLRSVLERLEQAYCGTIGYEYMHIPDREKCNWLRDRIETVNAREYSYDRRQVMLDRLMWSTQFESFLAQKWTTAKRFGLEGAETLIPGMKEMFDRAADLGVESIVIGMPHRGRLNVLGNVVRKPLRQIFSEFSGGTKPAEEGEGLYTGTGDVKYHLGTSYDRPTRGGKHIHLSLVANPSHLEAVDPVVAGKTRAKQYYSNDLDRTKNLGVLLHGDGSFSGQGVVYETLHLSALPNYTTGGTIHIVVNNQVAFTTDPRAGRSSQYCTDVAKALDAPIFHVNGDDLEAVVHVCELAAEWRQTFHSDVVVDIVCYRRFGHNEIDEPSFTQPKMYKIIRNHQSALEIYQNRLLESGKISKEDIDKMQKKVSTILNDEFQNSKEYIPNKRDWLSAYWTGFKSPEQISRIRNTGVKPEILKRVGEAMTTLPENFKPHRAVKKIFELRRQMIETGEGIDWAVGEALAFATLIIEGNHVRLSGQDVERGTFSHRHAVIHDQETGEQYCPLDNLVMNQDEELFTVSNSSLSEFAVLGFELGYSMENPNSLVLWEAQFGDFSNGAQVIFDQFLSSGEAKWLRQTGLVVCLPHGYDGQGPEHSSARLERFLQMSDDNPYVIPEMDPTLRKQIQQCNWQVVNVTTPANYFHVLRRQIHRDFRKPLIVMSPKNLLRHKDCKSNLSEFDDLAGHPGFDKQGTRFKRLIKDQNNHKDLEEGIKRLVLCSGKVYYELDEERRKKERDDVAICRVEQLCPFPYDLIQRELKRYPNAEIVWCQEEPMNMGAYSYINPRLLTAMRALGRGTIDDIKYVGRAPSAATATGFYSVHVQEQTELVQKALQRDPINSPF

>Brachypodium distachyon (XP_003579623.1)

MGWFRAASGLARVALRRNLSRVPASPFAGPAPRYFHSTRPRRFAAPEPRAVPLSRLTDSFLDGTSSVYLEELQRAWEADPSSVDESWDNFFRNFVGQAATSPGISGQTIQESMRLLLLVRAYQVSGHMKAKLDPLGLEERPVPDVLDPAFYGFSEDDLDREFFLGVWKMAGFLSENRPVQTLRSVVERLEQAYCGTIGYEYMHIPDREKCNWLRERIETVNPREYTYDRRQVMLDRLIWSTQFENFLAQKWTTAKRFGLEGAETLIPGMKEMFDRAADLGVESIVIGMPHRGRLNVLGNVVRKPLRQIFSEFSGGTKPVNEGEGLYTGTGDVKYHLGTSYDRPTRGGKHIHLSLVANPSHLEAVDPVVAGKTRAKQYYSNDLDRTKNLGVLLHGDGSFSGQGVVFETLHLSALPNYTTGGTIHIVVNNQVAFTTDPMSGRSSQYCTDVAKALDAPIFHVNGDDLEAVVHTCELAAEWRQTFHSDVVVDIVCYRRFGHNEIDEPSFTQPKMYKIIRNHPSALEIYQKQLLESGKLSKEDIDKLHKKVSTILNEEFQKSKDDIPNKRDWLSAYWTGFKSPEQISRIRNTGVKPEILKRVGEAMTTLPETFKPHRAVKKIFDLRRQMIETGEGIDWAVGEALAFATLIVEGNHVRLSGQDVERGTFSHRHSVIHDQETGEQYCPLDNLVMNQNEELFTVSNSSLSEFAVLGFELGYSMENPNSLVLWEAQFGDFSNGAQVIFDQFISSGEAKWLRQTGLVVCLPHGYDGQGPEHSSARMERFLQMSDDNPYVIPEMDPTLRKQIQQCNWQVVNVTTPANYFHVLRRQIHRDFRKPLIVMSPKNLLRHKECKSSLSEFDDLAGHPGFDKQGTRFKRLIKDRNDHKDLEEGINRLVLCSGKVYYELDEERKKLDRNDVAICRVEQLCPFPYDLIQRELKRYPNAEIVWCQEEPMNMGAYTYINPRLLTAMKALSRGSIEDIKYVGRAPSAATATGFYSVHVQEQTELVQKALQKDPIKGPF

>Ostreococcus tauri (XP_003078135.1)

MTLAIARRRLCALARHPRALVAVARDGASRASTVGTIGRDWPIEREFATKSVGSGAFPSLKRARATVERSLAVQTAHAREKATMSSTAAPKPTPVREMRDEFLNATSAAYLEAMEDDFRRDPKSVPESWAMLLRQMDSGVTGAEISDMHNAALTGTAPHAVGRPLDAQTIQESMRLMLLIRAYQTSGHAAARLDPLGLDKREGIIYLEPALYGFSEDDMDREFFIGTWKMQGFLSEDRPVQSLRQILTRLQDTYCGTIGYEYMHIQDRDQCNWLRSKIETERKKQYSTERKRIILDRLSWSELFENFLSNKYSAAKRFGLEGCESLVPGFKEAIDKAAEMGVENITIGMPHRGRLNVLANVVRKPLQTIFNEFKGGPKLVEELGNAGSSYTGSGDVKYHLGTSFDRPTLRGGQIHLSVVANPSHLEAVNTVVTGKTRAKQFYTKDPKGERSMAVLLHGDGAFSGQGIVYETLDMSKLPEYQVGGTLHIVVNNQVAFTTDPKYSRSSPYCTDVAKGMEVPIFHVNGDDVEAVAWVMELATEWRMKWKTDAVVDIVCYRKYGHNEIDEPMFTQPLMYKVIQKHPSALTQYSNKLIDDGTVTPEEVMEMRNRINSKMEEEFNSSKDYVPKQRDWLSSHWQGFKSPDQLSRIRDTGLPPEHLKNLGNLITTIPAGFTPHRVVKRVYEARRAMIDNGEGLDWAMGEALAFASLLDDGNHVRLSGQDVERGTFSHRHALLHDQITGERFIPLRNVYSGNMGRGRDFFTVCNSSLSEYGVLGFELGYSLEHPNALILWEAQFGDFANTAQVIIDQFISSGEAKWLRQSGLVMLLPHGYDGQGPEHSSARLERFLQMTDEDPTRIPEMSMEKRTQLQECNWQICNVTTPANYFHMLRRQVHREFRKPLVVMSPKNLLRHPKAVSKLNEFDNSDENDSLQGIRFKRLIMDKTSTSRSLDPPPQPEVDRVIFCSGKVYYDLDDARDKASKLDKVKICRIEQLAPFPWDLVQRELKRYPNAEVVWCQEEPMNMGAWTHVQARMSTLFDHLERPGRTRYAGRKPAASPATGYAAVHAQEQAQLRPGARNRRRLVIITGRRHAGRDHDWKIPRVGADLGVGDLRKTPRNTRNVHL

>Volvox carteri (XP_002950899.1)

MALAAAAKRAAAEGLPGILCILRLGRLGVHTTSSAFNPAEPKPVPLAKLKDSFNEGTSITYLEELEERYHRDPSSVDRTWQAFFRNLDQGVSGEAMAEAFDAFEKGKLHMSPFSAAAVSNQTVQESMRLLLLIRAYQVLGHFAADLDPLGISGHAHPPELDPSFWGFKETDLDREFYIGNWNQAGFLAEGRPMRTLREMLTRLQETYCSNIGYEYMHIPERDKCNWIRERIETIDKVQFTKAQKQHMLDRLAWSDMFETFLANKYTAAKRFGLEGAESLIPGMKTIIDTAADLGVQSVVIGMPHRGRLNVLANVVRKPLAQIFSEFTGKMHEAHEGEYTGSGDVKYHLGTSYNRPTVNGKMVHLSLVANPSHLEAVNTVVLGKTRGKQYYSDDHERLRNMAILLHGDGAFSGQGIVYETLDMSGLPDYTVGGTIHLVVNNQVAFTTDPKDSRSSPYCTDVAKSLNCPIFHVNADDVESVVRVCQLAAEWRQAWKSDVVVDLVCYRKHGHNEIDEPMFTQPLMYKKIKAKKHSHEMYAERLLEEGTFTKEEIRAIHDKIQSLLNESFEAAKDYKPQKKDWLASHWHGFMSPAQMSRIRNTGVPADLLRTVGHAITALPEDFIAHRQIRKVYEQRRSMVDTGEGVDWAMAEALAFATLLSEGNHVRLSGQDVERGTFSHRHAVLHDQNTGGKYVPLNHVFPGQTNNSFTVCNSSLSEFGVLGFELGYSMESPNSLVLWEAQFGDFANGAQIIFDQFLSSGEAKWLRQSGLVCLLPHGYDGQGPEHSSARLERFLQMCDENPYDMPHHDEAQWFTGGHLGTQIQRANWQVVNCTTPANYFHVLRRQVHRQFRKPLIVMSPKNLLRHPMCKSPLREFDDQPDDANIVGVRFKRVIMDDTGLLPKDRGPRPPQEPDVKRVVFCSGKVFYDLHAEREKQGKEGVVAIVRVEQLAPFPFDLVCREIRRYPNAQLLWCQEEPMNMGAYLHVQPRFDTCLREEGKPMLGRMPYAGRPPMAATATGFGDVHAREQAKLIKDALDVNYCYP

>Chlamydomonas reinhardtii (XP_001692870.1)

MSRRGMSPALRLWPIAKSVASLASARCTPAAWNPAEPKPVPLAKLKDSFNEGTSITYLEELEERYHKDPASVDRSWQAFFRNLDHGVTGEAMAESFDAFEKGKLAMSPFTAAAISNQTVQESMRLLLMIRAYQVLGHFAADLDPLRISGHTHPPELDPAFWGFKDTDLDREFFVGNWNQSGFLAEGRPTRTLREMLTRLRETYCSHIGYEYMHIPERDKCNWIRERIETIDPVQFTKQQKLHMLDRLSWSDMFETFLANKYTAAKRFGLEGAESLIPGMKTVIDTAADLGVQSVVIGMPHRGRLNVLANVVRKPMSQIFSEFAGKEPIAHEGEYTGSGDVKYHLGTSFNRPTVHGKMVHLSLVANPSHLEAVNTVVLGKTRAKQYYSEDHERGRHLAILLHGDGAFSGQGIVYETLDMSGLPDYTVGGTIHLVVNNQVAFTTDPKDSRSSPYCTDVAKSLNCPIFHVNADDVESVVRVCQLAAEWRQAWKSDVVVDLVCYRKHGHNEIDEPMFTQPLMYKKIKAHKHSAQLYAERLIAEGTFTKEEVQQVRDRIMQHLNAAFEGAKDYKPSKKDWLASHWSGFMSPAQLSRIRNTGVPAELLRSTGLAITALPEDFAFHRQIKKVYETRRAMIESGEGLDWAMAEALAFATLVSEGNHVRLSGQDVERGTFSHRHAVLHDQTNGAKYVPLNHVFPGQKPNSFTVCNSSLSEFGVLGFELGYSMESPNSLVLWEAQFGDFANGAQIIFDQFLSGGEAKWLRQSGLVCLLPHGYDGQGPEHSSARLERFLQMCDENPYEMPHHDEAQWFSGGHLGTQIQRANWQVVNCTTPANYFHVLRRQVHRQFRKPLIVMAPKNLLRHPRCKSPLYEFDDQPDDANIVGVRFKRVIMDDTGLTPKDRGPRPPAEPEIKRVVFCSGKVFYDLHDAREKQGKVGEVAIVRVEQLAPFPFDLVCREIRRYPNAQLLWCQEEPMNMGAYMHVQPRFDTCLREEGKPMMGRMPYAGRPPMAATATGFGEVHGKEQARLIANALDVNYSGAI

>Micromonas pusilla (XP_003062637.1)

MRRHIAARLASAGGDAIAVAARAIPHAAPVTSAPIAFSGAATRRFGALLGGSSPPSSRAPGASSILKRFLPSSSSPLHTSARHRAAAPPTPRATPNAQLQDEFLSGTSAAYVESMEDKFREDPNSVPASWASLLRQMDAGVTGAELSEIPGVAPSSQTIQESMRLLLMVRAFQVNGHAAAKLDPLGLDVRDVPVELDPALYGFTDADLDREFFLGSWRMKGFLSEDNPVQTLRQILTRLRETYCGTVGYEYMHIADRDQCNWLRERIEKAEKHEYSVERKKVLLDRLAWSDMFESFLSNKYTAAKRFGLEGCETLIPGFKEAIDKAAELGVESITIGMPHRGRLNVLANVVRKPLQTIFNEFKGGPKPAGNAAPGGSSYTGSGDVKYHLGTSYDRPTLRGGRMHLSLVANPSHLEAVNTVVIGKARAKQFYENDVERGKHMAVLLHGDGAFSGQGIVYETLDMSQLPEYTIGGTLHVVVNNQVAFTTDPKYSRSSPYCTDVAKGINIPVFHVNGDDVEAVARVMELAIEWRQQWKQDVVVDIVCYRKYGHNEIDEPMFTQPLMYKAIKKHPSAHQQYAEKLMGDGTLTPGDVKLVHDSVLKTLEESFEDSKDYVPKPRDWLASHWAGFKGPDQLSRIRETGVAMEKLKQIGIAATTIPETFTPHRVVKRVYDTRRKMIESGEGLDWAMAEALAFGTLLDEGNHVRLSGQDVERGTFSHRHALIHDQSTGERHVPLRNVYGEAKKKEFFTVSNSSLSEFGVLGFELGYSLENPNALVMWEAQFGDFANSAQIIIDQFISSGEAKWLRQTGLTLLLPHGYDGQGPEHSSCRVERYLQMSDEDPTKIPADMAFETRHQIQEHNWQICNVTTPANYFHLLRRQVHRDFRKPLIVVSPKNLLRHPKCVSPLSDFDDKEETQMEQGVRFKRLIMDKSATSRDKVNTPVENSAKRVVFCTGKVYYELDSEREALGREKDVKIVRIEQLCPFPWDLVGRELRRYPKAEVVWCQEEPMNMGAYSHVAPRFQTLFKDLKRPVDGLRYAGRAPAASTATGYGSVHSEEQVGLIKDALQ

>Saccharomyces cerevisiae (NP_012141)

MLRFVSSQTCRYSSRGLLKTSLLKNASTVKIVGRGLATTGTDNFLSTSNATYIDEMYQAWQKDPSSVHVSWDAYFKNMSNPKIPATKAFQAPPSISNFPQGTEAAPLGTAMTGSVDENVSIHLKVQLLCRAYQVRGHLKAHIDPLGISFGSNKNNPVPPELTLDYYGFSKHDLDKEINLGPGILPRFARDGKSKMSLKEIVDHLEKLYCSSYGVQYTHIPSKQKCDWLRERIEIPEPYQYTVDQKRQILDRLTWATSFESFLSTKFPNDKRFGLEGLESVVPGIKTLVDRSVELGVEDIVLGMAHRGRLNVLSNVVRKPNESIFSEFKGSSARDDIEGSGDVKYHLGMNYQRPTTSGKYVNLSLVANPSHLESQDPVVLGRTRALLHAKNDLKEKTKALGVLLHGDAAFAGQGVVYETMGFLTLPEYSTGGTIHVITNNQIGFTTDPRFARSTPYPSDLAKAIDAPIFHVNANDVEAVTFIFNLAAEWRHKFHTDAIIDVVGWRKHGHNETDQPSFTQPLMYKKIAKQKSVIDVYTEKLISEGTFSKKDIDEHKKWVWNLFEDAFEKAKDYVPSQREWLTAAWEGFKSPKELATEILPHEPTNVPESTLKELGKVLSSWPEGFEVHKNLKRILKNRGKSIETGEGIDWATGEALAFGTLVLDGQNVRVSGEDVERGTFSQRHAVLHDQQSEAIYTPLSTLNNEKADFTIANSSLSEYGVMGFEYGYSLTSPDYLVMWEAQFGDFANTAQVIIDQFIAGGEQKWKQRSGLVLSLPHGYDGQGPEHSSGRLERFLQLANEDPRYFPSEEKLQRQHQDCNFQVVYPTTPANLFHILRRQQHRQFRKPLALFFSKQLLRHPLARSSLSEFTEGGFQWIIEDIEHGKSIGTKEETKRLVLLSGQVYTALHKRRESLGDKTTAFLKIEQLHPFPFAQLRDSLNSYPNLEEIVWCQEEPLNMGSWAYTEPRLHTTLKETDKYKDFKVRYCGRNPSGAVAAGSKSLHLAEEDAFLKDVFQQS

>Aspergillus nidulans

MFRSTAMKATVRGAACSSCRRSMSLASTVRRADSSSKFGLATRRPLALVGKRFYASSAEDSGVAASDSFLSGNTANYIDEMYVAWRKDPSSVHISWQTYFRNMEEGKMPISQAFQPPPTLVPTPTGGVHQEMPGAGLGLSQGTDVTKHLKVQLLVRAYQARGHHKAKIDPLGIRGEAEAFGYSKPKELELDHYGFTERDLDEEFDLGPGILPRFATEGRKKMSLREIIAACEKIYCGSYGVEYIHIPDRKPCDWIRDRFEVPEPYKYSVDDKRRILDRLIWSSSFEAFLATKFPNDKRFGLEGCETLVPGMKALIDRSVDYGIKDIVIGMPHRGRLNVLSNVVRKPNESIFSEFAGSAEPSDEGSGDVKYHLGMNFERPTPSGKRVQLSLVANPSHLEAEDPVVLGKTRAIQHYNNDEKNFDSAMGVLLHGDAAFAAQGIVYETMGFHSLPAYSTGGTIHIVVNNQIGFTTDPRFARSTPYCSDIAKSIDAPVFHVNADDVEAVNYVCQVAADWRAEFKRDVVIDIVCYRKQGHNETDQPSFTQPLMYKRVAEKKLQLDMYVEKLISEGTFTKEDIDEHKKWVWGMLNDSFDRSKDYQPTGKEWLTSAWNGFKTPKELATEVLPHLPTAVEPPILKNVADKISGAPEGFTLHRNLKRILGNRKKTVEEGKNIDWATAEALAFGSLVSEGYHVRVSGQDVERGTFSQRHAVLHDQETEATYTPLQHISKDQGSFVISNSSLSEFGALGFEYGYSLTSPNALVMWEAQFGDFANNAQCIIDQFIASGESKWLQRSGLVLSLPHGYDGQGPEHSSGRMERYLQLCNEEPRVFPSQDKLDRQHQDCNMQVAYMTSPANLFHLLRRQIHRQFRKPLVIFFSKSLLRHPLARSSIEEFTGDSHFQWIIPDPAHGTAIDEPEKIERVILCSGQVYAALTKHREANNIRNTAITRVEQLHPFPWAQLKENLDSYPNARNIVWAQEEPLNAGSWSYVQPRIETLLNETEHHNRRHVMYAGRPPSASVATGLKSVHVKEEQEMLEEAFSVHQERLKGE

>Schizosaccharomyces pombe (NP_595772.1)

MLRFIPSSAKARALRRSAVTAYRLNRLTCLSSLQQNRTFATQPTDDFLTGGAADYVDEMYDAWKKDPNSVHASWQAYFKNVQERGVSPSKAFQAPPLLDYADSYTALDSSLINGNNYADIDVGIYMKVQLLVRAYQSRGHHLAKLDPLGINVNHNRPSELTLEHYGFTESDLNRTIHLGPGILPNFREAGRKTMTLREIVETCEKIYCGSFAVEFTHISSRKRSNWILSHLETPTPFRYSHDQKIMIFDRLSWADSFERFLFTKFPNDKRFGLEGCEAMVPGMKALIDRSVDEGISNIVIGMAHRGRLNLLHNIVRKPAQAIFSEFRGTQDPDDEGSGDVKYHLGMNYQRPTPSGKRVSLSLVANPSHLEAEDPVVLGKVRAIQHYTSDEASHEQSMGILIHGDAAFAAQGVVYETFGLHALPGYSTGGTVHIVINNQIGFTTDPRFARSTPYCTDIAKSMEAPIFHVNGDDVEAVTFICQLAADWRKAFKTDVVVDIVCYRRHGHNETDQPSFTQPRMYKAIAKHPPTFKIYTQQLLQEKTVSKAEVDAQEKRVWDILESSFESSKNYKSDHREWLSNPWVGFASPKDLMTKILPSYPTGVNIDTLKQIGKALYTLPEGFDAHRNLKRILNNRNKSISSGEGIDMPTAEALAFGTLLEEGHHVRVSGQDVERGTFSQRHAVLHDQSSENVYIPLNHLSPNQASFVIRNSSLSEYGVLGFEYGYSLSSPNALVVWEAQFGDFANNAQCIIDQFIAAGETKWLQRTGIVLSLPHGYDGQGPEHSSARMERYLQLCNEDPREFPSEEKLQRQHQDCNIQAIYVTKPSQYFHALRRNIHRQFRKPLVIFFSKSLLRHPAARSTIDEFDEKHGFKLILEEEEHGKSILPPEKIEKLIICSGQVWVALSKAREENKIDNIAITRVEQLHPFGWKQMAANISQYPNLKEIIWCQEEPLNAGAWTYMEPRIYTILKHLGRDLPVRYAGRPPSASVAAGNKQQHLAEQEQFLNDALL

>Candida albicans (XP_710646.1)

MLRAFRSAVPRTQLLKSRLTIPKTSVIGRRYLATDSFLQGSNSNYVDEMYEAWRQDPSSVHASWNAYFKNIENDNIPPSKAFQAPPTIVPTVSGGAAGFYPGQSPISEDVVTHLKVQLLVRAYQVRGHQKAKIDPLGISFGDNTTVPKELTLDYYGFTEQDLAKEITLGPGILPRFAQGGKKSMTLKEIINFCEKTYCSSYGVEYVHIPSKEQCDWLRDRIEVPQPFKYSPDQKRQILDRLIWATSFESFLSSKFPNDKRFGLEGAEAVVPGMKALIDTSVEYGVEDVVIGMPHRGRLNMLSNVVRKPNESIFSEFTGSKEFDEGSGDVKYHLGMNYARPTTSGKHVNLSIVANPSHLEAEDGVVLGKTRAIQQYKQDIGSFKKAMAVLLHGDAAFAGQGVVYETMGFANLPAYSTGGTIHVIVNNQIGFTTDPRFARSTLYPSDIAKAIDAPIFHVNADDVEACTFVFNLAAEWRATYHTDCIIDVVGYRKHGHNETDQPSFTQPLMYQEIAKKNSVIDIYEKQLIDEGTFTAEDIKEHKQWVWNILEDNFKKAKEYKPTSREWLTTPWEDFKSPKELATEVLPHLPTAVDEATLKKIGNAISETPEGFEVHRNLKRILNARKKSVETGEGIDYATGEALAYGSLALEGYHVRVSGQDVERGTFSQRHAVLHDQNSESVWTPLSNLSEDQGAFNISNSSLSEYGVLGFEYGYSLTSPDALVEWEAQFGDFANTAQVVIDQFVAGAESKWKQRSGVVLSLPHGYDGQGPEHSSSRLERYLQLCNEDQRFFPSPEKLERQHQDCNMQVAYPTTPANVFHLLRRQMHRQFRKPLILVFSKSLLRHPLARSNLSEFTGDSHFQWIIEDVLGDKSEVKRVVLLTGQVYAALHKKRASLDDKSTAFIKIEQLHPFPYAQLRDALNEYPNLEDLVWTQEEPLNMGAYNFAAPRVEAVLGETQKYKDLKLRYAGRDPSASVAAGSKAMHVAEEEEFLEETFRQ

>Homo sapiens (NP_001158508.1)

MFHLRTCAAKLRPLTASQTVKTFSQNRPAAARTFQQIRCYSAPVAAEPFLSGTSSNYVEEMYCAWLENPKSVHKSWDIFFRNTNAGAPPGTAYQSPLPLSRGSLAAVAHAQSLVEAQPNVDKLVEDHLAVQSLIRAYQVRGHHIAKLDPLGISCVNFDDAPVTVSSNVGFYGLDESDLDKVFHLPTTTFIGGQESALPLREIIRRLEMAYCQHIGVEFMFINDLEQCQWIRQKFETPGIMQFTNEEKRTLLARLVRSTRFEEFLQRKWSSEKRFGLEGCEVLIPALKTIIDKSSENGVDYVIMGMPHRGRLNVLANVIRKELEQIFCQFDSKLEAADEGSGDVKYHLGMYHRRINRVTDRNITLSLVANPSHLEAADPVVMGKTKAEQFYCGDTEGKKVMSILLHGDAAFAGQGIVYETFHLSDLPSYTTHGTVHVVVNNQIGFTTDPRMARSSPYPTDVARVVNAPIFHVNSDDPEAVMYVCKVAAEWRSTFHKDVVVDLVCYRRNGHNEMDEPMFTQPLMYKQIRKQKPVLQKYAELLVSQGVVNQPEYEEEISKYDKICEEAFARSKDEKILHIKHWLDSPWPGFFTLDGQPRSMSCPSTGLTEDILTHIGNVASSVPVENFTIHGG

>Felis catus (XP_003994212.1)

MSQLRLLPSRLGAQASRLLAPHDIQMFSRRSRSSGPPAPFPSSKRGGSSSYMEEMYFAWLENPQSVHKSWDSFFRKASEEAACDPAQSRFPESRPSVSSRTKTSKLVEDHLAVQSLIRAYQIRGHHVAQLDPLGILDADLDSFVPSDLITTIDKLAFYDLREADLDKEFQLPTTTFIGGSEHTLSLREIIRRLESTYCQHIGLEFMFINDVEQCQWIRQKFETPGVMQFSSEEKRTLLARLVRSMRFEDFLARKWSSEKRFGLEGCEVMIPALKTIIDKSSEMGIENVILGMPHRGRLNVLANVIRKDLEQIFCQFDPKLEAADEGSGDVKYHLGMYHERINRVTNRNITLSLVANPSHLEAVDPVVQGKTKAEQFYRGDAQGKKVMSILVHGDAAFAGQGVVYETFHLSDLPSYTTNGTVHVVVNNQIGFTTDPRMARSSPYPTDVARVVNAPIFHVNADDPEAVIYVCSVAAEWRNTFNKDVVVDLVCYRRRGHNEMDEPMFTQPLMYKQIHRQVPVLKKYADKLIAEGTVTLQEFEEEIAKYDRICEEAYGRSKDKKILHIKHWLDSPWPGFFNVDGEPKSMTCPATGIPEDVLTHIGEVASSVPLKDFKIHTGLSRILRGRADMIKNRTVDWALAEYMAFGSLLKEGIHVRLSGQDVERGTFSHRHHVLHDQEVDRRTCVPMNHLWPDQAPYTVCNSSLSEYGVLGFELGYAMASPNALVLWEAQFGDFHNTAQCIIDQFISTGQAKWVRHNGIVLLLPHGMEGMGPEHSSARPERFLQMSNDDSDAYPVFTKDFEVSQLYDCNWIVVNCSTPANYFHVLRRQVLLPFRKPLIIFTPKSLLRHPEAKSSFDQMVSGASFQRVIPEDGAAAQTPEQVRRLIFCTGKVYYDLVKERSSQGLEEQVAITRLEQISPFPFDLIKQEAEKYPGAELVWCQEEHKNMGYYDYISPRFMTILSRARPIWYVGRDPAAAPATGNRNTHLVSLKKFLDTAFNLQAFEGKTFLSRILKTRGEMVKNRTVDWALAEYMAFGSLLKEGIHIRLSGQDVERGTFSHRHHVLHDQNVDKRTCIPMNHLWPNQAPYTVCNSSLSEYGVLGFELGFAMASPNALVLWEAQFGDFHNTAQCIIDQFICPGQAKWVRQNGIVLLLPHGMEGMGPEHSSARPERFLQMCNDDPDVLPDLKEANFDINQLYDCNWVVVNCSTPGNFFHVLRRQILLPFRKPLIIFTPKSLLRHPEARSSFDEMLPGTHFQRVIPEDGPAAQNPENVKRLLFCTGKVYYDLTRERKARDMVGQVAITRIEQLSPFPFDLLLKEVQKYPNAELAWCQEEHKNQGYYDYVKPRLRTTISRAKPVWYAGRDPAAAPATGNKKTHLTELQRLLDTAFDLDVFKNFS

>Mus musculus (NP_035086.2)

MFHLRTCAAKLRPLTASQTVKTFSQNKPAAIRTFQQIRCYSAPVAAEPFLSGTSSNYVEEMYCAWLENPKSVHKSWDIFFRNTNAGAPPGTAYQSPLSLSRSSLATMAHAQSLVEAQPNVDKLVEDHLAVQSLIRAYQIRGHHVAQLDPLGILDADLDSSVPADIISSTDKLGFYGLHESDLDKVFHLPTTTFIGGQEPALPLREIIRRLEMAYCQHIGVEFMFINDLEQCQWIRQKFETPGIMQFTNEEKRTLLARLVRSTRFEEFLQRKWSSEKRFGLEGCEVLIPALKTIIDMSSANGVDYVIMGMPHRGRLNVLANVIRKELEQIFCQFDSKLEAADEGSGDMKYHLGMYHRRINRVTDRNITLSLVANPSHLEAADPVVMGKTKAEQFYCGDTEGKKVMSILLHGDAAFAGQGIVYETFHLSDLPSYTTHGTVHVVVNNQIGFTTDPRMARSSPYPTDVARVVNAPIFHVNSDDPEAVMYVCKVAAEWRNTFHKDVVVDLVCYRRNGHNEMDEPMFTQPLMYKQIRKQKPVLQKYAELLVSQGVVNQPEYEEEISKYDKICEEAFTRSKDEKILHIKHWLDSPWPGFFTLDGQPRSMTCPSTGLEEDVLFHIGKVASSVPVENFTIHGGLSRILKTRRELVTNRTVDWALAEYMAFGSLLKEGIHVRLSGQDVERGTFSHRHHVLHDQNVDKRTCIPMNHLWPNQAPYTVCNSSLSEYGVLGFELGFAMASPNALVLWEAQFGDFNNMAQCIIDQFICPGQAKWVRQNGIVLLLPHGMEGMGPEHSSARPERFLQMCNDDPDVLPDLQEENFDINQLYDCNWIVVNCSTPGNFFHVLRRQILLPFRKPLIVFTPKSLLRHPEARTSFDEMLPGTHFQRVIPENGPAAQDPHKVKRLLFCTGKVYYDLTRERKARNMEEEVAITRIEQLSPFPFDLLLKEAQKYPNAELAWCQEEHKNQGYYDYVKPRLRTTIDRAKPVWYAGRDPAAAPATGNKKTHLTELQRFLDTAFDLDAFKKFS

>Drosophila melanogaster (NP_730223.1)

MHRAHTAFSLALSPMAHKNFATWLLKSSSSQQMAKVTAAAAVRTYNSAAAEPFANGSTASYVEEMYNAWLRDPTSVHTSWDAYFRSNSYVSPPNLAPVQANTLPLTAFNFGGAVAGAAPDSKTIDDHLAVQAIIRSYQIRGHNIAHLDPLEINTPELPGNSSTKSIYANFSFGEQDMDRQFKLPSTTFIGGDEASLPLKEILNRLENVYCNKIGVEFMFINSLEQCNWIRKRFETPGVLNFSPEEKRLILARLTRATGFEAFLAKKYSSEKRFGLEGCEIMIPALKEIIDVSTELGVESVIMGMPHRGRLNTLANVCRKPLNQIFTQFAGLEAADDGSGDVKYHLGTYIERLNRVTNKNIRLAVVANPSHLEAVDPVVQGKTRAEQFYRGDQEGKKVMSILIHGDAAFCGQGVVYETMHLSDLPDYTTHGTIHVVANNQIGFTTDPRFSRSSPYCTDVARVVNAPIFHVNADDPEAVMHVCKVAAEWRATFHKDCVIDLVGYRRNGHNEIDEPMFTQPLMYQKIRKHKNCLDLYADKLIAEGTVTAEEVKSVAAKYENICEEAFALAKTETHVKYKDWLDSPWSGFFEGKDPLKVAPTGVKEETLIHIGNRFSSPPPNAAEFVIHKGLLRVLAARKAMVDEKVADWALGEAMAFGSLLKEGIHVRLSGQDVERGTFSHRHHVLHHQLVDKATYNSLQHMYPDQAPYSVSNSSLSEYAVLGFEHGYSMTNPNALVLWEAQFGDFSNTAQSIIDQFISSGQSKWVRQSGLVMLLPHGMEGMGPEHSSCRVERFLQMSSDDPDYFPPESDEFGVRQLHDINWIVANCSTPANYYHILRRQIALPFRKPLILCTPKSLLRHPEAKSPFSEMSEGSEFQRIIPDNGPAGQNPSNVKKVVFCSGRVYYDLTKTRREKQLEGEIAIVRVEQISPFPFDLVKEQANLYKNAELVWAQEEHKNQGSWTYVQPRFLTALNHSRDVSYVGRACGASTATGSKAQHIRELNALLNDAIST

>Drosophila melanogaster (NP_730223.1)

MHRAHTAFSLALSPMAHKNFATWLLKSSSSQQMAKVTAAAAVRTYNSAAAEPFANGSTASYVEEMYNAWLRDPTSVHTSWDAYFRSNSYVSPPNLAPVQANTLPLTAFNFGGAVAGAAPDSKTIDDHLAVQAIIRSYQIRGHNIAHLDPLEINTPELPGNSSTKSIYANFSFGEQDMDRQFKLPSTTFIGGDEASLPLKEILNRLENVYCNKIGVEFMFINSLEQCNWIRKRFETPGVLNFSPEEKRLILARLTRATGFEAFLAKKYSSEKRFGLEGCEIMIPALKEIIDVSTELGVESVIMGMPHRGRLNTLANVCRKPLNQIFTQFAGLEAADDGSGDVKYHLGTYIERLNRVTNKNIRLAVVANPSHLEAVDPVVQGKTRAEQFYRGDQEGKKVMSILIHGDAAFCGQGVVYETMHLSDLPDYTTHGTIHVVANNQIGFTTDPRFSRSSPYCTDVARVVNAPIFHVNADDPEAVMHVCKVAAEWRATFHKDCVIDLVGYRRNGHNEIDEPMFTQPLMYQKIRKHKNCLDLYADKLIAEGTVTAEEVKSVAAKYENICEEAFALAKTETHVKYKDWLDSPWSGFFEGKDPLKVAPTGVKEETLIHIGNRFSSPPPNAAEFVIHKGLLRVLAARKAMVDEKVADWALGEAMAFGSLLKEGIHVRLSGQDVERGTFSHRHHVLHHQLVDKATYNSLQHMYPDQAPYSVSNSSLSEYAVLGFEHGYSMTNPNALVLWEAQFGDFSNTAQSIIDQFISSGQSKWVRQSGLVMLLPHGMEGMGPEHSSCRVERFLQMSSDDPDYFPPESDEFGVRQLHDINWIVANCSTPANYYHILRRQIALPFRKPLILCTPKSLLRHPEAKSPFSEMSEGSEFQRIIPDNGPAGQNPSNVKKVVFCSGRVYYDLTKTRREKQLEGEIAIVRVEQISPFPFDLVKEQANLYKNAELVWAQEEHKNQGSWTYVQPRFLTALNHSRDVSYVGRACGASTATGSKAQHIRELNALLNDAIST

>Escherichia coli (gi|1786945)

MQNSALKAWLDSSYLSGANQSWIEQLYEDFLTDPDSVDANWRSTFQQLPGTGVKPDQFHSQTREYFRRLAKDASRYSSTISDPDTNVKQVKVLQLINAYRFRGHQHANLDPLGLWQQDKVADLDPSFHDLTEADFQETFNVGSFASGKETMKLGELLEALKQTYCGPIGAEYMHITSTEEKRWIQQRIESGRATFNSEEKKRFLSELTAAEGLERYLGAKFPGAKRFSLEGGDALIPMLKEMIRHAGNSGTREVVLGMAHRGRLNVLVNVLGKKPQDLFDEFAGKHKEHLGTGDVKYHMGFSSDFQTDGGLVHLALAFNPSHLEIVSPVVIGSVRARLDRLDEPSSNKVLPITIHGDAAVTGQGVVQETLNMSKARGYEVGGTVRIVINNQVGFTTSNPLDARSTPYCTDIGKMVQAPIFHVNADDPEAVAFVTRLALDFRNTFKRDVFIDLVCYRRHGHNEADEPSATQPLMYQKIKKHPTPRKIYADKLEQEKVATLEDATEMVNLYRDALDAGDCVVAEWRPMNMHSFTWSPYLNHEWDEEYPNKVEMKRLQELAKRISTVPEAVEMQSRVAKIYGDRQAMAAGEKLFDWGGAENLAYATLVDEGIPVRLSGEDSGRGTFFHRHAVIHNQSNGSTYTPLQHIHNGQGAFRVWDSVLSEEAVLAFEYGYATAEPRTLTIWEAQFGDFANGAQVVIDQFISSGEQKWGRMCGLVMLLPHGYEGQGPEHSSARLERYLQLCAEQNMQVCVPSTPAQVYHMLRRQALRGMRRPLVVMSPKSLLRHPLAVSSLEELANGTFLPAIGEIDELDPKGVKRVVMCSGKVYYDLLEQRRKNNQHDVAIVRIEQLYPFPHKAMQEVLQQFAHVKDFVWCQEEPLNQGAWYCSQHHFREVIPFGASLRYAGRPASASPAVGYMSVHQKQQQDLVNDALNVE

>Starkeya novella (YP_003695193.1)

MSRADLNEAFLNTSFLYGANAAWIEDLYARYEADPSSVDAEWQAFFAGLKDTPADVEKSARGASWKKEGWPIHANGELVSALDGNWIEVEKAVGKKIETKAAEKAQKAGVELTSADVQQATRDSVKALMMIRAYRMRGHLHAKLDPLGLEPERSAPELDPASYGFREADLDRPIFIDHVLGLEFATVRQMVAILQRTYCQTLGVEFMHISSPEEKAWIQERIEGPDKEISFTREGKRAILNKLVEAEGFEKFLDVRYTGTKRFGLDGGESLIPALEQIIKRGGNLGVKEIVLGMAHRGRLNVLTQVMGKPHRALFHEFKGGSWAPDEVEGSGDVKYHLGASSDREFDGNQVHLSLTANPSHLEIVDPVVLGKARAKQDLLGDTERTQVMPLLLHGDAAFAGQGVVAECLGLSGLKGHRTGGSIHFIINNQIGFTTYPRFSRSSPYPSDVAKTIEAPIFHVNGDDPEAVTFAAKIATEFRQRFKKPVVVDMFCYRRFGHNEGDEPAFTQPLMYKLIKQHPTTLEIYSRKLEAEGVLEAGEIDRMRADWRSRLDTEYDAGQAYKPNKADWLDGRWAGLKAAASEDDPRRGVTGVDLDVLKEIGQKITTVPEGFHAHRTIQRFLDSRRKAILEDGAGIDWSTAEALAFSTLLLDGHPVRLSGQDSERGTFSQRHSVLIDQENEDRYTPFNHLREGQSRYEVINSMLSEEAVLGFEYGYSLSEPNALTMWEAQFGDFANGAQVIFDQFLSSGERKWLRMSGLVCLLPHGYEGQGPEHSSARLERYLQMCAEDNMQVANLTTPANYFHALRRQLKRDFRKPLILMTPKSLLRHKRAVSKLAEMGAGTSFHRVLWDDADGASGHRLPEAIELQTDDKIRRVVLCSGKVYYDLYEERERRGIDDIYLLRVEQLFPFPLKTLVQELSRFKQAEIVWCQEEPKNQGAWAFVQPYLEWVLEQVGSASARPRYTGRPASAATATGLMSKHLAQLKAFLSDALG

>Chelativorans (YP_675934.1)

MARQNQANEDFSITSFLYGGNAAYIEDLYASYESDPSSVSDDWRTFFSQLKDNAADVKKSARGASWKQKGWPLTANGELVSALDGDWGALEGNIDLKLKKKAAEGGISLSEAELQRATRDSVRAIMMIRAYRMRGHLHADLDPLGLAKPMEDYNELSPEAYGFTEADFDRPIFIDNVLGLETATIREMLAILRRTYCSTLGVEFMHISNPEEKAWIQERIEGPDKGVAFTANGKKAILQKLIESEGFEQFLDVKYKGTKRFGLDGSESLIPALEQIIKRGGQLGLKEIVLGMAHRGRLNVLSQVMGKPHRAIFHEFKGGSFTPDEVEGSGDVKYHLGASSDREFDGNKVHLSLTANPSHLEIVNPVVMGKARAKQDQLFGRTREEIIPLAERSRVMPLLIHGDAAFAGQGVVAECLGLSGLRGHRVAGTVHVIINNQIGFTTNPRFSRSSPYPSDVAKMIEAPIFHVNGDDPEAVVYAAKVATEFRMAFQKPVVIDMFCYRRFGHNEGDEPAFTQPIMYRTIRSHPTTVEVYSRKLIDEGLVTKDDIDQMRAEWRATLEAEFDAGQSYKPNKADWLDGAWSGLKKADDGDEQRRGKTAVPVKTLKEIGKKLTEVPADFEVHRTVRRFLENRKQMIETGEGIDWATAEALAFGSILIEGNPVRLSGQDSERGTFSQRHSVLYDQRDENRYIPLNHLGPQQAYYDVINSMLSEEAVLGFEYGYSLAEPRALTLWEAQFGDFANGAQVVFDQFISSGERKWLRMSGLVCLLPHGYEGQGPEHSSARLERFLQLCAEDNMQVANCTTPANYFHILRRQLKRDFRKPLILMTPKSLLRHKRAVSTLAEMSGESTFHRLLWDDAQSLQNQPIKLVKDSKIRRVVMCSGKVYYDLYEEREKRGINDVYLLRVEQLYPFPAKALITELSRFRNAEMVWCQEEPKNMGAWSFIDPYLEWVLQHIEAKNKRVRYAGRPASASPATGLMSKHLEQLGQLLEDALG

>Pelagibacterium halotolerans (WP_014132324.1)

MARQDQNEAFLLTSFLYGGNADYIDALYARYKSDAKSVDPSWAEFFDNLADSADCVTKNADGPSWQRADWPRASNGEMISALDGNWGEVAVKAQKAVTEKARAGGEVVSTEAVMQATRDSIHAIMMIRAYRMRGHLHANLDPLGLENREEAPELDPAAYGFSEADYTREIFIDNYLGLEFATVPQMLEILRRTYCGTLGIEFMHISDPEAKAWIQERIEGPDKEITFTPQGKRAILNKLAEAEGFEKFLDVKYTGTKRFGLDGGESLIPALEQIVKRGGALGVKDIVLGMPHRGRLNVLTQLMAKPHRALFHEFKGGAFYPDDVEGSGDVKYHLGASSDREFDGNKVHLSLTANPSHLEIVNPVVLGKARAKQDQLSAIEGRFVADTRDTDRTAVLPLLLHGDAAFAGQGVVAECFALSGLKGHRTGGSIHFVVNNQIGFTTSPHFSRSSPYPTDVAKMIEAPVFHCNGDDPEAVVYAAKIAVEFRQKFGRPVVIDMFCYRRFGHNEGDEPSFTQPLMYKAIRGHKTTLEIYGNKLVEEGVLTAEEFDALKAEWRARLDSEFEAGQDYRPNKADWLDGAWKNIKLAEVDGPRRGVTGIEMERLKALGEKLTTVPGDFHAHRTVQRFLDNRKKMIAEGEGIDWATAEALAFASLLEDGHPVRLSGQDVERGTFSQRHSVLYDQENESTFTPLNNLVDGQARYEVINSLLSEEAVLGFEYGYSLAEPNALTVWEAQFGDFVNGAQVVIDQFISSGERKWLRMSGLVMLLPHGYEGQGPEHSSARPERFLQLCAEDNMQVLNCTTPANYFHALRRQLKRDFRKPLIIMTPKSLLRHKRAVSGLGEMGSDTFFHRLLWDDAEAPGLPKTEINLVGDDKIRRVVICTGKVYYDLLEDREKRGINDVYLMRLEQLYPFPAKALIDELSRFQNAEVVWCQEEPKNMGAWSFVQPYIEWVLEQMGRPGGRPRYVGRPASASTATGLMRTHVAQLQAFLDEAFE

>Labrenzia alexandrii (WP_008191149.1)

MARQEANNVFALTSLLYGANAAYIEDLYASYKTDPNSVDPEWRDFFAAFQDEKDAVLKEARGAPWKRKDWPLEASGDLVNAFDGNWAPIEQKLETKLKQKADTTGAPMSDAEVHQATRDSVRALMMIRAYRMRGHLHADLDPLQLATPGDHEELHPSSYGFTEADWDRSIFIDHVLGLEYATIREMLDILKRTYCSTLGVEFMHISDPAAKSWLQERIEGPDKQVAFTSEGKKAILNKLVEAEGFEKFLDVKYTGTKRFGLDGGEALIPALEQIIKRGGQMGLKDIVLGMAHRGRLNVLTQVMRKPHRAVFHEFKGGSYAPDEVEGSGDVKYHLGASSDRDFDGNNVHLSLTANPSHLEIVNPVVLGKARAKQDQLASKDDGTFIETTEVERSSVLPLLLHGDAAFAGQGVVAECFGLSALRGHRTGGSIHVIINNQIGFTTNPRFSRSSPYPSDMAKVIESPIFHVNADDPEAVVFAAKIAIEYRQTFGRPVVIDMICYRRFGHNEGDEPAFTQPIMYRKIRKHQTTLQLYSDRLIKEGVMSQADVDRMKAEWRSHLDTEFDSGQAFKPNKADWLDGKWAGLKRADDEEDPRRGETGVAIDELKDIGRKLTKIPNDFNAHRTIARFMNNRERMIETGEGIDWATAEAMAFATLLKEGHPIRLSGQDCERGTFSQRHSVLYDQENENRYIPLNHVGEGQQRYEVINSMLSEEAVLGFEYGYSLAEPRALTLWEAQFGDFANGAQVLFDQFISSGERKWLRMSGLVCLLPHGYEGQGPEHSSARLERFLQLCAEDNMQVANCSTPANYFHILRRQLCRDIRKPLILMTPKSLLRHKKAVSKIEELGPDSTFHRLLWDDAESNPAAETKLVPDDKIKRVVMCSGKVYYDLYEEREKRGIDDIYLFRVEQLYPFPKKALMMELARFPQAEMVWCQEEPKNMGSWFSVEPYIEWVLEQIDAKHKRPRYAGRAAMASTATGLMSAHLAQLQAFLEEALGN

________________________________________________________________________________

E1: Pyruvate dehydrogenase

>Arabidopsis thaliana (NM_203027.2)

MWTLNVQVMDFPGGKVAFTPEIQFISESDKERVPCYRVLDDNGQLITNSQFVQVSEEVAVKIYSDMVTLQIMDNIFYEAQRQGRLSFYATAIGEEAINIASAAALTPQDVIFPQYREPGVLLWRGFTLQEFANQCFGNKSDYGKGRQMPVHYGSNKLNYFTVSATIATQLPNAVGAAYSLKMDKKDACAVTYFGDGGTSEGDFHAALNIAAVMEAPVLFICRNNGWAISTPTSDQFRSDGVVVKGRAYGIRSIRVDGNDALAMYSAVHTAREMAIREQRPILIEALTYRVGHHSTSDDSTRYRSAGEIEWWNKARNPLSRFRTWIESNGWWSDKTESDLRSRIKKEMLEALRVAEKTEKPNLQNMFSDVYDVPPSNLREQELLVRQTINSHPQDYPSDVPL

>Glycine max (XP_003526663.1)

MASWLLGRSSRILIHQHHHLHKKYLPFLLGPFLNHNNSSCSSFPSTEPFRCRDAFPGKNSSPTLFRFSRHESTKAEAQLELEQDVATEDEPNQVIDFPGGKVGFTSEMRFISESPEKRVPCYRVLDDNGEIVKYSNYVQVSKEMGVKMYSDMVTLQTMDNIFYEVQRQGRISFYLTQMGEEAVNIASAAALAPDDIILPQYREPGVLLWRGFTLQQFVHQCFGNTHDFGKGRQMPIHYGSNQHNYFTVSSPIATQLPQAVGAAYSLKMDGKSACAVTFCGDGATSEGDFHAAMNFAAVMEAPVVFICRNNGWAISTPVEDQFRSDGIVVKGKAYGIWSIRVDGNDALAVYSAVHTAREIAIKEKRPVLIEALTYRVGHHSTSDDSTKYRGTDEIEYWKMARNPVNRFKRWVERNGWWSDKDELELRSSVRKQLMHAIQVAEKAQKPPLQDLFNDVYDQIPSNLQEQERLIRKTIEKHPKDYPSDVPL

>Vitis vinifera (XP_002265166.1)

MALPLGKSKRILQCLKSKICVVGLGKNSFWSGWFHHGCPSSMAPLAPTCRNPDAVLVNAATQFATRRFESTKAEKHVDSLCDAEGNQLLDFPGGKVAFTSEMRFIPESPMERAHCYRVLDDNGQPNMSNFVQVSKEVAVKMYATMLTLQVMDTIFYEAQRQGRISFYVTSIGEEAINVASAAALSIDDVIFPQYREPGVLLWRGFTLQEFANQCFGNKADYGKGRQMPIHYGSNKHNYVTVSSTVATQIPQAVGAAYSLKMDGKDACTVTYFGDGGSSTGDFHAALNFAAVMEAPVIFICRNNGWAISTPVSDQFRSDGVVVRGRGYGVRSIRVDGNDALAMYTAVHAARKMAITEHRPILIEALTYRAGHHSTSDDSSKYRPVSEIELWRMARDPVSRLRRWIESNDWWSGEAESELRSNVRKEVLDAIQVAERVEKPPIAEIFTDVYDAPPSNLCEQEKLLRETIRRYPQDYPPDVPAL

>Ricinus communis (XP_002516305.1)

MCLSASLSSPIHKHKTPYTAPLAQDFKNPDANCINRVSRRFESTKADSYSSFSNNKILDFPVGKIAFTPEMRFISESPEERIPCYRVLDDNGQLIEDGDNVGVSRHIARKMYCDMVTLQTMDTIFYEAQRQGRISFYVTAIGEEAINIASAAALTIDDLVVPQYREPGVLLWRGFSLQEFANQCFSNKGDNCKGRQMPAHYGSKKHNYFTVASTIASQLPHAVGAAYSLKMDGRDACVVAYFGDGGSSEGDFHAALNFAAVMEAPVIFICRNNGWAISTPVSDQFRSDGIVVRGQAYGVRSIRVDGNDALAMYSAVYAAREMAINEHRPILIEALTYRVGHHSTSDDSTKYRSVDEVEQWRLARDPVMRFLKWIESNGWWSNEAESELRSSIRKQLLNVIQVAERVEKPPVADMFSDIYDVPPPHLCDQEKWLREAIKRHPQDYPPDVPL

>Medicago truncatula (XP_003602628.1)

MSMNTMIRKSTTILTYFKSNYNNFFSTSTRRKPNLTNHHPRRFNSTTSPNPIHDQVIDFPGGNLKFIPQMTFLSESQQHRVPCYRVLDDNGEPIFGTDFVQVSEDFAVKMYNNMVALQTMDTIFYEAQRQGRISFYVTTNGEEAINIASAAALSMNDVIFPQYREQGVLLWRGFTLQEFANQCFSNKFDNGKGRQMPAHYGSNKHNYMNVASTVATQIPHAVGAAYSLKMDKKDACAVTYFGDGGSSEGDFHAGLNFAAVMEAPVIFICRNNGWAISTPTSDQFRSDGIVVKGQAYGVRSIRVDGNDALAIYSAVQAARQMAVSEERPILIEALTYRVGHHSTSDDSTKYRPANEIEWWRLSRDPVARFRKWIERNGWWNDMAESELRNSLRQQLLQTIQVAESVEKPPLADMFSDVYDVPPSNLHEQEKRLKETVKKHPHVYPTNISI

>Solanum lycopersicum (XP_004239633.1)

MALLLSKSRKLICLKPKIGFLSSVYHNHTLISPNLRTPFRILGLNQSHFAYFRFGSTKAQTQLNPMHSIHNDSQVLDFPGGEVKFTSHLNFIPETREERVHCYRVLDDDGYPITNDFAQIEKEVALKIYTDMVTLQTMDTIFYEAQRQGRISFYLTTVGEEAINIASAAALKTDDFIFPQYRESGVLLWRGFTIQEFANQLFGNKNDYGKGRQMPIHYGSNKHNYITVASTVATQLPHAVGAAYALKMDAKNACTIVYFGDGGSSTGDFHAALNFAAVLDAPVIFFCRNNGFAISTPVSDQFRSDGVVTKGQGYGIRSIRVDGNDALAVFTAVHEARKMAVNECKPILVEALTYRAGHHSTSDDSTKYRPAKEIEWWRRERDPVSRFKKWIEREGWLNPQVESDLRSNIRKQVLQAIQVAEKQEKPPIKDVFTDVYDVSPANLQEQEVSIRETVRKHPQDYPTDVPV

>Prunus persica (EMJ13293.1)

MHQWMRKSGTIVNHFKSLLLVTNNLSSWRSYSFVKRSQFAASFAHNLPVADRDFINSSTHFCPRRFESVKSEKIANNVDDNQALELPEGKVKFTPKLRFISETADERECCYRVLDENGNRISSSNYVEVSKEVAIKMYTDMVTLQTMDTIFYEAQRQGRISFYVTAAGEEAINIASAAALKIDDIVFPQYREAGVLLWRGFTIQEFANQCFSNKADYGKGRQMPIHYGSNKHNYVTVASTVATQLPQAVGAAYSLKMEKQDACAIAYVGDGGTSEGDFHAALNFAAVTEAPVIFFCRNNGWAISTPTSDQFRSDGVVVKGRAYGVRSIRVDGNDALAVYSAVHAARDMAIREQRPILIEALTYRVGHHSTSDDSTKYRPVKEIEWWKMEQDPVTRFRNWMENNSWWSDKAESEARNSARKQILHAIQEAEKVDKPPVADIFTDVYDSPPSHLCEQEKLLREAIKRHPQDYPSDVPL

>Populus trichocarpa (XP_002306669.1)

MALDFPGGKVTYTPEMRFLSESNGKRVPCYRVLDDNGEIIIGSDYEQLSEEVAVKIYSNMVSLQMMDTIFYEAQRQGRISFYLTSTGEEAINIASAAALSADDIILPQYREPGILLWRGFTIQEFANQCFGNKDDYGKGRQMPIHYGSKKHNFVTISSPIATQLPQAVGIAYSLKMDKKDSCVVTYTGDGGTSEGDFHAALNFAAVTEAPVVFICRNNGWAISTHISEQFRSDGIVVRGPAYGIRSIRVDGNDALAVYSAIHAAREMAISKQRPVLVEALSYRVGHHSTSDDSTKYRPVDEIDYWKKERNPVNRFRKWVERNVWWSEEKESELRSSIKKQLLQVIQVAEKTEKPPLKYLFSDVYDIPPPNLCEQEKQLRETIYAHPQDYPSDVPL

>Oryza sativa (ABA95968.2)

MAPWRAAAMASSQLARRAARRLLSSQHRRHCAAPACPWLLGSAPPVLAPSPVAAAAAGDRRGFCSVRRFTGESNAAAAVEEAENGLVAGGDQQAIDFPGGKVSFVAEMNFLPESQRDRINCYRVLDDDGRTISGSRFQEVSKELALKMYNEMATLQVMDTIFFEAQRQGRISFYLTSHGEEAINIASAAALTIDDIVLPQYREPGVLLWRGFTLQEFANQCFGNKLDYGKGRQMPIHYGSNRLNYFTVSSPIATQLPHAVGAAYSLKMDKKDACAITYFGDGGTSEGDFHAALNFAAVMEAPVIFFCRNNGWAISTPTSEQFRSDGAVIRGQAYGMRSIRVDGNDALAVYSAVHTAREMAIKEGRPILVEALTYRVGHHSTSDDSTKYRPVDEIEHWRTARDPISRYRKWVQGNGWWCDEDESELRNNVRQELLKAIQVAERMPKPPLAELFTDVYDEVPSNLREQERLLRDTIKKHPADYPADVHI

>Zea mays (NP_001130417.1)

MASLWLARASSQLARRAAARRPPPRPARHGSGQPTSSWFLGSVPQAALGSPALDTSRRGFCSVRRFAGESSAAAAAAAVVDEEPESGFAAGDQQAVDFPGGKVSFVAEMNFLPESTRERINCYRVLDDDGRTISGSRFQEVSRELALKMYSEMVTLQIMDTIFYEAQRQGRISFYLTSNGEEAINIASAAALSMDDIVLPQYREPGVLLWRGFTLQEFANQCFGNKLDYGKGRQMPIHYGSNRLNYFTVSSPIATQLPHAVGAAYSLKMDKKDACAITYFGDGGTSEGDFHAALNFAAVMEAPVIFFCRNNGWAISTPTTEQFRSDGVVIRGQAYGIRGIRVDGNDALAVYSAIHAAREMAVTEGRPILVEALTYRVGHHSTSDDSTKYRPVDEIEHWRTARDPISRYRKWVQGNDWWSDAEESELRSRVRKELLQAIQVAERMPKPPVTELFTDVYDKIPSNLHEQEQLLRDTIMRHPADYPTDVPV

>Brachypodium distachyon (XP_003578820.1)

MAAWMIARRAARRLAGELSRGGPRTWSQLASPTGPAPAWNRRGFCSVRRFAGDSAAAAADAEEEDEPDNGRSAGRDQVDFPGGKISFVGEMNFLPESQRERINCYRVLDDDGGTIYGSRFREVSKELALKMYNDMVTLQIMDTIFYEAQRQGRISFYLTSNGEEAINIASAAALTADDIVLPQYREPGVLLWRGFTLQEFANQCFGNKLDYGKGRQMPIHYGSNRLNYFTVSSPIATQLPQAVGAAYSLKMDKKDACAITYFGDGGTSEGDFHAGLNFAAVTEAPVIFFCRNNGWAISTPTTEQFRSDGIVTRGQAYGIRSTRIDGNDALAVYSAVRTAREMAITEGRPILIEAMTYRVGHHSTSDDSTKYRPADEIEHWRTARDPISRYRKWVQGNGWWCDAEESELRNKVRQELLQAIQVAERMPKPRLTELFSDVYDQIPSNLREQERSLLDTINKHPADYPADVPV

>Chlorella variabilis (EFN56666.1)

MDFPGGSVPFTPTLTFVGGTFSPRAPLPCYRTIDAAGHAVGEADVPHVLGQDTAVRMYQTMVKLQTVDTIFYEAQRQGRFSFYMTSSGEEATAIGSAAALTNDDVVFSQYREQGVILYRGFSVQDMAHQCFGNMHEQGRGRQMPIHYGSKALNFHTISSTLATQLPHAVGAAYALRLDKKQACSVAYFGEGAASEGDFHAAMNFAATLGAPVVFICRNNGWAISTPATDQYRGDGIAGRGPGYGMAALRVDGGDARAVFNATAEARRIAIEEHVPVLVEAMSYRSGHHSTSDDSSRYRAAEEMRQWRARDPVTRFQRWLIDQGWWDDAQDTAARQEARREVIQALETAQKAPKPPLSAMFSDVYAEMPWHLARQQAEVFAHVTAHPDACPADIPVK

>Coccomyxa subellipsoidea (EIE20627.1)

MDFPGGRVPFTDRLSFVGGAVSPSQPTSCYRTLDSTGACIEDAEVPHDIDEELALKMYETMVKLQTMDVIFYEAQRQGRFSFFMTSNGEEATIIGSAAALSPKDHIFSQYREHGALLYRGFSFLDMANQCFGNVLGHGKGRQMPIHYGSKAHNFQTVSAPLATQLPHAVGAAYALKLQGQSAVTAVYFGEGAASEGDFHAALNFAATLSAPVLFICRNNGWAISTPANEQYKGDGIVGRGPSYGIPSVRVDGGDARAVFSATAEARRIALEKTCPVLIEAMSYRSGHHSTSDDSSRYRTAEEMSAWRARDPATRFHNFIVSRGWWDDKRERQLRVATRKQVVDALTEAQKGAKPPLEDMFTDVYKEMPWHLQEQAAEARAHAQRHPESLHGIPLE

>Chlamydomonas reinhardtii (XP_001694054.1)

MLEVPGGRVPYTPELRFLGGPDAPIPTMPCYRTIDSTGQDVPGAHIPHPLSQVREHAGAGGKDMEQGRYRIGRFSFYLTCQGEEATNIGSAAGLGGQDMVFAQYREQGVLLWRGYTLDQFANQLLGNALEPGKGRQMPIHYGSPELAYQTISSPLATQMPHAVGTAYGYKMDRLPRVAVTYFGDGASSEGDAHAAFNFAAVLGAPCLFVCRNNGYAISTPAHEQYKGDGIAGRGPMYGIPSIRVDGGDVRAVYNAVGQGQQQAAEPGPVLIECMSYRSGHHSTSDDSTRYRTSEEMGAWRARDPVARFRSWLVRQGWWDEAREAELRRSTRQEVLAALDRAAQVPKPPLSDMFTDVY

>Saccharomyces cerevisiae (NP_011105.4)

MLAASFKRQPSQLVRGLGAVLRTPTRIGHVRTMATLKTTDKKAPEDIEGSDTVQIELPESSFESYMLEPPDLSYETSKATLLQMYKDMVIIRRMEMACDALYKAKKIRGFCHLSVGQEAIAVGIENAITKLDSIITSYRCHGFTFMRGASVKAVLAELMGRRAGVSYGKGGSMHLYAPGFYGGNGIVGAQVPLGAGLAFAHQYKNEDACSFTLYGDGASNQGQVFESFNMAKLWNLPVVFCCENNKYGMGTAASRSSAMTEYFKRGQYIPGLKVNGMDILAVYQASKFAKDWCLSGKGPLVLEYETYRYGGHSMSDPGTTYRTRDEIQHMRSKNDPIAGLKMHLIDLGIATEAEVKAYDKSARKYVDEQVELADAAPPPEAKLSILFEDVYVKGTETPTLRGRIPEDTWDFKKQGFASRD

>Schizosaccharomyces pombe (NP_594892.1)

MFRTCTKIGTVPKVLVNQKGLIDGLRRVTTDATTSRANPAHVPEEHDKPFPVKLDDSVFEGYKIDVPSTEIEVTKGELLGLYEKMVTIRRLELACDALYKAKKIRGFCHLSIGQEAVAAGIEGAITLDDSIITSYRCHGFAYTRGLSIRSIIGELMGRQCGASKGKGGSMHIFAKNFYGGNGIVGAQIPLGAGIGFAQKYLEKPTTTFALYGDGASNQGQAFEAFNMAKLWGLPVIFACENNKYGMGTSAERSSAMTEFYKRGQYIPGLLVNGMDVLAVLQASKFAKKYTVENSQPLLMEFVTYRYGGHSMSDPGTTYRSREEVQKVRAARDPIEGLKKHIMEWGVANANELKNIEKRIRGMVDEEVRIAEESPFPDPIEESLFSDVYVAGTEPAYARGRNSLEYHQYK

>Candida albicans (XP_715476.1)

MYRATATSRQLVGTTANILVAKRSMAKAASDLVTIELPASSYEGYNLEVPALSFETEKETLLKMYKDMIIIRRMEMAADALYKSKKIRGFCHLSVGQEAIAVGIENAITPTDTVITSYRCHGFAFMRGASVKSVLAELMGRRSGIANGKGGSMHMFTNGFYGGNGIVGAQVPLGAGLAFSHKYKNDKAVTFDLYGDGASNQGQVFEAYNMAKLWNLPVIFACENNKYGMGTSAARSSAMTEYYKRGQYIPGLKINGMDVLATYQASKFAKDWASQGNGPLVLEYETYRYGGHSMSDPGTTYRTREEVQHMRSRNDPIAGLKAVLLEKEIASEDEIKSYDKAARKYVDEQVAAAEADAPPEAKMDILFEDVYVPGSEIPVLRGRISDDSWDFKNKTFLNKVY

>Aspergillus nidulans (XP_662766.1)

MLFRTAWARQAAPLRRQAFAPLARRSVTTDAASSHAENIPEDENKPFTVRLSDESFETYEIDPPPYTLEVTKKELKQMYYDMVAMRRMEMAADRLYKEKKIRGFCHLSTGQEAVAVGIEHALTREDKIITAYRCHGYAMMRGGTIRSIIGELLGRREGIAYGKGGSMHMFAPNFYGGNGIVGAQVPVGAGLAFAQQYNEEKSTSVVLYGDGASNQGQVFEAFNMAKLWNLPVLFGCENNKYGMGTSAARSSALTDYYKRGQYIPGIKVNGMDVLATKAAVKYGKDYAISGNGPLVYEYVTYRYGGHSMSDPGTTYRSREEIQRMRSTQDPIQGLKQKILDWGVMSEEDLKGLDKSARAHVDEEVAIAEKMPLPENNSRILFEDIYVRGSEPRWMRGRTVDETFYY

>Homo sapiens (BAG36106.1)

MAVAIAAARVWRLNRGLSQAALLLLRQPGARGLARSHPPRQQQQFSSLDDKPQFPGASAEFIDKLEFIQPNVISGIPIYRVMDRQGQIINPSEDPHLPKEKVLKLYKSMTLLNTMDRILYESQRQGRISFYMTNYGEEGTHVGSAAALDNTDLVFGQYREAGVLMYRDYPLELFMAQCYGNISDLGKGRQMPVHYGCKERHFVTISSPLATQIPQAVGAAYAAKRANANRVVICYFGEGAASEGDAHAGFNFAATLECPIIFFCRNNGYAISTPTSEQYRGDGIAARGPGYGIMSIRVDGNDVFAVYNATKEARRRAVAENQPFLIEAMTYRIGHHSTSDDSSAYRSVDEVNYWDKQDHPISRLRHYLLSQGWWDEKQEKAWRKQSRRKVMEAFEQAERKPKPNPNLLFSDVYQEMPAQLRKQQESLARHLQTYGEHYPLDHFDK

>Mus musculus (P50136.1)

MSAAKIWRPSRGLRQAALLLLGRSGVRGLARSHPSRQQQQQFPSLDDKPQFPGASAEFVDKLEFIQPNVISGIPIYRVMDRQGQIINPSEDPHLPQEEVLKFYRSMTLLNTMDRILYESQREGRISFYMTNYGEEGTHVGSAAALERTDLVFGQYREAGVLMYRDYPLELFMSQCYGNVNDPGKGRQMPVHYGCKERHFVTISSPLATQIPQAVGAAYAAKRANANRIVICYFGEGAASEGDAHAGFNFAATLECPIIFFCRNNGYAISTPTSEQYRGDGIAARGPGYGIKSIRVDGNDVFAVYNATKEARRRAVAENQPFLIEAMTYRIGHHSTSDDSSAYRSVDEVNYWDKQDHPISRLRQYLLNQGWWDEEQEKAWRKQSRKKVMEAFEQAERKLKPNPSLLFSDVYQEMPAQLRRQQESLARHLQTYGEHYPLDHFEK

>Felis catus (XP_003997819.1)

MAVAAAAAKLRGSGRGLGQAGVLLLRRLGARGLARYHPQRQEQQHFPSLDDKPQFPGASAEFVDKLEFIQPNVISGIPVYRVMDRQGQIINPSEDPHLPQETVLKFYKSMTLLNTMDRILYESQRQGRISFYMTNYGEEGTHVGSAAALDSTDLVFGQYREAGVLMYRDYPLELFMAQCYGNASDLGKGRQMPVHYGCKERHFVTISSPLATQIPQAVGAAYAAKRANANRVVICYFGEGAASEGDAHAGFNFAATLECPVIFFCRNNGYAISTPTSEQYRGDGIAARGPGYGIMSIRVDGNDVFAVYNATKEARRRAVAENQPFLIEAMTYRIGHHSTSDDSSAYRSVDEVNYWDKQDHPISRLRHYLQGRGWWDDEQEKAWRKQSRKKVMEAFEQAERKPKPNPNLLFSDVYQEMPAQLRKQQESLARHLQAYGEHYPLDHFEK

>Drosophila melanogaster (NP_649905.1)

MSFLRKSVHLLGSVARRQRHLKVIEGAISTAARDFSSKPSEDYANFPGAKAPFVSKLNLIQPEDYAPIPIYRVMDQDGYIADETQDPQLGREVVEKMFRDMVLLNTMDKILYESQRQGRISFYMTNFGEEASHIGSAAALEMRDLIYGQYREAGVLVWRGFRIDQFIDQCYGNTDDLGRGKQMPVHYGSRELNFVTISSPLSTQMPQAVGAAYAMKLRPNNDACVVCYFGEGAASEGDAHAAFNFAATLGCPAILFCRNNGFAISTPSHEQYKGDGIAGRGPMGYGITTIRVDGTDVFAVYNAMKAAREYVLKENKPVVFEALAYRVGHHSTSDDSTAYRPAEEIEIWNSVEHPISKLKRYMVHKGWFDETVENEYVKDIRKKVLKQIAVSEKKLKPNWREMFEGVYAEMPDHLIEQRSELEKHIEAHKEHYPLKDFEK

>Caenorhabditis elegans (NP_001033377.1)

MMHRALLNASRRVATVRSMASTVEGDAFRLSEYSSKYLGHRKAAFTEKLEIVNADDTPALPIYRVTNAVGDVIDKSQDPNFDEQTSLKMYKTMTQLNIMDRILYDSQRQGRISFYMTSFGEEGNHVGSAAALEPQDLIYGQYREAGVLLWRGYTMENFMNQCYGNADDLGKGRQMPMHFGTKERNFVTISSPLTTQLPQAVGSAYAFKQQKDNNRIAVVYFGDGAASEGDAHAAFNFAATLKCPIIFFCRNNGYAISTPTSEQYGGDGIAGKGPAYGLHTIRVDGNDLLAVYNATKEARRVALTNRPVLIEAMTYRLGHHSTSDDSTAYRSSDEVQTWGDKDHPITRFKKYITERGWWNEEKEMEWQKEVKKRVLTEFAAAEKRKKAHYHDLFEDVYDELPLRLRRQRDELDAHVAEYKEHYPMLETLQSKP

________________________________________________________________________________

2-OXOGLUTARATE DECARBOXYLASE

>Mycobacterium (O50463.4)

MANISSPFGQNEWLVEEMYRKFRDDPSSVDPSWHEFLVDYSPEPTSQPAAEPTRVTSPLVAERAAAAAPQAPPKPADTAAAGNGVVAALAAKTAVPPPAEGDEVAVLRGAAAAVVKNMSASLEVPTATSVRAVPAKLLIDNRIVINNQLKRTRGGKISFTHLLGYALVQAVKKFPNMNRHYTEVDGKPTAVTPAHTNLGLAIDLQGKDGKRSLVVAGIKRCETMRFAQFVTAYEDIVRRARDGKLTTEDFAGVTISLTNPGTIGTVHSVPRLMPGQGAIIGVGAMEYPAEFQGASEERIAELGIGKLITLTSTYDHRIIQGAESGDFLRTIHELLLSDGFWDEVFRELSIPYLPVRWSTDNPDSIVDKNARVMNLIAAYRNRGHLMADTDPLRLDKARFRSHPDLEVLTHGLTLWDLDRVFKVDGFAGAQYKKLRDVLGLLRDAYCRHIGVEYAHILDPEQKEWLEQRVETKHVKPTVAQQKYILSKLNAAEAFETFLQTKYVGQKRFSLEGAESVIPMMDAAIDQCAEHGLDEVVIGMPHRGRLNVLANIVGKPYSQIFTEFEGNLNPSQAHGSGDVKYHLGATGLYLQMFGDNDIQVSLTANPSHLEAVDPVLEGLVRAKQDLLDHGSIDSDGQRAFSVVPLMLHGDAAFAGQGVVAETLNLANLPGYRVGGTIHIIVNNQIGFTTAPEYSRSSEYCTDVAKMIGAPIFHVNGDDPEACVWVARLAVDFRQRFKKDVVIDMLCYRRRGHNEGDDPSMTNPYVYDVVDTKRGARKSYTEALIGRGDISMKEAEDALRDYQGQLERVFNEVRELEKHGVQPSESVESDQMIPAGLATAVDKSLLARIGDAFLALPNGFTAHPRVQPVLEKRREMAYEGKIDWAFGELLALGSLVAEGKLVRLSGQDSRRGTFSQRHSVLIDRHTGEEFTPLQLLATNSDGSPTGGKFLVYDSPLSEYAAVGFEYGYTVGNPDAVVLWEAQFGDFVNGAQSIIDEFISSGEAKWGQLSNVVLLLPHGHEGQGPDHTSARIERFLQLWAEGSMTIAMPSTPSNYFHLLRRHALDGIQRPLIVFTPKSMLRHKAAVSEIKDFTEIKFRSVLEEPTYEDGIGDRNKVSRILLTSGKLYYELAARKAKDNRNDLAIVRLEQLAPLPRRRLRETLDRYENVKEFFWVQEEPANQGAWPRFGLELPELLPDKLAGIKRISRRAMSAPSSGSSKVHAVEQQEILDEAFG

>Mycobacterium marinum (YP_001852457.1)

MRRTAAVANISSPFGQNEWLVEEMYRKFRDDPSSVDPSWHEFLVDYNPESTQEATEPAVEKPAVAPAKPAAAPAKPAPAPAPAKPAAGPPAAGNGSPAAAPSAKPAAAPAKAPAPPPAEGDEMQVLRGAAAAVVKNMSASLDVPTATSVRAVPAKLLIDNRIVINNQLKRNRGGKISFTHLLGYALVQAVKKFPNMNRHYLDVDGKPNAVTPAHTNLGLAIDLQGKDGKRALVVAGIKRCETMRFAQFVTAYEDIVRRARDGKLTAEDFSGVTISLTNPGTIGTVHSVPRLMAGQGAIIGVGAMEYPAEFQGASEERIAELGIGKLITLTSTYDHRIIQGAESGDFLRTIHQMLLADEFWDEIFRELSIPYLPVRWRPDNPDSIVDKNARIIELIAAYRNRGHLMADIDPLRLDKTRFRSHPDLDVCTHGLTLWDLDRSFKVGGCFAGPQNMKLRDVLSILRDAYCRHVGVEYTHILEPEQQQWLQQRVEAKHVKPTVAQQKYVLSKLNAAEAFETFLQTKYVGQKRFSLEGAESVIPMMDAAIDQCAEYGLDEVVIGMPHRGRLNVLANIVGKPYSQIFSEFEGNLNPSQAHGSGDVKYHLGATGVYLQMFGDNDIQVSLTANPSHLEAVDPVLEGLVRAKQDLLEHGETDTENQRAFSVVPMMLHGDAAFAGQGVVAETLNLANLPGYRVGGTIHIIVNNQIGFTTAPEYSRSTEYCTDVAKTIGAPIFHVNGDDPEACVWVARLAVDFRQRFNKDVIIDMLCYRRRGHNEGDDPSMTNPRMYDVVDTKRGVRKSYTEALIGRGDISIKEAEDALRDYQGQLEQVFNEVRELEKHGAQPSESVESDQMIPAGLATAVDKSLLARIGDAFLAVPDGFTTHPRVQPVLEKRREMAYEGKIDWAFAELLALGSLVAEGKLVRFSGQDTRRGTFSQRHSVIIDRHTREEFTPLQLLTTNKDGSPTGGKFLVYDSPLSEYAAVGFEYGYTVGNPDAVVLWEAQFGDFVNGAQSIIDEFISSGEAKWGQLSNVVLLLPHGHEGQGPDHTSGRIERFLQLWAEGSMTIAMPSTPSNYFHLLRRHALDGIQRPLIVFTPKSMLRNKAAVSDIKDFTEIKFRSVLEEPTYEDGVGDRNLVNRILLTSGKIYYEMVARKAKDKREDVAIVRVEQLAPLPRRRLRETLDRYPNAKEFFWVQEEPANQGAWPRFGLELPELLPEKLSGVKRISRRAMSAPSSGSSKVHAVEQQEILDTAFG

________________________________________________________________________________

OGDC from cyanobacteria

>Synechococcus (YP_001735999.1)

MNTAELLIRCLENEGVEYIFGLPGEENLHILEALKESPIRFITVRHEQGAAFMADVYGRLTGKAGVCLSTLGPGATNLMTGVADANLDGAPLIAITGQVGTDRMHIESHQYLDLVAMFAPVTKWNKQIVRPNTTPEVVRRAFKIAQQEKPGAVHIDLPENIAAMPVEGQPLQRDGREKIYASSRSLNRAAEAIAHAKSPLILVGNGIIRADAAEALTDFATQLNIPVVNTFMGKGAIPYTHPLSLWTVGLQQRDFVTCAFEQSDLVIAVGYDLIEYSPKRWNPEGTTPIIHIGEVAAEIDSSYIPLTEVVGDIGDALNEIRKRTDREGKTAPKFLNVRAEIREDYERHGTDASFPVKPQKIIYDLRQVMAPEDIVISDVGAHKMWMARHYHCDRPNTCLISNGFAAMGIAIPGAVAAKLVYPEKNVVAVTGDGGFMMNCQELETALRIGANFVTLIFNDGGYGLIGWKQINQFGAPAFVEFGNPDFVQFAESMGLKGYRITAAADLVPTLKEALAQDVPAVIDCPVDYSENVKFSQKSGDLICRM

>Leptolyngbya (YP_007070940.1)

MNTAELLVKCLENEGVEYIFGLPGEENLHILEALKDSSIQFITVRHEQGGAFMADVYGRLTGKAGVCLSTLGPGATNLMTGVADANLDGAPLIAITGQVGTDRMHIESHQYLDLVAMFGPVTKWNRQIVRPSTTPEVVRRAFKIAQQEKPGAVHIDLPENIAAMETEGLPLTKASNEKIYASSRSLNQAAQAIARSKNPVILAGNGAIRAKAAEELTEFATKLNIPVVNTFMGKGAIPYTHPLALWTIGLQQRDFITCAFEQSDLVIAVGYDLIEYSPKRWNPDGSTPIIHVGEAAAEIDSSYIPLAEVVGDIGDSLSEIMKRADREGKPLPSYASVRSDIREDYEFYANDKGFPVKPQKIIYDLRQVMAPEDIVISDVGAHKMWMARHYHCERPNTCLISNGFAAMGIAIPGALAAKLIYPEKKIVAVTGDGGFMMNNQELETALRVGTNFVTLIFSDSCYGLIGWKQINQFGQPAFVDFTNPDFVKFAESMGLKGYRIEAADDLIPTLEEAFKQDVPVVIDCPVDYGENVKFSQKAGDLICRI

>Halothece (YP_007169058.1)

MNTAELLVKCLENEGVEYIFGLPGEENLDLLEALKDSSIQFITVRHEQGAAFMADVYGRLTGKAGVCLSTLGPGATNLMTGVADANLDGAPLIAITGQVGTDRMHIESHQYLDLVAMFAPVTKWNKQIVRPNTTPELLRRAFKIAQQEKPGAVHIDLPENIAAMPAEGLPLAKDGREQIYASSGSIEKAAEVISKADNPVILAGNGVIRSQAAQQLTTFATRLNIPVVNTFMGKGAIPYTHPLSLWTVGLQQRDFISCAFEQSDLVIAIGYDLIEYSPKRWNPNGTTPILHIAETASEIDSSYIPLGEVVGDIGNSLDQIIEQVHRDGKPLPIFANVRSEIRADYEFYAHDEEFPIKPQKIIYDLRQVMGAQDIVISDVGAHKMWMARHYHCESPNTCLISNGFAAMGISIPGALAAKLLYPEQNVVAVTGDGGFMMNCQELETALRVGTNFVTLIFNDSGYGLIGWKQMNHFGASSFIEFGNPDFVKFADSMGLKGYRVESVADLIPTLKEAQSQNVPTVIDCPVDYRENVKFSKKAGELICRI

>Microcoleus (YP_007122761.1)

MGEINTAELLVRCLENEGVEYVFGLPGEENLHVLEALRHSSIKFITTRHEQGAAFMADLYGRLTGKAGVCLSTLGPGATNLMTGVADANLDGAPLVAITGQVGTDRMHIESHQYLDLVAMFAPVTKWNAQIVRPSNTAEIVRKAFKIAQSEKPGAVHIDLPENIAAMPVLGQPLAKDEREKTYASFRSIGKAAVAISKAKNPLILVGNGAIRAGASEALTEFATRMNIPVVNSFMGKGVIPYTHPLALWTVGLQQRDHVSCAFDQADLIIAVGYDLVEYSPKKWNPEGKIPIIHIGMSPAEIDSSYIPLVEVVGDISDSLAEIMKQADREGKTIPYAVELRADIRADYELYANDEGFPIKPQKLIYDLRQVMGPDDIVISDVGAHKMWMARQYHCDRPNTCIISNGFAAMGIALPGAIGAKLVYPERNVVAVTGDGGFMMNCQELETALRVGTPFVTLIFNDGGYGLIEWKQQNHFGDSAFIRFGNPDFVKFAESMGLKGYRVNSAAELIPTLKEALAQDVPTVIDCPVDYGENLRFTQKAGDLSCKI

>Arthrospira platensis (YP_005071308.1)

MNTAELLVKCLENEGVKYVFGLPGEENMEVLESLKDSSIQFITTRHEQGAAFMADVYGRLTGKAGVCLSTLGPGATNLMTGVADANLDGAPLVAITGQVGTDRMHIESHQYLDLVAMFSPVTKWNAQIVRPSITPEIVRKAFKLAQTEKPGAVHIDLPENIASMAVEGQPLNRDRREKIYSAYQSMNEAASAISKAVNPLILVGNGALRANASEAVTEFATQLNIPVANTFMGKGAIPYTHPLSLWTTGLQQRDFISCAFDKADLVIAIGYDLIEYSPKKWNPKGTIPIIHVGANAAEIDSSYIPIAEIVGDISDSLNEILRRSDRTGKLDPYGVKLRSDIRQDYERYANDDGFPIKPQKIIYDLRQVMGPEDIVISDVGAHKMWMARHYHSDRPNTCLISNGFAAMGISIPGAIAAKLVNPELKVVAVTGDGGFMMNCQELETALRVGTPFVTLIFNDGGYGLIEWKQEDQFGSSSFIKFGNPDFVKFAESMGLKGYRVEAATDLVPILKEALASEVPVVIDCPVDYRENARFSQRAGGLCCPI

________________________________________________________________________________

ACETOLACTATE SYNTHASE

>Eupatorium cannabinum (CAX48741.1)

MATSLILKTSISGDSPISSRPFQSKPKLFLPLTLSNPKKTGLKFSITSALSDAKPIATTSPSPPPKAFVSRFGPDQPRKGSDVLVEALEREGVTNVFAYPGGATLEIHQALTRSSTIRNILPRHEQGGVFAAKGYARASGLTGVCIATSGPGATNLVSGLADALLDSVPLVAITGQVPRKMIGIDAFQETPIIEVTRSITKHNYLVLNVQDIPRIVQEAFYLASTGRPGPVLIDIPKDIQQQLVVPNWDEPMRLGGYLSRLPKPPNESHLQQIVRFIKESRKPVLYVGGGCVNASDELGHFVELTGIPVTNTLMGLGTYPGSHDLSLQMLGMHGTVYANYAIDKCDLLLAFGVRFDDRVTGKIETFASQAKIVHIDIDAAEIGKNKQPHLSICGDIKIALQEINKIMDRGEYKNLDFSTWKQELMDQKVSNPLTFKTFGDAIPPQYAIQVLDEMTNGNAIISTGVGQHQMWSAQFYKYNRPRQWLTSGGLGAMGFGLPAAIGAAVARPDAVVVDIDGDGSFMMNVQELATIRAENLPVKMMVLNNQHLGMVVQWEDRFYKANRAHTYLGNPTKESEIFPNMLKFAEACDIPSARVTKMKDLRTAIQKMLDTPGPYLLDVIVPHQEHVLPMIPAGGGFKDIITEGDGRTQT

>Cucumis sativus (XP_004138045.1)

MSSMAAAASPCASKSFSKSPSFNLTKPFSRFDLSFSSLPHNPTFRRSLRICSTSLSNPSPKPSSTAASAATVETSSITSSEIFASRFAADEPRKGADILVEALERQGVTNVFAYPGGASMEIHQALTRSSIIRNVLPRHEQGGVFAAEGYARSSGLPGVCIATSGPGATNLVSGLADALLDSVPLVAITGQVPRRMIGTDAFQETPIVEVTRSITKHNYLVLDVDDIPRIVSEAFFLASSGRPGPVLIDIPKDVQQQLAVPNWNQPMKLPGYLSRLPKPPTDSHLEQILRLISESKKPVLYVGGGCLNSSEELRRFVKLTGIPVASTLMGLGAYPCSDDLSLQMLGMHGTVYANYAVDKSDLLLAFGVRFDDRVTGKLEAFASRAKIVHIDIDSAEIGKNKQPHVSVCGDVKLALQGMNRLLEKAHMLHFDFSAWREELNEQKSKYPLTFKTFDEAIPPQYAIQLLDELTNGEAIVSTGVGQHQMWAAQFYKYKKPRQWLTSGGLGAMGFGLPAAMGAAVANPGAVVVDIDGDGSFIMNVQELATISVEKLPVKILLLNNQHLGMVVQWEDRFYKANRAHTYLGNPSNESEIFPNMLKFAEACGIPAARVTKKAELRAAMKKMLETEGPYMLDVIVPHQEHVLPMIPSGGAFKDVITEGDGRRSY

>Ricinus communis (XP_002511176.1)

MAATSAATATSIPKPSAAPSSSTSTSRSPISISRFTLPFSLNPQKSTAHRSLHITNAISKPATTIPATTAPVPQPSPNPRFAPDEPRKGSDILVEALERQGVTDVFAYPGGASLEIHQALTRSPIIRNVLPRHEQGGVFAAEGYARSSGKPGVCIATSGPGATNLVSGLADALLDSVPIVAITGQVPRRMIGTDAFQETPIVEVTRSITKHNYLVLDVDDIPRIVQEAFFLATSGRPGPVLIDVPKDIQQQLAVPNWNTPIKLPGYMSRLPKVPNDSHLEQIVRLISESKKPVLYVGGGCLNSSEELRRFVELTGIPVASTLMGLGAFPVGDELSLQMLGMHGTVYANYSVDKSDLLLAFGVRFDDRVTGKLEAFASRAKIVHIDIDSAEIGKNKQPHVSVCGDVKLALQGMNKILESKGAKSKLDFKAWREELNEQKVKYPLSFKTFGDAIPPQYAIQVLDELTNGNAIISTGVGQHQMWAAQFYKYKRPRQWLTSGGLGAMGFGLPAAIGAAVANPGAVVVDIDGDGSFIMNVQELATIRVENLPIKILLLNNQHLGMVVQWEDRFYKANRAHTYLGDPSNESEIFPNMLKFAEACGIPAARVTRKEDLRAAIQEMLDTPGPYLLDVIVPHQEHVLPMIPSGGAFKDVITEGDGRTQY
