# Supplementary material for: Evolution and Functional Implications of the Tricarboxylic Acid Cycle as Revealed by Phylogenetic Analysis
Source: Genome Biol Evol. 2014 Oct 1;6(10):2830–48. doi: 10.1093/gbe/evu221 (PMC4224347; doi:10.1093/gbe/evu221)
Supplement: Supplementary Data [file supp_evu221_Supplemental_DataSet_2.doc]

CITRATE SYNTHASE

PEROXISSOME

>Arabidopsis thaliana p1 (NM_115736.2)

MEISERARARLAVLNAHLTVSEPNQVLPAIEPWCTSAHITAAPHGSLKGNLKIVDERTGNEYQVPVSEHGTVKTVDLKKITTGKDDKGLNLYDPGYLNTAPVRSSISYIDGDEGILRYRGYPVEELAEKSTYTEVTYLLIYGNLPSQRQLADWEFAISQNSAVPQGVLDMIQSMPNDVHPVGALVTAMSALSIFYPDANPSLMGLGVYKSKQVRDKQIVRVLGQAPTIAAAAYLRKAGKPPVQPLSNLSYSENFLYMVESMGDRSYKPNPRLARVLDILFILQAEHEMNCSTAAARHLSSSGGDVYTAVSGGVGAIYGPLHGGAVEATINMLSEIGTVENIPEFIESVKNKKRRLSGFGHRIYKNYDPRGKVVKKLADEVFSILGRDPLVEVGDALEKAALSDEYFVKRKLYPNVDFYSGLINRAMGIPSSFTAVSRIAGYLSHWRESLDDPDTKIMRPQQVYTGAGIRHYETVRERTKL

>Arabidopsis thaliana p2 (NM_115737.2)

MEISQRVKARLAVLTAHLAVSDTVGLEQVLPAIAPWCTSAHITAAPHGSLKGNLTIVDERTGKKYQVPVSEHGTVKAVDLKKITTGKDDKGLKLYDPGYLNTAPVRSSICYIDGDEGILRYRGYPIEELAESSTFIEVAYLLMYGNLPSQSQLADWEFTVSQHSAVPQGVLDIIQSMPHDAHPMGVLVSAMSALSIFHPDANPALSGQDIYKSKQVRDKQIVRILGKAPTIAAAAYLRTAGRPPVLPSANLSYSENFLYMLDSMGNRSYKPNPRLARVLDILFILHAEHEMNCSTAAARHLASSGVDVYTACAGAVGALYGPLHGGANEAVLKMLAEIGTAENIPDFIEGVKNRKRKMSGFGHRVYKNYDPRAKVIKKLADEVFSIVGRDPLIEVAVALEKAALSDEYFVKRKLYPNVDFYSGLIYRAMGFPPEFFTVLFAVPRMAGYLSHWRESLDDPDTRIMRPQQAYTGVWMRHYEPVRERTLSSDSDKDKFGQVSISNASRRRLAGSSAL

>Arabidopsis thaliana p3 (NM_129840.4)

MEISERVRARLAVLSGHLSEGKQDSPAIERWCTSADTSVAPLGSLKGTLTIVDERTGKNYKVPVSDDGTVKAVDFKKIVTGKEDKGLKLYDPGYLNTAPVRSSISYIDGDEGILRYRGYPIEEMAENSTFLEVAYLLMYGNLPSESQLSDWEFAVSQHSAVPQGVLDIIQSMPHDAHPMGVLVSAMSALSIFHPDANPALRGQDIYDSKQVRDKQIIRIIGKAPTIAAAAYLRMAGRPPVLPSGNLPYADNFLYMLDSLGNRSYKPNPRLARVLDILFILHAEHEMNCSTAAARHLASSGVDVYTAVAGAVGALYGPLHGGANEAVLKMLSEIGTVENIPEFIEGVKNRKRKMSGFGHRVYKNYDPRAKVIKNLADEVFSIVGKDPLIEVAVALEKAALSDDYFVKRKLYPNVDFYSGLIYRAMGFPPEFFTVLFAIPRMAGYLSHWKESLDDPDTKIMRPQQVYTGVWLRHYTPVRERIVTDDSKESDKLGQVATSNASRRRLAGSSV

>Arabidopsis lyrata p1 (XP_002878237.1)

MEIPERASARLAVLTAHLAASEPTQVLPTIEPWCTSSHISATPHGSLEGNLTIVDERTGKKYQVPVSEHGTVKAVDLKKITTGEDDKGLKLYDPGYLNTAPVRSSISYIDGDEGILRYRGYSVEELAEKSTYTEVTYLLIYGNLPSQRQLADWEFAISQNSAVPQGVLDLIQSMPHDVHPIGALVTAMSALSIFYPDANPSLMGLGVYQSKQVRDKQIFRILGQAPTIAAAAYLRKAGKPPVQPLSNLSYSENFLYMVESMGNKSYKPNPRLARVLDIIFILHVEHEMNCSTAAARHLSSSGVDVYTAVAGGVGAIYGPLHGGAIEATVNMLSEIGTVENIPEFIESVKNKKRKLSGFGHRVYKKYDPRGKVVKKLADEVFSILGREPLFEVGDALEKAALSDEYFIKRKLYPNADFYSGLIYRAMGIPSSFMAVARIAGYLSHWRESLDDPDTKIMRPQQVYTGMGLRRYELVRERTKL

>Arabidopsis lyrata p2 (XP_002878238.1)

MEISQRVKARLAVLTAHLAVSDTVGLEQVLPAIEPWCTSAHITAAPHGSLKGNLTIVDERTGKKYQVPVSEHGTVKAVDLKKITTGKDDKGLKLYDPGYLNTAPVRSSICYIDGDEGILRYRGYPIEELAESSTFIEVAYLLMYGNLPSQSQLADWEFTVSQHSAVPQGVLDIIQSMPHDAHPMGVLVSAMSALSIFHPDANPALSGQDIYKSKQVRDKQIVRILGKAPTIAAAAYLRTAGRPPVLPSGNLSYSENFLYMLDSMGNRSYKPNPRLARVLDILFILHAEHEMNCSTAAARHLASSGVDVYTACAGAVGALYGPLHGGANEAVLKMLAEIGTAENIPDFIEGVKNRKRKMSGFGHRVYKNYDPRAKVIKKLADEVFSIVGRDPLIEVAVALEKAALSDEYFVKRKLYPNVDFYSGLIYRAMGFPPEFFTVLFAVPRMAGYLSHWRESLDDPDTRIMRPQQAYTGVWMRHYEPVRERTLSSDSGTDKFGQVSISNASRRRLAGSSAL

>Arabidopsis lyrata p3 (XP_002881873.1)

MEISERVRARLAVLSAHLAEGKQDSPAIERWCTSADTSVAPLGSLKGTLTIVDERTGKKYKVPVSDDGTVKAVDFKKIATGKEDKGLKLYDPGYLNTAPVRSSISYIDGDEGILRYRGYPIEEMAENSTFLEVAYLLMYGNLPSETQLSDWEFAVSQHSAVPQGVLDIIQSMPHDAHPMGVLVSAMSALSIFHPDANPALRGQDIYDSKQVRDKQIIRIIGKAPTIAAAAYLRMAGRPPVLPSGNLPYADNFLYMLDSLGNRSYKPNPRLARVLDILFILHAEHEMNCSTAAARHLASSGVDVYTAVAGAVGALYGPLHGGANEAVLKMLSEIGTVENIPEFIEGVKNRKRKMSGFGHRVYKNYDPRAKVIKNLADEVFSIVGKDPLIEVAVALEKAALSDDYFVKRKLYPNVDFYSGLIYRAMGFPPEFFTVLFAIPRMAGYLSHWKESLDDPDTKIMRPQQVYTGVWLRHYTPVRERIVTDDSKESDKLGQVSTSNASRRRLAGSSV

>Fragaria vesca subsp. vesca (XP_004306283.1)

MSAEFPVRARGRLAVLTAHLAAATIAEPTELEPQCLSAAVPPPGNLCGGLTIVDERTGKRYQVQVSEQGTIKATDLKKITTGKNDKGLKLYDPGYLNTAPVRSSICYIDGDEGILRYRGYPIEELAESSTFLEVAYLLMYGNLPTQSQLADWEFAVSQHSAVPQGILDIIQAIPHDAHPMGVLVSAMSALSVFHPDANPALRGQDLYQSKQVRDKQIARILGKAPTIAAAAYLRLAGRPAVLPSNNLSYSENFLYMLDSLGNRAYKPNPRLARVLDILFILHAEHEMNCSTAAARHLASSGVDVYTALAGAVGALYGPLHGGANEAVLKMLSEIGSVENIPEFIEGVKSRKRKMSGFGHRVYKNYDPRAKVIRKLADEVFSIVGRDPLIEVAVALEKAALSDEYFVKRKLYPNVDFYSGLIYRAMGFPTEFFPVLFAIPRMAGYLSHWRESLDDPDTKIMRPAQVYVGAWLRHYMPLKERIVSSDGDKLSQVSVSNASKRRLSGSGV

>Vitis vinifera (XP_002284064.1)

MERADSSALARSRLAILSAHLEACLKSSSFGSVIQPSCVSAQSGIQPPGDLKGSLTIVDDRTAKKYQVQVSSHGTVKATDLKKITTGNNDKGLKLYDPGYLNTAPVRSSICYIDGDAGILRYRGYPIEELAESSSFLEVAYLLMYGNLPSESQLADWEFAVSQHSAVPQGILDIIQAMPHDAHPMGVLVSAMSALSVFHPDANPALRGQDLYQSKQVRDKQIARVLGKAPTIAAAAYLRMAGRPPVLPSSSFSYSENFLYMLDSLSNRSYKPNPRLARVLDILFILHAEHEMNCSTAAARHLSSSGVDVYTALAGAVGALYGPLHGGANEAVLKMLSEIGTIENIPEFIEGVKNRKRKMSGFGHRVYKNYDPRAKVIKKLAEEVFSIVGRDPLIEVAVALEKAALSDEYFIKRKLYPNVDFYSGLIYRAMGFPTEFFPVLFAIPRMAGYLAHWRESLDDPDTKIMRPQQVYTGEWLRHFMPVKERMMSAEADRLGQVSISNATRRRLAGSGM

>Populus trichocarpa (XP_002320409.1)

MSKSDSSPETARSRLAVLTAHLVGATTLESSSSSINRSCVSAQVSPPGNLRGALTVIDERTGKKYQIPVSQDGTVKASDFKKISTGKNDKGLKLYDPGYLNTAPVRSSISYIDGDEGILRYRGYPIEELAESSTFVEVAYLVMYGSLPSQSQLADWEFAILQHSALPQGVLDIIQAMPHDAHPMGVLVSAMSALSIYHPDANPALRGQDLYKSKQVRDKQIARILGKAPTIAAAAYLRLAGRPPVIPSSNLSYSENFLYMLDSLGDRSYKPNPRLARVLDVLFILHAEHEMNCSTSAARHLASSGVDVYTALAGAVGALYGPLHGGANEAVLKMLSEIGTVENIPSFLEGVKNRKRKMSGFGHRVYKNYDPRAKVIKKLAEEVFSIVGRDPLIEVAVALEKAALDDEYFVKRKLYPNVDFYSGLIYRAMGFPTEFFPVLFAIPRMAGYLAHWRESLDDPDTKIMRPQQVYTGEWLRHYMPLKEREASSNADKPGQISVSNASMRRRAGSRV

>Populus trichocarpa (XP_002322875.1)

MSESSIIARGRLAVLTAHLLGAPLESSEDSDIQRWCVSAQVPTPGDLKGVLTVIDERTGKKYQIQVSQDGTVKASDFKKISTGKSDKGLKLYDPGYLNTAPVISKISYIDGDEGILRYRGYPIEELAESSTFLEVAYLILYGNLPSQSQLADWEFAISQHSAVPQGVLDIIQAMPHDAHPMGVLVSAMSSLSIFHPDANPALRGQDLYKSKQVRDKQIARILGKAPTIAAAAYLRLAGRPPVLPSSNLSYSENFLYMLDSLGNRSYKPNPRLARVLDILFILHAEHEMNCSTSAARHLASSGVDVYTALAGAVGALYGPLHGGANEAVLKMLSEIGTIENIPDFIEGVKNRKRKMSGFGHRVYKNYDPRAKVIKKLAEEVFSIVGRDPLIEVAVALEKAALADEYFVKRKLYPNVDFYSGLIYRAMGFPTEFFPVLFAIPRMAGYLAHWRESLDDPDTKIMRPQQVYTGEWLRHYMPLKERMIQTDADRLGQVSISNASRRRLAGSGV

>Cucumis sativus (XP_004143228.1)

MAVPETGHQKLFGGGHRKKSLEGCWQWSSEIDGQKNGDLRLLATVGGKRLLEVGRWKMINRSWLLTIAEKWSLEGGIGGDRRRLQGNCGRRKKKLEIPTDMELSPSNVARGRLAVLAAHLSAATLESPVMASLLEANCVSARTMLPPPEALKGTLTIVDERTGKRYQVQISEEGTIKATDLKKITTGPNDKGLKLYDPGYLNTAPVRSSISYIDGDEGILRYRGYPIEELAESSTYVEVAYLLMYGNLPSQSQLADWEFALSQHSAVPQGLLDIIQAMPHDAHPMGVLVSAMSALSVFHPDANPALRGQDLYKSKQVRDKQIARIIGKAPTIAAAAYLRLAGRPPVLPSSNLSYSENFLYMLDSLGNRSYKPNPRLARVIDILFILHAEHEMNCSTSAARHLASSGVDVFTALSGAVGALYGPLHGGANEAVLKMLSEIGTVDNIPGFIEGVKNRKRKMSGFGHRVYKNYDPRAKVIRKLAEEVFSIVGRDPLIEVAVALEKAALSDEYFVKRKLYPNVDFYSGLIYRAMGFPPEFFTVLFAIPRMAGYLAHWRESLDDPDTKIIRPQQVYTGEWLRHYIPPKERLVPAKADRLGQVSVSNASKRRLSGSGI

>Solanum lycopersicum p1 (XP_004251813.1)

MENGNSSSVARGRLAVLSAHLAASLNVSDLTTFKLLETSGVSSVSGVEPPRNLKGALTIIDERTGKKYPVQVSEDGTIKANDLKKITTGQNDKGLKLYDPGYLNTAPVRSSICYIDGDAGILRYRGYPIEELAEGSSFLEVAYLLLYGNLPSENQLADWEFTVSQHSAVPQGLLDIIQSMPHDAHPMGVLVSAMSALSVFHPDANPALRGQDIYKSKQVRDKQIVRILGKAPTIATAAYLRMAGRPPVLPSNNLSYAENFLYMLDSLGNRSYKPNPRLARVLDILFILHAEHEMNCSTAAARHLASSGVDVYTAIAGAVGALYGPLHGGANEAVLKMLSEIGSVENIPEFLEGVKNRKRKMSGFGHRVYKNYDPRAKVIKTLADEVFSIVGRDPLIEVAVALEKAALSDEYFVKRKLYPNVDFYSGLIYRAMGFPTEFFPVLFAIPRMAGYLSHWNESLDDPDTKIMRPAQVYTGVWMRHYMPLKERSPHSEADKLGHVSVSNATKRRLAGSGA

>Solanum lycopersicum p2 (XP_004243239.1)

MERGKSSAVARSRLSVITAHLAADYSASSHGLETSSVSAPTAAVSPPPNLKGALTIIDERTGKKYQVPVTEEGTVKATDFKKISTGYNDKGLKLYDPGYLNTAPVRSSICYIDGDAGILRYRGYPIEELAERSSFLEVAYLLMYGNLPSENQLSDWEFAVSHHSAVPQGVLDIIQSMPHDAHPMGVLVSAMSTLSVFHPDANPALRGQDIYNSKQVRDKQIVRILGKAPVIAAAAYLRMAGRPPVLPSNNLSYAENFLYMLDSLGNRSYKPNPRLARVVDILFILHAEHEMNCSTAAARHLASSGVDVYTAIAGAVGALYGPLHGGANEAVLRMLSEIASIDNIPEFIEGVKNRKRKMSGFGHRVYKNYDPRAKVIKKLAEEVFSIVGRDPLIEVAVALEKAALSDEYFVERKLYPNVDFYSGLIYRAIGFPTEFFPVLFAIPRMAGYLAHWRESLDDPDTKIMRPAQVYTGVWLRHYVPLRGRSPSTETDKFGQVSVSNATRRRLAGSGD

>Medicago truncatula p1 (XP_003599916.1)

MSTTTTTESKMHDAARNRLSTLTAHLLPSSTTSSAALLHPLHLSASSGISPPSNVKGTLTVVDERTGKKYSIEVSPDGTVKANDFKKISTGKNDKGLKLYDPGYLNTAPVRSTISYIDGDEGILRYRGYPIEELAEKSTFPEVSYLILYGNLPSASQLQEWEFAISQHSALPQGVLDLIQAMPQDAHPMGVLVNALSALSVFHPDANPALRGLDIYNSKQVRDKQIVRIIGKITTIAAAINLRLGGRPPVLPSNKLSYTENFLYMLDSLGNRSYKPNPRLTRALDIIFILHAEHEMNCSTSAVRHLASSGVDVYTAIAGGVGALYGPLHGGANEAVLKMLSEIGSVDNIPEFIEGVKARKRKLSGFGHRVYKNYDPRAKVLKKLTEEVFSIVGRDPLIEVAVALEKIALSDEYFVKRKLYPNVDFYSGLIYRAMGFPPEFFTILFAIPRMAGYLAHWRESLDDPDTKIMRPQQVYVGEWLRHYAPTKERTVPSGSNTDKLGQLSVSNASKRRLAGSGI

>Medicago truncatula p2 (XP_003617470.1)

MSTDSQPSTVLSLARLATLTAHLLPSGNQQPSDHLLPHPLLAQSPVANLNGTLTIVDERTGKKYQVQISPDATVKATDLKKITTGKNDKGLKLYDPGYLNTAPVRSTISYIDGDEGILRYRGYPIEELAEKSTFMEVSYLIMYGSLPTESQLAEWNFAISQHSAVPQGVLDIIQSMPHDAHPMGVLVNAISALSVFHPDANPALQGLDIYNSKEVRDKQIARIIGKITTIAATVYLRMAGRPPVLPSNQLSYTENFLYMLDSLGNRSYKPNPQLTRALDIIFILHAEHEMNCSTSAVRHLASSGVDVYTAIAGAVGALYGPLHGGANEAVLKMLSEIRTVENIPEFIEGVKTRKRKLSGFGHRVYKNYDPRAKVLKKLTEEVFSIVGRDPLIEVAVALEKVALSDEYFIKRKLYPNVDFYSGLIYRAMGFPPEYFTVLFAIPRMAGYLSHWRESLDDPDTKIMRPQQVYIGEWLRNYMPIKQRTESSDADKLGQVTISNASKRRLAGSRM

>Glycine max p1 (XP_003532107.1)

MSKTTTSSEQTARNRLATLAAHLLPSDATSAAAILHPHRLSAPTGDLPPANLKGTLTVVDERTGKKYQIEVTPDGTVRASDFKKISSGKNDKGLKLYDPGYLNTAPVISRISYIDGDAGILRYRGYPIEDLAEKSTFTEVSYLILYGNLPSESQLAEWEFALSQHSAVPQGVLDMIESMPHDAHPMGMLVNAMSALSVFHPDANPALKGLDIYNSKQVRDKQIARVIGKITTIAAAINLRLAGRPPVLPSNKLSYTENFLYMLDSFGNRSYKPNPRLTRALDIIFVLHAEHEMNCSTSAVRHLSSSGVDVYTAIAGAVGALYGPLHGGANEAVLKMLSEIGTVENIPAFIEGVKARKRKLSGFGHRVYKNYDPRAKVLRKLTEEVFSIVGRDPLIEIAVALEKIALSDEYFIKRKLYPNVDFYSGLIYRAMGFQPEFFTVLFAIPRMAGYLAHWRESLDDPDTKIMRPQQVYVGEWLRHYAPINQRTASSNTDKLGQLAVSNASKRRLAGSGA

>Glycine max p2 (XP_003519557.1)

MSSNSDAAAATVHAHGRLAMLAAHLLPSQLTHHGALHPLHLSSQLPPPPNLAGTLTVVDERTGKKYQLHVSKEGTVKASEFKKILTGKNDKGLKLYDPGYLNTAPVRSTISYIDGDEGILRYRGYPIEELADKSTFTEVSYLIMYGSLPSESQLAEWEFAISQHSAVPQGVLDMIQSMPHDAHPMGVLVNAMSALSVFHPDANPALRGLDIYDSKQIRDKQITRVIGKITTIAAAVYLRMAGRPPVLPSNLLSYTENFLYMLDSFGNRSYKPNPQLTRALDIIFILHAEHEMNCSTSAVRHLASSGVDVYTAIAGAVGALYGPLHGGANEAVLKMLSEIGTVQNIPEFIEGVKARKRKLSGFGHRVYKNYDPRAKVLRKLAEEVFSIVGRDPLIEVAVSLEKIALSDEYFIKRKLYPNVDFYSGLIYRAMGFPPEYFTVLFAIPRMAGYLAHWRESLDDPDTKILRPQQVYVGEWLRHYTQVNVRTTSSDADKLGEVAISNASKRRLAGSGV

>Zea mays (AFW71216.1)

MDRADPARGRLAVLSSHLRGAGAEEAAGLERSPVSAPAPGPRAGALAVVDGRTGKRHEVKVSEDGTVRATDFKKITTGKDDKGLKIYDPGYLNTAPVRSSICYIDGDEGILRYRGYPIEELAESSSFVEVAYLLMYGNLPTQSQLAGWEFAISQHSAVPQGLLDIIQSMPHDAHPMGVLASAMSTLSVFHPDANPALQGQDLYKSKQVRDKQIVRVLGKAPTIAAAAYLRLAGRPPVLPLNTLSYSENFLYMLDSLGDRTYKPNPRLARALDILFILHAEHEMNCSTAAVRHLASSGVDVFTALSGGVGALYGPLHGGANEAVLKMLNEIGSMENIPDFIVGVKNRKRKMSGFGHRVYKNYDPRAKVIRKLADEVFSIVGRDPLIEVAIALEKAALSDEYFIKRKLYPNVDFYSGLIYRAMGFPTEFFPVLFAIPRMGGWLAHWKESLDDPDTKIIRPQQVYTGFWLRHYTPVRERVLSSQSEELGQVATSNATRRRRAGSAL

>Brachypodium distachyon (XP_003571847.1)

MDRADPARARLAVLSSHLLRTGADPAAVLERSPVSAAQATPPGTRAGALSVLDTRTGKRYEVKVSEDGTVRATDFKKITTGEDDKGLKTYDPGYLNTAPVRSSICYIDGDEGILRYRGYPIEEVAESSSFVEVAYLLMYGNLPTQSQLAGWEFAISQHSAVPQGLLDIIQAMPHDAHPMGVLASAMSTLSVFHPDANPALRGQDLYKSKQVRDKQIVRVLGKAPAIAAAAYLRLAGRPPVLPSNNLSYSENFLYMLDSLGNKEYKPNPRLARVLDILFILHAEHEMNCSTAAVRHLASSGVDVFTALSGAVGALYGPLHGGANEAVLKMLNEIGSVESIPDFIEGVKNRKRKMSGFGHRVYKNYDPRAKVIRKLAEEVFSIVGRDPLIEVAVALEKAALSDEYFIKRKLYPNVDFYSGLIYRAMGFPTEFFPVLFAIPRMAGWLAHWKESLDDPDNKIMRPQQVYTGVWLRHYTPVRERVPSSQSEQLGQIATSNATRRRRAGSAL

>Hordeum vulgare (BAK04892.1)

MDRTDPARGRLAVLSSHLLAAGVEGADPAAASLERSPVSAASPGTRAGVLAVVDSRTGKRYEVKVSEDGTVRATDFKKITTGKDDNGLKTYDPGYLNTAPVRSSICYIDGDEGILRYRGYPIEEVAESSSFVEVAYLLMYGNLPTQSQLAGWEFAISQHSAVPQGLLDIIQSMPHDAHPMGVLASAMSTLSVFHPDANPALRGQDLYKSKQVRDKQIVRVLGKAPAIAAAAYLRLAGRPAVLPSNNLSYSENFLYMLDSLGNKEYKPNPRLARVLDILFILHAEHEMNCSTAAVRHLASSGVDVFTALSGGVGALYGPLHGGANEAVLKMLNEIGAVENIPDFIEGVKNRKRKMSGFGHRVYKNYDPRAKVIRKLAEEVFSIVGRDPLIEVAVALEKAALSDDYFIKRKLYPNVDFYSGLIYRAMGFPTEFFPVLFAIPRMAGWLAHWKESLDDPDNKIMRPQQVYTGVWLRHYTPVRERVTSNQDEELGQIATSNATRRRRAGSSL

>Ostreococcus tauri (XP_003083992.1)

MAEDAAHRRVGVLSAHVGGAGAGEGDAVRLAPHGGRGVLRCTDSRTGKTYEVMIDDDGSVDASAFKKIIAGGDGRGLVLYDPGYMNTAPCKSKISYIDGDKGVLRYRGYPIETLAERSTYLETAFALVYGDLPNASQLMEWERTIARHSALPVQVVHAIEALPHDAHPMAVMLAGLNSLSAMHPEQNPAIAGGGIYDSHAVQDKQIVRIIGKMTTLAAHAYHRNTGRLPAAPNTRMSYAENFLYMLDAGLDSRHKPHPKLAKALDVMFLLHAEHEMNCSTAACRHLASSGVDVFCAVAGAVGALYGPLHGGANEAVLKMLERIGTVENIPSFLQGVKEKKYVMFGFGHRVYKNFDPRAKIIRQIANDVFDLVGRDPLIDVAIELEKAARADEYFVKRKLYPNVDFYSGLVYRAMGFPPEFFTVLFAIPRATGYLAHWRESLTDPDKKIMRPQQVYQGEWLRDYEPIGGRSKSLTDAMDHLSPSNAARRRMAGDPPAGSAWKGIEMSTKAWESGASVGDATSGLEHYIVRK

>Micromonas pusilla (XP_003064237.1)

MPTRASDDRATARVGAIRRHLDGGARDEDDVQRNPTAAAASAAAASTQRPSPGGGPGSLTVVDNRTKKKYQIPIQPGGYVAATAFRAITAGGDGAGLRLFDPGYMNTAPCKSKISYIDGDAGILRYRGYPIEELAEKSSYLESAFALVYGDLPSAPQLREWEETIMRHSALPVPVVQALEALPHDAHPMGVVLAGLNALSTFHPEQNPAVRGGNVYAEHGTQDKQIVRIIGKMTTLAAHAYHRNTGRTPAVPNQKLSYAENFLYMLDAGLDPHHRPNPRLAKALDVMFLLHAEHEMNCSTAAARHLASSGVDVYSAVAGAVGALYGPLHGGANEARSQHAHWSPYDRVGAVNAAVLKMLARIGTADAIPAFLEGVKNKKEKMSGFGHRVYKNFDPRANVIRGVAETVFSLVGRDPLIDVAVQLEKAARSDPYFVDRNLYPNVDFYSGLVYRALGFPPEFFTVLFAIPRAAGYLAHWREQLTDPDLKIMRPQQIYQGAWLRPYVDINARPSAAEDSMWQVEPSNASRRRLAGMSVDADAVAAAARDDMRPLGVAGRADSSGRRNPSAIDLSPAFSSGVTPGDATSGINNLLDGEMN

>Ostreococcus lucimarinus (XP_001421921.1)

MGDSRDVANRRLGQINDHVRGDAKRANGGARAGGGDGSDARATPSGGAGALHVRDSRTGKEYEIAVSSDGAVDASAFKQITAGGDGRGLVMYDPGYMNTAPCKSKISYIDGERGILRYRGYAIESLAKSSTYLETAFALVYGDMPNASQLMEWERTIARHSALPVQVVHAIEALPHDAHPMAVMLAGLNSLSAMHPEQNPAIAGGAIYNSHTVQDKQIVRIIGKMTTLAAHAYHRNTGRSPAAPNTRMSYAENFLYMLDAGLDARHRPHPKLAKALDVMFLLHAEHEMNCSTAACRHLASSGVDVFCAVAGAVGALYGPLHGGANEAVLKMLERIGSVDNIPSFLAGVKEKRYVMFGFGHRVYKNFDPRAKIIRDIANDVFELVGRDPLIDIAIELEKAARADEYFVKRKLYPNVDFYSGLVYRAMGFPPEFFTVLFAIPRATGYLAHWRESLTDADKKIMRPQQIYQGEWLRDYEPIAARSRSLTDAMEDIQPSNAARRRMAGDPPSGTAWVGKGVEMSTPAWQSGASVGDATSGVENYLGRK

>Saccharomyces cerevisiae (NP_009931.1)

MTVPYLNSNRNVASYLQSNSSQEKTLKERFSEIYPIHAQDVRQFVKEHGKTKISDVLLEQVYGGMRGIPGSVWEGSVLDPEDGIRFRGRTIADIQKDLPKAKGSSQPLPEALFWLLLTGEVPTQAQVENLSADLMSRSELPSHVVQLLDNLPKDLHPMAQFSIAVTALESESKFAKAYAQGISKQDYWSYTFEDSLDLLGKLPVIAAKIYRNVFKDGKMGEVDPNADYAKNLVNLIGSKDEDFVDLMRLYLTIHSDHEGGNVSAHTSHLVGSALSSPYLSLASGLNGLAGPLHGRANQEVLEWLFALKEEVNDDYSKDTIEKYLWDTLNSGRVIPGYGHAVLRKTDPRYMAQRKFAMDHFPDYELFKLVSSIYEVAPGVLTEHGKTKNPWPNVDAHSGVLLQYYGLKESSFYTVLFGVSRAFGILAQLITDRAIGASIERPKSYSTEKYKELVKNIESKL

>Schizosaccharomyces pombe (NP_593718.2)

MMIILNAPRFMTNTRLASTRRLASSLLSQASLRSRQLNPLFTSSYSTRSSSLKDRLAELIPEKQAEIKKFRAEHGQDVIGEVTINQMYGGARGVRSLIWEGSVLDPNEGIRFRGYTIPECQKLLPSSPNGKQPLPESLFWLLVTGEIPTLSQVQALSADWAARSQLPKFVEELIDRCPPTLHPMAQFSLAVTALEHDSAFAKAYERGMNKHDYWKYEYEDCMDLIAKTVPIAGRIYRNLYRDGVVAPIQMDKDHSYNFANVLGFANNEEFVELMRLYLTIHADHEGGNVSAHTGHLVGSALSSPFLSMAASLNGLAGPLHGLANQEVLNFLITMKKEIGDDLSEETIKSYLWKLLNSGRVVPGYGHAVLRKTDPRYTAQREFALEHLPKDPMFQLVSRLYEIVPGVLTEHGKTKNPYPNVDSHSGVLLQYYGLKEQSFYTVLFGVSRTLGVASQLIWDRALGLPIERPKSFSTEALKKMVETK

>Candida albicans (EEQ43448.1)

MSAFRSIQRSTNVAKSTFKNSIRTYASAEPTLKQRLEEILPAKAEEVKQFKKEHGKTVIGEVLLEQAYGGMRGIKGLVWEGSVLDPIEGIRFRGRTIPDIQKELPKAPGGEEPLPEALFWLLLTGEVPTDAQTKALSEEFAARSALPKHVEELIDRSPSHLHPMAQFSIAVTALESESQFAQAYAKGANKSEYWKYTYEDSIDLLAKLPTIAAKIYRNVFHDGKLPAAIDSKLDYGANLASLLGFGDNKEFVELMRLYLTIHSDHEGGNVSAHTTHLVGSALSSPFLSLAAGLNGLAGPLHGRANQEVLEWLFKLREELNGDYSKEAIEKYLWKTLNSGRVVPGYGHAVLRKTDPRYTAQREFALKHMPDYELFKLVSNIYEVAPGVLTKHGKTKNPWPNVDSHSGVLLQYYGLTEQSFYTVLFGVSRAFGVLPQLILDRGIGMPIERPKSFSTEKYIELVKNINKA

>Aspergillus nidulans (XP_681544.1)

MASTLRLSTSALRSSTLAGKPVVQSVAFNGLRCYSTGKTKSLKETFADKLPGELEKVKKLRKEHGNKVIGELTLDQAYGGARGVKCLVWEGSVLDSEEGIRFRGLTIPECQKLLPKAPGGEEPLPEGLFWLLLTGEVPSEQQVRDLSAEWAARSDLPKFIEELIDRCPSTLHPMAQFSLAVTALEHESAFAKAYAKGINKKEYWHYTFEDSMDLIAKLPTIAAKIYRNVFKDGKVAPIQKDKDYSYNLANQLGFADNKDFVELMRLYLTIHSDHEGGNVSAHTTHLVGSALSSPMLSLAAGLNGLAGPLHGLANQEVLNWLTEMKKVVGNDLSDQSIKDYLWSTLNAGRVVPGYGHAVLRKTDPRYTSQREFALRKLPDDPMFKLVSQVYKIAPGVLTEHGKTKNPYPNVDAHSGVLLQYYGLTEANYYTVLFGVSRALGVLPQLIIDRAFGAPIERPKSFSTEAYAKLVGAKL

MITOCHONDRIAL

>Arabidopsis thaliana m1 (NM_180084.2)

MVFFRSVSAFTRLRSRVQGQQSSLSNSVRWIQMQSSTDLDLKSQLQELIPEQQDRLKKLKSEHGKVQLGNITVDMVIGGMRGMTGLLWETSLLDPEEGIRFRGLSIPECQKVLPTAQSGAEPLPEGLLWLLLTGKVPSKEQVEALSKDLANRAAVPDYVYNAIDALPSTAHPMTQFASGVMALQVQSEFQKAYENGIHKSKFWEPTYEDCLNLIARVPVVAAYVYRRMYKNGDSIPSDKSLDYGANFSHMLGFDDEKVKELMRLYITIHSDHEGGNVSAHTGHLVGSALSDPYLSFAAALNGLAGPLHGLANQEVLLWIKSVVEECGEDISKEQLKEYVWKTLNSGKVIPGYGHGVLRNTDPRYVCQREFALKHLPDDPLFQLVSKLYEVVPPVLTELGKVKNPWPNVDAHSGVLLNHYGLTEARYYTVLFGVSRSLGICSQLIWDRALGLALERPKSVTMDWLEAHCKKASSA

>Arabidopsis thaliana m2 (NM_115873.3)

MVFFRSVSAISRLRSRAVQQSSLSNSVRWLHSSELDLKSQMQEIIPEQQDRLKKLKSEQGKVPVGNITVDMVLGGMRGMTGLLWETSLLDADEGIRFRGMSIPECQKILPSAESGEEPLPESLLWLLLTGKVPTKEQANALSTELAHRAAVPAIDALPSTAHPMTQFASGVMALQVQSEFQKAYEQGDISKSKYWEPTFEDALNLIARVPVVASYVYRRMYKDGSIIPLDDSLDYGANFSHMLGFDSPQMKELMRLYVTIHSDHEGGNVSAHAGHLVGSALSDPYLSFAAALNGLAGPLHGLANQEVLLWIKLVVEECGESISKEQLKDYVWKTLNSGKVVPGYGHGVLRKTDPRYICQREFALKHLPDDPLFQLVSKLYEVVPPILTELGKVKNPWPNVDAHSGVLLNYYGLTEARYYTVLFGVSRSLGICSQLIWDRALGLPLERPKSVNMDWLDNFTRLNR

>Arabidopsis lyrata (XP_002880107.1)

MVFFRSVSAFTRLRSRVQGQQSSLSNSVRWIQMQSSTDLDLKSQLQELIPEQQDRLKKLKSEHGKVQLGNITVDMVIGGMRGMTGLLWETSLLDPEEGIRFRGLSIPECQKVLPTAQSGAEPLPEGLLWLLLTGKVPSKEQVEALSKDLASRAAVPDYVYNAIDALPSTAHPMTQFASGVMALQVQSEFQKAYENGIHKSKFWEPTYEDCLNLIARVPVVAAYVYRRMYKNGDSIPSDKSLDYGANFSHMLGFDDEKMKELMRLYITIHSDHEGGNVSAHTGHLVGSALSDPYLSFAAALNGLAGPLHGLANQEVLLWIKQVVEECGEDISKEQLKEYVWKTLNSGKVVPGYGHGVLRNTDPRYVCQREFALKHLPDDPLFQLVSKLYEVVPPVLTQLGKVKNPWPNVDAHSGVLLNHYGLTEARYYTVLFGVSRSLGICSQLIWDRALGLALERPKSVTMDWLEAHCKKASSA

>Solanum lycopersicum (XP_004229342.1)

MVFYRSVSLLSKLRSRAVQQSNVSNSVRWLQVQTSSGLDLRSELQELIPEQQDRLKKIKSEYGKVQLGNITVDMVLGGMRGMTGLLWETSLLDPDEGIRFRGLSIPECQKVLPAAKPGGEPLPEGLLWLLLTGKVPSKEQVNSLSQELRSRATIPDHVYKTIDALPVTAHPMTQFATGVMALQVQSEFQKAYEKGIHKSKFWEPTYEDSMNLIAQVPLVAAYVYRRMYKNGDTIPKDESLDYGANFAHMLGFSSSEMHELMRLYVTIHSDHEGGNVSAHTGHLVASALSDPYLSFAAALNGLAGPLHGLANQEVLLWIKSVVEECGENISKEQLKDYVWKTLNSGKVVPGFGHGVLRKTDPRYTCQREFAMKHLPKDPLFQLVSKLYEVVPPVLTELGKVKNPWPNVDAHSGVLLNYYGLTEARYYTVLFGVSRALGICSQLIWDRALGLPLERPKSITMEWLEKQCKKA

>Citrus maxima (ADZ05826.1)

MAFFRSVTALSRLRSRVGQQSNLSNSVRWLQMQSSADLDLHSQLKEMIPEQQERLKKVKSELGKAQLGNITIDMVIGGMRGMTGLLWETSLLDPDEGIRFRGLSIPECQKLLPAAKPDGEPLPEGLLWLLLTGKVPSKEQVDGLSKELRDRATVPDYVYKAIDALPVTAHPMTQFASGVMALQVQSEFQEAYEKGIHKSKYWEPTYEDSLNLIARVPVVAAYVYQRIYKDGKIIPKDDSLDYGGNFSHMLGFDDPKMLELMRLYVTIHSDHEGGNVSAHTGHLVASALSDPYLSFAAALNGLAGPLHGLANQEVLLWIKSVVDECGENVTTEQLKDYVWKTLNSGKVVPGFGHGVLRKTDPRYTCQREFALKHLPDDPLFQLVSKLFEVVPPILTKLGKVKNPWPNVDAHSGVLLNHFGLAEARYYTVLFGVSRSLGICSQLIWDRALGLPLERPKSVTLDWIEKNCKKAA

>Cucumis sativus (XP_004135902.1)

MAFFKSLTALSKLRSRVGQQSNLSNSVRWLQMQSSSDLDLQSHLRELIPEQQDRLKKFKAEHGKVQLGNITVDMVLGGMRGMTGLLWETSLLDPDEGIRFRGLSIPECQKLLPAAKPDGEPLPEGLLWLLLTGKVPSKEQVDALSRELQSRATVPDYVYKAIDALPITSHPMTQFATGVMGLQVQSEFQKAYEKGIHKSKYWEPTYEDSLNLIAQVPLVASYVYRRIYKDGHIIPKDDSLDYGGNFSHMLGFDSPQMQELMRLYVTIHSDHEGGNVSAHTGHLVASALSDPYLSFAAALNGLAGPLHGLANQEVLLWIKSVVEECGENITKDQLKDYVWKTLNSGKVVPGFGHGVLRKTDPRYTCQREFALKHLPDDPLFQLVSKLYEVVPPILTELGKVKNPWPNVDAHSGVLLNYFGLTEARYFTVLFGVSRSLGICSQLIWDRALGLPLERPKSVTMQWLEDYCKKAT

>Citrus sinensis (ACU42176.1)

MAFFRSVTALSRLRSRVGQQSNLSNSVRWLQMQSSSDLDLHSQLKEMIPEQQERLKKVKSELGKAQLGNITVDMVIGGMRGMTGLLWETSLLDPDEGIRFRGLSIPECQKLLPAAKPDGEPLPEGLLWLLLTGKVPSKEQVDGLSKELRDRATVPDYVYKAIGALPVTAHPMTQFASGVMALQVQSEFQEAYEKGIHKSKYWEPTYEDSLNLIARVPVVAAYVYQRIYKDGKIIPKDDSLDYGGNFSHMLGFDDPKMLELMRLYVTIHSDHEGGNVSAHTGHLVASALSDPYLSFAAALNGLAGPLHGLANQEVLLWIKSVVDECGENVTTEQLKDYVWKTLNSGKVVPGFGHGVLRKTDPRYTCQREFALKHLPDDPLFQLVSKLFEVVPPILTKLGKVKNPWPNVDAHSGVLLNHFGLAEARYYTVLFGVSRSLGICSQLIWDRALGLPLERPKSVTLDWIEKNCKKAA

>Vitis vinifera (XP_002271451.1)

MVFFRSVSALSKLRSRLAQPSSLNGSVRWLQVQTCSDLDLHSQLKELIPEQQERLKKLKAECGKIQLGNITVDMVLGGMRGMTGLLWETSLLDPDEGIRFRGMSIPECQKVLPNATPGGEPLPEGLLWLLLTGKVPTKGQVDALSKELSTRATVPDHVYKAIDALPQSAHPMTQFATGVMALQVQSEFQKAYEKGIPKSKYWEPTYEDSLSLIAQVPVVAAYVYRRVYKNGQIIPKDDSLDYGANFSHMLGFDSPKMQELMRLYVTIHSDHEGGNVSAHTGHLVASALADPYLSFAAALNGLAGPLHGLANQEVLLWIKSVVDECGENISTEQLKDYVWKTLKSGKVVPGFGHGVLRKTDPRYMCQREFALKHLPDDPLFQLVSKLYEVVPPILTELGKVKNPWPNVDAHSGVLLNHFGLHEARYFTVLFGVSRSIGICSQLIWDRALGLPLERPKSVTMEWLENHCKKAAA

>Populus trichocarpa (XP_002330895.1)

MVFFRSVPLLSRLRSHAQHQVQKQSNLSNSFRWIQTQTISSDLDLHSQLKELIPEQQERLKKLKSEYGKVQLGNITVDMVLGGMRGMTGLLWETSLLDPDEGIRFRGMSIPECQKLLPAAKPGGEPLPEGLLWLLLTGKVPTKEQVGALSKELRDRASVPDYVFKAIDALPVTAHPMTQFATGVMALQVQSEFQKAYEKGIHKSKYWEPTYEDSLSLIARVPIVASYIYRRIYKDGKVIPMNDSLDYGGNFSHMLGFDSPEMQELMRLYVTIHSDHEGGNVSAHTGHLVASALSDPYLSFAAALNGLAGPLHGLANQEVLLWIKSVVEECGENITTEQLKDYVWKTLNSGKVVPGFGHGVLRKTDPRYTCQREFALKHLPDDPLFQLVSKLYEVVPPVLTQLGKVKNPWPNVDAHSGVLLNYYGLTEARYYTVLFGVSRSIGICSQLIWDRALGLPLERPKSVTMELLENHCKKAPAN

>Fragaria vesca mt1 (XP_004306486.1)

MAFFRTVTKLRSRLGQPSSLRDSVRCLQTQASSDLDLHSQLKELIPEQQERLKKLKKEHGKVQLGTITVDMVIGGMRGMTGLLWETSLLDPDEGIRFRGLSIPECQKVLPGATPGGEPLPEGLLWLLLTGKVPSKEQVDALSSELRSRAKVPDYVFKAIDALPVGAHPMTQFTTGVMALQVQSEFQKAYDKGIPKSRYWEPTYEDSLSLIAQLPVVASYVYRRIYKGGRMIPVDDSLDYGGNFSHLLGFDDHKMQELMRLYVTIHSDHEGGNVSAHTGHLVASALSDPFLSFAAALNGLAGPLHGLANQEVLLWIKSVVDECGENITKDQLKDYVWKTLNSGKVVPGFGHGVLRKTDPRYTCQREFALKHLPDDPLFRLVSKLYDVVPPILTELGKVKNPWPNVDAHSGVLLNHFGLTEASIYFTVLFGVSRSIGIGSQLIWDRALGLPLERPKSVTMESLESFCKKAAS

>Fragaria vesca mt2 (XP_004306487.1)

MAFFRTVTKLRSRLGQPSSLRDSVRCLQTQASSDLDLHSQLKELIPEQQERLKKLKKEHGKVQLGTITVDMVIGGMRGMTGLLWETSLLDPDEGIRFRGLSIPECQKVLPGATPGGEPLPEGLLWLLLTGKVPSKEQVDALSSELRSRAKVPDYVFKAIDALPVGAHPMTQFTTGVMALQVQSEFQKAYDKGIPKSRYWEPTYEDSLSLIAQLPVVASYVYRRIYKGGRMIPVDDSLDYGGNFSHLLGFDDHKMQELMRLYVTIHSDHEGGNVSAHTGHLVASALSDPFLSFAAALNGLAGPLHGLANQEVLLWIKSVVDECGENITKDQLKDYVWKTLNSGKVVPGFGHGVLRKTDPRYTCQREFALKHLPDDPLFRLVSKLYDVVPPILTELGKVKNPWPNVDAHSGVLLNHFGLTEARYFTVLFGVSRSIGIGSQLIWDRALGLPLERPKSVTMESLESFCKKAAS

>Oryza sativa (AAG28777.1)

MAFFRGLTAVSRLRSRVAQEATTLGGVRWLQMQSASDLDLKSQLQELIPEQQDRLKKLKSEHGKVQLGNITVDMVLGGMRGMTGMLWETSLLDPDEGIRFRGLSIPECQKVLPTAVKDGEPLPEGLLWLLLTGKVPTKEQVDALSKELASRSSVPGHVYKAIDALPVTAHPMTQFTTGVMALQVESEFQKAYDKGMSKSKFWEPTYEDCLNLIARLPAVASYVYRRIFKGGKTIAADNALDYAANFSHMLGFDDPKMLELMRLYITIHTDHEGGNVSAHTGHLVGSALSDPYLSFAAALNGLAGPLHGLANQEVLLWIKSVIGETGSDVTTDQLKEYVWKTLKSGKVVPGFGHGVLRKTDPRYTCQREFALKYLPEDPLFQLVSKLYEVVPPILTELGKVKNPWPNVDAHSGVLLNHFGLSEARYYTVLFGVSRSIGIGSQLIWDRALGLPLERPKSVTMEWLENHCKKVAA

>Brachypodium distachyon (XP_003571200.1)

MAFFRGLAAVSRLRSRMGQDATTLGGVRWLQMQSASDLDLRSQLQEMIPEQQDRLKKLKSEHGKVQLGNITVDMVLGGMRGMTGMLWETSLLDPEEGIRFRGLSIPECQKVLPAAVKDGEPLPEGLLWLLLTGKVPTKEQVDALSKELLARSNVPDYVYKAIDALPVTAHPMTQFTTGVMALQVDSEFQKAYDKGMPKTKFWEPTYEDCLNLIARLPQVASYVYRRIFKDGKIISADNTLDYAANFSHMLGFDDPKMLELMRLYITIHTDHEGGNVSAHTGHLVGSALSDPYLSFAAALNGLAGPLHGLANQEVLLWIKTVMEETGSNITTDQLKEYVWKTLKSGKVVPGYGHGVLRNTDPRYSCQREFALKYLPEDPLFQLVSKLYEVVPPILTELGKVKNPWPNVDAHSGVLLNHFGLSEARYYTVLFGVSRSIGIGSQLIWDRALGLPLERPKSVTMEWLENYCKKAAA

>Sorghum bicolor (XP_002453470.1)

MAFFRGLTAVSRLRSRMAQEATTLGGVRWLQMQSASDLDLKSQMQELIPEQQPILANSVNPFSFSSRISVSDIFSSIHLNMRWNMKAIACLKVASTDVSLSGYRIALKKLKSEHGKVQLGNITVDMVLGGMRGMTGMLWETSLLDPEEGIRFRGLSIPECQKVLPTAVKGGEPLPEGLLWLLLTGKVPTKEQVDALSKELLARSTVPAHVYKAIDALPVTAHPMTQFTTGVMALQVESEFQKAYDNGLPKSKFWEPTYEDCLNLIARLPPVASYVYRRIFKGGKSIEADNSLDYAANFSHMLGFDDPKMLELMRLYVTIHTDHEGGNVSAHTGHLVGSALSDPYLSFAAALNGLAGPLHGLANQEVLLWIKSVIEETGSDVTTDQLKDYVWKTLKSGKVVPGFGHGVLRKTDPRYSCQREFALKHLPEDPLFQLVSKLYEVVPPILTELGKVKNPWPNVDAHSGVLLNHFGLSEARYYTVLFGVSRSMGIGSQLIWDRALGLPLERPKSVTMEWLENYCKNKAA

>Coccomyxa subellipsoidea (EIE25676.1)

MRGLRALRGLASLTSSTLIECAEATTRTLSTASDLKSVLKEKIPEQQERLKAIKKTHGSKELGKVTVDMAIGGMRGITGLLWETSLLDPEEGIRFRGYSIPELQEKLPAAKEGGEPLPEGLLWLLLTGEIPSKEQVDALSAELSYRGKALPGHVHKVLAALPAGTHPMTQFSTAVLALQPESHFAAAYQKGISKLNYWDPVYEDSMNLIAKLPGIAAAIYRNTYKGGKLIPHDTSLDWAANLSHQMGFDGEEVYELTRLYQTIHTDHEGGNVSAHATHLVGSALSDPYFSFSAGLNGLAGPLHGLANQEVLRWLFDVTKQIGPTPTKDEVEKFVWDTLKSGKVVPGFGHAVLRKTDPRYTCQREFAQKHLPDDPMFKLVSLFYEVVPGVLTEQGKVKNPWPNVDAHSGVLLQYYGIKEENFYTVLFGVSRAIGVLSQGIWSRALGLAIERPKSVTMDVLEKKFA

>Chlamydomonas reinhardtii (XP_001702983.1)

MLATAASKLGLTGLGIQAISAVGNSIRQFSSTENTDLKQALRELIPAQQERLKALKKGHGSKSLGEVTVDMAIGGMRGIPGMLWETSLLDPEEGIRFRGLSIPELQEKLPAAVPGGQPIPEGLLWLLLTGQVPTSSQAAAVTEELRARSNVPLHLRKVLDALPTEAHPMTQLSMGIMALQSGSRFQSAYARGVHKSRYWEYAYEDSMDLIAKLPQLAATIYRRTYRRGEYIPPSYNLDWAANLAHMMGYEDPGCLEMMRMYQTIHSDHEGGNVSAHATHLVGSALSDPYLSFAAGMNGLAGPLHGLANQEVLRWLKDLVAKLGPSPGRDAVRKYVEDTLASGKVVPGYGHAVLRKTDPRYTCQREFALKYMPDYPLFKLVADLYEVVPEVLGKTGKIKNPWPNVDAHSGVLLQYYGITEENYYTVLFGVSRALGVLSQGVWSRALGLPIERPKSLTMAALEQRVAGQSA

>Volvox carteri (XP_002948056.1)

MLAQAVGRLGLGLGAHSLPAVGNSLRRFCTDGVDDLKKTLSELIPEQQERLKALKKSHGSKSLGEVTVDMAIGGMRGIPGMLWETSLLDPEEGIRFRGLSIPELQAQLPAAIPGGQPIPEGLLWLLLTGKVPTKEQAASVTEELRERSHLGPHVLKVLNALPDDAHPMTQLSVGVMALQSGSHFAAAYTRGTHKSKYWETVYEDSMDLIAKLPQLAAIIYRRTYKDGSQIQPHHTLDWAANLAYMMGYEDVGCHEMMRMYQTIHSDHEGGNVSAHTTHLVGSALSDPYLSFAAGMNGLAGPLHGLANQEVLRWLKNLTAKLGPHPEKEAVRKYVEETLASGKVVPGYGHAVLRKTDPRYTCQREFAQRYMPEYPMFKLVSDLYEVVPEVLGKTGKIKNPWPNVDAHSGVLLQYYGITEENFYTVLFGVSRALGVLAQGIWSRALGLPIERPKSMTMGVLEKRFSAAPSSQ

>Caenorhabditis elegans (NP_499264.1)

MSLSGMAIRRLITKGVIPVCQVAPLSTSAEGSTNLKEVLSKKIPAHNAKVKSFRTEHGSTVVQNVNIDMIYGGMRSMKGMVTETSVLDPEEGIRFRGYSIPECQKLLPKAKGGEEPLPEAIWWLLCTGDVPSEAQTAAITKEWNARADLPTHVVRMLDNFPDNLHPMAQFIAAIAALNNESKFAGAYARGVAKASYWEYAYEDSMDLLAKLPTVAAIIYRNLYRDGSAVSVIDPKKDWSANFSSMLGYDDPLFAELMRLYLVIHSDHEGGNVSAHTSHLVGSALSDPYLSFSAAMAGLAGPLHGLANQEVLVFLNKIVGEIGFNYTEEQLKEWVWKHLKSGQVVPGYGHAVLRKTDPRYECQREFALKHLPNDDLFKLVSTLYKITPGILLEQGKAKNPWPNVDSHSGVLLQYFGMTEMSFYTVLFGVSRALGCLSQLIWARGMGLPLERPKSHSTDGLIKLALAAKK

>Homo sapiens (NM_004077.2)

MALLTAAARLLGTKNASCLVLAARHASASSTNLKDILADLIPKEQARIKTFRQQHGKTVVGQITVDMMYGGMRGMKGLVYETSVLDPDEGIRFRGFSIPECQKLLPKAKGGEEPLPEGLFWLLVTGHIPTEEQVSWLSKEWAKRAALPSHVVTMLDNFPTNLHPMSQLSAAVTALNSESNFARAYAQGISRTKYWELIYEDSMDLIAKLPCVAAKIYRNLYREGSGIGAIDSNLDWSHNFTNMLGYTDHQFTELTRLYLTIHSDHEGGNVSAHTSHLVGSALSDPYLSFAAAMNGLAGPLHGLANQEVLVWLTQLQKEVGKDVSDEKLRDYIWNTLNSGRVVPGYGHAVLRKTDPRYTCQREFALKHLPNDPMFKLVAQLYKIVPNVLLEQGKAKNPWPNVDAHSGVLLQYYGMTEMNYYTVLFGVSRALGVLAQLIWSRALGFPLERPKSMSTEGLMKFVDSKSG

>Canis lupus (XP_531634.2)

MALLTAAARLFGAKNASCLVLAARHASASSTNLKDILADLIPKEQARIKTFRQQHGKTVVGQITVDMMYGGMRGMKGLVYETSVLDPDEGIRFRGYSIPECQKLLPKAKGGEEPLPEGLFWLLVTGQIPTEEQVSWLSKEWAKRAALPSHVVTMLDNFPTNLHPMSQLSAAITALNSESNFARAYAEGINRTKYWELIYEDCMDLIAKLPCVAAKIYRNLYREGSSIGAIDSKLDWSHNFTNMLGYTDAQFTELMRLYLTIHSDHEGGNVSAHTSHLVGSALSDPYLSFAAAMNGLAGPLHGLANQEVLVWLTQLQKEVGKDVSDEKLRDYIWNTLNSGRVVPGYGHAVLRKTDPRYTCQREFALKHLPHDSMFKLVAQLYKIVPNVLLEQGKAKNPWPNVDAHSGVLLQYYGMTEMNYYTVLFGVSRALGVLAQLIWSRALGFPLERPKSMSTDGLMKFVDSKSG

>Felis catus (XP_003988937.1)

MALLTAAARLFGAKNASCLVLAARHASASPTNLKDILADLIPKEQARVKTFRQQHGKTVVGQITVDMMYGGMRGMKGLVYETSVLDPDEGIRFRGYSIPECQKLLPKAKGGEEPLPEGLFWLLVTGQIPTEEQVSWLSKEWAKRAALPSHVVTMLDNFPTNLHPMSQLSAAITALNSESNFARAYAEGINRTKYWELIYEDCMDLIAKLPCVAAKIYRNLYREGSSIGAIDSKLDWSHNFTNMLGYTDAQFTELMRLYLTIHSDHEGGNVSAHTSHLVGSALSDPYLSFAAAMNGLAGPLHGLANQEVLVWLTQLQKEVGKDVSDEKLRDYIWNTLNSGRVVPGYGHAVLRKTDPRYTCQREFALKHLPHDSMFKLVAQLYKIVPNVLLEQGKAKNPWPNVDAHSGVLLQYYGMTEMNYYTVLFGVSRALGVLAQLIWSRALGFPLERPKSMSTDGLMKFVDSKSG

>Rattus norvegicus (EDL84868.1)

MALLTAAARLLGAKNSSCLVLAARHASASSTNLKDILSNLIPKEQARVKTFRQQHGKTVVGQITVDMMYGGMRGMKGLVYETSVLDPDEGIRFRGYSIPECQKLLPKAKGGEEPLPEGLFWLLVTGQMPTEEQVSWLSQEWAKRAALPSHVVTMLDNFPTNLHPMSQLSAAITALNSESNFARAYAEGINRTKYWELIYEDCMDLIAKLPCVAAKIYRNLYREGSSIGAIDSKLDWSHNFTNMLGYTEPQFTELMRLYLTIHSDHEGGNVSAHTSHLVGSALSDPYLSFAAAMNGLAGPLHGLANQEVLVWLTQLQKEVGKDVSDEKLRDYIWNTLNSGRVVPGYGHAVLRKTDPRYSCQREFALKHLPKDPMFKLVAQLYKIVPNILLEQGKAKNPWPNVDAHSGVLLQYYGMTEMNYYTVLFGVSRALGVLAQLIWSRALGFPLERPKSMSTDGLMKFVDSKSG

>Saccharomyces cerevisiae (NP_014398.1)

MSAILSTTSKSFLSRGSTRQCQNMQKALFALLNARHYSSASEQTLKERFAEIIPAKAEEIKKFKKEHGKTVIGEVLLEQAYGGMRGIKGLVWEGSVLDPEEGIRFRGRTIPEIQRELPKAEGSTEPLPEALFWLLLTGEIPTDAQVKALSADLAARSEIPEHVIQLLDSLPKDLHPMAQFSIAVTALESESKFAKAYAQGVSKKEYWSYTFEDSLDLLGKLPVIASKIYRNVFKDGKITSTDPNADYGKNLAQLLGYENKDFIDLMRLYLTIHSDHEGGNVSAHTTHLVGSALSSPYLSLAAGLNGLAGPLHGRANQEVLEWLFKLREEVKGDYSKETIEKYLWDTLNAGRVVPGYGHAVLRKTDPRYTAQREFALKHFPDYELFKLVSTIYEVAPGVLTKHGKTKNPWPNVDSHSGVLLQYYGLTEASFYTVLFGVARAIGVLPQLIIDRAVGAPIERPKSFSTEKYKELVKKIESKN

>Schizosaccharomyces pombe (NP_593718.3)

MMIILNAPRFMTNTRLASTRRLASSLLSQASLRSRQLNPLFTSSYSTRSSSLKDRLAELIPEKQAEIKKFRAEHGQDVIGEVTINQMYGGARGVRSLIWEGSVLDPNEGIRFRGYTIPECQKLLPSSPNGKQPLPESLFWLLVTGEIPTLSQVQALSADWAARSQLPKFVEELIDRCPPTLHPMAQFSLAVTALEHDSAFAKAYERGMNKHDYWKYEYEDCMDLIAKTVPIAGRIYRNLYRDGVVAPIQMDKDHSYNFANVLGFANNEEFVELMRLYLTIHADHEGGNVSAHTGHLVGSALSSPFLSMAASLNGLAGPLHGLANQEVLNFLITMKKEIGDDLSEETIKSYLWKLLNSGRVVPGYGHAVLRKTDPRYTAQREFALEHLPKDPMFQLVSRLYEIVPGVLTEHGKTKNPYPNVDSHSGVLLQYYGLKEQSFYTVLFGVSRTLGVASQLIWDRALGLPIERPKSFSTEALKKMVETK

>Candida albicans (XP_715169.1)

MRGIKGLVWEGSVLDPIEGIRFRGRTIPDIQKELPKAPGGEEPLPEALFWLLLTGEVPTDAQTKALSEEFAARSALPKHVEELIDRSPSHLHPMAQFSIAVTALESESQFAQAYAKGANKSEYWKYTYEDSIDLLAKLPTIAAKIYRNVFHDGKLPAAIDSKLDYGANLASLLGFGDNKEFVELMRLYLTIHSDHEGGNVSAHTTHLVGSALSSPFLSLAAGLNGLAGPLHGRANQEVLEWLFKLREELNGDYSKEAIEKYLWETLNSGRVVPGYGHAVLRKTDPRYTAQREFALKHMPDYELFKLVSNIYEVAPGVLTKHGKTKNPWPNVDSHSGVLLQYYGLTEQSFYTVLFGVSRAFGVLPQLILDRGIGMPIERPKSFSTEKYIELVKNINKA

>Aspergillus nidulans (XP_68154.1)

MASTLRLSTSALRSSTLAGKPVVQSVAFNGLRCYSTGKTKSLKETFADKLPGELEKVKKLRKEHGNKVIGELTLDQAYGGARGVKCLVWEGSVLDSEEGIRFRGLTIPECQKLLPKAPGGEEPLPEGLFWLLLTGEVPSEQQVRDLSAEWAARSDLPKFIEELIDRCPSTLHPMAQFSLAVTALEHESAFAKAYAKGINKKEYWHYTFEDSMDLIAKLPTIAAKIYRNVFKDGKVAPIQKDKDYSYNLANQLGFADNKDFVELMRLYLTIHSDHEGGNVSAHTTHLVGSALSSPMLSLAAGLNGLAGPLHGLANQEVLNWLTEMKKVVGNDLSDQSIKDYLWSTLNAGRVVPGYGHAVLRKTDPRYTSQREFALRKLPDDPMFKLVSQVYKIAPGVLTEHGKTKNPYPNVDAHSGVLLQYYGLTEANYYTVLFGVSRALGVLPQLIIDRAFGAPIERPKSFSTEAYAKLVGAKL

>Escherichia coli (AAC73814.1)

MADTKAKLTLNGDTAVELDVLKGTLGQDVIDIRTLGSKGVFTFDPGFTSTASCESKITFIDGDEGILLHRGFPIDQLATDSNYLEVCYILLNGEKPTQEQYDEFKTTVTRHTMIHEQITRLFHAFRRDSHPMAVMCGITGALAAFYHDSLDVNNPRHREIAAFRLLSKMPTMAAMCYKYSIGQPFVYPRNDLSYAGNFLNMMFSTPCEPYEVNPILERAMDRILILHADHEQNASTSTVRTAGSSGANPFACIAAGIASLWGPAHGGANEAALKMLEEISSVKHIPEFVRRAKDKNDSFRLMGFGHRVYKNYDPRATVMRETCHEVLKELGTKDDLLEVAMELENIALNDPYFIEKKLYPNVDFYSGIILKAMGIPSSMFTVIFAMARTVGWIAHWSEMHSDGMKIARPRQLYTGYEKRDFKSDIKR

>Candidatus thessalonicensis (WP_010302460.1)

MTLSTDTHASMATVSYKGKTIELPVLQGSCGPDVIDIRNLYSQLGVFTFDPGFTATASCESKITFIDGDEGILLYGGYPIQDLAEKSDFMEVSYLLLYGDLPTAAQKKDFVKNITLHTMVHEQIPTFYRGFRRDAHPMAIMVGVVGALSAFYHDSLDIHDPRQREIAAYRMVAKVPTLAAMAYKYSIGQPFVYPRNDLDYASNFLHMMFSVPTEPYEVNPVLSSAMDKILILHADHEQNASTSTVRLAGSSAANPFACMAAGVAALWGPAHGGANEAALNMLREIRDPSRVKEFVNRAKDKNDPFRLMGFGHRVYKNYDPRAKVMRETCHKVLKELNIHNDPLLEVAMELEQIALSDPYFIEKKLYPNVDFYSGIIFKAMGIPTSMFTVIFALARTVGWVAQWKEMICDPLQKIGRPRQLYTGVIERQYVDMSQRR

>Mesorhizobium alhagi (WP_008837678.1)

MTESTAKLEFGGKALELKARSGSVGPDVIDISTLYRDTGAFTYDPGFTSTASCESEITFIDGDEGVLLHRGYPIDQLAEHSDFLEVCYLLLYGELPTKAQKDDFDYRVTRHTMVHEQMSRFFTGFRRDAHPMAVMCGVVGALSAFYHDSTDISDPHQRMVASIRLIAKMPTIAAMAYKYHIGQPFVYPKNELNFAANFLHMCFAVPCEEYKVNPVLARAMERIFILHADHEQNASTSTVRLAGSSGANPFACIAAGIACLWGPAHGGANEAALNMLSEIGHVDRIPEFIARAKDKNDPFRLMGFGHRVYKNYDPRAKIMHKTAHEVLGELGIKDDPMLDVAMELERIALTDEYFIEKKLYPNVDFYSGITLKALGFPTTMFTVLFAVARTVGWISQWKEMIEDPHQKIGRPRQLYTGHPQRDFIAIAKR

>Hoeflea phototrophica (WP_007197207.1)

MADRNAKLTLDGNEVDFAVKTGTIGPDVIDIAALYKHTGAFTYDPGFTSTASCESKITFIDGDEGMLLHRGYPIDQLAEHGDFLEVCYLLLYGELPTATQKSDFDYRVTRHTMVHEQMSRFFTGFRRDAHPMAVMCGCVGALSAFYHDSTDITDPHQRMVASMRMIAKMPTLAAMAYKYHVGQPFVYPKNDLDYASNFLRMCFAVPCEEYVINPVLSRAMDRIFILHADHEQNASTSTVRLAGSSGANPFACIAAGIACLWGPAHGGANEAALNMLGEIGHVDNIPEYVARAKDKNDPFRLMGFGHRVYKNYDPRAKIMQKTTHEVLGELGIKDDPLLEVAMELERIALTDSYFIEKKLYPNIDFYSGITLKALGFPTTMFTVLFALARTVGWIAQWSEMIEDPSQRIGRPRQLYTGEAARDYIPVSKR

>Synechocystis (NP_442192.1)

MNYMMTDNEVFKEGLAGVPAAKSRVSHVDGTDGILEYRGIRIEELAKSSSFIEVAYLLIWGKLPTQAEIEEFEYEIRTHRRIKYHIRDMMKCFPETGHPMDALQTSAAALGLFYARRALDDPKYIRAAVVRLLAKIPTMVAAFHMIREGNDPIQPNDKLDYASNFLYMLTEKEPDPFAAKVFDVCLTLHAEHTMNASTFSARVTASTLTDPYAVVASAVGTLAGPLHGGANEEVLNMLEEIGSVENVRPYVEKCLANKQRIMGFGHRVYKVKDPRAIILQDLAEQLFAKMGHDEYYEIAVELEKVVEEYVGQKGIYPNVDFYSGLVYRKLDIPADLFTPLFAIARVAGWLAHWKEQLSVNKIYRPTQIYIGDHNLSYVPMTERVVSVARNEDPNAII

>Cyanothece (YP_002371593.1)

MNVCEYIPGLENIPAAKSSISYVDGQEGILEYRGIRIEELATKGSFVETAYLLIWGELPTQEELDAFEGEIRYHRRIKYRIRDMMKCFPETGHPMDALQTSAAALGLFYARRALDNPDYIRQAVVRLLAKIPTMVAAAHQMRRGNDPIQPNDNLDYAANFLYMMTEQKPDPLAAKIFDVCLTLHAEHTINASTFSAMVTASTLTDPYAVVASAVGTLAGPLHGGANEEVLAMLEEIGSVENVRPYIEKLVANKQKIMGFGHRVYKVKDPRATILQNLAEQLFEKTGRDEYYAIAQEVEKVVEEKLGHKGIYANVDFYSGLVYRKLGIPSDLFTPLFAIARVAGWLAHWKEQLAVNRIFRPTQVYIGERNQPYVPMEKRLMVNRNGL

>Microcystis aeruginosa (ZP_16392266.1)

MTVCEYRPGLEGIPAAQSSISFVDGQKGILEYRGIRIEELAEKSTFLETAYLLIWGVLPTGAELEEFADEIRSHRRIKYRIEDMMKCFPEKGHPMDALQTSAAALGLYYARRALDNPEYIRQAVVRLLAKIPTMVAAFQLIRKGNHPIQPNDSLDYAANFLYMLTEKRPTDLAAKVFDVCLTLHAEHTMNASTFSAMVTASTLTDPYGVIASAVGTLAGPLHGGANEEVLLMLEEIGSVANVRPYVEKCIANKQKVMGFGHRVYKVKDPRATILQNLAEQLFAELGSDEYYDIALELEQAMVDIYGEKGIYPNVDFYSGLVYRKLGIPNDLFTPIFAISRVAGWLAHWKEQLNTNRIFRPTQIYTGEHEVPYTPIEQRS

>Nostoc punctiforme (YP_001868873.1)

MMVCEYKPGLEGIPAAQSSISYVDGQKGILEYRGIRIEELAEKSTFLETAYLLIWGELPSKEELEGFEHEVRYHRRIKYRIRDMMKCFPESGHPMDALQASAAALGLFYSLRDLHNPAYIRDSVVRLIATIPTMVAAFQLMRKGNDPVRPRDDLDYSSNFLYMLDEKEPDPLMARIFDICLILQVEHTMNASTFSARVTASTLTDPYAVVASAVGTLGGPLHGGANEEVIQMLEKIGSVENVRPYIEHCLETKSKIMGFGHRVYKVKDPRAIILQGLAEKLFEKFGYDKFYDIAQEMERVVGEKLGSKGIYPNIDFYSGLVYRKMGIPTDLFTPIFAIARVAGWLAHWKEQLAENRIFRPTQVYNGLHEVTYTPIDQR

>Oscillatoria acuminata (YP_007086506.1)

MTVADELKTGFEFKPGLEGVPVTLSSISFVDGKKGVLEYRNIPIEELALKSTFLETSYLLIWGELPTQDELDAFEKEIRYHRRIKYRIRDMMKCFPESGHPMDALQASAAALGLFYSRRALDDPAYIRAAAVRLLAKIPTMVAAFQMMRKGNDPIQPRDDLDYSANFLYMLTEQEPDPLEAQVLDVCLTLHAEHTINASTFSARVTASTLTDPYAVVASAVGTLAGPLHGGANEEVIDMLESIGSVENVRPYIENCLKTKSKIMGFGHRVYKVKDPRAIILQSLAEQLFEKFGRDNYYDIAVELERVVEEKLAYKGIYANVDFYSGLVYRKLGIPTDLFTPIFAISRVAGWLAHWKEQLGENRIYRPTQIYTGTHNAPYIPIAER

>Trichodesmium erythraeum (YP_721333.1)

MSVVCIEFKPGLEGVPATQSSVSYVDGQKGILEYRGINIEELAKKSSFLEAAYLLIWGNLPTQEELQSFEQEICLNRRIKYRIRDMMKCFPETGHPMDALQTSAAALGLFYSRRALDNPEYIRKAVVRLLANIPTMVAAFELMRKGNDPVKPRDDLGYAANFLYMLNEREPDPLAAKVFDICLTLHAEHTINASTFSAMVTASTLTDPYGVIASAVGTLAGPLHGGANEDVISMLSEIGSVENVKPYLKRCFETKAKIAGFGHRVYKVKDPRAIILQELAVDLFEKFGDDKYYQIALEMEKVVEEKLGHKGIYPNVDFYSGLVYRKLGIPSDLFTPVFAIARVAGWLAHWKEQLAANRIFRPTQIYSGDHNIAYTSLEERALI

________________________________________________________________________________

ACONITASE

CYTOSOLIC

>Arabidopsis thaliana (NP_195308.1)

MASENPFRSILKALEKPDGGEFGNYYSLPALNDPRIDKLPYSIRILLESAIRNCDEFQVKSKDVEKILDWENTSPKQVEIPFKPARVLLQDFTGVPAVVDLACMRDAMNNLGGDSNKINPLVPVDLVIDHSVQVDVARSENAVQANMELEFQRNKERFAFLKWGSNAFHNMLVVPPGSGIVHQVNLEYLARVVFNTNGLLYPDSVVGTDSHTTMIDGLGVAGWGVGGIEAEATMLGQPMSMVLPGVVGFKLTGKLRDGMTATDLVLTVTQMLRKHGVVGKFVEFHGEGMRELSLADRATIANMSPEYGATMGFFPVDHVTLQYLRLTGRSDDTVSMIEAYLRANKMFVDYSEPESKTVYSSCLELNLEDVEPCVSGPKRPHDRVPLKEMKADWHSCLDNRVGFKGFAVPKEAQSKAVEFNFNGTTAQLRHGDVVIAAITSCTNTSNPSVMLGAALVAKKACDLGLEVKPWIKTSLAPGSGVVTKYLAKSGLQKYLNQLGFSIVGYGCTTCIGNSGDIHEAVASAIVDNDLVASAVLSGNRNFEGRVHPLTRANYLASPPLVVAYALAGTVDIDFETQPIGTGKDGKQIFFRDIWPSNKEVAEVVQSSVLPDMFKATYEAITKGNSMWNQLSVASGTLYEWDPKSTYIHEPPYFKGMTMSPPGPHGVKDAYCLLNFGDSITTDHISPAGSIHKDSPAAKYLMERGVDRRDFNSYGSRRGNDEIMARGTFANIRIVNKHLKGEVGPKTVHIPTGEKLSVFDAAMKYRNEGRDTIILAGAEYGSGSSRDWAAKGPMLLGVKAVISKSFERIHRSNLVGMGIIPLCFKAGEDAETLGLTGQELYTIELPNNVSEIKPGQDVTVVTNNGKSFTCTLRFDTEVELAYFDHGGILQYVIRNLIKQ

>Citrus clementina (CBE71056.1)

MATENPFKSILKTLQRPDGGEFGKYYSLPALNDPRIGKLPYSIKILLESAIRNCDEFQVKSKDVEKIIDWETTSPKQVEIPFKPARVLLQDFTGVPAVVDLACMRDAMNKLGGDSNKINPLVPVDLVIDHSVQVDVARSENAVQANMEFEFRRNKERFAFLKWGSNAFHNMLVVPPGSGIVHQVNLEYLGRVVFNTNGMLYPDSVVGTDSHTTMIDGLGVAGWGVGGIEAEAAMLGQPMSMVLPGVVGFKLSGKLQDGVTATDLVLTVTQMLRKHGVVGMFVEFYGEGMSELSLADRATIANMSPEYGATMGFFPVDHVTLQYLKLTGRSDDTVSMIESYLRANKMFVDYSEPQSERVYSSYLELNLEEVVPCVSGPKRPHDRVPLNEMKADWHACLDNRVGFKGFAIPKEYQSKVAEFNFHGTPAQLRHGDVVIAAITSCTNTSNPSVMLGAALVAKKACELGLEVKPWIKTSLAPGSGVVTKYLQNSGLQKYLNHLGFHIVGYGCTTCIGNSGDIDDAVAAAITENDIVAAAVLSGNRNFEGRVHPLTRANYLASPPLVVAYALAGSVNIDFETEPVGVGKDGKKIFLRDIWPSSEEVAHVVQKSVLPDMFKATYEAITKGNPMWNQLSVPSGTLYAWDPKSTYIHEPPYFKDMTMSPPGPHGVKGAYCLLNFGDSITTDHISPAGSIHKDSPAAKYLMERGVDRRDFNSYGSRRGNDEIMARGTFANIRLVNKLLNGEVGPKTIHIPTGEKLSVFDAAMRYKNEGHDTVILAGAEYGSGSSRDWAAKGPMLLGVKAVIAKSFERIHRSNLVGMGIIPLCFKPGEDAETHGLTGHERYTIDLPSSVSEIRPGQDVRVVTDSGKSFTCVIRFDTEVELAYFDHGGILQYVIRNLINVRQ

>Ricinus communis (XP_002530635.1)

MVNESPFKSILKTLEKADGGAFGKYYSLPALNDPRIDRLPYSIRILLESAIRNCDEFQVKSNDVEKIIDWENTSPKQVEIPFKPARVLLQDFTGVPAVVDLACMRDAMNSLGGDSNKINPLVPVDLVIDHSVQVDVARSENAVQANMELEFQRNNERFAFLKWGSNAFHNMLVVPPGSGIVHQVNLEYLGRVVFNTDGMLYPDSVVGTDSHTTMIDGLGVAGWGVGGIEAEAAMLGQPMSMVLPGVVGFKLSGKLRDGVTATDLVLTVTQMLRKHGVVGKFVEFYGEGMSELSLADRATIANMSPEYGATMGFFPVDHVTLQYLKLTGRSDETVSMIESYLRANRMFVDYSEPQIERVYSSYLELNLEDVEPCIAGPKRPHDRVPLKEMKADWHSCLDNRVGFKGFAVPKESQSKVAEFNFHGTPAQLRHGDVVIAAITSCTNTSNPSVMLGAALVAKKACELGLEVKPWIKTSLAPGSGVVTKYLQKSGLQKYLNQLGFHIVGYGCTTCIGNSGDIDEAVASAITENDLVAAAVLSGNRNFEGRVHPLTRANYLASPPLVVAYALAGTVDIDFETEPIGVGKDGKKIYFRDIWPSNEEVAKVVQSNVLPDMFKATYEAITKGNPMWNHLSVPSSTLYSWDPKSTYIHEPPYFRNMTMSPPGPHGVKNAYCLLNFGDSITTDHISPAGSIHKDSPAARYLMERGVDRRDFNSYGSRRGNDEIMARGTFANIRLVNKFLGGEVGPKTVHIPSGEKLSVFDAAMRYKSEGHDTVILAGAEYGSGSSRDWAAKGPMLLGVKAVIAKSFERIHRSNLVGMGIIPLCFKPGEDAETFGLTGHERYNIDLPSSVAEIRPGQDVTVTTDNGKSFTCTLRFDTEVELAYFDHGGILPFVIRNLIQAKH

>Populus trichocarpa c1 (XM_002327692.1)

MVNENPFKSILKTLEKPGGEFGKYYSLPALNDPRIDRLPYSIKILLESAIRNCDEFQVKSNDVEKIIDWENTAPKLVEIPFKPARVLLQDFTGVPAVVDLACMRDAMNNLGGDSNKINPLVPVDLVIDHSVQVDVARSENAVQANMELEFQRNKERFAFLKWGSNAFQNMLVVPPGSGIVHQVNLEYLGRVVFNTNGLLYPDSVVGTDSHTTMIDGLGVAGWGVGGIEAEAAMLGQPMSMVLPGVVGFKLSGKLRDGVTATDLVLTVTQMLRKHGVVGKFVEFYGEGMSELSLADRATIANMSPEYGATMGFFPVDHVTLQYLKLTGRSDETISMIESYLRANRMFVDYSEPQIERMYSSYLALNLEDVEPCISGPKRPHDRVPLREMKADWHACLDNRVGFKGFAIPKESQSKVAEFSFRGTSAQLRHGDVVIAAITSCTNTSNPSVMLGAALVAKKACELGLEVKPWIKTSLAPGSGVVTKYLEKSGLQKYLNQLGFNIVGYGCTTCIGNSGDIDEAVASAITENDLVAAAVLSGNRNFEGRVHPLTRANYLASPPLVVAYALAGTVGIDFETEPIGVGKDGKKIFFRDIWPSNDEVAQVVHSSVLPDMFKATYQAITKGNPMWNQLSVPSGTLYAWDSKSTYIHEPPYFKSMTMSPPGPHGVKDAYCLLNFGDSITTDHISPAGSIHKDSPAARYLMERGVDRRDFNSYGSRRGNDEVMARGTFANIRLVNKLLGGEVGPKTIHISTGEKLSVFDVAMRYKSEGRDTVILAGAEYGSGSSRDWAAKGPMLLGVKAVIAKSFERIHRSNLVGMGIIPLCFKPGEDAETLGLTGHECYSIDLPSNVSEIRPGQDVTVVTDNGKSFACTLRFDTEVELAYFDHGGILQYAIRNLIHTNH

>Populus trichocarpa c2 (XM_002331719.1)

MANENPFKSILKTLEKPGGEFGKYYSLPALNDPRIDRLPYSIKILLESAIRNCDEFQVKSKDVEKIIDWENTSPKLVEIPFKPARVLLQDFTGVPAVVDLACMRDAMSNLGGDSNKINPLVPVDLVIDHSVQVDVSRSENAVQANMEFEFHRNKERFAFLKWGSNAFQNMLVVPPGSGIVHQVNLEYLGRVVFNTNGLLYPDSVVGTDSHTTMIDGLGVAGWGVGGIEAEAAMLGQPMSMVLPGVVGFKLSGKLRDGVTATDLVLTVTQMLRKHGVVGKFVEFYGEGMSELSLADRATIANMSPEYGATMGFFPVDHVTLQYLKLTGRSDGTVSMIESYLRANKMFVDYSEPQIDRVYSSYIALNLRDVEPCISGPKRPHDRVPLREMKADWHACLDNKVGFKGFAIPKESQSKVAEFNFHGTPAQLRHGDVVIAAITSCTNTSNPSVMLGSALVAKKACELGLEVKPWIKTSLAPGSGVVTKYMEKSGLQKYLNQLGFHIVGYGCTTCIGNSGDIDEAVASAITENDVVAAAVLSGNRNFEGRVHPLTRANYLASPPLVVAYALAGTVDIDFETEPIGVGKDGKKIFFRDIWPSNDEVAQVVHSSVLPDMFKATYQAITKGNPMWNQLSIPSGTLYDWDPKSTYIHEPPYFKSMTMSPPGPHGVKDAYCLLNFGDSITTDHISPAGSIHKDSPAARYLMERGVDRRDFNSYGSRRGNDDVMARGTFANIRIVNKLLGGEVGPKTIHFPTREKLSVFDVAMRYKSEGHDTVILAGAEYGSGSSRDWAAKGPMLLGVKAVMAKSFERIHRSNLVGMGIIPLCFKSGEDAETLGLTGHERYSLDLPSNVSEIRPGQDVTVVTDNGKQFTCTLRYDTEVELAYFDHGGILQYAIRNLIHTKH

>Vitis vinifera (XM_002263301.2)

MASSNPFASILKTLEKPAGGEFGKYYSLPALGDPRIDRLPYSIRILLESAIRNCDEFQVKAKDVEKIIDWENSSPKQVEIPFKPARVLLQDFTGVPAVVDLACMRDAMNKLGGDSNKINPLVPVDLVIDHSVQVDVAGSENAVQANMELEFQRNKERFGFLKWGSNAFHNMLVVPPGSGIVHQVNLEYLGRVVFNTEGILYPDSVVGTDSHTTMIDGLGVAGWGVGGIEAEAAMLGQPMSMVLPGVVGFKLSGKLRDGVTATDLVLTVTQMLRKHGVVGKFVEFYGEGMRELSLADRATIANMSPEYGATMGFFPVDHVTLQYLKLTGRRDETVSMIESYLRANNMFVDYSQPQVEKVYSSYLELNLEDVEPCVSGPKRPHDRVPLKEMKADWHSCLDNKVGFKGFAIPKESQSKVVEFSYHGTPAQLRHGDVVIAAITSCTNTSNPSVMLGAALVAKKACELGLEVKPWIKTSLAPGSGVVTKYLEKSGLQKYLNQLGFHIVGYGCTTCIGNSGDINESVASAISENDMVAAAVLSGNRNFEGRVHPLTRANYLASPPLVVAYALAGTVDIDFEKEPIGVGKDGKQIFFRDIWPSTEEVANVVQSSVLPAMFKATYEAITQGNPMWNQLSVPSSTLYTWDPKSTYIHDPPYFKSMTMSPPGPHGVKDAYCLLNFGDSITTDHISPAGSIHKDSPAARYLMERGVDRRDFNSYGSRRGNDEIMARGTFANIRIVNKLLKGEVGPKTLHIPSGEKLSVFDAAMRYKSEGQDTIILAGAEYGSGSSRDWAAKGPMLLGVKAVIAKSFERIHRSNLVGMGIIPLCFKPGQDAETLGLTGHERYTIDLPSSVSEIKPGQDITVVTDNGKSFTCTMRFDTEVELAYFDHGGILQYAIRNLIGGRS

>Oryza sativa c1 (BAD05751.1)

MAAEHPFKNILTTLPKPGGGEYGKFYSLPALNDPRIDKLPYSIRILLESAIRNCDNFQVNQNDVEKIIDWENTSPKLAEIPFKPARVLLQDFTGVPAVVDLAAMRDAMAKLGSDANKINPLVPVDLVIDHSVQVDVARSPNAVQSNMELEFKRNNERFGFLKWGSTAFHNMLVVPPGSGIVHQVNLEYLGRVVFNTDGIMYPDSVVGTDSHTTMIDGLGVAGWGVGGIEAEATMLGQPMSMVLPGVVGFKLTGKLQNGVTATDLVLTVTQMLRKHGVVGKFVEFYGEGMGKLSLADRATIANMSPEYGATMGFFPVDHVTLDYLKLTGRSDETVAMIEAYLRANKMFVDYNEPQTERVYSSYLELDLNEVEPCISGPKRPHDRVLLKEMKSDWHSCLDNRVGFKGFAVPKEQQDKVVKFDFHGQPAELKHGSVVIAAITSCTNTSNPSVMLGAALVAKKACELGLEVKPWVKTSLAPGSGVVTKYLLQSGLQEYLNKQGFHVVGYGCTTCIGNSGDLDESVSAAISENDVVAAAVLSGNRNFEGRVHPLTRANYLASPPLVVAYALAGTVDIDFEKEPIGVGKDGKEVFFRDIWPSTEEIAEVVQSSVLPDMFKSTYEAITKGNPMWNQLTVPEASLYSWDPNSTYIHEPPYFKDMTMSPPGPHGVKNAYCLLNFGDSITTDHISPAGSIHKDSPAAKYLLERGVDRKDFNSYGSRRGNDEVMARGTFANIRIVNKFLNGEVGPKTVHVPTGEKLYVFDAALKYKSEGHDTIVLAGAEYGSGSSRDWAAKGPMLLGVKAVIAKSFERIHRSNLVGMGIIPLCFKAGEDADSLGLTGHERYTIDLPTNVSEIRPGQDITVTTDNGKSFTCTLRFDTEVELAYFNHGGILPYVIRNLAQN

>Oryza sativa c2 (NP_001057461.2)

MSPPRTNMYAHLCSPGSPLVATMLHHVHSTGQQDNTGVPAIVDLAAMRDVMAKLGCDPYQINPLIPVDVVIDHAVRVDVVRSHDALDKNMELEFDRNKERFGFLKWASTAFHKMQVFPPGSGIVHQVNLEYLARVVFNADGIMYPDSVVGTDSHTTMINSLGVAGWGVGGIEAIVAMLGQPMDMVLPGVVGFKLSGMLRDGVTATDLVLTITQMLRKHGVVGKFVEFYGVGVGELSLPARATIANMSPEYGASMGFFPVDHVTLDYLKLTGRSNETVSMIEAYLRANNMFVEHHEPHTERVYSSYLELNLIDVEPCISGPKRPHDRVPLKEMKSDWHACLDSRVGFKGFAVPRECQDKVVKFDFQGQPAEIKHGSVVLAAICSSTNTSNPSVIVGAGLVAKKACELGLEGLPFRFRSKNRSSPVYRKQVKPWVKTSFTHGSAVTREYLKHSHLQDYLNQQGFHLAAFGCATCVGNSGDLDESVSAAITENDIVSVAVLSANRNFEGRVHPLTRANYLASPPLVVAYALAGTVDIDFEKEPIGHGKDGNEVYLRDIWPTNEEIEQVVKSSVLPHMFTQTYESIKRCNRRWNELRVPGEAAALYPWDPSSTYIRKPPYLEGMAMSPPSRPRSVRDAYCLLNLGDSVTTDHISYSGSITPGSAAAEYLRAAGVADRERLGSYGGRRGNDEVVVRGAFANARIVNKLMNGKVGPKTVHVPTGEELCVFDAAIKYKSEGHNMVIVIAGAEYGSGSSRDSAAKGPMLLTTVV

>Zea mays c1 (NP_001159229.1)

MIDGLGVAGWGVGGIEAEAAMLGQPMSMVLPGVVGFKLSGKLRNGVTATDLVLTVTQMLRKHGVVGKFVEFYGQGMSELSLADRATIANMSPEYGATMGFFPVDGKTLDYLKLTGRSDDTVAMVESYLRANKMFVDHSQAEAERVYSSYLELNLEEVEPCLSGPKRPHDRVTLKNMKSDWLSCLDSDVGFKGFAVPKESQGKVAEFLFHGTPAKIKHGDVVIAAITSCTNTSNPNVMLGAALVAKKACELGLEVKPWIKTSLAPGSGVVKQYLDKSGLQKYLDQLGFNIVGYGCTTCIGNSGELDESVSAAITENDIVSAAVLSGNRNFEGRVHPLTRANYLASPPLVVAYALAGTVNIDFEKEPIGISKDGKEVYFRDVWPSTEEIAEVVKSSVLPDMFKSTYESITQGNPMWNELPVSTSTLYPWDPSSTYIHEPPYFKDMTMTPPGPRPVKDAYCLLNFGDSITTDHISPAGNIHPDSAAATYLKERGVERKDFNSYGSRRGNDEIMARGTFANIRLVNKFLKGEVGPKTIHVPSGDKLSVFDAAMKYKNEGHDTIILAGAEYGSGSSRDWAAKGPMLQGVKAVIAKSFERIHRSNLAGMGIIPLCFKAGEDADTLGLTGHERYTVHLPTNVSEIKPGQDVTVTTDNGKSFTCTLRFDTEVELAYYDHGGILPYVIRKIAEQ

>Zea mays c2 (NP_001136484.1)

MIEAYLRANKMFVDYNEPPTERIYSSYLELNLDEVEPSMSGPKRPHDRVPLKEMKSDWHACLDNKVGFKGFAVPKEQQDKVVKFDFHGQPAEMKHGSVVIAAITSCTNTSNPSVMLGAGLVAKKACELGLEVKPWVKTSLAPGSGVVTKYLLQSGLQEYLNQQGFHIVGYGCTTCIGNSGDLDESVSTAITENDVVAAAVLSGNRNFEGRVHPLTRANYLASPPLVVAYALAGTVDIDFEKEPIGFGKDGKEVYFRDIWPSTEEIAQVVQSSVLPDMFKGTYEAITKGNPMWNQLTVPEASLYSWDSKSTYIHEPPYFKDMTMSPPGPSTVKDAYCLLNFGDSITTDHISPAGSIHKDSPAAKYLMERGVDRKDFNSYGSRRGNDEVMARGTFANIRIVNKFLNGEVGPKTIHVPTGEKLSVFDAAMRYKSEGHATIILAGAEYGSGSSRDWAAKGPMLLGVKAVIAKSFERIHRSNLVGMGIIPLCFKAGEDADSLGLTGHERYSIDLPTNLSEIRPGQDVTVTTDNGKSFTCIVRFDTEVELAYFNHGGILPYVIRNLAAAHN

>Brachypodium distachyon (XP_003573387.1)

MATSATEHAFKNILTSLPKPGGGEFGKFYSLPALNDPRIDKLPYSIRILLESAIRNCDDFQVTKNDVEKIIDWENTSPKLAEIPFKPARVLLQDFTGVPAVVDLAAMRDAMAKLGSDANKINPLVPVDLVIDHSVQVDVARSHNALQSNMELEFIRNKERFGFLKWGSTAFQNMLVVPPGSGIVHQVNLEYLGRVVFNTDGIMYPDSVVGTDSHTTMIDGLGVAGWGVGGIEAEATMLGQPMSMVLPGVVGFKLTGNLRSGVTATDLVLTVTQMLRKHGVVGKFVEFYGEGMGKLSLADRATIANMSPEYGATMGFFPVDHVTLDYLKLTGRSDETVSMIEAYLRANNMFVDYNEPQLERVYSSYLALDLDEVEPCISGPKRPHDRVTLKEMKSDWHSCLDNKVGFKGFAVPKEQQDKVVKFDFHGQPAELKHGSVVIAAITSCTNTSNPSVMLGAALVAKKACELGLEVKPWVKTSLAPGSGVVTKYLLKSGLQEYFNKQGFHLVGYGCTTCIGNSGDLDESVSAAITENDVVAAAVLSGNRNFEGRVHPLTRANYLASPPLVVAYALAGTVDIDFEKEPIGVGKDGKEVYFRDIWPTTEEIAQVVQSSVLPDMFKSTYEAITKGNPMWNQLPVPEATLYSWDPNSTYIHEPPYFKDMTMSPPGPHPVKDAYCLLNFGDSITTDHISPAGSIHKDSPAAKFLLERGVDRKDFNSYGSRRGNDEIMARGTFANIRIVNKFLGGEVGPKTIHVPSGEKLSVFDAATRYKSEGHDTIILAGAEYGSGSSRDWAAKGPMLLGVKAVISKSFERIHRSNLVGMGIIPLCYKTGEDADSLGLTGHERFTINLPTDVSKIRPGQDVTITTDNGKSFSCTLRFDTEVELAYYNHGGILPYVIRSLAGQQN

>Caenorhabditis elegans (NP_509898.1)

MAFNNLIRNLAIGDNVYKYFDLNGLNDARYNELPISIKYLLEAAVRHCDEFHVLKKDVETILDWKNSQRNQAEIPFKPARVILQDFTGVPAVVDLAAMRDAVQNMGADPAKINPVCPVDLVIDHSVQVDHYGNLEALAKNQSIEFERNRERFNFLKWGSKAFDNLLIVPPGSGIVHQVNLEYLARTVFVGKDGVLYPDSVVGTDSHTTMIDGSGVLGWGVGGIEAEAVMLGQPISMVIPEVIGYELVGTLSDTVTSTDLVLTITKNLRDLGVVGKFVEFFGTGVASLSIADRATIANMCPEYGATIGFFPVDSRTIDYLTQTGRDTDYTQRVEQYLKSVGMFVNFTDDSYRPTYTTTLKLDLGSVVPSVSGPKRPHDRVELASLAQDFSKGLTDKISFKAFGLKPEDATKSVTITNHGRTAELTHGSVVIAAITSCTNTSNPSVMLAAGLVAKKAVELGLNVQPYVKTSLSPGSGVVTKYLEASGLLPYLEKIGFNIAGYGCMTCIGNSGPLDEPVTKAIEENNLVVAGVLSGNRNFEGRIHPHVRANYLASPPLAVLYSIIGNVNVDINGVLAVTPDGKEIRLADIWPTRKEVAKFEEEFVKPQFFREVYANIELGSTEWQQLECPAVKLYPWDDASTYIKKVPFFDGMTSELPSQSDIVNAHVLLNLGDSVTTDHISPAGSISKTSPAARFLAGRGVTPRDFNTYGARRGNDEIMARGTFANIRLVNKLASKVGPITLHVPSGEELDIFDAAQKYKDAGIPAIILAGKEYGCGSSRDWAAKGPFLQGVKAVIAESFERIHRSNLIGMGIIPFQYQAGQNADSLGLTGKEQFSIGVPDDLKPGQLIDVNVSNGSVFQVICRFDTEVELTYYRNGGILQYMIRKLIQ

>Drosophila melanogaster c1 (NP_477371.1)

MSGSGANPFAQFQESFTQDGNVYKYFDLPSIDSKYESLPFSIRVLLESAVRNCDNFHVLEKDVQSILGWTPSLKQETSDVEVSFKPARVILQDFTGVPAVVDFAAMRDAVRELGGNPEKINPICPADLVIDHSVQVDFVRSSDALTKNESLEFQRNKERFTFLKWGARAFDNMLIVPPGSGIVHQVNLEYLARVVFESDSSADGSKILYPDSVVGTDSHTTMINGLGVLGWGVGGIEAEAVMLGQSISMLLPEVIGYRLEGKLGPLATSTDLVLTITKHLRQLGVVGKFVEFYGPGVAELSIADRATISNMCPEYGATVGYFPIDENTLSYMRQTNRSEKKIDIIRKYLKATRQLRDYSLVDQDPQYTESVTLDLSTVVTSVSGPKRPHDRVSVSSMCEDFKSCLISPVGFKGFAIPPSALAASGEFQWDDGKSYKIGHGSVVIAAITSCTNTSNPSVMLGAGLLAKNAVQKGLSILPYIKTSLSPGSGVVTYYLRESGVIPYLEQLGFDIVGYGCMTCIGNSGPLDENVVNTIEKNGLVCCGVLSGNRNFEGRIHPNTRANYLASPLLVIAYAIAGRVDIDFEIEPLGVDSNGKEVFLRDIWPTRSEIQEVEHKHVIPAMFQEVYSKIQLGSRDWQTLEVSDSKLYPWSEISTYIKLPPFFEGMTRALPKLKGIEKARCLLLLGDSVTTDHISPAGSIARKSPAARYLSERGLTPRDFNSYGSRRGNDAVMARGTFANIRLVNKLASKTGPSTLHVPSGEEMDIFDAAERYASEGTPLVLVVGKDYGSGSSRDWAAKGPFLLGIKAVIAESYERIHRSNLVGMGIIPLQFLPGQSADTLKLSGREVYNIVLPEGELKPGQRIQVDADGNVFETTLRFDTEVDITYYKNGGILNYMIRKMLD

>Drosophila melanogaster c2 (NP_524303.2)

MSGANPFAQFEKTFSQAGTTYKYFDLASIDSKYDQLPYSIRVLLESAVRNCDNFHILEKDVQSILGWSPALKQGSNDVEVSFKPARVILQDFTGVPAVVDFAAMRDAVLDLGGDPEKINPICPADLVIDHSVQVDFARAPDALAKNQSLEFERNKERFTFLKWGAKAFNNMLIVPPGSGIVHQVNLEYLARVVFENDATDGSKILYPDSVVGTDSHTTMINGLGVLGWGVGGIEAEAVMLGQSISMLLPEVIGYKLEGKLSPLVTSTDLVLTITKHLRQLGVVGKFVEFYGPGVAELSIADRATISNMCPEYGATVGYFPIDENTLGYMKQTNRSEKKIDIIRQYLKATQQLRNYADAAQDPKFTQSITLDLSTVVTSVSGPKRPHDRVSVSDMPEDFKSCLSSPVGFKGFAIAPEAQSAFGEFQWDDGKTYKLHHGSVVIAAITSCTNTSNPSVMLGAGLLAKKAVEKGLSILPYIKTSLSPGSGVVTYYLKESGVIPYLEKLGFDIVGYGCMTCIGNSGPLEENVVNTIEKNGLVCAGVLSGNRNFEGRIHPNTRANYLASPLLVIAYAIAGRVDIDFEKEPLGVDANGKNVFLQDIWPTRSEIQEVENKHVIPAMFQEVYSKIELGSQDWQTLQVSEGKLFSWSADSTYIKRPPFFEGMTRDLPKLQSIQKARCLLFLGDSVTTDHISPAGSIARTSPAARFLSERNITPRDFNSYGSRRGNDAIMSRGTFANIRLVNKLVEKTGPRTVHIPSQEELDIFDAAERYREEGTPLVLVVGKDYGSGSSRDWAAKGPFLLGVKAVIAESYERIHRSNLVGMGIIPLQFLPGQSAETLNLTGREVYNIALPESGLKPGQKIQVEADGTVFETILRFDTEVDITYYKNGGILNYMIRKMLS

>Gallus gallus (NP_001025707.1)

MSNPFVQIVEPLDAKEPVKKFFNLSKLEDVRYARLPFSIRVLLEAAIRNCDEFLVKKQDVENILNWKVMQHKNVEVPFKPARVILQDFTGVPAVVDFAAMRDAVKKLGGDPEKINPICPADLVIDHSIQVDFNRRSDSLQKNQDLEFERNKERFEFLKWGSQAFKNMRIIPPGSGIIHQVNLEYLARVVMDQDGYYYPDSVVGTDSHTTMVDGLGVLGWGVGGIEAEAVMLGQPISMVLPEVVGYKLLGNPQPLVTSTDIVLTITKHLRQVGVVGKFVEFFGPGVAQLSIADRATIANMCPEYGATAAYFPVDDISIGYLVQTGRDKEKVLCTKKYLEAVGMLRDFKNSSQDPDFTQVVELDLHTVVPCCSGPKRPQDKVAVSDMKKDFETCLGAKQGFKGFQIAPDRHNSVIKFNFEGCDFELAHGSVVIAAITSCTNTSNPSVMLGAGLLAKKAVEAGLTVKPYIKTSLSPGSGVVTYYLRESGVMSYLSQLGFDVVGYGCMTCIGNSGPLPDSVVEAITQGDLVAVGVLSGNRNFEGRVHPNTRANYLASPPLVIAYAIAGTVRIDFEKEPLGISASGKKIFLKDIWPTRNEIQAVERQYVIPGMFKEVYQKIETVNEAWNALDAPSDKLYTWNPKSTYIKSPPFFDGLTLALQTPKTIEDAYVLLNFGDSVTTDHISPAGNIARNSPAARYLTSRGLTPREFNSYGSRRGNDAVMARGTFANIRLVNKFIDKQGPQTIHFPSGETLDVFDAAERYKQAGHPLIVLAGKEYGAGSSRDWAAKGPFLLGVKAVLAESYERIHRSNLVGMGVIPLQYLPGEDARTLGLTGRERYTIIIPENLKPQMNIQIKLDTGKTFHAIMRFDTDVELTYFHNGGILNYMIRKMAS

>Felis catus (XP_003995587.1)

MSNPFAHLVEPLDPAQPGKKFFNLNKLKDSRYERLPFSIRVLLEAAIRNCDQFLVKKNDIENILNWNVMQHKNIEVPFKPARVILQDFTGVPAVVDFAAMRDAVKKLGGDPEKINPVCPADLVIDHSIQVDFNRRADSLQKNQDLEFERNRERFEFLKWGSQAFRNMRIIPPGSGIIHQVNLEYLARVVFDHDGYYYPDSLVGTDSHTTMIDGLGVLGWGVGGIEAEAVMLGQPISMVLPQVIGYRLMGNPHPLVTSTDIVLTITKHLRQVGVVGKFVEFFGPGVAQLSIADRATIANMCPEYGATAAFFPVDEVSIKYLVQTGRDEEKVKQMKKYLQAVGMFRDFSNLSQDPEFAQVVELNLRTVVPCCSGPKRPQDKVAVTDMKKDFESCLGAKQGFKGFQVALDHHNDHKTFIYNNSEFTLTHGSVVIAAITSCTNTSNPSVMLGAGLLAKKAVNAGLHVKPYIKTSLSPGSGVVTYYLRESGVMPYLSQLGFDVVGYGCMTCIGNSGPLPEAVVEAITQGDLVAVGVLSGNRNFEGRVHPNTRANYLASPPLVIAYAIAGTIRINFEKEPLGVNAKGQQVFLKDIWPTRDEIQAVERQYVIPGMFKEVYQKIETVNESWNALAAPSDKLYCWNPKSTYIKSPPFFENLTSDIQPPKSIVDAYVLLNLGDSVTTDHISPAGNIARNSPAARYLTNRGLTPREFNSYGSRRGNDAIMARGTFANIRLLNKFLNKQAPQTIHLPSGEILDVFDAAEQYQQAGLPLIVLAGKEYGSGSSRDWAAKGPFLLGIKAVLAESYERIHRSNLVGMGVIPLEYLPGETADILGLTGRERYTIIIPENLKPRMKVQVKLDTGKSFQAVMRFDTDVELAYFHNGGILNYMVRKMAK

>Homo sapiens (NP_002188.1

MSNPFAHLAEPLDPVQPGKKFFNLNKLEDSRYGRLPFSIRVLLEAAIRNCDEFLVKKQDIENILHWNVTQHKNIEVPFKPARVILQDFTGVPAVVDFAAMRDAVKKLGGDPEKINPVCPADLVIDHSIQVDFNRRADSLQKNQDLEFERNRERFEFLKWGSQAFHNMRIIPPGSGIIHQVNLEYLARVVFDQDGYYYPDSLVGTDSHTTMIDGLGILGWGVGGIEAEAVMLGQPISMVLPQVIGYRLMGKPHPLVTSTDIVLTITKHLRQVGVVGKFVEFFGPGVAQLSIADRATIANMCPEYGATAAFFPVDEVSITYLVQTGRDEEKLKYIKKYLQAVGMFRDFNDPSQDPDFTQVVELDLKTVVPCCSGPKRPQDKVAVSDMKKDFESCLGAKQGFKGFQVAPEHHNDHKTFIYDNTEFTLAHGSVVIAAITSCTNTSNPSVMLGAGLLAKKAVDAGLNVMPYIKTSLSPGSGVVTYYLQESGVMPYLSQLGFDVVGYGCMTCIGNSGPLPEPVVEAITQGDLVAVGVLSGNRNFEGRVHPNTRANYLASPPLVIAYAIAGTIRIDFEKEPLGVNAKGQQVFLKDIWPTRDEIQAVERQYVIPGMFKEVYQKIETVNESWNALATPSDKLFFWNSKSTYIKSPPFFENLTLDLQPPKSIVDAYVLLNLGDSVTTDHISPAGNIARNSPAARYLTNRGLTPREFNSYGSRRGNDAVMARGTFANIRLLNRFLNKQAPQTIHLPSGEILDVFDAAERYQQAGLPLIVLAGKEYGAGSSRDWAAKGPFLLGIKAVLAESYERIHRSNLVGMGVIPLEYLPGENADALGLTGQERYTIIIPENLKPQMKVQVKLDTGKTFQAVMRFDTDVELTYFLNGGILNYMIRKMAK

>Mus musculus aconitase (NM_007386.2)

MKNPFAHLAEPLDAAQPGKRFFNLNKLEDSRYGRLPFSIRVLLEAAVRNCDEFLVKKNDIENILNWNVMQHKNIEVPFKPARVILQDFTGVPAVVDFAAMRDAVKKLGGNPEKINPVCPADLVIDHSIQVDFNRRADSLQKNQDLEFERNKERFEFLKWGSQAFCNMRIIPPGSGIIHQVNLEYLARVVFDQDGCYYPDSLVGTDSHTTMIDGLGVLGWGVGGIEAEAVMLGQPISMVLPQVIGYKLMGKPHPLVTSTDIVLTITKHLRQVGVVGKFVEFFGPGVAQLSIADRATIANMCPEYGATAAFFPVDEVSIAYLLQTGREEDKVKHIQKYLQAVGMFRDFNDTSQDPDFTQVVELDLKTVVPCCSGPKRPQDKVAVSEMKKDFESCLGAKQGFKGFQVAPDRHNDRKTFLYSNSEFTLAHGSVVIAAITSCTNTSNPSVMLGAGLLAKKAVEAGLSVKPYIKTSLSPGSGVVTYYLRESGVMPYLSQLGFDVVGYGCMTCIGNSGPLPEPVVEAITQGDLVAVGVLSGNRNFEGRVHPNTRANYLASPPLVIAYAIAGTVRIDFEKEPLGVNAQGRQVFLKDIWPTRDEIQAVERQHVIPGMFKEVYQKIETVNKSWNALAAPSEKLYAWNPKSTYIKSPPFFESLTLDLQPPKSIVDAYVLLNLGDSVTTDHISPAGNIARNSPAARYLTNRGLTPREFNSYGSRRGNDAIMARGTFANIRLLNKFLNKQAPQTVHLPSGETLDVFDAAERYQQAGLPLIVLAGKEYGSGSSRDWAAKGPFLLGIKAVLAESYERIHRSNLVGMGVIPLEYLPGETADSLGLTGRERYTINIPEDLKPRMTVQIKLDTGKTFQAVMRFDTDVELTYFHNGGILNYMIRKMAQ

MITOCHONDRIAL

>Arabidopsis thaliana m1 (NP_178634.2)

MYLTASSSASSSIIRAASSRSSSLFSFRSVLSPSVSSTSPSSLLARRSFGTISPAFRRWSHSFHSKPSPFRFTSQIRAVSPVLDRLQRTFSSMASEHPFKGIFTTLPKPGGGEFGKFYSLPALNDPRVDKLPYSIRILLESAIRNCDNFQVTKEDVEKIIDWEKTSPKQVEIPFKPARVLLQDFTGVPAVVDLACMRDAMNKLGSDSNKINPLVPVDLVIDHSVQVDVARSENAVQANMELEFQRNKERFAFLKWGSTAFQNMLVVPPGSGIVHQVNLEYLGRVVFNTKGLLYPDSVVGTDSHTTMIDGLGVAGWGVGGIEAEATMLGQPMSMVLPGVVGFKLAGKMRNGVTATDLVLTVTQMLRKHGVVGKFVEFYGNGMSGLSLADRATIANMSPEYGATMGFFPVDHVTLQYLKLTGRSDETVAMIEAYLRANNMFVDYNEPQQDRVYSSYLELNLDDVEPCISGPKRPHDRVTLKEMKADWHSCLDSKVGFKGFAIPKEAQEKVVNFSFDGQPAELKHGSVVIAAITSCTNTSNPSVMLGAGLVAKKACDLGLQVKPWIKTSLAPGSGVVTKYLLKSGLQEYLNEQGFNIVGYGCTTCIGNSGEINESVGAAITENDIVAAAVLSGNRNFEGRVHPLTRANYLASPPLVVAYALAGTVNIDFETEPIGKGKNGKDVFLRDIWPTTEEIAEVVQSSVLPDMFRATYESITKGNPMWNKLSVPENTLYSWDPNSTYIHEPPYFKDMTMDPPGPHNVKDAYCLLNFGDSITTDHISPAGNIQKDSPAAKFLMERGVDRKDFNSYGSRRGNDEIMARGTFANIRIVNKLMNGEVGPKTVHIPSGEKLSVFDAAMRYKSSGEDTIILAGAEYGSGSSRDWAAKGPMLQGVKAVIAKSFERIHRSNLVGMGIIPLCFKSGEDADTLGLTGHERYTIHLPTDISEIRPGQDVTVTTDNGKSFTCTVRFDTEVELAYFNHGGILPYVIRNLSKQ

>Arabidopsis thaliana m2 (NP_567763.2)

MYRRATSGVRSASARLSSSLSRIASSETASVSAPSASSLRNQTNRSKSFSSALRSFRVCSASTRWSHGGSWGSPASLRAQARNSTPVMEKFERKYATMASEHSYKDILTSLPKPGGGEYGKYYSLPALNDPRIDKLPFSVRILLESAIRNCDNYQVTKDDVEKILDWENTSTKQVEIAFKPARVILQDFTGVPVLVDLASMRDAVKNLGSDPSKINPLVPVDLVVDHSIQVDFARSEDAAQKNLELEFKRNKERFTFLKWGSTAFQNMLVVPPGSGIVHQVNLEYLGRVVFNSKGFLYPDSVVGTDSHTTMIDGLGVAGWGVGGIEAEAAMLGQPMSMVLPGVVGFKLDGKLKEGVTATDLVLTVTQILRKHGVVGKFVEFYGEGMSELSLADRATIANMSPEYGATMGFFPVDHVTLEYLKLTGRSDETVSMIESYLRANNMFVDYNEPQQERAYTSYLQLDLGHVEPCISGPKRPHDRVPLKDMKADWHACLDNPVGFKGFAVPKEKQEEVVKFSYNGQPAEIKHGSVVIAAITSCTNTSNPSVMIGAALVAKKASDLGLKVKPWVKTSLAPGSRVVEKYLDRSGLRESLTKQGFEIVGYGCTTCIGNSGNLDPEVASAIEGTDIIPAAVLSGNRNFEGRVHPQTRANYLASPPLVVAYALAGTVDIDFEKEPIGTRSDGKSVYLRDVWPSNEEVAQVVQYSVLPSMFKSSYETITEGNPLWNELSAPSSTLYSWDPNSTYIHEPPYFKNMTANPPGPREVKDAYCLLNFGDSVTTDHISPAGNIQKTSPAAKFLMDRGVISEDFNSYGSRRGNDEVMARGTFANIRIVNKLLKGEVGPNTVHIPTGEKLSVFDAASKYKTAEQDTIILAGAEYGSGSSRDWAAKGPLLLGVKAVIAKSFERIHRSNLAGMGIIPLCFKAGEDAETLGLTGHERYTVHLPTKVSDIRPGQDVTVTTDSGKSFVCTLRFDTEVELAYYDHGGILPYVIRSLSAK

>Citrus clementina m1 (CBE71058.1)

MAAEHPFKEILTALPKPGGGEFGKFYSLPALNDPRIEKLPYSIRILLESAIRNCDNFQVKKEDIEKIIDWENSAPKQVEIPFKPARVLLQDFTGVPAVVDLACMRDAMNKLGSDSNKINPLVPVDLVIDHSVQVDVTRSENAVKANMEFEFQRNKERFAFLKWGSSAFHNMLVVPPGSGIVHQVNLEYLGRVVFNTNGMLYPDSVVGTDSHTTMIDGLGVAGWGVGGIEAEAAMLGQPMSMVLPGVVGFKLSGKLHNGVTATDLVLTVTQMLRKHGVVGKFVEFHGDGMGELSLADRATIANMSPEYGATMGFFPVDHVTLQYLKLTGRSDETLAMVEGYLRANKMFVDYNEPQQERVYSSYLELNLADVEPCISGPKRPHDRVPLKEMKADWHSCLDNKVGFKGFAVPKETQEKVVKFSFHGQPAELKHGSVVIAAITSCTNTSNPSVMLGAGLVAKKACELGLQVKPWVKTSLAPGSGVVTKYLLQSGLQKYLNEQGFHIVGYGCTTCIGNSGDLDESVASAITDNDIVAAAVLSGNRNFEGRVHPLTRANYLASPPLVVAYALAGTVDIDFDKEPIGTTKDGKSVYFKDIWPTTEEIAEVVQSSVLPDMFKSTYEAITKGNPTWNQLSVPASKLYSWDPNSTYIHEPPYFKDMTMDPPGAHGVKDAYCLLNFGDSITTDHISPAGSIHKDSPTAKYLLERGVERRDFNSYGSRRGNDEVMARGTFANIRLVNKLLNGEVGPKTVHVPTGEKLSVFDAAMKYKSAGHGTIILAGAEYGSGSSRDWAAKGPMLLGVKAVIAKSFERIHRSNLVGMGIIPLCFKAGEDADSLGLTGHERFSIDLPSKISEIRPGQDVTVTTDSGKSFTCTVRFDTEVELAYFDHGGILPFVIRNLIKQ

>Citrus clementina m2 (CBE71057.1)

MAPENAFKGILTSLPKPGGGEFGKFFSLPALNDPRIDRLPYSIRILLESAIRNCDNFQVTKDDVEKIIDWENTSPKQVEIPFKPARVLLQDFTGVPAAVDLACMRDAMKNLNSDPKKINPLVPVDLVVDHSVQVDVARSENAVQANMEFEFQRNQERFAFLKWGSSAFHNMPVVPPGSGIVHQVNLEYLGRVVFNTDGILYPDSVGGTDSHTTMIDGLGVAGWGVGGIEAEAAMLGQPMSMVLPGVVGFKLTGKLRDGVTATDLVLTVTQMLRKHGVVGKFVEFYGEGMGQLPLADRATIANMSPEYGATMGFFPVDHVTLQYLKLTGRSDETVSMIEEYLRANKMFVDYNKPEQERSYSSYLQLDLADVEPCISGPKRPHDRVPLKDMKADWHACLENQVGFKGFAVPKQEQDKVAKFSFHGQPAELKHGSVVIAAITSCTNTSNPSVMLGAGLVAKKACELGLEVKPWVKTSLAPGSGVVTKYLQQSGLQKYLNQQGFHIVGYGCTTCIGNSGDLDESVATAITENDIVAAAVLSGNRNFEGRVHPLTRANYLASPPLVVAYALAGTVDIDFEKEPIGTGKDGKGVYFKDIWPSNEEIAEVVQSSVLPDMFKSTYEAITKGNPMWNQLSVPTSMLYSWDPNSAYIHEPPYFKNMTMEPPGPHGVKDAYCLLNFGDSITTDHISPAGSIHKDSPAAKYLLERGVDRKDFNSYGSRRGNDEVMARGTFANIRIVNKLLNGEVGPKTVHIPTGEKLYVFDAAMRYKAAGHETIVLAGAEYGSGSSRDWAAKGPMLLGVKAVIAKSFERIHRSNLVGMGIIPLCFKPGEDADTLGLAGHERYTINLPNKVSEIRPGQDITVTTDTGKSFTCTVRFDTEVELAYFDHGGILPYVIRNLIKQ

>Oryza sativa (NP_001048898.1)

MPPLTSALLSRSSSTRIPAAAAAAAAISNPAGAAASSSSPSPPPPSSRPRPASPFASGLAGRIFGGRRAAARSSSSAAAVFERRFASAAAKNSYDEILTGLAKPGGGAEFGKYYSLPALSDPRIERLPYSIRILLESAIRNCDEFQVTGKDVEKILDWENSAPKQVEIPFKPARVLLQDFTGVPAVVDLACMRDAMSKLGSDPNKINPLVPVDLVIDHSVQVDVARSENAVQANMELEFHRNKERFGFLKWGSTAFRNMLVVPPGSGIVHQVNLEYLARVVFNNGGILYPDSVVGTDSHTTMIDGLGVAGWGVGGIEAEATMLGQPMSMVLPGVVGFKLTGKLRNGVTATDLVLTVTQMLRKHGVVGKFVEFYGGGMSELSLADRATIANMSPEYGATMGFFPVDGKTLDYLKLTGRSDDTVAMIESYLRANKMFVDYNQPEAERVYSSYLELNLEEVEPCLSGPKRPHDRVTLKNMKSDWLSCLDNDVGFKGFAVPKESQGKVAEFSFHGTPAKLKHGDVVIAAITSCTNTSNPNVMLGAALVAKKACELGLEVKPWIKTSLAPGSGVVKKYLDKSGLQKYLDQLGFHIVGYGCTTCIGNSGELDETVSAAISDNDIVAAAVLSGNRNFEGRVHALTRANYLASPPLVVAYALAGTVNIDFEKEPIGISKDGKEVYFRDIWPSTEEIAEVVKSSVLPDMFKSTYEAITKGNPMWNELSVSASTLYPWDPTSTYIHEPPYFKDMTMSPPGPRPVKDAYCLLNFGDSITTDHISPAGSIHPDSPAARYLKERGVERKDFNSYGSRRGNDEIMARGTFANIRLVNKFLKGEVGPKTIHIPSGEKLSVFDAATKYKNEGHDTIILAGAEYGSGSSRDWAAKGPMLQGVKAVIAKSFERIHRSNLAGMGIIPLCFKSGEDADTLGLTGHERFTVHLPANVSEIKPGQDVTVTTDNGKSFTCTLRFDTEVELAYYDNGGILPYVIRKIAEQ

>Zea mays (NP_001147431.1)

MANPTATKHAFKRILTSLLKPGGGEYGKFFSLPALNDPRIDKLPYSIRVLLESAIRHCDNFQVTESDVEKIIDWENTSPKLAEIPFKPARCILMDNTGVPAVVDLAAMRDMMPKLGCDPYKINPLIPVDAVIDHAVRVDVAGTYDALDRNEELEFQRNKERFAFLKWASNAFHNMQVFPPGSGTVHQVNLEYLARVVFNEDGILYFDSVVGTDSHTTMINSLGVAGWGVGGIEAVVAMLGQPMGMVLPGVVGFKLSGKLRDGVTTTDIVLTMTQMLRKHGAIGKFVEFYGVGVGELSLPARATIANMSPEYGATMGFFPVDQVALDYLKLTGRSDETVSMIEAYLRANKMFVDKHEPETERVFSSHLELDLSEVEPCVSGPKRPHDRVPLKEMKSDWHACLDNEVGFKGYAVPKEQQGKVVKFDFHGRPAEIKHGSVVLAAICSSTNTSNPSVMIGAGLVAKKACELGLEVKPWVKTSLTPGSVVATEYLKHSGLQDYLNQQGFHVAAHGCATCVGNSGDLDGSVSAAITENDMVAAAVLSANRNFEGRVNPLTRANYLASPPLVVAYALAGTVDIDFEKEPIGVGKGGKEVFLRDIWPSNQEIDEVVESSVQTHLFKKVYDSIMERNPRWNQLPVPKEALYPWEDRSTYIRKPTYLEGMSMTPPAAPPTVTEAYCLLNLGDSITTDHISYSGKIPEGTPAAKYLLEYGVEPKNFSSYGGRRGNNEVVMRGAFANMRIVNKLLDGKAGPWTIHVPTGEKLYVYDAAMKYKSEGHDMVIIAGSEYGSGSSRDSAAKGPMLLGVKSVIAKSFERIHRSNLVGMGIIPLCFKAGEDADSLGLTGRERYTIHLPTSTAELSPGQDVTVTTHDGRSFTCTLRLDTQLEVTYFNHGGILPYMVRNLAAQMK

>Brachypodium distachyon (XP_003558862.1)

MPPLASSLLSRSAAGPVSSARVTAAAAVAAVSKPVAASEPAASPPVTPTPTAGTSSSPRSPFASGLAGRLFGGRRAAARSSSSAAAVFERRFASAATKNSYDEILTSLAKPGGGADFGKYYSLPRLADPRIDRLPYSIRILLESAIRNCDEFQVTGKDVEKILDWENSATKQVEIPFKPARVLLQDFTGVPAVVDLACMRDAMSKLGSDPNKINPLVPVDLVIDHSVQVDVARSQNAVQANMELEFSRNKERFGFLKWGSTAFNNMLVVPPGSGIVHQVNLEYLARVVFNNGGILYPDSVVGTDSHTTMIDGLGVAGWGVGGIEAEATMLGQPMSMVLPGVVGFKLTGKLKNGVTATDLVLTVTQMLRKHGVVGKFVEFYGGGMSELSLADRATIANMSPEYGATMGFFPVDAKTLDYLKLTGRSDDTVAMIETYLRANNMFVDYNQVQAERVYSSYLELNLEEVEPCLSGPKRPHDRVTLKNMKSDWLSCLDNDVGFKGFAVPKESQGKVADFSFHGTPAKIKHGDVVIAAITSCTNTSNPNVMLGAALVAKKACELGLEVKPWIKTSLAPGSGVVKKYLDKSGLQKYLDQLGFNIVGYGCTTCIGNSGDLDESVAAAISENDVVAAAVLSGNRNFEGRVHALTRANYLASPPLVVAYALAGTVNIDFEKEPVGISKDGKEVYFRDIWPTTEEISEVVKSSVLPDMFKSTYEAITKGNPMWNELPVSASTLYPWDSSSTYIHEPPYFKDMTMTPPGARPVKDAYCLLNFGDSITTDHISPAGSIHPESPAAKFLSERNVERKDFNSYGSRRGNDEIMARGTFANIRLVNKFLKGEVGPKTIHIPSGEKLAVFDAAMKYKNEGHDTIILAGAEYGSGSSRDWAAKGPMLQGVKAVIAKSFERIHRSNLAGMGIIPLCFKAGEDADTLGLTGHERFTIQLPTNVSDIKPGQDVTVTTDAGKSFTCTLRFDTEVELAYYTNGGILPYVIRKIAAEP

>Sorghum bicolor mt1 (XM_002465856.1)

MPPLTSSLLSRSTTGGSSARGVAAAAAISRPAADAAPSSSSPPARSTPTPRPRPSTASPFASGLAGRLFGGHRAAARSASSATAVFERRFASAATRNTYDEILTGLKRPGAGDEFGKYYSLPALSDPRIDRLPYSIRILLESAIRNCDDFQVTGNDVEKILDWEKSAPKLVEIPFKPARVLLQDFTGVPAVVDLACMRDAMSKLGSDPNKINPLVPVDLVIDHSVQVDVARSANAAQANMELEFHRNKERFGFLKWGSSAFRNMLVVPPGSGIVHQVNLEYLARVVFNNGGILYPDSVVGTDSHTTMIDGLGVAGWGVGGIEAEAAMLGQPMSMVLPGVVGFKLSGKLKNGVTATDLVLTVTQMLRKHGVVGKFVEFYGQGMSELSLADRATIANMSPEYGATMGFFPVDAKTLDYLKLTGRSDDTVAMVESYLRANKMFVDHSQVEAERVYSSYLELNLEEVEPCLSGPKRPHDRVTLKNMKSDWLSCLDSDVGFKGFAVPKESQGKVAEFSFHGTPAKIKHGDVVIAAITSCTNTSNPNVMLGAALVAKKACELGLEVKPWIKTSLAPGSGVVKKYLDKSGLQKYLDQLGFHIVGYGCTTCIGNSGELDESVSAAITENDVVAAAVLSGNRNFEGRVHPLTRANYLASPPLVVAYALAGTVNIDFEKEPIGISKDGKEVYFRDVWPSTEEIAEVVKSSVLPDMFKSTYESITKGNPMWNELSVSTSTLYPWDPTSTYIHEPPYFKDMTMTPPGPRPVKDAYCLLNFGDSITTDHISPAGNIHPDSPAAKYLKERGVERKDFNSYGSRRGNDEIMARGTFANIRLVNKFLKGEVGPKTIHVPSGEKLAVFDAAMKYKNEGHDTIILAGAEYGSGSSRDWAAKGPMLQGVKAVIAKSFERIHRSNLAGMGIIPLCFKAGEDADTLGLTGHERYTVHLPTNVSEIKPGQDVTVTTDNGKSFTCTLRFDTEVELAYYDHGGILPYVTRKIAEQ

>Sorghum bicolor mt2 (XM_002445129.1)

MYKAANARLLLRSLSSNSAASASVPVNNPRLAAASSSSSAARLALSLPCGAWVGRSSGLVRAAGWSGTRPRFAGARAQIGAAAVPAVERFQRRMATQATEHAFKDILTSLPKPGGGEYGKFYSLPALNDPRIDKLPYSIRILLESAIRNCDNFQVTKNDVEKIIDWENTSPKLAEIPFKPARVLLQDFTGVPAVVDLAAMRDAMAKLGSDANKINPLVPVDLVIDHSVQVDVARSQNAVQANMELEFSRNKERFGFLKWGSSAFQNMLVVPPGSGIVHQVNLEYLGRVVFNTDGILYPDSVVGTDSHTTMIDGLGVAGWGVGGIEAEATMLGQPMSMVLPGVVGFKLTGKLRSGVTATDLVLTVTQMLRKHGVVGKFVEFYGEGMGKLSLADRATIANMSPEYGATMGFFPVDHVTLDYLKLTGRSDETVSMIEAYLRANKMFVDYNEPPTERIYSSYLELDLDEVEPSMSGPKRPHDRVPLKEMKSDWHACLDNKVGFKGFAVPKEQQDKVVKFDFHGQPAEMKHGSVVIAAITSCTNTSNPSVMLGAGLVAKKACELGLEVKPWVKTSLAPGSGVVTKYLLQSGLQEYLNQQGFHIVGYGCTTCIGNSGDLDESVSAAITENDVVAAAVLSGNRNFEGRVHPLTRANYLASPPLVVAYALAGTVDIDFEKEPIGLGKDGKEVYFRDIWPSTEEIAQVVQSSVLPDMFKGTYEAITKGNPMWNQLTVPEASLYSWDSKSTYIHEPPYFKDMTMSPPGPSAVKDAYCLLNFGDSITTDHISPAGSIHKDSPAAKYLMERGVDRKDFNSYGSRRGNDEIMARGTFANIRIVNKFLNGEVGPKTIHVPTGEKLYVFDAAMRYKSEGHATIILAGAEYGSGSSRDWAAKGPMLLGVKAVIAKSFERIHRSNLVGMGIIPLCFKAGEDADSLGLTGHERYSIDLPANLSEIRPGQDVTVITDNGKSFTCTLRFDTEVELAYFNHGGILPYVIRNLAGAQN

>Sorghum bicolor mt3 (XM_002460720.1)

MYRAAASPLRHSLRRLSSSYSAATPLAGAARFLAPSSEGRLSALTSSRLLGSAPCRCCSGAGIAERPASAWRGLATMADGNSRFGHVLTSLPKPGGGEYGKYYSLPALNDPRIERLPYSIRYLLESAIRNCDGFQITEKDVENIIDWENTAPKLVEIPFKPARVLLQDFTGVPAIVDLASMRDAMAQLGDDPGKIDPMIPVDLVIDHSVQADVVKSENALQANMQREFDRNKERFAFLRWGSTAFNNMLIVPPGSGIVHQVNLEYLGRVVFNTDGILYLDSVLGTDSHTTMIDGMGVAGWGVGGIEAEATMLGQPMSMVLPSVVGFKLTGKLRDGVTATDLVLTVTHILRKHGVVGKFVEFYGEGMSELAVANRATIANMSPEYGATMGFFPVDHVTLGYLKLTGRSDEKVDMVEAYLRANKMFVDYNETQTERVYSSYLELDLADVEPCVSGPKRPHDRVALKDMKADWRACLRNKVGFKGFGIPKEQQDKLVKFTFHGQPAEIRHGSIVIAAITSCTNTSNPSVMLGAGLVAKKACELGLEVNPWIKTSLAPGSGAVTKYLLKSGLQKYLDHLGFNLIGYGCTTCIGNSGELDEDVAKAVTDNDIIAAAVLSGNRNFEGRIHALVRANYLASPPLVVAYALAGTVDIDFETEPIGKGKNGKDVYFKDIWPSNEEIAEVEQSSVLPDMFRSTYEAITQGNPMWNQLSVPKAKRFPWDPSSTYIHDPPFFKDITPTPPGPRSIENAYCLLKFGDSITTDHISPAGSIPRDSPAGMYLLERGVQPKDFNSYGSRRGNDEVMARGTFANIRIVNRLLNGEVGPKTIHVPTSDKLFVFDAAMRYKADGHHTIVLAGEEYGSGSSRDWAAKGPMLLGVKAVIAKSFERIHRSNLVGMGVLPLCFKPGEDADSLGLTGHERYTIRLPTNVSEIQPGQDVQVVTDTGKSFTCKLRIDTLVELAYFDHGGILHYVLRNLVKQQQQQ

>Ricinus communis mt1 (XP_002524184.1)

MYIMTASSTASSLLRASRARLLSSSSSSVISRTTPLPPPLPKFSVTNRSLSFSAAVRSLRCSVPRWSHGVDWRSPVSLRSQIRTASPVIERFQRKISTMAAEHPFKGIVTPLPKPGGGEFGKFYSLPALNDPRIDKLPYSIRILLESAIRNCDNFQVTKQDVEKIIDWENSAPKQVEIPFKPARVLLQDFTGVPAVVDLASMRDAMNKLGGDSNKINPLVPVDLVIDHSVQVDVTRSENAVQANMELEFQRNKERFAFLKWGSNAFQNMLVVPPGSGIVHQVNLEYLGRVVFNKDGILYPDSVVGTDSHTTMIDGLGVAGWGVGGIEAEAAMLGQPMSMVLPGVVGFKLSGKLHNGVTATDLVLTVTQMLRKHGVVGKFVEFYGEGMGELSLADRATIANMSPEYGATMGFFPVDHVTLQYLKLTGRSDETISMIESYLRANKMFVDYNEPQQERVYSSYLQLDLGEVEPCISGPKRPHDRVPLKEMKADWHSCLDNKVGFKGFAIPKEVQEKVAKFSFHGQPAELKHGSVVIAAITSCTNTSNPSVMLGAGLVAKKACELGLQVKPWIKTSLAPGSGVVTKYLLQSGLQKYLNQQGFHIVGYGCTTCIGNSGDLDESVASAISENDIVAAAVLSGNRNFEGRVHALTRANYLASPPLVVAYALAGTVDIDFDKEPIGTGKDGKDVYFRDIWPSTEEIAEAVQSSVLPHMFRSTYEAITKGNPMWNQLTVPATTSYSWDPNSTYIHDPPYFKSMTLNPPGAHGVKDAYCLLNFGDSITTDHISPAGSIHKDSPAAKFLLERGVDRQDFNSYGSRRGNDEVMARGTFANIRLVNKLLNGEVGPKTVHIPTGEKLYVFDAASRYMAAGHDTIVLAGAEYGSGSSRDWAAKGPMLLGVKAVIAKSFERIHRSNLVGMGIIPLCFKPGQDADTLGLSGHERYTIDLPSNISEIKPGQDVTVTTDNGKSFTCTARFDTEVELEYFNHGGILPYVIRNLMKTE

>Ricinus communis mt2 (XP_002532564.1)

MYLPTSPYSSSSALLRSSHLVSRISSLSPKFPSPTSFRSLSPSSFRYQNSRSFSFSSALRSLPCAVSRWSHRVDWRSPISLRAQAPAASPVIERKLSTIASEHPXXXKPGGGEFGKFYSLTALDDPRIDRLPYSIKILLESAIRNCDNFQVAKEHVEKIIDWENTSLKQVEIPFKPARVLLQDFTGVPAVVDLACMRDAIKNLGSDPKKINPLVPVDLVVDHSVQVDVTRSENAVQANMENEFQRNKERFAFLKWGASAFQNMLVVPPGSGIVHQVNLEYLGRVVFNTDGLLYPDSLVGTDSHTTMIDGLGVAGWGVGGIEAEATMLGQPMSMVLPGVVGFKLSGKLRDGVTATDLVLTVTQMLRKHGVVGKFVEFYGDGMDELSLADRATIANMSPEYGATMGFFPVDHATLQYLKLTGRSDETVAMIEAYLRANKMFVDYNEPQEEQVYTSYLQLDLADVEPCVSGPKRPHDRVPLKEMKADWQKCLDNRAGFKGFGVPKEEQDKVAKFSFHGQPAELKHGSVVIAAITSCTNTSNPSVMLGAGLVAKKACELGLQVKPWIKTSLAPGSGVVTKYLLQSGLQRYLNELGFHIVGYGCTTCIGNSGELDPSVASAISDNDIIAAAVLSGNRNFEGRVHPLTRANYLASPPLVVAYALAGTVNIDFEEEPIGTNKDGKNIYFKDVWPTNDEIAEVVQSNVLPSMFKSTYEAITKGNPMWSQLSIPTSVLYSWDPKSTYIHEPPYFEDMTMDPPGPHGVKDAYCLLTFGDSITTDHISPAGSIHKDSPAAKYLLERGVEPKDFNSYGSRRGNDEVMARGTFANIRLVNKLLNGEVGPKTIHIPTGEKLHVFDAAMRYKEAGENTIVLAGAEYGSGSSRDWAAKGPMLLGVKAVIAKSFERIHRGNLVGMGIIPLCFKPGEDADTLGLTGHERYTIDLPDKISEIRPGQEVGVKTDTGKSFACRVRFDTEVELAYFDHGGILPYVIRNLSKE

>Populus trichocarpa mt1 (XM_002301587.1)

MAHEHPFKGIFTSLPKPGGGEFGKFYSLPALNDPRIEKLPYSIRILLESAIRNCDNFQVTKGDVEKIIDWENTAPKLVEIPFKPARVLLQDFTGVPVVVDLASMRDAMAQLGGDSNKINPLVPVDLVIDHSVQVDVARSENAVQANMELEFHRNKERFAFLKWGSTAFQNMLVVPPGSGIVHQVNLEYLGRVVFNTDGVLYPDSVVGTDSHTTMIDGLGVAGWGVGGIEAEAAMLGQPMSMVLPGVVGFKLNGKLHNGVTATDLVLTVTQMLRKHGVVGKFVEFYGDGMSKLSLADRATIANMSPEYGATMGFFPVDHVTLQYLKLTGRSDETVAMIEAYLRANKMFVDYNEPQPERVYSAYLQLDLADVEPCISGPKRPHDRVPLKEMKADWHACLNNKVGFKGFAVPKEAQDKVAKFSFHGQPAELKHGSVVIAAITSCTNTSNPSVMLGAGLVAKKACELGLKVKPWIKTSLAPGSGVVTKYLLQSGLQKYLNEQGFNIVGYGCTTCIGNSGDLDESVGAVITENDILAAAVLSGNRNFEGRVHALTRANYLASPPLVVAYALAGTVNIDFDKEPIGTGKDGKSVYFRDIWPTAEEIAEVVQSSVLPAMFKSTYESITKGNPMWNQLSVPASTSYSWDPSSTYIHEPPYFKNMTMNPPGAHGVKDAYCLLNFGDSITTDHISPAGSIHKDSPTAKYLLEHGVDRKDFNSYGSRRGNDEVMARGTFANIRLVNKFLNGEVGPKTVHIPTGEKLSVYDAAMRYKNAGLDTIVLAGAEYGSGSSRDWAAKGPMLLGVKAVIAKSFERIHRSNLVGMGIIPLCFKAGQDADTLGLTGHERYTIDLPSNISEIRPGQDVTVTTDNGKSFTCTARFDTAVELEYFNHGGILPYAIRSLMKQ

>Populus trichocarpa mt2 (XM_002321090.1)

MAPEHPFKGIFTSLPKPGGGEFGKFYSLPALNDPRIDKLPYSIRILLESAIRNCDNFQVTKDDVEKIIDWENTSPKQVEIPFKPARVLLQDFTGVPAVVDLASMRDAMGQLGGDSNKINPLVPVDLVIDHSVQVDVARSENAVQANMELEFKRNKERFAFLKWGSTAFQNMLVVPPGSGIVHQVNLEYLGRVVFNTDGVLYPDSVVGTDSHTTMIDGLGVAGWGVGGIEAEATMLGQPMSMVLPGVVGFKLNGKLRNGVTATDLVLTVTQMLRKHGVVGKFVEFYGDGMGELSLADRATIANMSPEYGATMGFFPVDHVTLQYLKLTGRSDETVARIEAYLRANKMFVDYDEPQAERVYSSYLQLDLADVEPCVSGPKRPHDRVPLREMKADWHSCLSNKVGFKGFAVPKEAQDKVAKFSFHGQPAELKHGSVVIAAITSCTNTSNPSVMLGAALVAKKACELGLKVKPWIKTSLAPGSGVVTKYLQKSGLQKYFNEQGFHIVGYGCTTCIGNSGDLDESVASAISENDILAAAVLSGNRNFEGRVHPLTRANYLASPPLVVAYALAGTVDIDFDKEPIGTGKDGKSVYFKDIWPTTEEVAEVVQSSVLPDMFKSTYEAITKGNPMWNELTVPAATSYAWDPNSTYIHEPPYFKNMTLNPPGAHGVKDAYCLLNFGDSITTDHISPAGSIHRDSPAAKFLLERGVDPKDFNSYGSRRGNDEVMARGTFANIRLVNKLLNGEVGPKTVHIPTGEKLYVFDAAMRYKSAGYDTIVLAGAEYGSGSSRDWAAKGPMLLGVKAVIAKSFERIHRSNLVGMGIIPLCFKDGQDADTLGLTGHERYSIDLPSNIGEIRPGQDVTVTTDNGKSFICTVRFDTAVELEYFNHGGILPYAIRNLMKQ

>Vitis vinifera mt1(XM_002279224.2)

MYITASSLSSSLLRASRAHFSSSLSRVSLSRAISSNPLSSSSLACRSLRFSSSAFRSLRSVNFRPPMSLRAQIGAAVPVVEQFQRRIATMAPENAFKGILTGLPKASGGEFGKYYSLPALNDPRVDKLPYSIRILLESAIRNCDNFQVTKEDVEKIIDWENTSPKQVEIPFKPARVILQDFTGVPAVVDLACMRDAMNKLGSDSNKINPLVPVDLVVDHSVQVDVARSENAVQANMELEFQRNKERFAFLKWGSTAFHNMLVVPPGSGIVHQVNLEYLGRVVFNADGLLYPDSVVGTDSHTTMIDGLGVAGWGVGGIEAEAAMLGQPMSMVLPGVVGFKLTGKLRNGVTATDLVLTVTQMLRKHGVVGKFVEFYGDGMAELSLADRATIANMSPEYGATMGFFPVDRVTLQYLKLTGRSDETVALIEAYLRANKMFVDHNEPQQERAYSSYLELDLVNVEPCVSGPKRPHDRVTLKEMKVDWHSCLDNKVGFKGFAVPKEAQDKVAKFSFHGQPAELKHGSVVIAAITSCTNTSNPSVMLGAALVAKKACELGLEVKPWIKTSLAPGSGVVTKYLLQSGLQKYLNQQGFHIVGYGCTTCIGNSGEIDESVASAITENDIVAAAVLSGNRNFEGRVHPLTRANYLASPPLVVAYALAGTVDIDFEKEPIGTGKDGKSVYFKDIWPSTEEIAEVVQSSVLPNMFKSTYEAITKGNSMWNDLSVPANTLYSWDAKSTYIHEPPYFKNMTMDPPGAHGVKDAYCLLNFGDSITTDHISPAGSIHKDSPAAKYLLERGVDRKDFNSYGSRRGNDEVMARGTFANIRLVNKLLNGEVGPKTIHVPTGEKLSVFDAAMKYKTANQGTIILAGAEYGSGSSRDWAAKGPMLLGVKAVIAKSFERIHRSNLVGMGIIPLCFKPGEDADTLGLTGHERYTIDLPSNIDEIRPGQDITVTTNTGKSFICTARFDTEVELAYFNHGGILPYVIRNLIKQ

>Vitis vinifera mt2 (XM_002278102.2)

MYMATSPYSSLALLRASRVRFAPSISRVSLSSSSSSSSPHPPCPSRIPASASSSSLPFSTISGGYRSLGFLSAFRSRRWSHGVDWRSPVSLRAQIRAAAPVIERFERKMATIASEHPFKGILTSVPKPGGGEFGKFYSLPALNDPRIDKLPYSIRILLESAIRNCDNFQVTKDDVEKIIDWENTSPKQVEIPFKPARVLLQDFTGVPAVVDLACMRDAMNNLGSDSNKINPLVPVDLVIDHSVQVDVTRSENAVQANMDLEFQRNKERFSFLKWGSTAFRNMLVVPPGSGIVHQVNLEYLGRVVFNNDGILYPDSVVGTDSHTTMIDGLGVAGWGVGGIEAEAAMLGQPMSMVLPGVVGFKLSGKLCSGVTATDLVLTVTQMLRKHGVVGKFVEFYGEGMGELSLADRATIANMSPEYGATMGFFPVDHVTLQYLKLTGRSDETVAMIEAYLRANRMFVDYNEPQVERFYSSYLQLNLEDVEPCMSGPKRPHDRVPLKEMKTDWKACLDNKVGFKGFAVPKEAQDKVAKFSFHGQPAELKHGSVVIAAITSCTNTSNPSVMLGAGLVAKKASELGLEVKPWIKTSLAPGSGVVTKYLLQSGLQKYLNQQGFHIVGYGCTTCIGNSGDLDESVASAISENDIIAAAVLSGNRNFEGRVHALTRANYLASPPLVVAYALAGTVDIDFEKEPIGTGKDGKDVYFKDIWPTSEEIAEVVQSSVLPEMFKSTYEAITKGNPIWNQLSVHSSSLYSWDPNSTYIHEPPYFKNMTMNPPGPHGVKDAYCLLNFGDSITTDHISPAGSIHKDSPAAKYLIERGVAPKDFNSYGSRRGNDEVMARGTFANIRIVNKLLNGEVGPKTIHIPTGEKLYVFDAAMRYKADGHDTIVLAGAEYGSGSSRDWAAKGPMLQGVKAVIAKSFERIHRSNLVGMGIIPLCFKAGEDADTLGLTGHERYNIDLPSKISEIRPGQDVTVTTDNGKSFTCTVRFDTEVELEYFNHGGILPYAIRNLINQ

>Caenorhabditis elegans (NP_741235.1)

MNSLLRLSHLAGPAHYRALHSSSSIWSKVAISKFEPKSYLPYEKLSQTVKIVKDRLKRPLTLSEKILYGHLDQPKTQDIERGVSYLRLRPDRVAMQDATAQMAMLQFISSGLPKTAVPSTIHCDHLIEAQKGGAQDLARAKDLNKEVFNFLATAGSKYGVGFWKPGSGIIHQIILENYAFPGLLLIGTDSHTPNGGGLGGLCIGVGGADAVDVMADIPWELKCPKVIGIKLTGKLNGWTSAKDVILKVADILTVKGGTGAIVEYFGPGVDSISATGMGTICNMGAEIGATTSVFPYNESMYKYLEATGRKEIAEEARKYKDLLTADDGANYDQIIEINLDTLTPHVNGPFTPDLASSIDKLGENAKKNGWPLDVKVSLIGSCTNSSYEDMTRAASIAKQALDKGLKAKTIFTITPGSEQVRATIERDGLSKIFADFGGMVLANACGPCIGQWDRQDVKKGEKNTIVTSYNRNFTGRNDANPATHGFVTSPDITTAMAISGRLDFNPLTDELTAADGSKFKLQAPTGLDLPPKGYDPGEDTFQAPSGSGQVDVSPSSDRLQLLSPFDKWDGKDLEDMKILIKVTGKCTTDHISAAGPWLKYRGHLDNISNNLFLTAINADNGEMNKVKNQVTGEYGAVPATARKYKADGVRWVAIGDENYGEGSSREHAALEPRHLGGRAIIVKSFARIHETNLKKQGMLPLTFANPADYDKIDPSDNVSIVGLSSFAPGKPLTAIFKKTNGSKVEVTLNHTFNEQQIEWFKAGSALNRMKEVFAKSK

>Drosophila melanogaster (NP_524708.1)

MAARLMNAQAQVCRLGKHVASEATVVRQFHASCYTASKVALSKFDSDVYLPYEKLNKRLEVVRGRLNRPLTLSEKVLYSHLDDPANQDIVRGTSYLRLRPDRVAMQDATAQMALLQFISSGLKKVAVPSTVHCDHLIEAQIGGPKDLARAKDLNKEVYDFLASTCAKYGLGFWKPGSGIIHQIILENYAFPGLLMIGTDSHTPNGGGLGGLCIGVGGADAVDVMADIPWELKCPKVIGVNLTGKISGWTSPKDVILKVADILTVKGGTGAIIEYHGKGVDSISCTGMATICNMGAEIGATTSLFPFNQRMADYLKSTGRAGIASEAQKYQAKILSADKNCEYDELIEINLDTLEPHVNGPFTPDLGHPISKLGENSKKNGYPMDIRVGLIGSCTNSSYEDMGRCASIAKDAMSHGLKSKIPFNVTPGSEQIRATIERDGISEVFDKFGGTVLANACGPCIGQWDRKDVKKGDKNTIVTSYNRNFTGRNDANPATHCFVTSPELVTALSIAGRLDFNPLTDELTGADGKKFKLKAPFGDELPAKGFDPGQDTYTAPPPSGENVKVAVDPKSTRLQLLEPFDKWNGQDLTDLTVLIKVKGKCTTDHISAAGPWLKYRGHLDNISNNMFIGATNYENNEMNNIKNQRNGSWGGVPDVARDYKANGIKWVAVGDENYGEGSSREHAALEPRHLGGRAIIVKSFARIHETNLKKQGLLPLTFANPADYDKIQPTSKISLLNLKSLAPGKPVDAEIKNGDKVERIKLNHTLNDLQIGWFKAGSALNRMKELAQ

>Gallus gallus (NP_989519.1)

MAPYCVWLARLRHALNGGIRRYHVASVLCQRAKVAMSHFEPNEYINYEKLEKNINIVRKRLDRPLTLSEKIVYGHLDDPAKQEIERGKTYLRLRPDRVAMQDATAQMAMLQFISSGLPKVAVPSTIHCDHLIEAQLGGEKDLRRAKDINQEVYNFLSTAGAKYGVGFWKPGSGIIHQIILENYSYPGVMLIGTDSHTPNGGGLGGICIGVGGADAVDVMAGIPWELKCPKVIGVKLTGKLSGWSSPKDVILKVAGILTVKGGTGAIIEYHGPGVDSISCTGMATICNMGAEIGATTSVFPYNTRMKKYLGKTGRADIAALADEFQQYLVPDAGCQYDQVIEINLSELKPHINGPFTPDLAHPVSDVGAVAEKEGWPVDIRVGLIGSCTNSSYEDMGRSAAVAKQALAHGLKCKSKFTITPGSEQIRATIERDGYAQILRDVGGLILANACGPCIGQWDRKDIKKGEKNTIVTSYNRNFTGRNDANPETHAFVTSPEIVTALSIAGTLKFNPETDFLTGADGKKFKLEAPDADELPRLDFDPGQDTYQYPPKDGSGQHVDVSPTSQRLQLLEPFDKWDGKDLEDMLILIKVKGKCTTDHISAAGPWLKFRGHLDNISNNLLIGAINIENGKANSVRNALTQEFGPVPDTARYYKKMGVKWAVIGDENYGEGSSREHAALEPRHLGGRVIITKSFARIHETNLKKQGLLPLTFADPADYNKIHPVDKLSIVGLADFAPGKPLKCIIKHPNGSQETIMLNHTFNESQIEWFQAGSALNRMKELQQKSS

>Felis catus (XP_003989381.1)

MAPYSLLVTRLQKALGVRQYHVASVLCQRAKVAMSHFEPNEYIRYDLLEKNIDIVRKRLNRPLTLSEKIVYGHLDDPAKQEIERGKTYLRLRPDRVAMQDATAQMAMLQFISSGLPKVAVPSTIHCDHLIEAQLGGEKDLRRAKDINQEVYNFLATAGAKYGVGFWRPGSGIIHQIILENYAYPGVLLIGTDSHTPNGGGLGGICIGVGGADAVDVMAGIPWELKCPKVIGVKLTGSLSGWTSPKDVILKVAGILTVKGGTGAIVEYHGPGVDSISCTGMATICNMGAEIGATTSVFPYNHRMKKYLSKTGREDIAKLADEYKDHLVPDPGCHYDQLIEINLNELKPHINGPFTPDLAHPVAEVGTVAEKEGWPLDIRVGLIGSCTNSSYEDMGRSAAVAKQALAHGLKCKSQFTITPGSEQIRATIERDGYAQILREVGGIVLANACGPCIGQWDRKDIKKGEKNTIVTSYNRNFTGRNDANPETHAFVTSPEIVTALAIAGTLKFNPETDFLTGKDGKKFKLEAPDADELPRAEFDPGQDTYQHPPKDSSGQRVDVSPTSQRLQLLEPFDKWDGKDLEDLQILIKVKGKCTTDHISAAGPWLKFRGHLDNISNNLLIGAINVENGKANSVRNAVTQEFGPVPDTARYYKKHGIRWVVIGDENYGEGSSREHAALEPRHLGGRAIITKSFARIHETNLKKQGLLPLTFADPADYNKIHPVDKLTIQGLKDFAPGKPLKCIIKHPNGTQETILLNHTFNETQIEWFRAGSALNRMKELQQQ

>Homo sapiens (NM_001098.2)

MAPYSLLVTRLQKALGVRQYHVASVLCQRAKVAMSHFEPNEYIHYDLLEKNINIVRKRLNRPLTLSEKIVYGHLDDPASQEIERGKSYLRLRPDRVAMQDATAQMAMLQFISSGLSKVAVPSTIHCDHLIEAQVGGEKDLRRAKDINQEVYNFLATAGAKYGVGFWKPGSGIIHQIILENYAYPGVLLIGTDSHTPNGGGLGGICIGVGGADAVDVMAGIPWELKCPKVIGVKLTGSLSGWSSPKDVILKVAGILTVKGGTGAIVEYHGPGVDSISCTGMATICNMGAEIGATTSVFPYNHRMKKYLSKTGREDIANLADEFKDHLVPDPGCHYDQLIEINLSELKPHINGPFTPDLAHPVAEVGKVAEKEGWPLDIRVGLIGSCTNSSYEDMGRSAAVAKQALAHGLKCKSQFTITPGSEQIRATIERDGYAQILRDLGGIVLANACGPCIGQWDRKDIKKGEKNTIVTSYNRNFTGRNDANPETHAFVTSPEIVTALAIAGTLKFNPETDYLTGTDGKKFRLEAPDADELPKGEFDPGQDTYQHPPKDSSGQHVDVSPTSQRLQLLEPFDKWDGKDLEDLQILIKVKGKCTTDHISAAGPWLKFRGHLDNISNNLLIGAINIENGKANSVRNAVTQEFGPVPDTARYYKKHGIRWVVIGDENYGEGSSREHAALEPRHLGGRAIITKSFARIHETNLKKQGLLPLTFADPADYNKIHPVDKLTIQGLKDFTPGKPLKCIIKHPNGTQETILLNHTFNETQIEWFRAGSALNRMKELQQ

>Mus musculus (NM_080633.2)

MAPYSLLVTRLQKALGVRQYHVASVLCQRAKVAMSHFEPSEYIRYDLLEKNINIVRKRLNRPLTLSEKIVYGHLDDPANQEIERGKTYLRLRPDRVAMQDATAQMAMLQFISSGLPKVAVPSTIHCDHLIEAQVGGEKDLRRAKDINQEVYNFLATAGAKYGVGFWRPGSGIIHQIILENYAYPGVLLIGTDSHTPNGGGLGGICIGVGGADAVDVMAGIPWELKCPKVIGVKLTGSLSGWTSPKDVILKVAGILTVKGGTGAIVEYHGPGVDSISCTGMATICNMGAEIGATTSVFPYNHRMKKYLSKTGRTDIANLAEEFKDHLVPDPGCQYDQVIEINLNELKPHINGPFTPDLAHPVADVGTVAEKEGWPLDIRVGLIGSCTNSSYEDMGRSAAVAKQALAHGLKCKSQFTITPGSEQIRATIERDGYAQILRDVGGIVLANACGPCIGQWDRKDIKKGEKNTIVTSYNRNFTGRNDANPETHAFVTSPEIVTALAIAGTLKFNPETDFLTGKDGKKFKLEAPDADELPRSDFDPGQDTYQHPPKDSSGQRVDVSPTSQRLQLLEPFDKWDGKDLEDLQILIKVKGKCTTDHISAAGPWLKFRGHLDNISNNLLIGAINIENGKANSVRNAVTQEFGPVPDTARYYKKHGIRWVVIGDENYGEGSSREHAALEPRHLGGRAIITKSFARIHETNLKKQGLLPLTFADPSDYNKIHPVDKLTIQGLKDFAPGKPLKCVIKHPNGTQETILLNHTFNETQIEWFRAGSALNRMKELQQ

>Saccharomyces cerevisiae mt1 (AAA34389.1)

MLSARSAIKRPIVRGLATVSNLTRDSKVNQNLLEDHSFINYKQNVETLDIVRKRLNRPFTYAEKILYGHLDDPHGQDIQRGVSYLKLRPDRVACQDATAQMAILQFMSAGLPQVAKPVTVHCDHLIQAQVGGEKDLKRAIDLNKEVYDFLASATAKYNMGFWKPGSGIIHQIVLENYAFPGALIIGTDSHTPNAGGLGQLAIGVGGADAVDVMAGRPWELKAPKILGVKLTGKMNGWTSPKDIILKLAGITTVKGGTGKIVEYFGDGVDTFSATGMGTICNMGAEIGATTSVFPFNKSMIEYLEATGRGKIADFAKLYHKDLLSADKDAEYDEVVEIDLNTLEPYINGPFTPDLATPVSKMKEVAVANNWPLDVRVGLIGSCTNSSYEDMSRSASIVKDAAAHGLKSKTIFTVTPGSEQIRATIERDGQLETFKEFGGIVLANACGPCIGQWDRRDIKKGDKNTIVSSYNRNFTSRNDGNPQTHAFVASPELVTAFAIAGDLRFNPLTDKLKDKDGNEFMLKPPHGRWFASKEVMMLVRTLTKLHLQTVATVEVKVSPTSDRLQLLKPFKPWDGKDAKDMPILIKAVGKTTTDHISMAGPWLKYRGHLENISNNYMIGAINAENKKANCVKNVYTGEYKGVPDTARDYRDQGIKWVVIGDENFGEGSSREHAALEPRFLGGFAIITKSFARIHETNLKKQGLLPLNFKNPADYDKINPDDRIDILGLAELAPGKPVTMRVHPKNGKPWDAVLTHTFNDEQIEWFKYGSALNKIKADEKK

>Saccharomyces cerevisiae mt2 (NP_012335.1)

MLSSANRFYIKRHLATHANMFPSVSKNFQTKVPPYAKLLTNLDKIKQITNNAPLTLAEKILYSHLCDPEESITSSDLSTIRGNKYLKLNPDRVAMQDASAQMALLQFMTTGLNQTSVPASIHCDHLIVGKDGETKDLPSSIATNQEVFDFLESCAKRYGIQFWGPGSGIIHQIVLENFSAPGLMMLGTDSHTPNAGGLGAIAIGVGGADAVDALTGTPWELKAPKILGVKLTGKLNGWSTPKDVITKLAGLLTVRGGTGYIVEYFGEGVSTLSCTGMATICNMGAEIGATTSTFPYQEAHKRYLQATNRAEVAEAADVALNKFNFLRADKDAQYDKVIEIDLSAIEPHVNGPFTPDLSTPISQYAEKSLKENWPQKVSAGLIGSCTNSSYQDMSRVVDLVKQASKAGLKPRIPFFVTPGSEQIRATLERDGIIDIFQENGAKVLANACGPCIGQWNREDVSKTSKETNTIFTSFNRNFRARNDGNRNTMNFLTSPEIVTAMSYSGDAQFNPLTDSIKLPNGKDFKFQPPKGDELPKRGFEHGRDKFYPEMDPKPDSNVEIKVDPNSDRLQLLEPFKPWNGKELKTNVLLKVEGKCTTDHISAAGVWLKYKGHLENISYNTLIGAQNKETGEVNKAYDLDGTEYDIPGLMMKWKSDGRPWTVIAEHNYGEGSAREHAALSPRFLGGEILLVKSFARIHETNLKKQGVLPLTFANESDYDKISSGDVLETLNLVDMIAKDGNNGGEIDVKITKPNGESFTIKAKHTMSKDQIDFFKAGSAINYIGNIRRNE

>gi|429242751|ref|NP_594031.2| aconitate hydratase (predicted)

>Candida albicans mt1 (XP_716225.1)

MLSASRTALRAPRSVRGLATASLTKDSQVNQNLLESHSFINYKKHLENVEIVKSRLNRPLTYAEKLLYGHLDDPHNQEIERGVSYLKLRPDRVACQDATAQMAILQFMSAGIPQVATPSTVHCDHLIQAQVGGPKDLARAIDLNKEVYDFLSTACAKYNLGFWKPGSGIIHQIVLENYAFPGALLIGTDSHTPNAGGLGQLAIGVGGADAVDVMSGLPWELKAPKIIGVKLTGKMSGWTSPKDIILKLAGITTVKGGTGSIVEYFGSGVDTFSCTGMGTICNMGAEIGATTSVFPFNDSMVDYLNATGRSEIAQFAQVYKKDFLSADEGAEYDQVIEIDLNTLEPHINGPFTPDLATPVSKMKETAIANGWPLEVKVGLIGSCTNSSYEDMTRAASIIKDAGAHGLKSKALYTVSPGSEQVRATIARDGQLKTFEDFGGVVMANACGPCIGQWDRQDIKKGDKNTIVSSFNRNFTARNDGNPATHAFVASPEMATVYAISGDLGFNPITDTLVGADGKEFKLKEPQGVGLPPDGYDPGENTYQAPPEDRASVEVVISPTSDRLQKLSPFKPWDGKDAERLPILIKAVGKTTTDHISMAGPWLKYRGHLENISNNYMIGAINAENGKANEVRNHYTGKYDGVPQTAAAYRDAGHKWVVIGDENFGEGSSREHAALEPRFLGGFAIITKSFARIHETNLKKQGLLPLNFKNPADYDKINFDDEVDLIGLTTLAPGKDVILRVHPKEGEAWEAVLTHTFNSEQLEWFKHGSALNFIKSKY

>Candida albicans mt2 (XP_712172.1)

MLRARSVLSGSVSRRALATQAFNFSVPPEYQSRTPPYSKLINNLSTVKKILKNQPLTLAEKILYSHLCNPEESLTSSNVSDIRGQQYLKLHPDRVAMQDASAQMALLQFMTCGMSSTAVPASIHCDHLIVGKDGAEEDLVKSIATNKEVFDFLQSCGEKYGIQFWGPGSGIIHQIVLENFAAPGLMMLGTDSHTPNAGGLGAIAIGVGGADAVDALTGTPWELKAPKILGVKLTGKLSGWSSPKDVITTLAGILTVRGGTGYIVEYFGDGVKTLSCTGMATICNMGAEIGATTSTFPYQEAHKRYLIDTHRAPIAEAADSVNSEFKFLQADEGAEYDKVIEINLSELEPHVNGPFTPDLSTPISKFGETAQKEGWPETVSAGLIGSCTNSSYQDMSRAVSIIKQAEAVGLKPKIPFFVTPGSEQIRATIERDGLINTFESNGAIVLANACGPCIGQWDRTDVPKTSTDFNAIFTSFNRNFRARNDGNRNTMNFLTSPDMVTAMIYSGDMNFNPVTDSIELPNGKEFRFQPPQGKDLPQSGFIPGRSEFYPEANPQPKPEVEVNVSPSSDRLQLLEPFQPWSGEELSTTVLLKVEGKCTTDHISAAGVWLKYKGHLENISYNTLIGAVNKETGEVNKAYDLNGDAYGIPELMIKWKDESRPWVVVAEHNYGEGSAREHAALSPRFLGGSVILVKSFARIHETNLKKQGMLPLTFADEADYDKISSGDLLTTIDLKDMIAKDGNNGGTLRVKVTKRDGSEFEILAKHTMSKDQVEFFKAGSAINYIGNTKKGL

>Schizosaccharomyces pombe mt1

MFCKISRAPARMGSRIFTQSTLRSFSCAPVAANIDAKKVAMSNFEKNKFINYQRIKDNLEIVKKRLNRPLTYSEKILYGHLDDPVNQDIERGVSYLKLRPDRVACQDATAQMAILQFMSAGMPEVAVPVTVHCDHLIEAYEGGPIDLERANVTNKEVYDFLQTACAKYNIGFWRPGSGIIHQIVLENYAFPGGLLIGTDSHTPNAGGLGMVAIGVGGADAVDVMANLPWELKCPKVIGVKLTGQLKGWTSPKDVILKVAGILTVKGGTGAIVEYFGPGVESLSCTGMGTICNMGAEIGATTSIFPFNPRMSEYLRATNRSAIADYAEEFAPIIAADENAHYDQIIEIDLNTLEPHLNGPFTPDLATPISKFKEAVKKNDWPQELKVGLIGSCTNSSYEDMSRAASICQQAIDKGIKTKSLFTITPGSEQVRATLTRDGQLDTMRKAGGIVLANACGPCIGQWKRTDVKKGEKNSIVTSYNRNFTGRNDANPATHAFVTSPDIVTAMVFSGDMNFNPLTDTLKDKDGNDFKFEPPTGAGLPSKGYDPGSNTYVAPSSVNVKDVAIDPHSKRLQRLTPFKKWDGKDMKGLKILIKAKGKCTTDHISAAGPWLKYRGHLQNISNNYMIGAINAENGEANKLKDQLTGEYKTVPNVAIDYRDHGIRWVTLGEQNFGEGSSREHAALEPRYLGGAAVITKSFARIHETNLKKQGLLPLTFADPAAYDKISPFDTVDIDGLTTFAPGKPLTLVVHPADGSAEWSTKLNHTFNKDQIEWFKAGSALNHMANMHKQK

>Schizosaccharomyces pombe mt2

MATFARMKLCLSGSSQAIPSKGISLVAARFQSTASRASYVTPPYEKLMGKLQQVRKFLPGQKLTLAEKVLYSHLVNPEESFSGVSPSDIRGSLYLKLNPDRVAMQDASAQMALLQFMTCGLEKTMIPASIHCDHLIVGHRGANSDIPDSIANNKEIFDFLQSAAKKYGIQFWGPGSGIIHQIVLENYAAPGGMMLGTDSHTPNAGGLGMIAIGVGGADAVDAMTNTPWELKAPKIIGVNLTGAMSGWTTPKDLILHLAGKLTVRGGTGHIIEYFGPGVASLSCTGMATVCNMGAEVGATTSIFPYTDSMRRYLIATHRAEVADAASEVHSEYNYLAADTGAKYDQIIDINLSELTPSLNGPFTPDLSTPVSKFGEAIEKNKWPKKLSAGLIGSCTNSSYQDMTCVVDVVEQAISAGLKPKVPFLVTPGSEQIRATIERDGITERLEEAGATVLANACGPCIGMWKRTDDIASGEPNAILTSFNRNFRSRNDGNPSTMNFLTSPVIVAAKIFSSDLAFDPTHDTLQTPDGKAFKFRPPQGVELPSAGFIAGDSSYIPEPNPQPVPETEVTIDPKSDRLEALEPFEPYQGGEMENLKVAVKVKGKCTTDHISAAGKWLKYKGHLSNICNNTLIGAMNAATGEVNRAYDNGKGMTIPELMWKWKKDGQPWLVVAEHNYGEGSAREHAALQPRAMNGRIILTKSFARIHETNLKKQGVLPLTFVNEADYEKIDAEDKVSTRGIEQLLEGVLDQPITLVVTKKDGSVVEIPCKHTMSKDQIEFFKAGSALNLIREKAHSGVVNQKVIDSIKQQPDHYADAYIFNRHFVIAKGDQLGLPFHLKGVQVGDTIRLDKIASFGSRDFTLFGNPYVDPSLFTIEAVVLSFPKSALSVRVKHKRRHRHDRVMKHKQTYTILRVTELKLN

>Aspergillus niger mt1 (XP_001393157.2)

MITTRLARMGALAPKSRLMLGARGMATVTDSPLDKKVEMSNLEKGNYINYKKMSENLDIVRRRLSRPLTYAEKVLYSHLDDPHGQEIERGSSYLKLRPDRVACQDATAQMAILQFMSAGMPSVATPTTVHCDHLIEAQVGGDKDLARANDINKEVYDFLASATAKYNIGFWKPGSGIIHQIVLENYAFPGGLMIGTDSHTPNAGGLATAAIGVGGADAVDVMAGLPWELKAPKVIGVKLTGQMSGWTTPKDIILKVAGLLTVKGGTGAIIEYHGPGVNSLSCTGMATICNMGAEIGATTSMFPFNDRMYDYLKATKRQHIGNFSREYAKELREDEGAEYDQLIEINLSELEPHVNGPFTPDLATPISKFKEAVEANKWPEELKVGLIGSCTNSSYEDMSRAASIAQDALDHGLKSKSLFTVTPGSEQIRATIERDGQLKTLEEYGGVILANACGPCIGQWDRKDVKKGEANSIISSYNRNFTGRNDANPATHAFVASPDLVVAMTVAGTLKFNPLTDKLKDKDGKEFMLKPPSGEGLPAQGYDPGRDTYQAPPTNREGVNVAVAPTSDRLQILAGFEKWDGKDATGIPILIKCQGKTTTDHISMAGPWLKYRGHLDNISNNMLIGAINAENGEANKVKNAFTGEYDAVPATARDYKARGVKWVVIGDWNYGEGSSREHAALEPRHLGGLAIITRSFARIHETNLKKQGMLPLTFSDPADYDKIKPEDKVDLLCTELEVGKPVTLRVHPKDGASFDIKLSHTFNESQIEWFRDGSALNTMARKAGN

>Aspergillus niger mt2 (XP_001393703.1)

MVRQLVWQRATASQRLAPKCLSPQRLFSRRGLATEASPAASSRLPPYPKIVQNLENVRRVLGQDRNLTLAEKILYAHLDNPEESLLTGTNNGRDIRGKANLKLKPDRVAMQDASAQMALLQFMSCGLPSTAVPASIHCDHMIVGERGADTDLPASIKGNSEVFDFLESAAKRYGIEFWPPGAGIIHQSVLENYAAPGLMMLGTDSHTPNAGGLGAIAIGVGGADAVDALVDAPWELKAPRVLGVRLEGKLNGWAAPKDIILHLAGKLTVRGGTGFIIEYHGPGLETLSCTGMATICNMGAEVGATTSVFPFSPNHVPYLQATHRADVARAASEIASSGPRNLLRADGGAEYDELITIDLSTLEPHINGPFTPDFSVPLSAFADTVREKQWPESLGAGLIGSCTNSSYEDMTRAENLVKQASAAGLKPKSDFFITPGSEQIRATLDRDQTLSTFSQAGGTVLANACGPCIGQWKRTDGVPKGEDNAILTSYNRNFPGRNDGNRRTMNFLASPELVTAMVYSGSTTFNPMTDSITTPSGEEFRFQPPTGYALPSDGFADGNPDFQPTAAVPDPSCEVIVSPTSDRLALLEPFAPFPKGDLSGLKVLYKVRGQCTTDTISAAGPWLKYKGHLPNISANTLIGAVNAATGETNVAYDEAGKQYSIPDLAAQWKADGVEWLVVAEENYGEGSAREHAALQPRYLGGRIIVAKSFARIHETNLKKQGVVPLTFENPADYDKIDACDQVATEGLYEVLQAGGQGSIQLRVTKRSGESVVIPVKHTLSPDQCAFVLAGSALNLLAQKANKKSA

BACTERIA

>Escherichia coli (AAC74358.1)

MSSTLREASKDTLQAKDKTYHYYSLPLAAKSLGDITRLPKSLKVLLENLLRWQDGNSVTEEDIHALAGWLKNAHADREIAYRPARVLMQDFTGVPAVVDLAAMREAVKRLGGDTAKVNPLSPVDLVIDHSVTVDRFGDDEAFEENVRLEMERNHERYVFLKWGKQAFSRFSVVPPGTGICHQVNLEYLGKAVWSELQDGEWIAYPDTLVGTDSHTTMINGLGVLGWGVGGIEAEAAMLGQPVSMLIPDVVGFKLTGKLREGITATDLVLTVTQMLRKHGVVGKFVEFYGDGLDSLPLADRATIANMSPEYGATCGFFPIDAVTLDYMRLSGRSEDQVELVEKYAKAQGMWRNPGDEPIFTSTLELDMNDVEASLAGPKRPQDRVALPDVPKAFAASNELEVNATHKDRQPVDYVMNGHQYQLPDGAVVIAAITSCTNTSNPSVLMAAGLLAKKAVTLGLKRQPWVKASLAPGSKVVSDYLAKAKLTPYLDELGFNLVGYGCTTCIGNSGPLPDPIETAIKKSDLTVGAVLSGNRNFEGRIHPLVKTNWLASPPLVVAYALAGNMNINLASEPIGHDRKGDPVYLKDIWPSAQEIARAVEQVSTEMFRKEYAEVFEGTAEWKGINVTRSDTYGWQEDSTYIRLSPFFDEMQATPAPVEDIHGARILAMLGDSVTTDHISPAGSIKPDSPAGRYLQGRGVERKDFNSYGSRRGNHEVMMRGTFANIRIRNEMVPGVEGGMTRHLPDSDVVSIYDAAMRYKQEQTPLAVIAGKEYGSGSSRDWAAKGPRLLGIRVVIAESFERIHRSNLIGMGILPLEFPQGVTRKTLGLTGEEKIDIGDLQNLQPGATVPVTLTRADGSQEVVPCRCRIDTATELTYYQNDGILHYVIRNMLK

>Azospirillum lipoferum (YP_005039952.1)

MTTFTGQDTLKTRRSLSAGGKSYDYFSLKAAEEAGLGDLSRLPFSMKVLLENLLRFEDGRTVSVDDVKAVAQWLVDKRSDREIAYRPARVLMQDFTGVPAVCDLAAMREAMASLGGDPAKINPLVPVDLVIDHSVMVDYFGGNDAFEKNVELEFERNLERYAFLRWGQKAFDNFRVVPPGTGICHQVNTEYLAQVVWTDSDPSGKPVAYPDTLVGTDSHTTMVNGLSVLGWGVGGIEAEAAMLGQPISMLIPEVIGFKLTGRLKEGMTATDLVLTVTQMLRKKGVVGKFVEFFGPGLDSMTLPDRATIGNMAPEYGATCGIFPIDAETIRYLTFTGRDPDRVALVEAYAKAQGMWREPDSPDPVFSDILELDMGTVEPSLAGPKRPQDRVALSGIAQGFAKDMTEAYKADDPTKAVPVQGADYSLEQGAVVIAAITSCTNTSNPAVLVAAGLLAKKAVEKGLKQKPWVKTSLAPGSQVVTDYLAKAGLQPYLDRIGFNIVGYGCTTCIGNSGPLPEPIAAAVEEGNLVVGAVLSGNRNFEGRVNPHTRANYLASPPLCVAYALAGNLNIDLTKDPIGTGTDGPVYLKDIWPSNREVQDAIDASLTADMFRSRYSDVFKGPEQWQAIATAEGQTYQWQEGSTYVKLPPFFTGLTKTPDPVSDVRGARALAVLGDSITTDHISPAGSIKRTSPAGEYLLSYQVRPQDFNSYGARRGNHEVMMRGTFANIRIRNELIPGVEGGETKHYPSGERLPIYTAAMRYADEGVPLVVVAGKEYGTGSSRDWAAKGTRLLGIRAVIAESFERIHRSNLVGMGILPLQFKDGLTRADLNLDGSETFDIAGIEQDLRPRKDVTLTLTRADGKVETYPLLLRIDTLDEVEYYRNGGVLNFVLRNLAK

>Rhodospirillum centenum (YP_002299535.1)

MSTITGQDSLKTRRTLDVGGRTYDYFSLKAAEKAGLGDLSRLPFSMKVLLENLLRFEDGRTVSVDDVKAVAQWLKDRRSDREIAYRPARVLMQDFTGVPAVCDLAAMREAMQALGGDPQKINPLTPCDLVIDHSVMVDSFGSPTAFQENVDLEFQRNGERYAFLRWGQKAFANFRVVPPGTGICHQVNLEYLAQTVWTDTDQTGTEVAYPDTLVGTDSHTTMVNGLAVLGWGVGGIEAEAAMLGQPISMLIPEVVGFRLTGKLKEGATATDLVLTVTQMLRRKGVVGKFVEFYGPGIEHLTLADRATIANMAPEYGATCGIFPIDAETIRYLSFTGRDPNRVELVEAYARAQGMWWTPDAAEPVFTDTLELDLSTVESSLAGPKRPQDRVQLSDACADFKAFLEKDVAGRPASQPTPVPGTDYAIDHGHVVIAAITSCTNTSNPSVLVAAGLLAKKAVEKGLTRKPWVKTSLAPGSQVVTEYLEASGLQGWLDRLGFNLVGYGCTTCIGNSGPLPDPISKAVDDGKLTVASVLSGNRNFEGRVNAQVRANYLASPPLVVAYALAGSMNLDLTREPLGTGKDGQPVYLRDIWPSNHEVEATIAQYLTPEMYRSRYSNVFAGPEQWQAIRTAEGETYRWEGASTYVKRPPLFDGIAPVPGDVSDVTGARALAILGDSITTDHISPAGSIKKASPAGEYLTGHGVGVVDFNSYGARRGNHEVMMRGTFANIRIRNEMVPGVEGGVTRFVPTGEVMPIYDAAMKYQAEGTPLVVFAGQEYGTGSSRDWAAKGTRLLGVRAVVAESFERIHRSNLVGMGVLPLQFPAGVNRQTLKLDGSETFDIAGVEAGLKPRMTLALTITRADGSKQTVDLLCRIDTLDEVDYYKHGGILQYVLRSLAAPKVAAE

>Oceanibaculum indicum (WP_008945706.1)

MTGTDTLKTRRTLVVDGDEYEYYSIEAAQKAGLGDVSRLPMSLKVLLENLLRYEDGRTVTVDDVMAMGAWLKERKSTREIAYRPARVLMQDFTGVPAVVDLAAMRDAVAAMGGDPRTINPLSPVDLVIDHSVMVDNFGTMTSFEENVDHEFQRNGERYAFLRWGQKAFDNFRVVPPGTGICHQVNLEYLSQVVWTGKDGNRTVAYPDTLVGTDSHTTMVNGLAVLGWGVGGIEAEAAMLGQPVSMLIPEVVGFKLTGKLREGMTATDLVLTITQMLRARGVVGKFVEFYGPGLDNLSLADRATIANMAPEYGATCGFFPIDKEALRYLAFTGRDDKRVKLVEAYAKAQGMWRDEKSPDPVFTDSMELDLDTVQPSLAGPKRPQDRVLLSDAAASFGKAMAEIGASGKQVPVKGADYKLEDGRVVIAAITSCTNTSNPSVLIAAGLVAQKALAKGLKAKPWVKTSLAPGSQVVTDYLEAAGLQKSLDQVGFNLVGYGCTTCIGNSGPLPEPIANAVDEGDLLVCSVLSGNRNFEGRVHPQVKANYLASPPLVVAYALAGTMKLDLTTEPLGTGSDGKPVYLKDIWPSNKEIQDAMEKSLTAEMFKRRYANVFQGPEQWQAIDTPESLTYSWDDRSTYVKNPPYFKGMSKTVEGGFSNVSGARVLALLGDSITTDHISPAGSIKKDSPGGSYLMEHGVPPAEFNSYGARRGNHEVMMRGTFANIRLKNEAAGNTQGGFAKYVPSGEVMSIYDASMKYQAEGTPLVVVAGKEYGTGSSRDWAAKGTNLLGIKAVLAESFERIHRSNLVGMGVLPLQFKDGDTRKTLGLTGDEIIDISGIGTGISPRMDVPVTIRYADGRTKQITALCRIDTADEVEYFRNGGILHYVLRNMVKAA

>Azospirillum brasilense (YP_005030377.1)

MTTFTGQDSLKTRRSLSVGGKSYDYFSIKAAEDAGLGDLSRLPYSMKVLLENLLRFEDGRTVSTDDVKAVAQWLHDKRSDREIAYRPARVLMQDFTGVPAVCDLAAMREAMAALGGDPKKINPLVPVDLVIDHSVMVDYFGNPSAFEKNVELEFERNLERYAFLRWGQKAFDNFRVVPPGTGICHQVNVEYLAQGVWTDTDPAGKLVAYPDTLVGTDSHTTMVNGLGVLGWGVGGIEAEAAMLGQPISMLIPEVVGFKLTGRLKEGTTATDLVLTVTQMLRKKGVVGKFVEFYGPGLDHLTLADRATIGNMAPEYGATCGIFPIDAETIRYLTFTGRDADRVAMVEAYARAQGMWRDAGTPDPVFTDALELDMTTVEPSLAGPKRPQDRVPLSQAAQSFGTDLVGAFKAEDADRSVPVKGCGYNLDQGAVVIAAITSCTNTSNPAVLVAAGLLARKAVEKGLKSKPWVKTSLAPGSQVVTDYLAKAGLQPYLDQLGFNIVGYGCTTCIGNSGPLPDPIAAAVEEGNLVVAAVLSGNRNFEGRVNPHTRANYLASPPLCVAYALAGNMKIDLAKDPIGTGHDGQPVYLKDVWPTNQEVQDAIDASLSAEMFRSRYGNVFEGPEQWRGIQTAEGQTYEWQAGSTYVKLPPFFADMPKTPDAVSDVRGARALAVLGDSITTDHISPAGSIKKTSPAGEYLLSHQVRPQDFNSYGARRGNHEVMMRGTFANIRIRNEMLAGVEGGETRHYPSGEQLPIYTAAMRYAQEGVPLVVIAGKEYGTGSSRDWAAKGTKLLGIRAVIAESFERIHRSNLVGMGILPLQFKDGLTRNDLALDGTETFDIDGIEQDLRPRKDVTMTITRADGQTRQVPLLLRIDTVDEVEYYRNGGVLNFVLRNLAK

>Methylobacterium extorquens (YP_001640100.1)

MASLDSFKARQTLQAGGKTYTYYSIPEAEKNGLADSTALPFSMKVILENLLRFEDDRSVKRADIEAAVAWLGNQGRAETEIAFRPSRVLMQDFTGVPAVVDLAAMRDAMVALGGDPQKINPLVPVDLVIDHSVIVDEFGTPKALGDNVALEYARNGERYTFLKWGQSAFDNFSVVPPGTGICHQVNLEYLSQTVWTRTEDGAEIAYPDSLVGTDSHTTMVNGLAVLGWGVGGIEAEAAMLGQPLSMLIPEVIGFKLSGKLPEGTTATDLVLTVTQMLRKKGVVGKFVEFYGPGLDDMPVADRATISNMAPEYGATCGFFPIDQKTIDFLKVTGRQDDRIALVEAYAKAQGMWRDAKTPDPVFTDTLELDMSTVRPSLAGPKRPQDRVLLDSAKAGFADSMEKEFKKAADIARRYPVEGTNFDIGHGDVVIAAITSCTNTSNPSVMIGAGLLARNAVAKGLTSKPWVKTSLAPGSQVVGEYLDKSGLQASLDALGFNLVGFGCTTCIGNSGPLPAPISKAINDNDVVAAAVLSGNRNFEGRVNPDVRANYLASPPLVVAYALAGSLQIDITTEPLGQGSDGKPVYLKDIWPSSEEVNRFIEENITSELFKSRYADVFGGDENWKGVEVTEAETFAWDGGSTYVQNPPYFEGMTKTPDPITDIEGARILGLFLDSITTDHISPAGNIRAASPAGAYLQEHQVRVQDFNQYGTRRGNHEVMMRGTFANIRIKNQMVRDEAGNVVEGGWTLHQPDGERMYIYDAAMRYAEEGTPLVVFAGKEYGTGSSRDWAAKGTKLLGVRAVIAESFERIHRSNLVGMGVVPLVFQGEESWESLGLKGDETVTIKGLSGELKPRQTLTAEITSADGSKREVPLTCRIDTLDELEYFRNGGILPYVLRSLAA

CYANOBACTERIA

>Nostoc punctiforme (YP_001865517.1)

MLEQYRKHVAERAALGIPPLPLDAKQTSELCELLKNPPKGQEEILLHLLRDRVSPGVDPAAYVKAGFLTAIAKKEITSPLVSRIEAVQLLGTMIGGYNVQSLIDLLQFPTVSVSDSSETPLVMGGQGKEPIAAYAANALSKILLVYDAYHDVLELSKTNPFAKVVINSWAEAEWFTMRPTVSEAITVTVFKVPGETNTDDLSPAQSATTRPDIPLHALVMLESRQPGSLQTIAELKKKGHPVAYVGDVVGTGSSRKSAINSVLWHMGNDIPFVPNKRAGGYVLGGAIAPIFFNTAEDAGALPIQCDVTKLETGMVITIHPYKGEITNEAGEVISTFALKPDTILDEVRAGGRIPLLIGRTLTDKTRLALGLEHSTVFTRPQQAFDTGKGYSLAQKMVGKACGLTGVRPGTSCEPIITTVGSQDTTGPMTRDELKELACLGFSADLVIQSFCHTAAYPKPVDIQTHHELPDFFASRGGVALRPGDGIIHSWLNRMLLPDTVGTGGDSHTRFPLGISFPAGSGLVAFAAALGVTPLDMPESVLVRFKGELQPGITLRDVVNAIPYVAIQKGLLTVEKQNKKNVFSGRILEIEGLPDLKVEQAFELTDASAERSCAGCTIKLSAETISEYLRSNVALLKNMIARGYHDPRTMLRRVAKMEEWLANPVLLEGDADAEYAEIIEIDLNEIKEPIVAAPNDPDNVKLLSEVANDPVQEVFVGSCMTNIGHYRATAKVLEGAGEVKTRLWIAPPTRMDEHQLKEEGVYSVFGAAGARTEMPGCSLCMGNQARVADGTTVFSTSTRNFNNRMGKDARVYLGSAELAAVCALLGRLPTVQEYLDIVASRIHPFADDLYQYLNFDQILGFEDEGRVIALEDMPRLEDILGMPASSLR

>Cylindrospermum stagnale (YP_007147681.1)

MLESYRQHVVERAALGIPPLPLDAKQTSALCELLKNPPKGEEEVLLQLLRDRIPPGVDAAAYVKAGFLTAIAKEEITSPLVSPIEAVELLGTMVGGYNVQSLIDLLQLPTVSVSDSSETPLVMGGQGKEPIAAYAATALSKILLVYDAYHDVLELSKTNPLAKQVIDSWAEAEWFTIRPTLPEAITVTVFKVPGETNTDDLSPAQSATTRPDIPLHALVMLESRQPGSLETIAELKKKGHPVAYVGDVVGTGSSRKSAINSVLWHLGNDIPFVPNKRAGGYILGSAIAPIFFNTAEDAGALPIQCDVTKLETGDVITIHPYKGEITNEAGEVVSTFTLKPETIFDEVRAGGRIPLLIGRTLTDKTRQALGLPPSTVFIRPQAPADSGKGYTLAQKMVGKACGLPGVRPGTSCEPIMTTVGSQDTTGPMTRDELKELACLGFSADLVIQSFCHTAAYPKPVDIKTHHDLPDFFASRGGVALRPGDGIIHSWLNRMLLPDTVGTGGDSHTRFPLGISFPAGSGLVAFAGALGVMPLDMPESVLVRFKGELQPGITLRDIVNAIPYVAMQKGLLTVEKQNKKNVFSGKILEIEGLPDLKVEQAFELTDASAERSCAGCTIKLSEETVAEYLRSNVALLTNMIARGYHDERTLLRRIAKMEEWLANPVLLAGDTDAEYAEIIEIDLNEIKEPIVAAPNDPDNVKLLSEVANDPVQEVFVGSCMTNIGHYRATAKVLEGAGEVKTRLWIAPPTRMDEHQLKEEGVYSVFGAAGARTEMPGCSLCMGNQARVADGTTVFSTSTRNFNNRMGKDARVYLGSAELAAVCALLGRIPTVEEYLEIVAKKIQPFADNLYRYLNFDQIAGFEDEGRVIPLEEMPRIEEILGMPAAVK

>Microcoleus vaginatus (ZP_08491449.1)

MLESYRRNAAERAALGIPPLPLDAQQTSELCELLKNPPAGEEETLIELLRDRVPPGVDNAAYVKAGFLTGIAKGEIHSPLIAPKWAVYLLGTMMGGYNVQSLIDLLNPTAEVAIPATAARALSKTLLVFDAFHDVIALSDTNPYAKQVVDSWADAEWFTGRPVLPEAITVTVLKVPGETNTDDLSPAPDATTRPDIPLHALAMLESKMPGTLQTIAHLKQKGHPVAYVGDVVGTGSSRKSAINSVLWHIGNDIPFVPNKRSGGYILGSAIAPIFFNTAEDSGALPIECDVSKMETGMVITIHPYKGEITNEAGEIISTFTLKPDTILDEVRAGGRIPLLIGRTLTDKTRAALGLEPSTIFTRPTIPVDTGKGFTLAQKMVGKACGLSGVRPGTSCEPVMTTVGSQDTTGPMTRDELKELACLGFSADLVIQSFCHTAAYPKPVDVKTHHELPDFISERGGIALRPGDGIIHSWLNRMLLPDTVGTGGDSHTRFPLGISFPAGSGLVAFAAALGVMPLDMPESVLVRFKGELQPGVTLRDVVNAIPYVAIQKGLLTVEKQNKKNIFSGRIMEIEGLPNLKVEQAFELTDATAERSCAGCTIKLSVETVSEYIRSNVALLKNMVARGYQDARTMMRRVAKMEEWLANPVLLEADADAEYVEVIEIDLNEIKEPIVAAPNDPDNVKLLSEVAGDRVDEVFVGSCMTNIGHYRATAKVLEGAGEVKTRLWICPPTRMDEQQLKEEGFYSIFGAAGARTEMPGCSLCMGNQARVKDATTVFSTSTRNFNNRMGKDAQVYLGSAELAAVCSLLGRIPTVQEYNEIVAKRIDPFAGDLYRYLNFDQIAGFEDEGRVIALEDMPRIEDILGIPDGVLSK

>Crinalium epipsammum (YP_007144948.1)

MLEAYRQHVAERAKLGIPPLPLDAQQTSELCELLKNPPAGEEETLLELLRDRIPPGVDSAAYVKAGFLTAIAKGEITSSLVSPIYAVELLGTMIGGYNVQSLIDLLQFNQNASLQEAAAKALSKMVLVYDAFHDVEELAKTNSYAQQVIEAWETAEWFTSRPPVPEAITVTVFKVPGETNTDDLSPATHATTRPDIPLHALAMLESRQPGSLETIVELKKKGHPVAYVGDVVGTGSSRKSAINSVLWHIGNDIPFVPNKRAGGYVLGSAIAPIFFNTAEDSGALPIECDVSSMETGMVITIHPYKGEITNEAGEVISTFTLKPDTILDEVRAGGRIPLLIGRTLTDKIRAAQGLEPSPIFTRPRPPIDTGKGYTLAQKMVGKACGLQGVRPGTSCDPIMTTVGSQDTTGPMTRDELKELACLGFSADLVMQSFCHTAAYPKPVDIKTHHELPDFISQRGGVALRPGDGIIHSWLNRMLLPDTVGTGGDSHTRFPLGISFPAGSGLVAFAAALGVMPLDMPESVLVRFKGELQPGITLRDIVNAIPYVAMQKGLLTVEKKNKKNIFSGRIMEIEGLPDLKVEQAFELTDATAERSCAGCTIKLSVETISEYIRSNIALLKNMVARGYADARTIMRRVAKMEEWLANPVLMEADADAEYAEIIEIDLNEIKEPIVAAPNDPDNVKLLSEVANDLVQEVFVGSCMTNIGHYRATAKVLEGEGAVKTRLWICPPTRMDETQLKEEGYYGIFDAAGARTEMPGCSLCMGNQARVADGATVFSTSTRNFNNRLGKDAKVYLGSAELAAVCALLGRLPNVQEYLDIVGKKINPFAGDLYRYLNFDQITGFEDEGRVVSKEEEAMLAGVK

>Anabaena cylindrica (YP_007159342.1)

MLAKYQQHTQERAQLDIPPLPLDAQQTSELCELLKNPPKGEEELLLNLLRDRIPPGVDQAAYVKAGFLTAIAKEEITSPLVSPIDAVELLGTMIGGYNVQSLIDLLQVSTTSVSTSSETPLVMKGEGREQIAAYAASSLSRILLVYDAFHDVLELSKTNPYAKRVINSWAEAEWFLSRPTLPEAITVTVFKVPGETNTDDLSPATHATTRPDIPLHALAMLETRQPGSLETIAELKKKGFPVAYVGDVVGTGSSRKSAINSVLWHLGNDIPFVPNKRAGGYILGSAIAPIFFNTAEDAGALPIQCDVTKMETGDIITIYPYKGEVTNAAGEVISTFSLKPDTILDEVRAGGRIPLLIGRTLTDKTRQALGLEPSNLFIRPQAPADTGKGYTLAQKMVGKACGLPGVRPGTSCEPIMTTVGSQDTTGPMTRDELKELACLGFSADLVMQSFCHTAAYPKPVDIKTHQELPDFFAQRAGVALRPGDGIIHSWLNRMLLPDTVGTGGDSHTRFPLGISFPAGSGLVAFAGALGVMPLDMPESVLVRFKGELQPGITLRDVVNAIPYVAMQKGLLTVEKQNKKNIFSGKILEIEGLPDLKVEQAFELTDASAERSCAGCTIKLSEETVAEYLRSNIALLTNMVARGYHDERTIMRRVAKMEEWLANPVLLSADTDAEYAEIIEIDLSEIKEPIVAAPNDPDNVKLLSEVANDPVQEVFVGSCMTNIGHYRATAKVLEGAGEVKARLWIAPPTRMDEHQLKAEGVYNVFVAANARTEIPGCSLCMGNQARVDDNTTVFSTSTRNFNNRMGKGAQVYLGSAELAAVCALLGRLPNVQEYLDIVAERIHPFADDLYRYLNFDQIAGFEDEGRVISKEEQAALV

>Nodularia spumigena (ZP_01631498.1)

MLEQYRHHVAERAQLGIPPLPLDAKQTSELCELLQNPPEGEEDTLLHLLCDRVPPGVDEAAYVKAGFLTAIAKSEITSPLVSPIAAVELLGTMVGGYNVQSLIDLLQVSSGESKDGEAPLVMKGEGREPIAAHAATALSKILLVYDAFHDVLELAQSNPFAKRVVDSWANAEWFTIRPTVPEAITVTVFKVPGETNTDDLSPATHATTRPDIPLHALAMLESRQPGSLETIVELKQKGHPLAYVGDVVGTGSSRKSAINSVLWHLGNDIPYVPNKRAGGYILGSAIAPIFFNTAEDAGALPIECDVSNLETGMVITIHPYKGEITNQAGEVISTFTLKPETIFDEVRAGGRIPLLIGRTLTDKTREALGLEASNLFIRPQQPNDTGKGYTLAQKMVGKACGLPGVRPGTSCEPLMTTVGSQDTTGPMTRDELKELACLGFSADLVMQSFCHTAAYPKPVDIKTHQELPDFFAQRAGVALRPGDGIIHSWLNRMLLPDTVGTGGDSHTRFPLGISFPAGSGLVAFAGALGVMPLDMPESVLVRFKGELQPGITLRDIVNAIPYVAIQKGLLTVEKKNKKNIYSGRILELEGLPDLKVEQAFELTDASAERSCAGCTIKLSIETVSEYLRSNVTLLKNMVARGYTDARTIMRRVAKMEEWLANPTLMEADADAEYAEIIDIDLNEITQPIVAAPNDPDNVKLLSEVANDPVQEVFVGSCMTNIGHYRATGKVLEGAGAVKARLWICPPTRMDEHQLKTEGVYNIFEAANARTEMPGCSLCMGNQARVEDGTTVFSTSTRNFNNRMGKDARVYLGSAELAAVCALLGRIPTVREYLDIVAEKIHPFAGDLYRYLNFDQIAGFEDEGRVIAVEDMPKLEDILGMSAVTK

>Raphidiopsis brookii (ZP_06305547.1)

MLEEYQRHTQERENLGIPPLPLDVEQTSQLCELLKNPPLGQEELLLHLLRDRIPPGVDQSAYIKAGFLTAIAKQEITSPLISPLTAVELLGTMVGGYNVQSLINLLSSPVAGYAATALSKILLVYDSFNDVLELSQTNSYAKQVIDSWSAGEWFTSRPTLPDKITVTVFKVPGETNTDDLSPATHATTRPDIPLHALAMLETRQPGSLETIAQLKQTGYPIAYVGDVVGTGSSRKSAINSVLWHIGNDIPFVPNKRAGGYILGGAIAPIFFNTAEDAGALPIQCDVTQMETGDIITIYPYQGEITKEGKIIATFNLKPDTILDEVRAGGRIPLLIGRTLTDKTRQALGLAPSDLFIRPQLPKDTGKGYTLGQKMVGRACGLPGVRPGTSCEPIMTTVGSQDTTGPMTRDELKELACLGFSADLVMQSFCHTAAYPKPVDVKTHQELPDFFASRGGVALRPGDGIIHSWLNRMLLPDTVGTGGDSHTRFPLGISFPAGSGLVAFAGALGVMPLDMPESVLVRFKGELQPGVTLRDIVNAIPYVAIQKGLLTVEKKNKKNIFSGRIMEIEGLPDLKVEQAFELTDASAERSCAGCTIKLSEETIAEYLRSNIALLKNMVARGYGDGRTIMRRVAKMEAWLANPLLLEADPDAEYAEVIEINLTEIKEPIVAAPNDPDNVKLLSAVANDTVQEVFVGSCMTNIGHYRATAKVLEGAGPVKARLWIAPPTRMDEYQLKQEKVYDVFVNAQARTEVPGCSLCMGNQARVEDNTTVFSTSTRNFNNRMGNGAQVYLGSAELAAVCALLGRIPTPKEYMSVVIEKINPFADNLYRYLNFDQITGFEDQGRVISKEQQATLI

________________________________________________________________________________

ISOCITRATE DEHYDROGENASE

MITOCHONDRIAL (NAD-dependent)

>Arabidopsis thaliana (NM_119730.2)

MARRSVSIFNRLLANPPSPFTSLSRSITYMPRPGDGAPRTVTLIPGDGIGPLVTGAVEQVMEAMHAPVHFERYEVLGNMRKVPEEVIESVKRNKVCLKGGLATPVGGGVSSLNMQLRKELDIFASLVNCINVPGLVTRHENVDIVVIRENTEGEYSGLEHEVVPGVVESLKVITKFCSERIARYAFEYAYLNNRKKVTAVHKANIMKLADGLFLESCREVAKHYSGITYNEIIVDNCCMQLVAKPEQFDVMVTPNLYGNLIANTAAGIAGGTGVMPGGNVGAEHAIFEQGASAGNVGNDKMVEQKKANPVALLLSSAMMLRHLRFPTFADRLETAVKQVIKEGKYRTKDLGGDCTTQEVVDAVIAALE

>Arabidopsis lyrata (XP_002869074.1)

MARRSVSMLSRLLANPPSPFTALSRSITYMPRPGDGAPRTVTLIPGDGIGPLVTGAVEQVMEAMHAPVHFERYEVLGHMRKVPEEVIESVKRNKVCLKGGLATPVGGGVSSLNMQLRKELDIFASLVNCINVPGLVTRHENVDIVVIRENTEGEYSGLEHEVVPGVVESLKVITKFCSERIARYAFEYAYLNNRKKVTAVHKANIMKLADGLFLESCREVAKHYPGITYNEIIVDNCCMQLVAKPEQFDVMVTPNLYGNLVANTAAGIAGGTGVMPGGNVGAEHAIFEQGASAGNVGNDKIVEQKKANPVALLLSSAMMLRHLRFPTFADRLETAVKQVIQEGKYRTKDLGGDCTTQEVVDAVIAALE

>Ricinus communis (XP_002528761.1)

MARRSIPILKKLLSSSNNESTCSRLVSRRSVTYMPRPGDGAPRGVTLIPGDGIGPLVTGAVEQVMEAMHAPVYFERYEVHGDMKKVPAEVIESIKKNKVCLKGGLATPMGGGVSSLNVQLRKELDLYASLVNCFNLPGLPTRHENVDIVVIRENTEGEYSGLEHEVVPGVVESLKVITKFCSERIAKYAFEYAYLNNRKKVTAVHKANIMKLADGLFLESCREVATKYPGIKYNEIIVDNCCMQLVSKPEQFDVMVTPNLYGNLVANTAAGIAGGTGVMPGGNVGADHAIFEQGASAGNVGNEKIVEQKKANPVALLLSSAMMLRHLQFPSFADRLETAVERVISEGKYRTKDLGGDSSTQEVVDAVIAALD

>Vitis vinifera (XP_002265376.2)MAPRTLPILKQLLSKSSSYNTNFIGSRFAPKRSVTYMPRPGDGAPRPVTLIPGDGIGPLVTGAVEQVMDAMHAPVYFERYEVHGDMKKVPEEVLESIRKNKVCLKGGLATPMGGGVSSLNVQLRKELDLYASLVNCFNLPGLPTRHQNVDIVVIRENTEGEYSGLEHEVVPGVVESLKVITKFCSERIAKYAFEYAYLNNRKKVTAVHKANIMKLADGLFLESCREVATKYPGIKYSEIIVDNCCMQLVSKPEQFDVMVTPNLYGNLVANTAAGIAGGTGVMPGGNVGADHAVFEQGASAGNVGHQKLVEQKKANPVALLLSSAMMLRHLQFPSFADRLETAVKRVISEGKYRTKDLGGDSSTQEIVDAVIATLD

>Fragaria vesca (XP_004287740.1)

MARRSISILRHLLTKPLTPAVAAQTRSVTYKPRPGDGAPRAVTLIPGDGIGPLVTNAVEQVMEAMHAPVYFEKFEVTGDMPRVPEEVIESIRKNKVCLKGGLATPMGGGVSSLNMQLRRDLDLYASLVNCFNMRGLQTKHDNVDIVVIRENTEGEYSGLEHEVIPGVVESLKVITKFCSERIAKYAFEYAYLNNRKKVTAVHKANIMKLADGLFLESCREVAEKYPGIAYNEIIVDNCCMQLVSKPEQFDVMVTPNLYGNLVANTAAGIAGGTGVMPGGNVGADHAIFEQGASAGNVGNQKMLEQKKANPVALLLSSAMMLRHLQFPSFSDRLENAVERVILEGKFRTKDLGGHSTTQEVVDAVIAALD

>Glycine max (XP_003537678.1)

MATTRSAPLLKHLLTRLNPSRSVTYMPRPGDGTPRGVTLIPGDGIGPLVTGAVEQVMEAMHAPLYFEKYEVHGDMKAVPAEVLESIRKNKVCLKGGLATPMGGGVNSLNVQLRKELDLYASLVNCFNLPGLPTRHDNVDIVVIRENTEGEYSGLEHEVVPGVVESLKVITKFCSERIAKYAFEYAYLNNRKKVTAVHKANIMKLADGLFLESCREVATRYPGIKYNEIIVDNCCMQLVSKPEQFDVMVTPNLYGNLVANTAAGIAGGTGVMPGGNVGADHAVFEQGASAGNVGNDKVVEQQKANPVALLLSSAMMLRHLQFPAFADRLETAVKKVILEGKYRTKDLGGTSTTQEVVDAVIDALD

>Medicago truncatula (XP_003612317.1)

MATRRSTNLLKHLISTRHHLTHHRTVTYMPRPGDGTPRTVTLIPGDGIGPLVTGAVEQVMEAMHAPVLFEKFEVHGNMKAIPSEVMESIKKNKVCLKGGLATPMGGGVSSLNLQLRKELDLYASLVNCFNLEGLTTRHDNVDIVVIRENTEGEYAGLEHEVVPGVVESLKVITKFCSERIAKYAFEYAYLNNRKKVTAVHKANIMKLADGLFLESCREVATKYPGIKYNEIIVDNCCMQLVSKPEQFDVMVTPNLYGNLVANTAAGIAGGTGVMPGGNVGADHAVFEQGASAGNVGKEKVVQEKKANPVALLLSSAMMLRHLQFPVFAERLESAVKRVILEGKYRTKDLGGTSTTQEVVDAVIDALQ

>Brachypodium distachyon (XP_003579972.1)

MARRSAPLLRRLVSSPPPLPGHGAAARRTVTYMPRPGDGTPRPVTLIPGDGIGPLVTGSVQQVMDAMHAPVYFETYDVHGDMPSVPPAVIDSIRRNKVCLKGGLATPVGGGVSSLNMQLRKELDLYASLVNCANVPGLPTRHKNVDIVVIRENTEGEYSGLEHEVVPGVVESLKVITKFCSERIAKYAFEYAYLNYRKKVTAVHKANIMKLADGLFLESCREVASKYPGIEYNEIIVDNCCMQLVSKPEQFDVMVTPNLYGNLVANTAAGLVGGTGVMPGGNVGQDHAIFEQGASAGNVGNDNLVEQKKANPVALLLSSAMMLRHLQFPSFADRLETAVKRVVAEGKYRTKDLGGTSTTQEVTDAVIANLD

>Zea mays (ACG37220.1)

MARRSAPLLRRLVSASSPPALQSLPDHVGVLARRTVTYMPRPGDGAPRAVTLIPGDGIGPLVTGAVRQVMEAMHAPVYFETYDVHGDMPTVPPAVIESIRRNKVCIKGGLATPVGGGVSSLNMQLRKELDLYASLVQCSNLPGLPTRHEGVDIVVIRENTEGEYSGLEHEVVPGVVESLKVITKFCSERIAKYAFEYAYLNNRKKVTAVHKANIMKLADGLFLESCREVASKYPGIQYNEMIVDNCSMQLVSKPEQFDVMVTPNLYGNLVANTAAGIVGGTGIMPGGNVGQDYAIFEQGASAGNVGNENLVEQKKANPVALLLSSAMMLRHLQFPSFADRLETAVKRVVAEGTYRTKDLGGSSTTQEVTDAVVANLD

>Hordeum vulgare (BAJ97934.1)

MARRSAPLLRRLVASAPSLPGHGGGARRTVTYMPRPGDGTPRPVTLIPGDGIGPLVTGAVEQVMEAMHAPVYFETYDVHGDMPAVPPAVIESIRRNKVCLKGGLATPVGGGVSSLNMQLRKELDLFASLVNCANVPGLPTRHKNVDIVVIRENTEGEYSGLEHEVVPGVVESLKVITKFCSERIAKYAFEYAYLNYRKKVTAVHKANIMKLADGLFLESCREIAAKYPSIEYNEIIVDNCCMQLVSRPEQFDVMVTPNLYGNLVANTAAGLVGGTGVMPGGNVGQDHAIFEQGASAGNVGNDNLVEQQKANPVALLLSSAMMLRHLQFPSFADRLETAVKRVVAEGQYRTKDLGGTSTTQEVTDAVIANLD

>Coccomyxa subellipsoidea (EIE23533.1)

MLRQARKLLGFSRTLQSSFPPALASQQTRTKITYVPSPGDARPQTVTLIPGDGIGPEISEAVKEVFEALKAPVVWEQFDNVHGSDIYGNPNLEIPEEVLESITRNGVCLKGTLFTPLSANNTSTQSLNVQLRKTLDLHVNLVHGWTMPGVPSRFSDIDIVVIRENTEGEYAGLEHEVVPDVVESLKIITEEKSRRTVEYAFGYAYLNNRKKVTAVHKANIMKLSDGLFLREFNKVAKKYPSIKAEAMIVDNTCMQLVSNPQQFDVMVTPNLYGNLVMNVVAGLTGGPGLFPGVNVGENVAIFEQGARHVAKDIAGMGVANPSAALLSAAMMLRHLNLPGFSDRLERAVLNTISNEADSIKTPDIGGTGTTRSFVNSIIEKL

>Chlamydomonas reinhardtii (XP_001697281.1)

MLSRLGLGLLARAAVAGGEGLAARAFGTGSAYLPLPGDARSQIVTLIPGDGIGPEVTKAVVDVVAAMQAPITWERFDYLSGSEETAAGSVPRTSVPKEVLDSIRRNGVCLKGTLFTPLNKENTNTQSLNVQLRKDLDLHVNVVHGFSIPGLPTRYNNLDIVVIRENTEGEYSGLEHEVVEGVVESLKVITYEKSLRTAQYAFEFAYLNHRKKVSAIHKANIMKLGDGMFLKACREVARNFPNIKYEEVIVDNTCMQLVNKPHQFDVMVTPNLYGNLVSNVVAGLCGGFGVVPGGNIGDGVAVFEQGARHVAKDLAGAGVANPTATLLSTAMLLRHLKLAGFADRLEAAVLKVYTDGDEAALTPDVGGSGTLLRFTEAVVRNLQE

>Homo sapiens (NM_005530.2)

MAGPAWISKVSRLLGAFHNPKQVTRGFTGGVQTVTLIPGDGIGPEISAAVMKIFDAAKAPIQWEERNVTAIQGPGGKWMIPSEAKESMDKNKMGLKGPLKTPIAAGHPSMNLLLRKTFDLYANVRPCVSIEGYKTPYTDVNIVTIRENTEGEYSGIEHVIVDGVVQSIKLITEGASKRIAEFAFEYARNNHRSNVTAVHKANIMRMSDGLFLQKCREVAESCKDIKFNEMYLDTVCLNMVQDPSQFDVLVMPNLYGDILSDLCAGLIGGLGVTPSGNIGANGVAIFESVHGTAPDIAGKDMANPTALLLSAVMMLRHMGLFDHAARIEAACFATIKDGKSLTKDLGGNAKCSDFTEEICRRVKDLD

>Canis lupus (XP_536213.2)

MAGPAWISKVSRLLGAFHNQKQVTRAFAGGVQTVTLIPGDGIGPEISAAVMKIFDAAKAPIQWEERNVTAIQGPGGKWMIPPEAKESMDKNKMGLKGPLKTPIAAGHPSMNLLLRKTFDLYANVRPCVSIEGYKTPYTDVNIVTIRENTEGEYSGIEHVIVDGVVQSIKLITEEASRRIAEFAFEYARNNHRSNVTAVHKANIMRMSDGLFLQKCREVAENCKDIKFNEMYLDTVCLNMVQDPSQFDVLVMPNLYGDILSDLCAGLIGGLGVTPSGNIGANGVAIFESVHGTAPDIAGKDMANPTALLLSAVMMLRHMGLFDHAARVEAACFATIKDGKSLTKDLGGNAKCSDFTEEICRRVRDLD

>Rattus norvegicus (EDL95540.1)

MRPGVAAVAAVREVDAMAGSAWVSKVSRLLGAFHNTKQVTRGFAGGVQTVTLIPGDGIGPEISASVMKIFDAAKAPIQWEERNVTAIQGPGGKWMIPPEAKESMDKNKMGLKGPLKTPIAAGHPSMNLLLRKTFDLYANVRPCVSIEGYKTPYTDVNIVTIRENTEGEYSGIEHVIVDGVVQSIKLITEGASKRIAEFAFEYARNNHRSNVTAVHKANIMRMSDGLFLQKCREVAENCKDIKFNEMYLDTVCLNMVQDPSQFDVLVMPNLYGDILSDLCAGLIGGLGVTPSGNIGANGVAIFESVHGTAPDIAGKDMANPTALLLSAVMMLRHMGLFDHAAKIEAACFATIKDGKSLTKDLGGNSKCSDFTEEICRRVKDLD

>Oreochromis niloticus (XP_003437638.1)

MAGNAWRSLLTQAVGVAVRKPALASASFSRGVKTVTMIPGDGIGPEISAAVMKIFEAAKAPITWEERNVTAIKGPGGRWMIPPDAKESMDRSKIGLKGPLKTPIAAGHPSMNLLLRKTFDLYANVRPCVSIEGYKTPYTDVNLVTIRENTEGEYSGIEHMIVDGVVQSIKLITENASRRIAEYAFEYARNNKRTSVTAVHKANIMRMSDGLFLRKCREVAENYKDIKFTEMYLDTVCLNMVQDPTQFDVLVMPNLYGDILSDLCAGLIGGLGVTPSGNIGANGVAIFESVHGTAPDIAGMDLANPTALLLSAVMMLHHMGLHDHADKIQTACFDTIRDKKVLTKDLGGSAKCSEFTAEICRRVQDLD

>Caenorhabditis elegans (NP_492330.2)

MLGKCIKKASSTVGQSIRYSSGDVRRVTLIPGDGIGPEISASVQKIFEAADAPIAWDPVDVTPVKGRDGVFRIPSRCIELMHANKVGLKGPLETPIGKGHRSLNLAVRKEFSLYANVRPCRSLEGHKTLYDNVDVVTIRENTEGEYSGIEHEIVPGVVQSIKLITETASRNVASFAFEYARQNGRKVVTAVHKANIMRQSDGLFLSICREQAALYPDIKFKEAYLDTVCLNMVQDPSQYDVLVMPNLYGDILSDLCAGLVGGLGVTPSGNIGKGAAVFESVHGTAPDIAGQDKANPTALLLSAVMMLRYMNLPQHAARIEKAVFDAIADGRAKTGDLGGTGTCSSFTADVCARVKDLE

>Saccharomyces cerevisiae (NP_014779.1)

MLRNTFFRNTSRRFLATVKQPSIGRYTGKPNPSTGKYTVSFIEGDGIGPEISKSVKKIFSAANVPIEWESCDVSPIFVNGLTTIPDPAVQSITKNLVALKGPLATPIGKGHRSLNLTLRKTFGLFANVRPAKSIEGFKTTYENVDLVLIRENTEGEYSGIEHIVCPGVVQSIKLITRDASERVIRYAFEYARAIGRPRVIVVHKSTIQRLADGLFVNVAKELSKEYPDLTLETELIDNSVLKVVTNPSAYTDAVSVCPNLYGDILSDLNSGLSAGSLGLTPSANIGHKISIFEAVHGSAPDIAGQDKANPTALLLSSVMMLNHMGLTNHADQIQNAVLSTIASGPENRTGDLAGTATTSSFTEAVIKRL

>Candida albicans (EEQ45740.1)

MFRQVTKSAPVIRTTQRLFARSYIAGQFTGSKGSDGKYTVTLIEGDGIGPEISQAVKDIYAAADVPIHWEPVDVTPLLIDGKTTLPQPAVDSVNKNLVALKGPLATPVGKGHTSMNLTLRRTFNLFANVRPCKSIAGYETPYENVDTVLIRENTEGEYSGIEHTIVPGVVQSIKLITKPASEKVIRYAFEYAKSINKPHVLVVHKASIMKLSDGLFVNTAKEVAQEYPDVSLDFELLDNTSLRLTADPSQYKNVVMVMPNLYGDIMSDLSSGLIGGLGLTPSGNMGNKVSIFEAVHGSAPDIAGKGLANPTALLLSSCMMLRHMSLNSDADRIENAVLKTIASGPENRTGDLKGTATTTRFTEEVIKNL

>Schizosaccharomyces pombe (NP_595203.2)

MSMLSTLRTAGSLRTFSRSACYSFQRFSSTKAAAGTYEGVKNANGNYTVTMIAGDGIGPEIAQSVERIFKAAKVPIEWERVKVYPILKNGTTTIPDDAKESVRKNKVALKGPLATPIGKGHVSMNLTLRRTFGLFANVRPCVSITGYKTPYDNVNTVLIRENTEGEYSGIEHEVIPGVVQSIKLITRAASERVIRYAFQYARQTGKNNITVVHKATIMRMADGLFLECAKELAPEYPDIELREEILDNACLKIVTDPVPYNNTVMVMPNLYGDIVSDMCAGLIGGLGLTPSGNIGNQASIFEAVHGTAPDIAGKGLANPTALLLSSVMMLKHMNLNDYAKRIESAIFDTLANNPDARTKDLGGKSNNVQYTDAIISKLK

>Aspergillus nidulans (CBF88356.1)

MFAARNFATPARQCLRSTRVAPNLASTRLQFRCYSAAADERVAKFKGQKDTDGKYTVTLIEGDGIGPEISQSVKDIFSAANAPIKWESVDVTPILKDGKTAIPDAAIDSVRKNYVALKGPLATPVGKGHVSLNLTLRRTFNLFANLRPCRSVAGYKTPYDNVDTVLIRENTEGEYSGIEHVVVDGVVQSIKLITREASERVLRFAFQYARSINKKKVRVVHKATIMKMSDGLFLNTAREVAKDFPDVEFDAELLDNSCLKITTDPTPYNDKVLVMPNLYGDILSDMCAGLIGGLGLTPSGNIGDECSIFEAVHGSAPDIAGKGLANPTALLLSSIMMLQHMGLNEHASRIQKAIFDTLAEGKTLTGDLGGKAKTHEYADAIIKRL

CYTOSOLIC

>Arabidopsis thaliana (AY093091.1)

MAFEKIKVANPIVEMDGDEMTRVIWKSIKDKLITPFVELDIKYFDLGLPHRDATDDKVTIESAEATKKYNVAIKCATITPDEGRVTEFGLKQMWRSPNGTIRNILNGTVFREPIICKNVPKLVPGWTKPICIGRHAFGDQYRATDAVIKGPGKLTMTFEGKDGKTETEVFTFTGEGGVAMAMYNTDESIRAFADASMNTAYEKKWPLYLSTKNTILKKYDGRFKDIFQEVYEASWKSKYDAAGIWYEHRLIDDMVAYALKSEGGYVWACKNYDGDVQSDFLAQGFGSLGLMTSVLVCPDGKTIEAEAAHGTVTRHFRVHQKGGETSTNSIASIFAWTRGLAHRAKLDDNAKLLDFTEKLEAACVGTVESGKMTKDLALIIHGSKLSRDTYLNTEDFIDAVAAELKERLNA

>Capsella rubella (EOA35225.1)

MAFEKIKVTNPIVEMDGDEMTRVIWKSIKDKLITPFVELDIKYFDLGLPHRDATDDKVTIESAEATKKYNVAIKCATITPDEGRVTEFGLKQMWRSPNGTIRNILNGTVFREPIICKNVPKLVPGWTKPICIGRHAFGDQYRATDAVIKGPGKLTMTFEGKDGKTETEVFTFTGEGGVSMAMYNTDESIRAFADASMNTAYEKKWPLYLSTKNTILKKYDGRFKDIFQEVYEASWKSKYEAAGIWYEHRLIDDMVAYALKSEGGYVWACKNYDGDVQSDFLAQGFGSLGLMTSVLVCPDGKTIEAEAAHGTVTRHYRVHQKGGETSTNSIASIFAWTRGLAHRAKLDDNAKLLEFTEKLEAACVGTVESGKMTKDLALIIHGSKLSRDTYLNTEEFIDAVAAELKTRLGA

>Ricinus communis (XP_002528517.1)

MAFEKIKVANPIVEMDGDEMTRIFWKSIKDKLIFPFLELDIKYFDLGLPHRDATDDKVTIESAEATLKYNVAIKCATITPDEARVKEFNLKQMWKSPNGTIRNILNGTVFREPIICKNVPRLVPGWTKPICIGRHAFGDQYRATDAVIKGAGKLKLVFVPEGQDEKTELEVYNFTGAGGVALSMYNTDESIRAFADASMNTAYQKKWPLYLSTKNTILKKYDGRFKDIFQEVYEASWKSKFEAAGIWYEHRLIDDMVAYALKSEGGYVWACKNYDGDVQSDFLAQGFGSLGLMTSVLVCPDGKTIEAEAAHGTVTRHYRVHQKGGETSTNSIASIFAWSRGLAHRAKLDDNARLLDFTEKLEAACIGVVESGKMTKDLALLIHGSKVTRDQYLNTEEFIDAVAADLAERLSKA

>Daucus carota (BAA34112.1)

MAFQKIKVANPIVEMDGDEMTRVFWKSIKDKLIFPFVELDIKYFDLGLPHRDATDDKVTVESAEATLKYNVAIKCATITPDEARVKEFGLKQMWKSPNGTIRNILNGTVFREPIICKNIPKLIPGWTKPICIGRHAFGDQYRATDAVIQGPGKLKLVFVPEGKEEKTELEVYNFTGAGGVALSMYNTDESIRSFAEASMATAYEKKWPLYLSTKNTILKKYDGRFKDIFQEVYEASWKSKYDAAGIWYEHRLIDDMVAYALKSDGGYVWACKNYDGDVQSDFLAQGFGSLGLMTSVLVCPDGKTIEAEAAHGTVTRHYRVHQKGGETSTNSIASIFAWSRGLAHRAKLDDNAALLSFAEKLEAACVGTVESGKMTKDLALILHGSKLSREHYLNTEEFIDAVASDLKARLAN

>Pisum sativum (AAS49171.1)

MAFQKIKVASPIVEMDGDEMTRVIWKSIKDKLIFPFLELDIKYFDLGLPHRDQTDDKVTVESAEATLKYNVAIKCATITPDEARVEEFGLKSMWRSPNGTIRNILNGTVFREPIICKNVPRLIPGWTKPICIGRHAFGDQYRATDAVIKGPGKLKMVFVPEGKGETTDLEVYNFTGEGGVALAMYNTDESIRSFAEASMATALEKKWPLYLSTKNTILKKYDGRFKDIFQEVYEASWKSKYEAAGIWYEHRLIDDMVAYALKSEGGYVWACKNYDGDVQSDFLAQGFGSLGLMTSVLVCPDGKTIEAEAAHGTVTRHFRVHQKGGETSTNGIASIFAWTRGLAHRAKLDDNAKLLELTEKLEAACIGAVESGKMTKDLALILHGSQLKREHYLNTEEFIDAVAAELKSKISA

>Zea mays (NP_001140324.1)

MAFNKIKVTNPIVEMDGDEMTRVFWKSIKDKLIFPFVDLDIKYFDLGLPHRDATDDKVTVEAAEATLKYNVAIKCATITPDEARVNEFGLKAMWKSPNGTIRNILNGTVFREPIICKNIPRLVPGWTKPICIGRHAFGDQYRATDAVIKGPGKLKLVFEGKEEQVELEVFNFTGAGGVALSMYNTDESIHAFADASMATAYEKKWPLYLSTKNTILKKYDGRFKDIFQEVYEAGWKTKFEAAGIWYEHRLIDDMVAYALKSEGGYVWACKNYDGDVQSDFLAQGFGSLGLMTSVLVCPDGKTIEAEAAHGTVTRHYRVHQKGGETSTNSIASIFAWTRGLAHRAKLDDNARLLDFTQKLEAACVGAVESGKMTKDLALLVHGSSNITRSHYLNTEEFIDAVADELRSRLAANSNL

>Oryza sativa (NP_001043749.1)

MAFEKIKVANPIVEMDGDEMTRIFWQSIKDKLIFPFLDLDIKYYDLGVLHRDATDDKVTVEAAEATLKYNVAIKCATITPDEARVKEFNLKQMWKSPNGTIRNIINGTVFREPIICKNVPRLVPGWTKPICIGRHAFGDQYRATDAVLKGPGKLKLVFEGKDEQIDLEVFNFTGAGGVALSMYNTDESIRAFAEASMTTAYEKKWPLYLSTKNTILKKYDGRFKDIFQEVYEAGWKSKFEAAGIWYEHRLIDDMVAYALKSEGGYVWACKNYDGDVQSDFLAQGFGSLGLMTSVLVCPDGKTIEAEAAHGTVTRHFRVHQKGGETSTNSIASIFAWTRGLAHRAKLDDNARLLDFALKLEAACVGTVESGKMTKDLALLIHGSSNVTRSHYLNTEEFIDAVAAELRSRLAAN

>Brachypodium distachyon (XP_003569476.1)

MAFEKIKVANPIVEMDGDEMTRVFWQSIKDKLIFPFLDLDIKYYDLGVLHRDATDDKVTVEAAEATLKYNVAIKCATITPDEDRVKEFNLKQMWRSPNGTIRNIINGTVFREPIICKNVPKLVPGWTKPICIGRHAFGDQYRATDAVLKGPGKLRLVFEGKEETVDLEVFSFTGAGGVALSMYNTDESIQGFAAASMATAYDKKWPLYLSTKNTILKKYDGRFKDIFQEVYEAEWKSKFEAAGIWYEHRLIDDMVAYALKSEGGYVWACKNYDGDVQSDFLAQGFGSLGLMTSVLVCPDGKTIEAEAAHGTVTRHFRVHQKGGETSTNSIASIFAWTRGLAHRAKLDDNARLLEFAQKLEDACVGTVESGKMTKDLALLVQGSSNVTRSHYLNTEEFIDAVAAELRTRLAAN

>Volvox carteri (XP_002957593.1)

MKALIGATRALGLLQQQSHYACLNPVLAAQTGVRTMATKITVANPVVDLDGDEMTRVIWQQIKDKLIKPYLDLKIIYFDLGLPNRDKTNDKVTEEAAYAIKEHNVGIKCATITPDEARVKEFGLKKMWKSPNGTIRNILNGTVFREPIVIKNIPRLVPGWTKPIVVGRHAFGDQYKATDFVVDGPGKLELSFTPADGGPARKFEVFKFEGSGVALAMYNTEESIRGFASSCFEYALQKKWPLYLSTKNTILKAYDGRFLQIFAETYESQYKQQYEAMGIWYEHRLIDDMVAQALKSNGGFVWACKNYDGDVQSDIVAQGYGSLGLMTSVLVTPDGKTVEAEAAHGTVTRHWREFQKGKPTSTNPVASIFAWTRGLAHRGKLDGNQELIQWCQDLEAAVIETIELGHMTKDLAICVHGTSKVAPNQYLNTEPFMDAIAETFARKRGGASK

>Chlamydomonas reinhardtii (XP_001698704.1)

MPLVSELGLRGYAFGPAKRVMWPLAALIPARQLPASRPCRGAAGRGGLSLRARAPNRTPVEWNSGVRTMATAGKIHVANPVVDLDGDEMTRVIWQQIKDKLILPYLDLKIVYFDLGLPNRDKTNDKVTEEAAYAIKEHNVGIKCATITPDEARVKEFGLKKMWKSPNGTIRNILNGTVFREPIVISNIPRLVPGWTKPIVVGRHAFGDQYKATDFVVDGPGKLEMIFTPAAGGAPRKFEVYSFEGPGVAMGMYNTEESIRGFASSCFEYALQKRWPLYLSTKNTILKSYDGRFLQIFAETYETQYKKQYEEAGIWYEHRLIDDMVAQGLKSSGGFVWACKNYDGDVQSDIVAQGYGSLGLMTSVLVTPDGKTVEAEAAHGTVTRHWREYQKGKPTSTNPVASIFAWTRGLAHRGKLDNNAELIQWTHDLEAAVIETIEQGHMTKDLAICVHGTTKVTPDQYLNTEPFMDAVADTFAKKRGGKK

>Ostreococcus tauri (XP_003082777.1)

MGGRGVDRSTAMTRFERCGSTASSKITAAPMVYVRGEEMTAYVMDLIRSRWIEPRVDVGGWETFDLRAKNRDDTEDRVLRDVIEAGKRIKAIFKEPTVTPTADQVKRLGLRKSWGSPNGAMRRGWNGITISRDTIHIDGVELGYKKPVLFERHAVGGEYSAGYKNVGKGKLTTTFTPSEGPDAGKTVVVDEREIVDEEAAVVTYHNPYDNVHDLARFFFGRCLEAKVTPYVVTKKTVFKWQEPFWQIMRTVFDEEFKAQFVAAGVMKEGEELVHLLSDAATMKLVQWRQGGFGMAAHNYDGDVLTDELAQVHKSPGFITSNLVGVHEDGTLIKEFEASHGTVADMDEARLRGEETSLNPLGMVEGLIGAMNHAADVHNIDRDRTHAFTTKMRTVIHQLFREGKGTRDLCGPSGLTTEQFIDAVAERLDA

>Homo sapiens (NP_005887.2)

MSKKISGGSVVEMQGDEMTRIIWELIKEKLIFPYVELDLHSYDLGIENRDATNDQVTKDAAEAIKKHNVGVKCATITPDEKRVEEFKLKQMWKSPNGTIRNILGGTVFREAIICKNIPRLVSGWVKPIIIGRHAYGDQYRATDFVVPGPGKVEITYTPSDGTQKVTYLVHNFEEGGGVAMGMYNQDKSIEDFAHSSFQMALSKGWPLYLSTKNTILKKYDGRFKDIFQEIYDKQYKSQFEAQKIWYEHRLIDDMVAQAMKSEGGFIWACKNYDGDVQSDSVAQGYGSLGMMTSVLVCPDGKTVEAEAAHGTVTRHYRMYQKGQETSTNPIASIFAWTRGLAHRAKLDNNKELAFFANALEEVSIETIEAGFMTKDLAACIKGLPNVQRSDYLNTFEFMDKLGENLKIKLAQAKL

>Mus musculus (NP_034627.3)

MSRKIQGGSVVEMQGDEMTRIIWELIKEKLILPYVELDLHSYDLGIENRDATNDQVTKDAAEAIKKYNVGVKCATITPDEKRVEEFKLKQMWKSPNGTIRNILGGTVFREAIICKNIPRLVTGWVKPIIIGRHAYGDQYRATDFVVPGPGKVEITYTPKDGTQKVTYMVHDFEEGGGVAMGMYNQDKSIEDFAHSSFQMALSKGWPLYLSTKNTILKKYDGRFKDIFQEIYDKKYKSQFEAQKICYEHRLIDDMVAQAMKSEGGFIWACKNYDGDVQSDSVAQGYGSLGMMTSVLICPDGKTVEAEAAHGTVTRHYRMYQKGQETSTNPIASIFAWSRGLAHRAKLDNNTELSFFAKALEDVCIETIEAGFMTKDLAACIKGLPNVQRSDYLNTFEFMDKLGENLKAKLAQAKL

>Canis lupus (XP_536047.2)

MSQKIRGGSVVEMQGDEMTRIIWELIKEKLIFPYVELDLHSYDLGIENRDATNDQVTKDAAEAIKKYNVGVKCATITPDEKRVEEFKLKQMWKSPNGTIRNILGGTVFREAIICKNIPRLVSGWVKPIIIGRHAYGDQYRATDFVVPGPGKVEITYTPSDGSEKMTYLVHNFEEGGGVAMGMYNQDKSIEDFAHSSFQMALSKSWPLYLSTKNTILKKYDGRFKDIFQEIYDKQYKSQFEAQNIWYEHRLIDDMVAQAMKSEGGFIWACKNYDGDVQSDSVAQGYGSLGMMTSVLVCPDGKTVEAEAAHGTVTRHYRMYQKGQETSTNPIASIFAWTRGLAHRAKLDNNKELSFFAKALEEVCVETIEAGFMTKDLAACIKGLPNVQRSDYLNTFEFMDKLGENLNIKLAQAKL

>Gallus gallus (XP_421965.2)

MSKKIHGGSVVEMQGDEMTRVIWELIKEKLIFPYVDLDLHSYDLGIEHRDATNDKVTVEAAEAIKKYHVGIKCATITPDEKRVEEFKLKQMWKSPNGTIRNILGGTVFREAIICKNIPRLVSGWVKPIVIGRHAYGDQYRATDFVVPGPGKVEMTYTPGDGGKPVTYLVHNFESCGGVAMGMYNLDQSIKDFAHSSFQMALSKGWPLYMSTKNTILKRYDGRFKDIFQEIYDREYKSQFEAKKIWYEHRLIDDMVAQALKSEGGFVWACKNYDGDVQSDSVAQGYGSLGMMTSVLICPDGKTVEAEAAHGTVTRHYRMHQKGQETSTNPIASIFAWTRGLAHRAKLDNNTSLKTFATALEEVCIETIESGFMTKDLAACIKGLPNVTRSDYLNTFEFMDKLAANLKGKLASLPKL

>Caenorhabditis elegans (NP_001255393.1)

MAAQKIQGGDIVEMQGDEMTRIIWDLIKEKLILPYVDLNVHFFDLGIEHRDATDDQVTIDAANATLKYNVAVKCATITPDEARVEEFKLKKMWKSPNGTIRNILGGTVFREPIIVKNVPRLVNTWSKPIIIGRHAHADQYKATDFVVPGAGKLEIKFVSADGTQTIQETVFDFKGPGVSLSMYNTDDSIRDFAHASFKYALQRKFPLYLSTKNTILKKYDGRFKDIFAEIYPEYEAEFKAAGIWYEHRLIDDMVAQAMKSDGGFVWACKNYDGDVQSDSVAQGYGSLGLMTSVLVCPDGKTVEAEAAHGTVTRHYRMHQKGQETSTNPIASIFAWSRGLAHRATLDKNSALETFANNLEAVCIETMEAGFLTKDLAICVKGGNASAVTRTDYLNTFEFLDKLAENLAKKQAH

>Saccharomyces cerevisiae (NP_013275.1)

MTKIKVANPIVEMDGDEQTRIIWHLIRDKLVLPYLDVDLKYYDLSVEYRDQTNDQVTVDSATATLKYGVAVKCATITPDEARVEEFHLKKMWKSPNGTIRNILGGTVFREPIIIPRIPRLVPQWEKPIIIGRHAFGDQYKATDVIVPEEGELRLVYKSKSGTHDVDLKVFDYPEHGGVAMMMYNTTDSIEGFAKASFELAIERKLPLYSTTKNTILKKYDGKFKDVFEAMYARSYKEKFESLGIWYEHRLIDDMVAQMLKSKGGYIIAMKNYDGDVESDIVAQGFGSLGLMTSVLITPDGKTFESEAAHGTVTRHFRQHQQGKETSTNSIASIFAWTRGIIQRGKLDNTPDVVKFGQILESATVNTVQEDGIMTKDLALILGKSERSAYVTTEEFIDAVESRLKKEFEAAAL

>Aspergilus nidulans (AAK76730.1)

MSSVRFTSALARRSLNSPLLPCGAPPISSALRSFSSASYSAFSSSSSSFPSPSSARALASTARISRPAATRSLYRVQTRTMATEVQKIKVKNPVVELDGDEMTRIIWKEIREKLILPFLDIDLKYYDLGLEYRDQTDDKVTTESAEAIKKYGVGVKCATITPDEARVEEFKLKKMWLSPNGTIRNILGGTVFREPIVIPRIPRLVPGWTKPIIIGRHAFGDQYRATDRVIPGPGKLELVYTPEGGQPEAIKVFDFPGGGVTQTQYNTDESIRGFAHASFKLALTKGLPLYMSTKNTILKKYDGRFKDIFQEIFESDYKKEFDAKGIWYEHRLIDDMVAQMIKSEGGFIMALKNYDGDVQSDIVAQGFGSLGLMTSTLITPDGQAFESEAAHGTVTRHYREHQKGRETSTNPIASIFAWTRGLIQRGKLDETPDVVKFAEELERACIDVVNEEGIMTKDLALSCGRKERDAWVTTREYMAAVERRLRANLKARL

>Candida albicans (XP_713421.1)

MGEIQKIKVKNPIVEMDGDEMTRIIWQFIKDKLITPYLDVDLKYYDLGIEYRDQTDDKVTTDAANAILKYGVGVKCATITPDEARVKEFNLKKMWLSPNGTLRNILGGTVFREPIVIDNIPRIVPSWEKPIIIGRHAFGDQYKATDIVVPGAGELKLVFKPKDGGEIQEYPVYNFEGPGVGLSMYNTDASIQDFAESSFQLAIERKLNLFSSTKNTILKKYDGRFKDIFEGLYASKYKTKMDELGIWYEHRLIDDMVAQMLKSKGGYIIAMKNYDGDVQSDIVAQGFGSLGLMTSVLVTPDGKAFESEAAHGTVTRHYRQHQQGKETSTNSIASIYAWTRGLIQRGKLDETPEVVKFAEDLEKAIIDTVSKDNIMTKDLALTQGKTDRSSYVTTEEFIDAVANRLNKNLGYA

>Schizosaccharomyces pombe (NP_594105.2)

MLEVRAAVKAPFKLAAAGRGFMNMRMASSKSFQKITVKNPVVEMDGDEMTRVIWKIIREKLVLPYMDIKLDYYDLGIEARDKTNDQITVDAAKAILKNDVGIKCATITPDEARVKEYNLKKMWKSPNGTIRNILNGTVFREPILIKNIPKYIPGWTNPICIGRHAFGDQYKSTDLVASGPGKLELSFTPKGNPSAKETYNVYEFNGSGVAMSMYNTDDSIRGFAHSSFQMALQKKMPLYLSTKNTILKKYDGRFKDTFQEVYESDYKQKFEELGLWYQHRLIDDMVAQAIKSNGGFVWACKNYDGDVMSDVVAQAYGSLGLMTSVLIHPNGRTFESEAAHGTVQRHYMQYLKGKKTSTNSIASIFAWTRGLAHRGRLDGNERLVKFANALEHACVRCVEKGIMTKDLYLLSKSPNGYVDTFEFLDAVKSELDSELVNIA

PEROXISSOME

>Arabidopsis thaliana (BT025983.1)

MEFEKIKVINPVVEMDGDEMTRVIWKFIKDKLIFPFLELDIKYFDLGLPNRDFTDDKVTIETAEATLKYNVAIKCATITPDEARVREFGLKKMWRSPNGTIRNILNGTVFREPIICRNIPRLVPGWTKPICIGRHAFGDQYRATDLIVNEPGKLKLVFEPSGSSQKTEFEVFNFTGGGVALAMYNTDESIRAFAESSMYTAYQKKWPLYLSTKNTILKIYDGRFKDIFQEVYEANWRSKYEAAGIWYEHRLIDDMVAYAMKSEGGYVWACKNYDGDVQSDFLAQGYGSLGMMTSVLVCPDGKTIEAEAAHGTVTRHYRVHQKGGETSTNSIASIFAWSRGLAHRAKLDSNAALLSYTEKLEAACMGTVESGKMTKDLALLIHGAKVRRDQYVNTEEFIDAVAWELKRRLLGNNSRL

>Populus trichocarpa (XP_002305928.1)

MAYEKIKVANPIVEMDGDEMTRIFWQSIKDKLIFPFVELDIKYFDLGLPHRDATDDKVTVESAEAALKYNVAIKCATITPDEARVKEFNLKQMWKSPNGTIRNILNGTVFREPIICKNIPRLVPGWTKPICIGRHAFGDQYRATDAVIKGAGKLKLVFVPEGQDEKTELEVYNFTGAGGVALAMYNTDESIRAFAEASMNTAYQKKWPLYLSTKNTILKKYDGRFKDIFQEVYEANWKSKYEAAGIWYEHRLIDDMVAYALKSEGGYVWACKNYDGDVQSDFLAQGFGSLGLMTSVLVCPDGKTIEAEAAHGTVTRHYRVHQKGGETSTNSIASIFAWSRGLAHRAKLDDNARLLDFTEKLEAACIGAVESGKMTKDLALLIHGSKVSRDHYLNTEEFIDAVAEELKARLSIKA

>Vitis vinifera (XP_002270617.1)

MAFDKIKVANPIVEMDGDEMTRVFWKSIKDKLIFPFLELDIKYFDLGLPHRDATDDKVTVESAEATLKYNVAIKCATITPDEGRMKEFDLKQMWKSPNGTIRNILNGTVFREPIICKNIPRLVPSWTKPICIGRHAFGDQYRATDTVIKGAGKLKLVFVPEGKDEKTELEVFNFTGAGGVALSMYNTDESIYAFAEASMNTAYLKQWPLYLSTKNTILKKYDGRFKDIFQEVYETQWKSKYEAAGIWYEHRLIDDMVAYALKSEGGYVWACKNYDGDVQSDFLAQGFGSLGLMTSVLVCPDGKTIEAEAAHGTVTRHFRVHQKGGETSTNSIASIFAWSRGLAHRAKLDDNARLLDFTEKLEAACVGTVESGKMTKDLALLIHGSKVTRDWYLNTEEFIDAVAAELTAKLSC

>Cucumis sativus (XP_004157023.1)

MAFQKIKVANPIVEMDGDEMTRVIWESIKNKLIFPFLELDIKYFDLGLPHRDATDDKVTIESAEATLKYNVAIKCATITPDEARVKEFGLKQMWRSPNGTIRNILNGTVFREPILCKNVPRLVPGWTKPICIGRHAFGDQYRATDTVIRGPGKLKLVFEGQETQEIEVFNFTGAGGVALAMYNTDESIRSFAEASMATAYEKKWPLYLSTKNTILKKYDGRFKDIFQEVYESQWKSKFEAAGIWYEHRLIDDMVAYALKSEGGYVWACKNYDGDVQSDFLAQGFGSLGLMTSVLVCPDGKTIEAEAAHGTVTRHFRVHQKGGETSTNSIASIFAWSRGLAHRAKLDDNASLLEFTEKLELACIDTVESGKMTKDLALILHGSKLSRDQYLNTEEFIDAVAEELKSRLLKA

>Glycine max (AAA33978.1)

AIHTGLCFSLLISHLTFYSSQSQIRTLAMAAFQKIKVANPIVEMDGDEMTRVIWKSIKDKLILPFLELDIKYYDLGLPYRDETDDKVTIESAEATLKYNVAIKCATITPDEARVKEFGLKSMWKSPNGTIRNILNGTVFREPILCKNIPRLVPGWTKAICIGRHAFGDQYRATDTVIKGAGKLKLVFVPEGQGEETEFEVFNFTGEGGVSLAMYNTDESIRSFAEASMATALEKKWPLYLSTKNTILKKYDGRFKDIFQEVYEASWKSKFEAAGIWYEHRLIDDMVAYALKSEGGYVWACKNYDGDVQSDFLAQGFGSLGLMTSVLVCPDGKTIEAEAAHGTVTRHFRVHQKGGETSTNSIASIFAWTRGLAHRAKLDDNAKLLDFTEKLEAACIGVVEAGKMTKDLALILHGSKLSREHYLNTEEFIDAVAAELSARLSA

BACTERIAS

>Escherichia coli (AAC74220.1)

MESKVVVPAQGKKITLQNGKLNVPENPIIPYIEGDGIGVDVTPAMLKVVDAAVEKAYKGERKISWMEIYTGEKSTQVYGQDVWLPAETLDLIREYRVAIKGPLTTPVGGGIRSLNVALRQELDLYICLRPVRYYQGTPSPVKHPELTDMVIFRENSEDIYAGIEWKADSADAEKVIKFLREEMGVKKIRFPEHCGIGIKPCSEEGTKRLVRAAIEYAIANDRDSVTLVHKGNIMKFTEGAFKDWGYQLAREEFGGELIDGGPWLKVKNPNTGKEIVIKDVIADAFLQQILLRPAEYDVIACMNLNGDYISDALAAQVGGIGIAPGANIGDECALFEATHGTAPKYAGQDKVNPGSIILSAEMMLRHMGWTEAADLIVKGMEGAINAKTVTYDFERLMDGAKLLKCSEFGDAIIENM

>Oceanicaulis (WP_009802584.1)

MNVHVSASKSAATATRTPTPITVAYGDGIGPEIMKASLKVLNAAGAALAPEPIEIGEQVYLKGVSAGIEDSAWDSLRRTKVFYKAPITTPQGGGFKSLNVTVRKSLGLFANVRPCAALSPFVATRHPDMDVVIVRENEEDLYAGIEHRQTDEVVQCLKLISRPGCERIVRYAFDYARANGRKKVTCLSKDNIMKLTDGLFHEVFDEIAKEYPDIKTDHWIIDIGTAKLADAPEQFDVIVTSNLYGDIISDVAAEITGSVGLGGSANIGEHCAMFEAIHGSAPMIAGQGIANPSGLLLAGVQMLVHIGQADVAEKIHNAWLKTLEDGVHTVDIASEDMTKEQVGTDAFADAVIARLGQEPSTFTPVRYNADAGGVEIAPYKRRERQTKTLVGVDVFVDMPTQNPDELADALNAAAGDASLSLKMITNRGVKVWPNGLPETFKTDHWRCRFMSGAVSKNADVVDLLGRIEAAGIDFVKTEQLYEFDGKPGYSLGQGQ

>Rhodospirillum centenum (YP_002297149.1)

MSDVTKVTIARGDGIGPEIMDATLEILEAAGAALEYEEIQIGESVYRRGILNGMEPAAWDSLRRTRVFLKAPITTPQGGGYKSLNVTIRGALGLYANIRPCVSYHPFVNTKHPQMDVVIVRENEEDLYAGIEYRQTQDVTQAVKLISRPGSEKIIRYAFEYARANHRRKVTAFVKDNIMKISDGLFHRVFDEIAAEYPEIEHETMIIDIGAARLADTPEQFDVIVTLNLYGDIISDIAAQITGSVGLAGSSNIGDQCAMFEAIHGSAPNIAGKGIANPSGLILASVMMLVHVGQAEAAERIHNAWLRMIEDGIHTVDIFKRGISKARVGTRDFAANLIDRLGERPVTLTPASYEVGAKATFAGIQLKTRNDAKKRLVGCDIFIDHTGGDGGAMAARVLPLVQDGLKLSVISNRGQKVWPDGAPETFCIDHWRLRVMGEGNAEVSQNAIAALIVRMADAGFDIVQTARLYTFDGAPGFSLAQGQ

>Azospirillum brasilense (YP_005032114.1)

MRETTPITVARGDGIGPEITDAVLHVMEAAGARLKVEEVPAGEQVYRRGHLGGLDAAGWGSIRRTRVFLKGPITTPQGYGNKSLNVTARTTLGLFANVRPCVSHHPYVRTRHPRMDVVIIRENEEDLYAGIEHRQTDDVIQSVKLISRPGSERIVRYAFDYARANHRRKVTAFVKDNVMKMTDGLFLKIFNEIAAEYPEIKADHLIVDIGAARLADQPERFDVIVTLNLYGDIVSDIAAQITGSVGLAGSANIGDACAMFEAIHGSAPMIAGQGIANPSGLLMAAVMMLVHIGQGDVAARIHNAWLKTIEDGVHTVDIFNRGVSRQRVGTHAFADAVVERLGQMPVTLPTVGYAVTARPAYESGVRLSPRRKAVKELVGVDVFLHWPGGNPDELAGLVLPVATPALRLSSISNRSQKVWPEGNIGVFCTDHWRCRFLAPEGVAVGHPDIVELLSGLARAGLDFTQTENLCNFDGKSGFSAT

>Azospirillum lipoferum (YP_005039206.1)

MRDITPVTVARGDGIGPEITDAVLYVMNAAGARLKVEEVPAGEAVYKRGHPGGLDAAGWGSIRRTRVFLKGPITMPQGYGNKSLNVVARTTLGLFANVRPCVSYHPYVRTRHPRMDVVIIRENEEDLYAGIEHRQTDDVIQSVKLISRPGSERIVRYAFDFARSNHRQKVTAFVKDNVMKMTDGLFLKIFYEIAADYPEIKADHMIVDIGAARLADQPERFDVIVTLNLYGDIVSDIAAQLTGSVGLAGSANIGETCAMFEAIHGSAPMIAGQGIANPSGLLMAAVMMLVHIGQGDIAARIHNAWLKTIEDGIHTADIYARGLGRVRAGTNAFAEAVVERLGQTPVTMPPVGYSTMRPAYDGLNRLAPRVRALKELVGVDVFLQWSGGLPDDLAELVLPLSTEALTLASISNRSQRVWPDGNADVFCTDHWRCRFLANGGPVQHGAIVELLGRLAEAGIDFTQTENLCNFDGKAGFSSPGQ

>gi|521982415|ref|WP_020493686.1| isocitrate dehydrogenase [Methylobacterium sp. WSM2598]

MSTQSNDRIAATLIPGDGIGPEISNAVVKILDALEAPFAWDVQQGGMAGIESSGDPLPAALLESVGRTKLALKGPLTTPVGGGFRSVNVRLREAFGLYANLRPVRTMIPGGRYEDIDIVLVRENLEGLYVAFEHFIAVGDDPRAVAISQGINTREEARRIVRFAFEYAVQHGRKKVTIVHKANVLKALTGLFLEAGREIAKEYEGRIAVDDRIVDACAMQLVLNPWQFDVIVTTNLFGDILSDQLAGLVGGLGMAPGANIGEKAAIFEAVHGSAPDIAGQGIANPLALLLAAALMLEHVQRSDLAGRLRSAILQTVQADSVRTRDIGGSASTQEFADAIIRRVLA

CYANOBACTERIA

>Nostoc punctiforme (YP_001868724.1)

MYEKITPPAAGAKIAFKNGEPIVPDNPIIPFIRGDGTGIDIWPATQKVLDAAVAKAYKGQRQISWFKVYAGDEACDLYGTYQYLPQDTLTAIEEYGVAIKGPLTTPVGGGIRSLNVALRQIFDLYACVRPCRYYAGTPSPHKNPEKLDVIVYRENTEDIYLGIEWRQGSEIGDRLIKILNEELIPATPEHGKKRIPLDSGIGIKPISKTGSQRLVRRAIKHALLLPKNKQQVTLVHKGNIMKYTEGAFRDWGYELATSEFRQETVTEQESWILSNKEKNPNISLEENARQIEPGFDNLTPDKKAQVVKEVETVLNTIWATHGDGKWKEKVLVNDRIADSIFQQIQTRPDEYSILATMNLNGDYLSDAAAAIVGGLGMGPGANIGDSSAIFEATHGTAPKHAGLDRINPGSVILSGVMMLEFLGWQEAADLIKKGLSDAIANSQVTYDLARLLEPPVEPLKCSEFADAIIQHFG

>Anabaena variabilis (YP_325323.1)

MYNKITPPTTGEKITFKNGEPVVPDNPIIPFIRGDGTGIDIWPATEKVLDAAVAKAYQGKRKISWFKIYAGDEACDLYGTYQYLPEDTLTAIREYGVAIKGPLTTPVGGGIRSLNVALRQIFDLYACVRPCRYYAGTPSPHKNPEKLDVIVYRENTEDIYLGIEWKQGSEIGDRLISILNKELIPATPEHGKKQIPLDAGIGIKPISKTGSQRLVRRAIKHALTLPKHKQQVTLVHKGNIMKYTEGAFRDWGYELATSEFRQETVTERESWILSNKEKNPNISLEDNARQIDPGFDALTPEKKAQIVKEVETVLNSIWETHGNGKWKEKVLVNDRIADSIFQQIQTRPDEYSILATMNLNGDYLSDAAAAIVGGLGMGPGANIGDSCAVFEATHGTAPKHAGLDRINPGSVILSGVMMLEYLGWQEAADLIKKGLSDAIANSQVTYDLARLLEPPVEPLKCSEFADAIIKHFG

>Anabaena cylindrica (YP_007155468.1)

MYDKITPPTTGAKITFKNGEPVVPENPIIPFIQGDGTGIDIWPATEKVLDAAVAKAYKGQRKISWFRVYAGDEACDLYGTYQYLPQDTLTAIEEYGVAIKGPLTTPVGGGIRSLNVALRQIFDLYTCVRPCRYYAGTPSPHKTPEKLDVIIYRENTEDIYLGIEWKQGSEIGDRLIKFLNEELIPATPEHGKKQIPLDAGIGIKPISKTGSQRLVRRAIKHALLLPKNKQQVTLVHKGNIMKYTEGAFRDWGYELATSEFRNETVTERESWILGNKEKNANLSLEENARMIDPGFDSLTPEKKAQIVKEVETTLNTIWESHGNGKWKEKIMVNDRIADSIFQQIQTRPDEYSILATMNLNGDYLSDAAAAIVGGLGMGPGANIGDSCAIFEATHGTAPKHAGLDRINPGSVILSGVMMLEFMGWQEAADLVKKGLGDAIANSQVTYDLARLMEPPVEPLKCSEFADAIIKHFG

>Synechocystis (WP_009630133.1)

MYEKIAPPTTGSTVTFNNGEPIVPDNPIIPFIRGDGTGVDIWPASQKVIDAAVAIAYKGAREISWFRVYAGDEACEQYGTYQYLPEDTLKAIKEYGIAIKGPLTTPVGGGIRSLNVALRQINDLYACVRPCRYYPGTPSPHKYPEKMDVIIYRENTEDIYLGIEWKQGSEIGTRLIKILNEELIPATPEHGKKQIPLDSGIGIKPISKTGSQRLVRRALKHALRLPKSKQMVTLVHKGNIMKYTEGAFRDWGYELATTEFRSDCVTERESWILGNKESNAELSLEANARLIEPGYSSLTPEKQNQICQEVEAVLSQIWATHGNGQWKDKVMVNDRIADSIFQQLQTRPDEYSILATMNLNGDYLSDAAAAVVGGLGMGPGANIGDDCAIFEATHGTAPKHAGLDRVNPGSVILSGVMMLEYMGWQEAADLIIQGIGDAIASGHVTYDLARLMEPPVQPLKCSEFADAIINAFSK

>Oscillatoria (WP_007358445.1)

MYEKITAPTTGSKITFKDGEPIVPDDPIIPFIRGDGTGVDLWPASQKVMDAAVETAYGGKKKINWFKIYAGDEACEVYGTYQYLPADTSNAIKEYGVAIKGPLTTPIGGGIRSLNVALRQNHDLYSCIRPCKYYPGTPSPHKTPELLDVIIYRENTEDIYLGIEWKEGSEIGNKLIALLNNELIPATPEHGKKQIPLDSGIGIKPISKKGSQRLIRRAIKHALRLPKAKQQVTLVHKGNIMKYTEGAFRDWGYELVKSEFRNECVTEMESWILSNKERNPEISLEDNARQIEPGYDALTPEKQAKICKDVEEVLSAIWESHGDGKWKEKIMVNDRIADSIFQQIQTRPAEYSILATMNLNGDYLSDAAAAIVGGLGMGPGANIGDECAIFEATHGTAPKHAGLDKVNPGSLILSGVMMLEYLGWQEAAELIKKGLAAAISNGEVTYDLARMMEPPVPELKCSEFADAIIKHFG
