# Supplementary material for: Evolution and Functional Implications of the Tricarboxylic Acid Cycle as Revealed by Phylogenetic Analysis
Source: Genome Biol Evol. 2014 Oct 1;6(10):2830–48. doi: 10.1093/gbe/evu221 (PMC4224347; doi:10.1093/gbe/evu221)
Supplement: Supplementary Data [file supp_evu221_Supplemental_DataSet_3.doc]

SUCCINIL-COA LIGASE

ALPHA CHAIN

>Arabidopsis thaliana 1 (NM_120913.5)

MSRQVARLIGSLSSKARRCSTGGSEVFPSCQSLTSLTQSRSFASDPHPPAAVFVDKNTRVLCQGITGKNGTFHTEQAIEYGTKMVAGVTPKKGGTEHLGLPVFNSVAEAKADTKANASVIYVPAPFAAAAIMEGIEAELDLIVCITEGIPQHDMVRVKHALNSQSKTRLIGPNCPGIIKPGECKIGIMPGYIHKPGKIGIVSRSGTLTYEAVFQTTAVGLGQSTCVGIGGDPFNGTNFVDCLEKFFVDPQTEGIVLIGEIGGTAEEDAAALIKASGTEKPVVAFIAGLTAPPGRRMGHAGAIVSGGKGTAQDKIKSLNDAGVKVVESPAKIGSAMYELFQERGLLKQ

>Arabidopsis thaliana 2 (NM_001125791.1)

MSRQVTRLLGSLRHSGGGCSGSSKVCSLTSLVQSRSFGTTPPPPAAVFVDKNTRVICQGITGKNGTFHTEQAIEYGTKMVAGVTPKKGGTEHLGLPVFNTVAEAKAETKANASVIYVPAPFAAAAIMEGLAAELDLIVCITEGIPQHDMVRVKAALNSQSKTRLIGPNCPGIIKPGECKIGIMPGYIHKPGKIGIVSRSGTLTYEAVFQTTAVGLGQSTCVGIGGDPFNGTNFVDCLEKFFVDPQTEGIVLIGEIGGTAEEDAAALIKENGTDKPVVAFIAGLTAPPGRRMGHAGGK

>Arabidopsis lyrata 1 (XP_002871316.1)

MSRQVARLIGSLSSKARRCSTGGSEVFPSCQSLTSLTQSRSFASDPHPPAAVFVDKNTRVLCQGITGKNGTFHTEQAIEYGTKMVAGVTPKKGGTEHLGLPVFNSVAEAKADTKANASVIYVPAPFAAAAIMEGIEAELDLIVCITEGIPQHDMVRVKHALNSQSKTRLIGPNCPGIIKPGECKIGIMPGYIHKPGKIGIVSRSGTLTYEAVFQTTAVGLGQSTCVGIGGDPFNGTNFVDCLEKFFVDPQTEGIVLIGEIGGTAEEDAAALIKASGTEKPVVAFIAGLTAPPGRRMGHAGAIVSGGKGTAQDKIKSLNDAGVKVVESPAKIGAAMYELFQERGLLKQ

>Arabidopsis lyrata 2 (XP_002872045.1)

MSRQVTRLLGSLRRSGGGGSSEVCSLTSLTQSRSFGTTPPPPAAVFVDKNTRVICQGITGKNGTFHTEQAIEYGTKMVAGVTPKKGGTEHLGLPVFNTVAEAKAETKANASVIYVPAPFAAAAIMEGLAAELDLIVCITEGIPQHDMVRVKAALNSQSKTRLIGPNCPGIIKPGECKIGIMPGYIHKPGKIGIVSRSGTLTYEAVFQTTAVGLGQSTCVGIGGDPFNGTNFVDCLEKFFADPQTEGIVLIGEIGGTAEEDAAALIKESGTDKPVVAFIAGLTAPPGRRMGHAGAIVSGGKGTAQDKIKSLRDAGVKVVESPAKIGAAMFEIFQERGLLK

>Solanum lycopersicum 1 (NP_001234277.1)

MARQATKLIANLSKKLSSSNPHTRCSEQTVWIGAAPPAVFVDKNTRVICQGITGKNGTFHTEQAIEYGTKMVGGVTPKKGGTEHLGLPVFNTVEEAKAETKANASVIYVPPPFAAAAIMEGLEAELDLIVCITEGIPQHDMVRVKAALKKQSRTRLIGPNCPGIIKPGECKIGIMPGYIHKPGRIGIVSRSGTLTYEAVFQTTAVGLGQSTCVGIGGDPFNGTNFVDCLEKFIADPQTEGIVLIGEIGGTAEEDAAALIKESGTQKPVVAFIAGLTAPPGRRMGHAGAIVSGGKGTAQDKIKALKEAGVTVCESPAKIGVSMLEVFKQRGLV

>Solanum lycopersicum 2 (NP_001234574.1)

MARQATRLISNLSTKLNPSSPTMSASPLWHQYRYFGSPPPPPAVFVDKNTRVICQGITGKNGTFHTEQAIEYGTKMVGGVTPKKGGTEHLGLPVFNTVAEAKVETKANASVVYVPPPFAAAAIMEAMEAELDLVVCITEGIPQHDMVRVKAALKKQLRTRLIGPNCPGIIKPGECKIGIMPGYIHKPGRIGIVSRSGTLTYEAVFQTTAVGLGQSTCVGIGGDPFNGTNFVDCLERFIADPQTEGIVLIGEIGGTAEEDAAALIKESGTQKPVVAFIAGLTAPPGRRMGHAGAIVSGGKGTAQDKIKALKEAGVTVCESPAKIGVTMLDVFKQRGLA

>Cucumis sativus (XP_004137529.1)

MGRQAVAKLIGSAINRKTSSSSSSSSVFSRAASQIRHYSAAPSPPPAVFVDKNTRVICQGITGKNGTFHTEQAIEYGTKMVGGVTPKKGGTEHLGLPVFNSVAEAKAETKANASVIYVPPPFAAAAIMEAMEAELDLVVCITEGIPQHDMVRVKAALNKQSKTRLIGPNCPGIIKPGECKIGIMPGYIHKPGRIGIVSRSGTLTYEAVFQTTAVGLGQSTCVGIGGDPFNGTNFVDCIEKFLVDPQTEGIVLIGEIGGTAEEDAAALIKESGTDKPIVGFIAGLTAPPGRRMGHAGAIVSGGKGTAQDKIKTLREAGVTVVESPAKIGVSMLEVFKQRGLVS

>Fragaria vesca (XP_004299462.1)

MARQSVAKLIGSIASRKLSSPSTSTTSIIQRRHYAAAPPPPAVFVDKNTRVICQGITGKNGTFHTEQAIEYGTKMVGGVTPKKGGTEHLGLPVFNSVVEAKAETKANATVIYVPPPFAAAAIMEAMEAELDLVVCITEGIPQHDMVRVKAALNSQSKTRLIGPNCPGIIKPGECKIGIMPGYIHKPGRVGIVSRSGTLTYEAVFQTTAVGLGQSTCVGIGGDPFNGTNFVDCMEKFLADPQTEGIVLIGEIGGTAEEDAAALIKASGTEKPIVAFIAGLTAPPGRRMGHAGAIVSGGKGTAQDKIKTLREAGVTVVESPAKIGVAMLDVFKQRGLVN

>Vitis vinifera 1 (XP_002268479.1)

MLTKMARRAAAQQLIDSIASKLSSATASSAQTRGFAAAAHPPPAVFVDKNTRVICQGITGKNGTFHTEQAIEYGTKMVGGVTPKKGGTEHLGLPVFNTVAEAKAETKANTSVIYVPPPFAAAAIMEAMEAELDLVVCITEGIPQHDMVKVKAALNRQTKTRLIGPNCPGIIKPGECKIGIMPGYIHKPGRVGIVSRSGTLTYEAVFQTTAVGLGQSTSVGIGGDPFNGTNFVDCIKKFLVDPQTEGIVLIGEIGGTAEEDAAQLIKESKTEKPIVAFIAGLTAPPGRRMGHAGAIVSGGKGTAQDKIKTLREAGVTVVESPAKMGVAMLEAFKQKGLIPA

>Vitis vinifera 2 (XP_002271746.1)

MVGQVRRLIPYVVSRIKSPLAPPSRHLGTTAPPAVFVDKNTRVICQGITGKNGTFHTEQAIEYGTKMVGGVTPKKGGMEHLGLPIFNTVAEAKAETRANASVIYVPPPFAAAAIMEAVEAELDLVVCITEGIPQHDMVKVKAALNRQSKTRLIGPNCPGIIKPGECKIGIMPGYIHKPGRVGIVSRSGTLTYEAVFQTTAVGLGQSTCVGIGGDPFNGTNFVDCVKKFLADPQTEGIILIGEIGGTAEEDAAALIKESGTEKPVVAFIAGLTAPPGRRMGHAGAIVSGGKGTAQDKIKTLRDVGATVVESPAKIGVAMLDVFKQRGLVS

>Medicago truncatula (XP_003620150.1)

MTFHRVTKLFSSIASNNRRNFSSTPSPAVFVDKSTRVICQGITGKNGTFHTEQAIEYGTNMVGGVTPKKGGTEHLGLPVFNSVAEAKTETKANASVIYVPPPFAAAAIMEAVEAELDLVVCITEGIPQHDMVRVKAALNRQSKTRLIGPNCPGIIKPGECKIGIMPGYIHKPGRIGIVSRSGTLTYEAVYQTTAVGLGQSTCVGIGGDPFNGTNFVDCLTKFLADPQTEGIILIGEIGGTAEEDAAALIKESGTEKPVVAFIAGLTAPPGRRMGHAGAIVSGGKGTAQDKISTLREAGVTVVESPAKIGAAMFEIFKQKGLVQ

>Glycine max (XP_003548295.1)

MARQALTKLLSSIASRRHHGLRHFSAASPAVFVDKSTRVICQGITGKNGTFHTEQAIEYGTNMVGGVTPKKGGTEHLGLPVFNTVAEAKAETKANASVIYVPPPFAAAAIMEAMEAELDLVVCITEGIPQHDMVRVKAALNRQSKTRLIGPNCPGIIKPGECKIGIMPGYIHKPGRIGIVSRSGTLTYEAVFQTTAVGLGQSTCVGIGGDPFNGTNFVDCLQKFLTDPQTEGIILIGEIGGTAEEDAAALIKESGTEKPVVAFIAGLTAPPGRRMGHAGAIVSGGKGTAQDKIKTLREAGVTVVESPAKIGAAMLDVFKQRGLVQ

>Brachypodium distachyon (XP_003562832.1)

MAASSRRASQLLGSAASRLIHARGYAAAPSPAVFVDKSTRVICQGITGKNGTFHTEQAIEYGTNMVGGVTPKKGGTEHLGLPVFNSVAEAKAETKANASVIYVPPPFAAAAIMEAMEAELDLVVCITEGIPQHDMVKVKAALNRQSKTRLIGPNCPGIIKPGECKIGIMPGYIHKPGRIGIVSRSGTLTYEAVFQTTAVGLGQSTCVGMGGDPFNGTNFVDCLEKFVADPQTEGIVVIGEIGGTAEEDAAAFIQESKTEKPVVAFIAGLTAPPGRRMGHAGAIVSGGKGTAQDKIKALRDAGVTVVESPAKIGSTMFELFKQRGMVE

>Zea mays (NP_001136494.1)

MAASSRRASQLLGSAASRLLLGRGFAAAAAAAPSPAVFVDKSTRVICQGITGKNGTFHTEQAIEYGTNMVGGVTPKKGGTEHLGLPVFNSVAEAKAETKANASVIYVPPPFAAAAIMEAMEAELDLVVCITEGIPQHDMVKVKAALNRQSKTRLIGPNCPGIIKPGECKIGIMPGYIHKPGRIGIVSRSGTLTYEAVFQTTAVGLGQSTCVGIGGDPFNGTNFVDCLEKFVDDPQTEGIVLIGEIGGTAEEDAATFIQESKTQKPVVAFIAGLTAPPGRRMGHAGAIVAGGKGTAQDKIKALREAGVTVVESPAKIGSTMFEIFKQRGMVE

>Ostreococcus tauri (XP_003078495.1)

MRVLALARRARESQRGVANALTRSSIARTADYSTAPAVFVDGNTRVIVQGFTGKNGTFHSEQAIAYGTKVVGGVTPKKGGTTHLGLPVFDTVREAVVETKANASVIYVPPPFAAKAIMEAVEAEIDLVVCITEGVPQHDMVRVKRALMSQSKTRLIGPNCPGIIKPGECKIGIMPGYIHKPGRIGVVSRSGTLTYEAVYQTTVTGLGQSTCVGIGGDPFNGTNFVDCLEKFVKDPQTEGIIMIGEIGGTAEEEAAEFIRASGTDKPIVSFIAGLTAPPGRRMGHAGAIISGGKGGASDKIAALEAAGVRVVKSPAKMGQAMYDMFAERKML

>Chlamydomonas reinhardtii (XP_001693108.1)

MWRLFLTAAGSAKSESSALAGFVRGFASQAAHNPAVFVDKNTKVICQGLTGKNGTFHTEQAIAYGTQMVGGVTPKKGGTSHLGLPVFNTVAEAKAATGCHATVIYVPPPFAAKAILEAVEAELELVVCITEGIPQHDMVRVKKAMQGQTKTRLIGPNCPGIIKPGECKIGIMPGYIHTPGKIGIVSRSGTLTYEAVFQTTNQGLGQSTVVGIGGDPFNGTNFVDCLERFVKDPQTEGIIMIGEIGGTAEEEAAEFIRKSGTKKPVVSFIAGLTAPPGRRMGHAGAIISGGKGTATDKIKALEEAGVTVTRSPAQMGVTMMRVMKERGLA

>Volvox carteri (XP_002957241.1)

MWRLFLNAAGTAKSEGAYAGLVRGFASQAAYNPAVFVDKNTKVICQGITGKNGTFHTEQAIAYGTQMVGGVTPKKGGSTHLGLPVFNSVKEAKSATGCHASVIYVPPPFAARAILEAVEAELDLVVCITEGIPQHDMVKVKKTLQEQSKTRLIGPNCPGIIKPGECKIGIMPGYIHTPGKIGIVSRSGTLTYEAVFQTTNQGLGQSTVVGIGGDPFNGTNFVDCLERFVKDPQTEGIIMIGEIGGTAEEEAAEFIKASGTQKPVVSFIAGLTAPPGRRMGHAGAIISGGKGRASDKIEALESAGVTVTRSPAQMGSTMMRVMRERGLA

>Micromonas pusilla (XP_003054766.1)

MALRRASARALDRIAPPAVFVDKDTKVLVQGFTGKNGTFHSQQAIAYGTRMVGGVTPKKGGSTHLGLPVFNTVAEAKTATGANASAIYVPPPFAAKAILEAVEAELDLVVCITEGIPQHDMIRVKQAMMTQSKTRLIGPNCPGIIKPGECKIGIMPGYIHTPGRIGVVSRSGTLTYEAVYQTTACGLGQSTVVGIGGDPFNGTNFVDCLEKFVKDDQTEGIIMIGEIGGTAEEEAAEFIKASGTTKPVVSFIAGLTAPPGRRMGHAGAIIAGGKGGAGDKIAALKAAGVTVVPSPAGMGEAMHQIFKERKMV

>Saccharomyces cerevisiae (EGA56703.1)

MLRSTVSKASLKICRXFHRESIPYDKTIKNLLLPKDTKVIFQGFTGKQGTFHASISQEYGTNVVGGTNPKKAGQTHLGQPVFASVKDAIKETGATASAIFVPPPIAAAAIKESIEAEIPLAVCITEGIPQHDMLYIAEMLQTQDKTRLVGPNCPGIINPATKVRIGIQPPKIFQAGKIGIISRSGTLTYEAVQQTTKTDLGQSLVIGMGGDAFPGTDFIDALKLFLEDETTEGIIMLGEIGGKAEIEAAQFLKEYNFSRSKPMPVASFIAGTVAGQMKGVRMGHSGAIVEGSGTDAESKKKALRDVGVAVVESPGYLGQALLDQFAKFK

>Aspergillus niger (XP_001398396.1)

MQAVRRNTVAALRNAAATQRRAYSASASPAYAETVNNLRINGDTKVIFQGFTGKQGTFHAEQAIAYGTKVVGGTNPKKAGSMHLDRPVFANVSEAVKETGATATALFVPPPLAAKGIEEAIEAEIPLAVCITEGIPQHDMVRITDILKTQNKTRLVGPNCPGIIAPGQCKIGIMPGFIHKRGRIGIVSRSGTLTYEAVNQTTQAGLGQSLVVGIGGDPFSGTNFIDCLKIFLEDEETDGIIMIGEIGGSAEEDAAEFLKANNKYNKPAVGFIAGISAPPGRRMGHAGAIVSGGKGGADSKIAALEAAGVVVERSPASLGKALLNEFVKRDLV

>Schizosaccharomyces pombe (NP_594230.1)

MFKTQTTLLTSLRRFSSSSQLKNSKSLYEQTIPNLMINSDTKVIFQGFTGKQGTFHAQHAMDYGTKVVGGTNPKKAGTTHLGKPVFGTIEEAMKETKADASAVFVPPPLAAGAIEEAIAAEVPLIVAITEGIPQHDMLRVSDILKTQSKSRLVGPNCPGIIRPGQCKIGIMPSHIHKPGCIGIVSRSGTLTYEAVNQTTQTDLGQSLVIGIGGDPFPGTNFIDALKLFLDDPNTQGIILIGEIGGSAEEDAAEFIRAANASRSTPKPVVSFIAGATAPKGRRMGHAGAIVAGGKGTAAAKFEALEAAGVRISRSPATLGSLIVEELNKLKH

>Candida albicans (EEQ42982.1)

MYRQFTRGFSSSLPARNYASTIKNLKVTKDTKVIYQGFTGKQATFHAEQAIAYGTDVVGGINPRKAGTTHLDRPVFGTVAEAMKEAGASATGIFVPPPLAAAAIEEAIAAEIPLAVAITEGIPQKDMVRISQILKTQEKTRFVGPNCPGLIAPDQCKIGIMPSSIHQRGKVGVISKSGTLTYEAVAQTTAVGLGQSLVIGMGGDPFPGTNFIDALTLYLNDPETEGIIMIGEIGGSAEEEASEFLKQHNLTRPEGPKPVVGFIAGVSAPPGRRMGHAGAIVAGGKGDAKSKIAALESAGVVVEKSPARLGNSLLAEFKNKNLL

>Caenorhabditis elegans (NP_510450.1)

MLSQQIANNARTLQKGAARFYNSTYNNLKINKDTKVIVQGFTGKQGTFHGKQMLEYNTKVVGGVNANKAGTEHLGLPVFKNVSEARNKTGADASVIYVPASAAGSAIEEAMDAEIPLVVCITEGIPQHDMVRVKSRLLKQNKTRLVGPNCPGIISADQCKIGIMPGHIHKRGCIGIVSRSGTLTYEAVHQTTQVGFGQTLCVGIGGDPFNGTNFIDCLNVFLEDPETKGIILIGEIGGSAEEEAAAYLKEHNSGANRKPVVSFIAGVTAPPGRRMGHAGAIISGGKGTAADKINALREAGVVVTDSPAKLGTSMATAFLGKI

>Caenorhabditis elegans (NP_497288.1)

MASTLASAARAATRAAVTRSVYNDTRNNLMINKSTKVIVQGFTGRQGTFHSKQMLEYNTNLVGGVSPNKAGQTHLGLPVFGSVAEAKDRTGADATVIYVPAAGAARAIHEAMDAEIGLIVAITEGIPQQDMVRVKNRLLKQNKSRLLGPNCPGIIASGDCKIGIMPGHIHKKGCIGIVSRSGTLTYEAVHQTTTVGLGQTRCIGIGGDPFNGTNFIDCLEVFLEDEQTKGIILIGEIGGQAEEQAAEFLKSRNSGSNAKPVVSFIAGVTAPPGRRMGHAGAIIAGGKGTAGDKIEALRNANVVVTDSPAKLGVAMQKALLG

>Rattus norvegicus (NP_446204.2)

MTAAVVAAAATATMVSGSSGLAAARLLSRTFLLQQNGIRHGSYTASRKNIYIDKNTKVICQGFTGKQGTFHSQQALEYGTKLVGGTTPGKGGKKHLGLPVFNTVKEAKEKTGATASVIYVPPPFAAAAINEAIDAEIPLVVCITEGIPQQDMVRVKHKLTRQGKTRLIGPNCPGIINPGECKIGIMPGHIHKKGRIGIVSRSGTLTYEAVHQTTQVGLGQSLCIGIGGDPFNGTNFIDCLDVFLKDPATEGIVLIGEIGGHAEENAAEFLKEHNSGPKAKPVVSFIAGITAPPGRRMGHAGAIIAGGKGGAKEKISALQSAGVIVSMSPAQLGTCMYKEFEKRKML

>Mus musculus (BAB23804.1)

MVSSSSGLAAARLLSRTFLLQQNGIRHGSYTASRKHIYIDKNTKIICQGFTGKQGTFHSQQALEYGTKLVGGTTPGKGGQKHLGLPVFNTVKEAKEKTGATASVIYVPPPFAAAAINEAIDAEIPLVVCITEGIPQQDMVRVKHRLTRQGTTRLIGPNCPGVINPGECKIGIMPGHIHKKGRIGIVSRSGTLTYEAVHQTTQVGLGQSLCIGIGGDPFNGTDFIDCLEVFLNDPATEGIILIGEIGGHAEENAAAFLKEHNSGPKAKPVVSFIAGITAPPGRRMGHAGAIIAGGKGGAKEKISALQSAGVVVSMSPAQLGTTIYKEFEKRKML

>Canis lupus (XP_532985.2)

MASGSSGLAAARLLSRSFLLQQNGIRHCSYIASRKHLYVDKNTKVICQGFTGKQGTFHSQQALDYGTQLVGGTTPGKGGKTHLGLPVFNTVKEAKEETGATASVIYVPPPFAAAAINEAVEAEVPLVVCITEGIPQQDMVRVKHKLLRQGKTRLIGPNCPGIINPGECKIGIMPGHIHKKGRIGIVSRSGTLTYEAVHQTTQVGLGQSLCVGIGGDPFNGTDFIDCLEIFLNDPATEGIILIGEIGGNAEENAAEFLKQHNSGPKAKPVVSFIAGLTAPPGRRMGHAGAIIAGGKGGAKEKISALQSAGVVVSMSPAQLGTTIYKEFEKRKLL

>Homo sapiens (NP_003840.2)

MTATLAAAADIATMVSGSSGLAAARLLSRSFLLPQNGIRHCSYTASRQHLYVDKNTKIICQGFTGKQGTFHSQQALEYGTKLVGGTTPGKGGQTHLGLPVFNTVKEAKEQTGATASVIYVPPPFAAAAINEAIEAEIPLVVCITEGIPQQDMVRVKHKLLRQEKTRLIGPNCPGVINPGECKIGIMPGHIHKKGRIGIVSRSGTLTYEAVHQTTQVGLGQSLCVGIGGDPFNGTDFIDCLEIFLNDSATEGIILIGEIGGNAEENAAEFLKQHNSGPNSKPVVSFIAGLTAPPGRRMGHAGAIIAGGKGGAKEKISALQSAGVVVSMSPAQLGTTIYKEFEKRKML

>Escherichia coli (AAC73823.1)

MSILIDKNTKVICQGFTGSQGTFHSEQAIAYGTKMVGGVTPGKGGTTHLGLPVFNTVREAVAATGATASVIYVPAPFCKDSILEAIDAGIKLIITITEGIPTLDMLTVKVKLDEAGVRMIGPNCPGVITPGECKIGIQPGHIHKPGKVGIVSRSGTLTYEAVKQTTDYGFGQSTCVGIGGDPIPGSNFIDILEMFEKDPQTEAIVMIGEIGGSAEEEAAAYIKEHVTKPVVGYIAGVTAPKGKRMGHAGAIIAGGKGTADEKFAALEAAGVKTVRSLADIGEALKTVLK

>Parvibaculum lavamentivorans (YP_001412730.1)

MSVLVDKNTKVICQGFTGNQGTFHSEQAIAYGTKMVGGVSPGKGGSKHLDLPVFNTVAEAVEKTGATASAIYVPPPFAADAILEAIDAGIELAVCITEGIPVLDMVKVKRALQGSNTRLVGPNCPGVITPDECKIGIMPGHIHKPGSVGIVSRSGTLTYEAVAQTTAAGLGQSTCIGIGGDPVNGTNFIDCLEMFLGDPDTTSIIMIGEIGGSAEEDAAEFLKASKVKKPIVGFIAGVTAPPGRRMGHAGAIISGGKGGAEDKMEAMRSAGITVAKSPSALGTTLVEVLKG

>Candidatus Puniceispirillum (YP_003552160.1)

MILRMPPPRLLQLSNKEADMSVLVNKDTRVICQGFTGAQGTFHSEQAIAYGTKMVGGVTPGKGGQTHLDLPVFNSVVEAMRETEANASVIYVPPPFAADSIMEAIDAGMPLIVCITEGIPVLDMVKVKRALDASKSRLIGPNCPGIITPGECKIGIMPGHIHKPGKVGIVSRSGTLTYEAVAQTTAAGLGQSTCVGIGGDPVNGTNFIDCLEMFLADDQTEAIVMIGEIGGSAEEDAAAYYMAQPNRKPIAGFIAGQTAPPGRRMGHAGAIVSGGSGAAVDKIAAMETAGFVMASSPAALGDAVMTAIGNHSS

>Rickettsia bellii (YP_001495655.1)

MAILVNKKTKVICQGFTGSQGTFHSEQAIAYGTKMVGGVTPGKGGKTHLDLPIYNTVHEAKAKTGANASVIYVPPPFAADSILEAIDAGIEIVVCITEGIPVLDMVRVKRALVGSRTRLIGPNCPGIITPDECKIGIMPGHIHRKGSIGIVSRSGTLTYEAVAQTTAVGLGQSTCVGIGGDPINGTNFVDCIDMFLQDDETKAIIMIGEIGGDAEENAADFIKHSKIKKPIVSFIAGITAPPGKRMGHAGAIIAGGKGSAEDKLEALQSAGVTITRSPADIGKTMLDLLNG

>Micavibrio aeruginosavorus (YP_004864987.1)

MAVLVDKNTKVICQGFTGAQGTFHSEQAIAYGTKMVGGVTPGKGGTKHLNLPVFDTVAQAKAETGCNASVIYVPPPFAADAILEAIDAGIELAICITEGIPVLDMVRVRRALDGSNTRLIGPNCPGIITPDECKIGIMPGHIHKRGKIGVVSRSGTLTYEAVAQTTAAGLGQSTCIGIGGDPVNGTNFIDCLKMFHDDPETEAIIMIGEIGGSAEVDATEYYKSLTNKKPIAGFIAGVTAPKGKRMGHAGAIISGGADTAEAKIAAMKAAGFVVADSPASLGQAVQEAMKKAA

>Anabaena variabilis (YP_322301.1)

MNLTPDSKVIIQGFHEFISENHIAQMKAYGTNLVAGVQPGYGGQELYHLPVFDLVEEVVGQFGSIDTTIICVHPYQVLDAALEAIASHIRQIIIISAGVPPLDMVELLRKAEAGETLVVGPNSPGIIVPGKILLGTQPSEFYTPGVVGIVSRSSTLTYEVARELTQAGWGQSISVSIGSDAIVGSSFLQWLQILDEDETTEAIVLVGQPGGGSEEAAARYITEAIDKPVIAYIAGRQAPPGKTWRQTGTLATVVGRPPNFGTAQSKITAFEAAEVPVAERPSLIPQLLKKAIH

>Nostoc punctiforme (YP_001868459.1)

MNLTPESKVLIQGFCEFISGTHVAQMQAYGTNLVAGVNPGCGGQELHGLPVFDLVEEVIEQFGVIDTTIICVDPYDVLDAALEAIASHISQIIIITAGVPPLDMVQLLRKAEACETLVIGPNSPGIIVPGKILLGTQPSEFYTPGVVGIVSRSSTLTYEVAYELTKAGLGQSISVSIGSDAIVGSSFLQWLQILDEDETTEAIVLVGQPGGGSEEAAARYIAEAIDKPVIAYIAGRQAPPGKSWRQTGTLATVIGRDPNFGTAQSKLAALKEAEVPVAERPSQIPELLRKEIKIN

>Cylindrospermum stagnale (YP_007146430.1)

MNLTPDSKVLIQGFSEFISETHVAQMKAYGTNLIAGVNPGCGGQLLYDLPIFDLVEEVVAQFGAIDTTIICVHPYQVLDAALEAIASNIRQIIIISGGVPPLDMVQLLRKAEACETIVVGPNSPGIIVPGKILLGTHPSELYTPGHVGIVSRSSTLTYEIAWELTKAGMGQSISASIGSDAIIGSSFLQWLQILDEDEATQAIVLVGQPGGGSEEAAAQYITETIDKPVIAYIAGRHAPPAKHWRQTGTLATFVGRDPSYGTAQSKLAAFQTAQVSVAERPSEIPELLKKVLK

>Nodularia spumigena (ZP_01629428.1)

MNLTPDSKVLIQGFSEFISATHIAQMKAYGTNLVAGVNPGCGGQKLHNLPIFDLVEEVVAKFGAIDTAIICVHPYQVLDAALEAIACNINQIIIITGGVPPLDMVQLLRKAETGETLIVGPYSPGIIVPGKILLGTQPSELYTPGTVGIVSRSSTLTYEVAWELTKAGLGQSISVSIGSDAIVGSSFLQWLQILDEDDTTQAIVLVGQPGGDSEEAAARYIIEAIDKPVIAYIAGRHAPRAKNWRQSGTLATVIGRDPNFGTAQSKLAAFKEAKVPVAERPSLIPELVKKGIKLVEKSH

BETA CHAIN

>Arabidopsis thaliana (NM_127601.3)

MRGLVNKLVSRSLSISGKWQNQQLRRLNIHEYQGAELMGKYGVNVPKGVAASSLEEVKKAIQDVFPNESELVVKSQILAGGRGLGTFKSGLKGGVHIVKRDEAEEIAGKMLGQVLVTKQTGPQGKVVSKVYLCEKLSLVNEMYFSIILDRKSAGPLIIACKKGGTSIEDLAEKFPDMIIKVPIDVFAGITDEDAAKVVDGLAPKAADRKDSIEQVKKLYELFRKTDCTMLEINPLAETSTNQLVAADAKLNFDDNAAFRQKEVFAMRDPTQEDPREVAAAKVDLNYIGLDGEIGCMVNGAGLAMATMDIIKLHGGTPANFLDVGGNASEHQVVEAFKILTSDDKVKAILVNIFGGIMKCDVIASGIVNAAKEVALKVPVVVRLEGTNVEQGKRILKESGMKLITADDLDDAAEKAVKALAH

>Arabidopsis lyrata (XP_002884202.1)

MRGLVNKLVSRSLSISGKWQQQQLRRLNIHEYQGAELMGKYGVNVPKGVAASSLEEVKKAIQEVFPNESELVVKSQILAGGRGLGTFKSGLKGGVHIVKRGQAEDIAGKMLGQVLVTKQTGPQGKVVSKVYLCEKLSLVNEMYFSIILDRKSAGPLIIACKKGGTSIEDLAEKFPDMIIKVPIDVFAGITDEDAAKVVDGLAPKAADRKDSIEQVKKLYELFRKTDCTMLEINPLAETSTNQLVAADAKLNFDDNAAFRQKEIFAMRDPTQEDPREVAAAKVDLNYIGLDGEIGCMVNGAGLAMATMDIIKLHGGTPANFLDVGGNASEHQVVEAFKILTSDDKVKAILVNIFGGIMKCDVIASGIVNAAKEVSLKVPVVVRLEGTNVEQGKRILKESGMKLITADDLDDAAEKAVKALAN

>Solanum lycopersicum (NP_001234293.1)

MLRKLANQSLSVAGKWQQQQLRRLNIHEYQGAELMSKYGINVPKGVAVASLDEVKKAIQDVFPNQSEVVVKSQVLAGGRGLGTFKNGFQGGVHIVKADQAEDIASKMLGQILVTKQTGAQGKVVSKVYLCEKMSLVNEMYFSIILDRATAGPLIIACRKGGTSIEDLAEKFPDMIIKVPIDVFKGISDADAAKVVDGLAPKVADRNDSIEQVKKLYKLFCETDCTMLEINPLAETSDNKLVAADAKLNFDDNAAYRQKEIFSLRDSSQEDPREVAAAKADLNYIGLDGEIGCMVNGAGLAMATMDIIKLHGGTPANFLDVGGNATEGQVVEAFKILTADEKVKAILVNIFGGIMKCDVIASGIVNAAKQVQLKVPVIVRLEGTNVEQGKRILKESGMKLITAEDLDDAAEKAVKALA

>Cucumis sativus (XP_004140571.1)

MVRGLLNKLVSRSLSVAGKWQHQQLRRLNIHEYQGAELMSKYGINVPKGLAVSSVDEVKSAMQAAFPDAKELVVKSQILAGGRGLGTFKSGLKGGVHIVKVDQVEEIAGKMLGQILVTKQTGPQGKVVSKVYLCEKLSLTNEMYFAITLDRKTAGPLIIACRKGGTSIEDLAENFPDMIIKVPIDVFQGITDEDAAKVVDGLAPKGADRQDSIEQVKKLYKLFCECDCTLLEVNPMAETSDNQLVAADAKLNFDDNAAFRQKAIFALRDPTQEDPREVDAAKADLNYIGLDGEIGCMVNGAGLAMATMDIIKLHGGTPANFLDVGGNASEGQVVEAFKILTSDEKVKAILVNIFGGIMKCDVIASGIVNAAKQVALKVPVIVRLEGTNVDQGKRILKESGMTLITAEDLDDAAEKAVKAAY

>Fragaria vesca (XP_004289972.1)

MVRGLLKQLSSRSLSFAGKWQQQQLRRLNIHEYQGAELMSKYGVNVPKGVAVGSVDEVKKVIESTFPNQSELVVKSQVLAGGRGLGTFKNGLKGGVHIVKADQVEDLAGKMLGQILVTKQTGAEGKIVSKVYLCEKLSLVNEMYFAITLDRKSAGPLIIACAKGGTSIEDLAEKFPDSIVKVPIDVFKGITDEDAAKVVDGLSPKGADRNSSIEQVKKLYKLFSETDCTLLEINPLAETADNQLVAADAKLNFDDNAAFRQKEIFALRDPTQEDPREVAAAKADLNYIGLDGEIGCMVNGAGLAMSTMDIIKLHGGTPANFLDVGGNASEGQVVEAFKILTSDDKVKAILVNIFGGIMKCDVIASGIVNAAKQVQLKVPVVVRLEGTNVDQGKRILKESGMALITAEDLDDAAKKAVEAISK

>Vitis vinifera (XP_002275953.1)

MVRGLVNKLVSRSLNVAGKWQQQQLRRLNIHEYQGAELMSKYGINVPKGVAVGSIEEVRKTIQDVFPKENELVVKSQILAGGRGLGTFKSGLKGGVHIVKTDQVEELAGKMLGQILVTKQTGPQGKVVSKVYLCEKMSLVNEMYFAITLDRSSAGPIIIACRKGGTSIEDLAEKFPDMIVKVPIDVFQGITDEDAAKVVDGLAPKVADRNASIEQVKKLYKLFCECDCTLLEINPIAETSDNQLVAADAKLNFDDNAAFRQKHIFALRDATQEDPREVAAAKADLNYIGLDGEIGCMVNGAGLAMATMDIIKLHGGTPANFLDVGGNASESQVVEAFKILTADDKVKAILVNIFGGIMKCDIIASGIVNAAKEVSLKVPVVVRLEGTNVDQGKRILKESGMALITAEDLDDAAEKAVKASGTC

>Glycine max (XP_003537078.1)

MVRGLLNKLVSRSLSVAGKWQHNQLRRLNIHEYQGAELMSKHGVNVPRGVAVSSVEEARKVIKDLFPNENELVVKSQILAGGRGLGTFKSGLKGGVHIVKTDQVEDIAGKMLGQILVTKQTGPQGKIVSKVYLCEKLSLVNEMYFAITLDRTSAGPIIIACSKGGTSIEDLAEKFPDMIIKVPVDVFEGITDEGAAKVVDGLALKVADRNKSIEQVKNLYKLFVDCDCTLLEINPIAETADNQLVAADAKLNFDDNAAYRQKEIFSLRDTTQEDPREVTAAKADLNYIGLDGEIGCMVNGAGLAMATMDIIKLHGGTPANFLDVGGNASENQVVEAFKILTADDKVKAILVNIFGGIMKCDVIASGIVNAAKEVALKVPVVVRLEGTNVDQGKRILKESGMALITAEDLDDAAQKAVKAAYK

>Brachypodium distachyon (XP_003575382.1)

MVRGSLGKLASRALSVAGKWQHQQLRRLNIHEYQGAELMGKYGINVPKGAVVGSVQEVKEVLNKVFPSEKEIVVKSQILAGGRGLGTFKSGLQGGVHIVKAEEAEGLAAKMLNQILVTKQTGPQGKVVGKVYLCEKMSLVNEMYFAITLDRKTAGPLIIACSKGGTSIEDLAEKYPDMIIKVPIDVFKGITDEDAGKVVDGLAPKTADRQASIEQIKKLYELFCKSDCTMLEINPLAETADKKLVAADAKLNFDDNAAFRQKEIFALRDTTQEDPREVAAAKADLNYIGLDGEIGCMVNGAGLAMATMDIIKLHGGTPANFLDVGGSASEGQVVEAFKILTSDDRVKAILVNIFGGIMKCDVIASGIVNAAKQVDLKVPVVVRLEGTNVDQGKRILKESGMALITAEDLDDAAEKAVKAYIK

>Zea mays (NP_001150653.1)

MVRGSLGKLASRALSVAGRWQHQQLRRLNIHEYQGAELMGKYGINVPRGAAAGSVQEVNDALKNMFPSEKEIVVKSQILAGGRGLGTFKSGLQGGVHIVKAEEAESIARKMLGQILVTKQTGPEGKIVSKVYLCEKLSLTNEMYFAITLDRKTAGPLIIACSKGGTSIEDLAEKYPDMIIKVPVDVFKGITDEDAAKVVDGLALKSADRQSSIEQIKKLYELFCKSDCTLLEINPLAETADNKLVAADAKLNFDDNAAFRQKEIFALRDTTQEDPREVAAAKADLNYIGLDGEIGCMVNGAGLAMATMDIIKLHGGTPANFLDVGGSASEGQVVEAFKILTSDDRVKAILVNIFGGIMKCDVIASGIVNAAKQVDLKVPVVVRLEGTNVDQGKRILKESGMTLITAEDLDDAAEKAVKASVK

>Escherichia coli (AAC73822.1)

MNLHEYQAKQLFARYGLPAPVGYACTTPREAEEAASKIGAGPWVVKCQVHAGGRGKAGGVKVVNSKEDIRAFAENWLGKRLVTYQTDANGQPVNQILVEAATDIAKELYLGAVVDRSSRRVVFMASTEGGVEIEKVAEETPHLIHKVALDPLTGPMPYQGRELAFKLGLEGKLVQQFTKIFMGLATIFLERDLALIEINPLVITKQGDLICLDGKLGADGNALFRQPDLREMRDQSQEDPREAQAAQWELNYVALDGNIGCMVNGAGLAMGTMDIVKLHGGEPANFLDVGGGATKERVTEAFKIILSDDKVKAVLVNIFGGIVRCDLIADGIIGAVAEVGVNVPVVVRLEGNNAELGAKKLADSGLNIIAAKGLTDAAQQVVAAVEGK

>Rhodobacter capsulatus (YP_003576892.1)

MNIHEYQAKQLLKSYGCPVSNGRIVLKADEVKAAASELDGPLWVVKAQIHAGGRGKGHFKEAEAGEKGGVRLARSVAEAETLARQMLGRTLVTHQTGPAGKQVNRIYIEEGSDISRELYLALLIDRKSSRVSFVCSTEGGMDIEEVAAHTPEKILSFSVDPATGLSDFHGRRIAFALGLEGNQVKQCVSLIKKLYKAFLEKDMEMLEINPLIVTTDGQLKVLDAKLGFDNNALYRQPDVMDLRDVTEEDPKELEASRFDLNYIALDGEIGCMVNGAGLAMATMDIIKLYGAEPANFLDVGGGATKEKVTEAFKIITSDPKVKGILVNIFGGIMRCDIIAEGVLAAVKEVGLKVPLVVRLEGTNVELGKDIIAKSGLNVIAADDLADGAKKIVAAVKG

>Caulobacter crescentus (NP_419156.1)

MNIHEHQAKAVLAEFGAPVPRGFAAFTPDEAAAAAEKLGGPVFVVKSQIHAGGRGKGKFEGLGPDAKGGVRVVKSVEEVRSNAEEMLGRVLVTHQTGPKGKQVNRLYIEEGAAIAKEFYLSLLVDRASSKVSVVASTEGGMDIEDVAHSTPEKIHTFTIDPATGVWPTHHRALAKALGLTGGLAKEAASLLNQLYTAFMAKDMAMLEINPLIVTADDHLRVLDAKLSFDGNSLFRHPDIKALRDESEEDPKEIEASKYDLAYIALDGEIGCMVNGAGLAMATMDIIKLYGAEPANFLDVGGGASKEKVTAAFKIITADPAVKGILVNIFGGIMRCDIIAEGVIAAVKEVGLQVPLVVRLEGTNVELGKKIISESGLNVIAANDLSDGAEKIVAAVKGAR

>Tistrella mobilis KA081020-065 (YP_006372430.1)

MNIHEYQAKALLAKYGVAVPRGGVAYTPEEAVEVAKSLGGPVWVVKAQIHAGGRGKAGGVKVVKSLDDVAATAKNMLGMTLVTHQTGPEGKEVKRVYIEEGCDIRRELYLGVVIDRETSSVTFMASTEGGMEIEEVAKETPEKIIKVPVDVITGMQPFHARKIAYGLGLEGKQVSVATKFMLAMYQAVIGTDASIVEINPLVVTGAGDVIALDAKMNFDDNALFRHKDIEDLRDESEEDPSELEASRHALNYIKLDGNIGCMVNGAGLAMATMDIIKLYGGEPANFLDVGGGATRERVTTAFKLILSDPNVEGILVNIFGGIMRCDVIAEGVVAAAREISLQVPLVVRLEGTNVEKGKAILANSGLPIMSADDLADAAEKIVKAVKEAA

>Magnetococcus marinus (YP_865661.1)

MNIHEYQAKEIIAKFGVNVPRGRVAYTPQEAEMAAKELDSEVFVVKAQIHAGGRGKAGGVKVVKTTDAVREEASRMLGLTLVTHQTGPEGKEVKRLYVEEGCDIARELYLGIVIDRATSRVTFMASTEGGMEIEEVAEKSPEKILREVVDPAVGLVAFQARNLAFGLGLEGKQINKAVQLMSGLYNAFMETDASLMEINPLVITGAGDVIALDCKMNFDANALYRQKAIEELRDFDEENPKEIEASKHELNYIALDGNIGCMVNGAGLAMATMDIIKIKGGAPANFLDVGGGATTERVTEAFKLILSDENVKAVLVNIFGGIMRCDVIANGVVEAAKQVNLNVPLIVRLEGTNVEKGKEILANSGLPIISADNLDDAATKAVASLKEG

>Saccharomyces cerevisiae (NP_011760.3)

MYSRKSLSLISKCGQLSRLNAQAALQARRHLSIHEYRSAQLLREYGIGTPEGFPAFTPEEAFEAAKKLNTNKLVIKAQALTGGRGKGHFDTGYKSGVHMIESPQQAEDVAKEMLNHNLITKQTGIAGKPVSAVYIVKRVDTKHEAYLSILMDRQTKKPMIIASSQGGMNIEEVAERTPDAIKKFSIETSKGLSPQMAKDVAKSLGFSPDAQDEAAKAVSNLYKIFMERDATQVEINPLSEIEHDPTHKIMCTDAKFGFDDNASFRQEKIYSWRDLSQEDPDEVKAKKYDLNFVKLKGNIGCLVNGAGLAMATMDVIKLNGGDPANFLDCGGGATPETIKQGFELILSNKNVDAIFVNIFGGIVRCDYVALGLVEAARELEVRVPIVARLQGTKVEEGRDIINKSGVKIYSFDELDPAAKKVVELTQN

>Schizosaccharomyces pombe (NP_588466.1)

MLTRSVLRKAPRAFSPFLQKRNLALHEYISHDILRKFGVDVPRGAPARSGEEAEKVARDLKVTDLVVKAQVLAGGRGKGQFDSGLRGGVRPVYDATEARMFAEQMIGHKLITRQTGPAGKICNVVYVCERKFIRKEYYFAILMDRENQCPMIVASDQGGVDIETVAAENPSAIIKRSLPNSPNLDPHIAEELVDKLGFSSSSKPKAVDAIVKLYKVFNDCDATQVEINPLAETTDHKVLCMDAKLNFDDNAEFRHSNIFVLRDISQEDPDEARAAKVGLNFIKLDGNIGCLVNGAGLAMATMDIIKLHGGEPANFLDVGGNANAEAIREAFSLITNDPKTTAIFVNIFGGIVRCDVIAKGLISVVSALNLNIPIICRLQGTNQGAAKEVINNSGLRIFSFDDLDEAAKKACRFSRVVEMAREADVNVSFELPL

>Candida albicans (EEQ43728.1)

MLSRSFARISRSAAQQKRFLSLHEYRSAALLSEYGVPIPKGYPATTPEGAYDAAKKLGTNELVIKAQALTGGRGKGHFDSGLQGGVKLISSAEEAKDLASQMLNHKLITKQTGAAGKEVTAVYIVERRDAASEAYVAILMDRTRQTPVIVASAQGGMDIEGVAAKDPSAIKTFPVPLEEGVSDSLATEIAGALGFTQDAIPEAAKTIQSLYKCFIERDCTQVEINPLSETPDHKVLAMDAKLGFDDNASFRQEEVFSWRDPTQEDPQEAEAGKYGLNFIKLDGNIANIVNGAGLAMATMDIIKLYGGEPANFLDCGGTATPETIEKAFELILSDPKVNGIFVNIFGGIVRCDYVAKGLIAATKNFNLDIPVVVRLQGTNMAEAKELIDNSGLKLYAFEDLDPAAEKIVQLAPKNN

>Aspergillus niger (XP_001400660.1)

MFKLARSKPVTAALRAATESSVQSRVAQQQRNLSIHEYLSANLLKSYGIGMPKGEVARSAEEAEAVAKSLGNDDMVIKAQVLAGGRGKGTFDNGLKGGVRVIYSPTEAKMFASQMIGQKLITKQTGAAGRLCNSVYICERKFARREFYLAVLMDRASQSPVIVASSQGGMDIEAVAKENPEAIITTPIDIKVGVTDAIAHKIVTELGFSEQCVEEAKDTIQKLYKVFMEKDATQIEINPLSETSDHKVMAMDAKLGFDDNAEFRQKEVFSWRDTTQEDADEVKAAEHGLNFIKLDGDIGCLVNGAGLAMATMDIIKLNGGAPANFLDVGGGATPAAIKSAFELITSDPKVTAIFVNIFGGIVRCDAIAQGLINVVQEMGLRTPIVARLQGTNMEQAHKLINESGLKIFSIEDLQSAAEKSVQFSKVVKMAREIDVGVEFTLGI

>Caenorhabditis elegans (NP_501266.1)

MLRAAGNLSKSMMKSQRRFLNLQEFQSKEILEKHGCSVQNFVVASNRKEAEEKWMSFGDHEYVVKAQILAGGRGKGKFINGTKGIGGVFITKEKDAALEAIDEMIGKRLVTKQTTSEGVRVDKVMIAEGVDIKRETYLAVLMDRESNGPVVVASPDGGMDIEAVAEKTPERIFKTPIDIQMGMTEGQSLKIAKDLQFEGKLIGVAAQEIKRLYDLFIAVDATQVEINPLVETADGRVFCVDAKMNFDDSAAYRQKEIFAYETFEEHDPREVDAHQFNLNYIGMDGNIACLVNGAGLAMATMDLIKLHGGEPANFLDVGGAVTEDAVFNAVRIITSDPRVKCVLINIFGGIVNCATIANGVVSAVNKIGLNVPMVVRLEGTNVDAAKQIMKKSGLKILTANNLDEAAAKAVSSLPK

>Caenorhabditis elegans (NP_509821.1)

MIGRISQPLLNTSQKFMAPAARTLMLHEHHGMKILQNYEIKVPPFGVAQDAETAFSEAKRIGGKDYVVKAQVLAGGRGKGRFSSGLQGGVQIVFTPDEVKQKAGMMIGANLITKQTDHRGKKCEEVMVCKRLFTRREYYFSITLDRNTNGPIVIASSQGGVNIEEVAATNPDAIVKMPIDVNVGITKELAHEIAVKMGFSKDCEQQASEIIEKLYQMFKGSDATLVEINPMAEDVNGDVYCMDCKLLLDSNAEFRQAKLFDLKDKKQEDELEIRAAAANLNYIRLDGTIGCMVNGAGLAMATMDIIKLHGGEPANFLDVGGGATVEQVTEAFKIITADKDKVSAILVNIFGGIMRCDVIAQGIIQAARELDLKIPIVVRLQGTKVEDAKALIATSQLRILPCDNLDEAAKMVVKLSNIVDLARATNVDVKFELSI

>Rattus norvegicus (NP_001094220.1)

MASAVAIAAQAGKLLRERSLRPRLLAVRSQAGHLTPRRWLNLQEYQSKKLMSEHGVRVQRFFVASTAKEAQEAAKRLNAKEIVLKAQILAGGRGKGVFDSGLKGGVHLTKDPKVVGQLAQQMIGYNLATKQTPKEGVKVNKVMVAEALDISRETYLAILMDRSHNGPVLVGSPQGGVDIEEVAASSPELIFKEQIDIFEGIKDSQAQRMAENLGFLGSLKNQAADQIKKLYHLFLKIDATQVEVNPFGETPEGQVVCFDAKINFDDNAEFRQKDIFAMDDKSENEPIENEAARYDLKYIGLDGNIACFVNGAGLAMATCDIIFLNGGKPANFLDLGGGVKESQVYQAFKLLTSDPKVEAILVNIFGGIVNCAIIANGITKACQELELKVPLVVRLEGTNVQEAQNILKSSGLPITSAVDLEDAAKKAVASVAKK

>Mus musculus (NP_035637.2)

MASPVAIAAQAGKLLRERALRPLLAVRSQAGHLTPRRWLNLQEYQSKKLMSEHGVRVQRFFVANTAKEALEAAKRLNAKEIVLKAQILAGGRGKGVFNSGLKGGVHLTKDPKVVGELAQQMIGYNLATKQTPKEGVKVNKVMVAEALDISRETYLAILMDRSHNGPVIVGSPQGGVDIEEVAASSPELIFKEQIDIFEGIKDSQAQRMAENLGFLGSLKNQAADQITKLYHLFLKIDATQVEVNPFGETPEGQVVCFDAKINFDDNAEFRQKDIFAMDDKSENEPIENEAARYDLKYIGLDGNIACFVNGAGLAMATCDIIFLNGGKPANFLDLGGGVKEAQVYEAFKLLTSDPKVEAILVNIFGGIVNCAIIANGITKACRELELKVPLVVRLEGTNVQEAQNILKSSGLPITSAVDLEDAAKKAVASVAKK

>Canis lupus (XP_533767.1)

MASPAAARARTLLRELVLRPPLLAARWQAVQLTPRRWLNLQEYQSKKLMSDNGVKVQRFFVADTANEALEAAKKLNAKEFVLKAQILAGGRGKGVFSSGLKGGVHLTKDPQVVGQLAKQMIGYNLATKQTPKEGVKVNKVMVAEALDISRETYLAILMDQSCNGPVLVGSPQGGVDIEEVAASNPELIFKEQIDIMEGIKDSQAQRMAENLGFLGPLKNQAADQIKKLYNLFLKIDATQVEVNPFGETPEGQVVCFDAKINFDDNAEFRQKDIFAMDDKSENEPIENEAAKYDLKYIGLDGNIACFVNGAGLAMATCDIILLKGGKPANFLDLGGGVKEAQVYQAFKLLTADPKVEAILVNIFAGIVNCAIIANGITKACQELELKVPLVVRLEGTNVHEARNILSNSRLPITSAVDLEDAAKKSVASVAKK

>Homo sapiens (NP_003839.2)

MASPVAAQAGKLLRALALRPRFLAAGSQAVQLTSRRWLNLQEYQSKKLMSDNGVRVQRFFVADTANEALEAAKRLNAKEIVLKAQILAGGRGKGVFNSGLKGGVHLTKDPNVVGQLAKQMIGYNLATKQTPKEGVKVNKVMVAEALDISRETYLAILMDRSCNGPVLVGSPQGGVDIEEVAASNPELIFKEQIDIFEGIKDSQAQRMAENLGFVGPLKSQAADQITKLYNLFLKIDATQVEVNPFGETPEGQVVCFDAKINFDDNAEFRQKDIFAMDDKSENEPIENEAAKYDLKYIGLDGNIACFVNGAGLAMATCDIIFLNGGKPANFLDLGGGVKEAQVYQAFKLLTADPKVEAILVNIFGGIVNCAIIANGITKACRELELKVPLVVRLEGTNVQEAQKILNNSGLPITSAIDLEDAAKKAVASVAKK

>Ostreococcus tauri (XP_003078975.1)

MRYGGDETEASRGRRAAPTREREPTTPTRAARLRRETNDFGERSWKIYERSSNGRARRLTGGVFAGRRVASPSSVRRRGASYSRPCVXXXXXXXDNAEYRQRETFALRDHSQEDAREVQAGKFDLNYIGLDGNIGCMVNGAGLAMATMDIIQLYGGSPANFLDVGGNASEEQVVEAFKILTADPKVKAILVNIFGGIMKCDVIAAGIVAAAKQVDLRVPLIVRLEGTNVEAGNAILSSSDLTIITASDLDDAAQKACASLK

>Micromonas pusilla (XP_003059577.1)

MLRASLATLRRGARAGVASPATQLQQIRRLNIHEYQARSAELMAQYGVRVPPGIACTTPDEVAAAAAKLSGESGEVVVKSQVLAGGRGLGTFTSGLKGGVHIVPAAEAKTLSEKMLGQTLVTKQTGAAGKPVNTLMVAEKMKLVNEMYFAILLDRASAGPMIIACSEGGTSIEDLAESHPEKIIKMKVDVNRGLTTDQALELARGLKVSGSADDAAAQLKALYAVFEKSDCTMLEVNPLAETDEKLLIAADAKLNFDDNAEYRQRELFALRDHSQEDPREVAAGKFDLNYIGLDGNIGCMVNGAGLAMATMDIISLHGASPANFLDVGGNASEEQVVEAFKILTADVKVKALLVNIFGGIMKCDVIASGIVNAAKQVGIKVPLVVRLEGTNVEAGKEILAESGLNIVSADDLDDAAKKAVAALA

>Chlamydomonas reinhardtii (XP_001691581.1)

MLPALTGKLLKQATGSLAALSQCQQLRFFNVHEYQGAQIMSKFGVNVPPGIPATTLDEVKKAVDQMADEKGEVVIKSQILAGGRGLGKFTNGLQGGVHIVPKAKALELAKQMLGATLVTKQTGPAGKPVNTLLISKKMKLKREMYFAILLDRKTAGPMMIGCSEGGTSIEDLAEKFPEKIIKIPIDIRVGITDAQAMQMVEGLRVTGDKAAAAKQIKALYTLFDKSDCTMVEVNPLAEGLDGSLIAADAKLGFDDNAAYRQKPIFDMKDESQIDPREVAASKYDLNYIGLDGSIGCMVNGAGLAMATMDIIKMHGGAPANFLDVGGSANEQQVVEAFKILTGDKQVKAILVNIFGGIMKCDVIASGIVNAAKQVGVQVPLVVRLEGTNVARGKEILATSGMTIITADDLDDAAKKAVASIA

>Volvox carteri (XP_002949757.1)

MLASLVRVFAKQGLGSLGALSQCQQLRFFNVHEYQGAQLMSKFGVNVPPGIPAKTLDEVAQAVEQMADENGEVVLKSQILAGGRGLGRFTNGLQGGVHIVPKARALELAKQMLGGTLVTKQTGPAGKPVNTLLIARKMKLRREMYFAILLDRKTAGPMMIGCSEGGTSIEDLAEKYPEKIIKVPVDSRTGITDAQAAKMVEGLRVTGDKAKAAQQIKALYDLFVKCDCTMVEVNPLAESLDGQLIAADAKLGFDDNAAYRHKEIFDLKDESQIDPREVAAAKYDLNYIGLDGSIGCMVNGAGLAMATMDIIKMHGGSPANFLDVGGSANEQQVVEAFKILTSDKQVKAILVNIFGGIMKCDVIARGIVNAAKQVGVSVPLVVRLEGTNVAQGKDIIRSSGMAIIAADDLDDAAKKAVAAIA

>Microcystis aeruginosa (ZP_16389818.1)

MDLLEYQAKEIFAQVGIPILPSQPIHEPGGLKRLNIPYPIVLKSQVRTGGRGKAGGVRFVANTIDAIAAAHAIFHLPIAGEYPEVILAEARYNPQQEIFLAILLDYHLQRPVLLGASQGGMDVDSLLATMQKVVIEERFSPYLCRQLAVSMGLRGSLIESISQILEKMYSLFVSKDLDIIEINPLGINEAGEVMALDGKISVNDAALARHLDLLALTPPQLQSPWSIIDQTGRIAVISNGQGLMSSIWDVLAGQGAKLAAWLILEERLELEQLNEQIEAGLQQFQLLADLKVVIVDIVSYPDFGQKAIETISNYYRSYSPLSPSRGSGERTIRATRQERQTLEPRSFLNPTLPQIIFRVLADNQVIDLSQSLADVNFHWLESLEEVVTAAIKLA

>Crocosphaera watsonii (ZP_00518900.1)

MDLLEYQAKELFHQVGIPVLPSQPIAKLSELKHLHIPYPVVLKSQVHSGGRGRAGGIRFVQNTIDAVAAAQAIFSLPILKEYPEVILAEARYDAQEEFFLSIVLDYQLQRPVLMGSAKGGIDVETLLKHTQKVVLHQGFSPFYARRLATKMGLQGRLIHGVSIILEKMYQLFIEKDLDLVEINPLAVSSSGEFMALDGKITVNDMALSRHLDLLSFLKPRVDQPSSQTPQATIVTTPPQKPCWLPAREKGGNIGLIGNGFGLTLSSWDLMLEQKGEILGAFVIEEKSSSKSLIKQLGIAIEKMLTVPDIKVIFINIVTKHQTSELVAQALLDYCQPLVVTSGSEERLPRPTGSNSTRQRPITPSPMENPIEWVIRLPVDDLQPIEESFSGLSVNCFKEMMEAVKKTVALSSQASKRKS

>Oscillatoriales cyanobacterium (ZP_11390494.1)

MDLLEYQAKELFRDVGIPVLPSQRIDFPKDLKGLTIPYPVVLKSQVYIGGRGRVGGVKFVENTIDAIAAAQTIFNLPIMGEYPKMLLAEAKYNADQEFYLAITLNRSVCRPVLLGSTQGGIDVQSAIDQMQHVIVEQEFSPYYARRLTIKMGLQGALIAAVSSVIEKMYQLFVKHDLDLVEINPLGVSASGEVMALDGKISVNDDALGRHPLLVSLISKLGGNLPQEDDIAPVVMDADGQIGIVCNGAGLTMATMDLVGQAGGKTASFLNIGGENRWDSSPALLKSRLEKGLTLISQHKSVRVILINLISNLVPCDEVAEVIVSYLERRTSKPRLADMHADVMFTQPRRRLPLVVRLVGSQSDRARTQLEATHLSLVENLDEAVTEAVSLAKSGRMES

>Nostoc punctiforme (YP_001868458.1)

MDLLEYQVKEWFGKIGIPVLPSQRIDHPTDLKRLKIRFPIVLKSQVHGAERAKAGGVRFAETTIDAIAAAQNIFSLPIWGELPEVVLAESQYDANQEFYLAVVLDTAVCRPVLLGCKEADIDWESAGEKMHHVVVEQEFSPFYARRLALKMGLQGTLMQSVSSVLEKMYHLFVQKDLDLVEINPLAVSANGQVMALNGKVRINERAIKRHPDLAEMAAKIISRHTSTEINGILGDWDGVKMHGKIGILGNGTGSVMATLDLVANAGGNPGVCLNLRHAFLTDTTPTTFRDRLETGLKILEADKGIQVILINFLGSIPQTEEVVKVIARVVQQDNSELESQVVRSNGSKSRQVQNFTPLVIRLAGSEFNAARKYLATLKTHSDALLVVENLDEAVAAAVRLAKPTANKK

________________________________________________________________________________

SUCCINATE DEHYDROGENASE

FLAVOPROTEIN

>Arabidopsis thaliana (NM_126074.2)

MWRCVSRGFRAPASKTSSLFDGVSGSRFSRFFSTGSTDTRSSYTIVDHTYDAVVVGAGGAGLRAAIGLSEHGFNTACITKLFPTRSHTVAAQGGINAALGNMSEDDWRWHMYDTVKGSDWLGDQDAIQYMCREAPKAVIELENYGLPFSRTEEGKIYQRAFGGQSLDFGKGGQAYRCACAADRTGHALLHTLYGQAMKHNTQFFVEYFALDLLMASDGSCQGVIALNMEDGTLHRFRSSQTILATGGYGRAYFSATSAHTCTGDGNAMVARAGLPLQDLEFVQFHPTGIYGAGCLITEGSRGEGGILRNSEGERFMERYAPTAKDLASRDVVSRSMTMEIREGRGVGPHKDHIYLHLNHLPPEVLKERLPGISETAAIFAGVDVTKEPIPVLPTVHYNMGGIPTNYHGEVVTIKGDDPDAVIPGLMAAGEAACASVHGANRLGANSLLDIVVFGRACANRVAEISKPGEKQKPLEKDAGEKTIAWLDRLRNSNGSLPTSTIRLNMQRIMQNNAAVFRTQETLEEGCQLIDKAWESFGDVQVKDRSMIWNSDLIETLELENLLINASITMHSAEARKESRGAHAREDFTKREDGEWMKHTLGYWEDEKVRLDYRPVHMDTLDDEIDTFPPKARVY

>Fragaria vesca (XP_004287904.1)

MWRCVSRRLGVSSPSRSVAANDSLRSHFSRLFSSDSATGRSSYTVVDHTYDAVVVGAGGAGLRAAIGLSEHGFNTACITKLFPTRSHTVAAQGGINAALGNMTEDDWRWHMYDTVKGSDWLGDQDAIQYMCREAPKAVIELENYGLPFSRTEDGKIYQRAFGGQSLDFGKGGQAYRCACAADRTGHALLHTLYGQAMRHNTQFFVEYFALDLLMNSDGSCQGVIALNMEDGTLHRFQASSTILATGGYGRAYFSATSAHTCTGDGNAMVARAGLPLQDLEFVQFHPTGIYGAGCLITEGSRGEGGILRNSEGERFMERYAPTAKDLASRDVVSRSMTMEIREGRGVGPMKDHIYLHLNHLPPDVLKERLPGISETAAIFAGVDVTKEPIPVLPTVHYNMGGIPTNYHGEVVTIKGDNPDAVIPGLMAAGEAACASVHGANRLGANSLLDIVVFGRACANRVAEISKPGEKQKPLEKDAGEKTIAWLDKLRNSNGSLPTSKIRLNMQRIMQNNAAVFRTHETLVEGAELIDKAWESFNDVQVKDRTLIWNSDLIETIELENLLINACITMHSAEARKESRGAHAREDFTTRDDEKWMKHSLGYWENEKVRLDYRPVHMNTLDDEIDTFPPKARVY

>Solanum lycopersicum (XP_004232400.1)

MWRCVSRGLRASSNRSISGGGHFRRLFSSQTNVGGSSYTVVDHTYDAVVVGAGGAGLRAAIGLSEHGFNTACITKLFPTRSHTVAAQGGINAALGNMTEDDWRWHMYDTVKGSDWLGDQDAIQYMCREAPKAVIELENYGLPFSRTEDGKIYQRAFGGQSLDFGKGGQAYRCACAADRTGHALLHTLYGQAMKHNTQFFVEYFALDLLMNSDGSCQGVIALNMEDGTLHRFRAANTILATGGYGRAYFSATSAHTCTGDGNAMVARAGLPLQDLEFVQFHPTGIYGAGCLITEGSRGEGGILRNSEGERFMERYAPTAKDLASRDVVSRSMTMEIREGRGVGPLKDHIYLHLNHLPPEVLKERLPGISETAAIFAGVDVTKEPIPVLPTVHYNMGGIPTNYHGQVVTKNGDDPDAVIPGLMAAGEAACASVHGANRLGANSLLDIVVFGRACANRVAEIQKPGAEQKPLEKDAGERTIAWLDKIRNSNGSLPTSKIRLDMQRVMQNNAAVFRTQETLEEGCQLIDKTWESFHDVQLKDRGLIWNTDLIETIELENLLINACITMHSAEARKESRGAHAREDFTKRDDEKWMKHTIGYWEDEKVRLEYRPVHMNTLDDEVESFPPKARVY

>Cucumis sativus (XP_004149154.1)

MWRCVSRGLRASSSFSRKSSPNDHLRSQFSRFFSADSTAGRSSYTVVDHTYDAVVVGAGGAGLRAAIGLSEHGFNTACITKLFPTRSHTVAAQGGINAALGNMTEDDWRWHMYDTVKGSDWLGDQDAIQYMCREAPKAVIELENYGLPFSRTEDGKIYQRAFGGQSLDFGKGGQAYRCACAADRTGHALLHTLYGQAMKHNTQFFVEYFALDLIMNSDGSCQGVIALNMEDGTLHRFRAASTILATGGYGRAYFSATSAHTCTGDGNAMVARAGLPLEDLEFVQFHPTGIYGAGCLITEGSRGEGGILRNSEGERFMERYAPTAKDLASRDVVSRSMTMEIREGRGVGPLKDHIYLHLNHLPPDVLKERLPGISETAAIFADVDVTKEPIPVLPTVHYNMGGIPTNHHGEVVTIKGNDPDAIIPGLMAAGEAACASVHGANRLGANSLLDIVVFGRACANRVAEIGRPGKFQKPLEKDAGEKTIAWLDKLRNSNGSLATSKIRLNMQRVMQNNAAVFRTQETLEEGCKLIDKAWESFRDVRVKDRNLIWNTDLIETIELENLLINACITMHSAEARKESRGAHAREDFTKRDDENWMKHTLGFWENEKVRLDYRPVHMNTLDDEIETFPPKARVY

>Glycine max (XP_003537582.1)

MWRCIARGLRGPASTRSTSNHSLGSQLSRFFSSGANSSYTVVDHTYDAVVVGAGGAGLRAAIGLSEHGFNTACITKLFPTRSHTVAAQGGINAALGNMTEDDWRWHMYDTVKGSDWLGDQDAIQYMCREAPKAVIELENYGLPFSRTEDGKIYQRAFGGQSLNYGKGGQAYRCACAADRTGHALLHTLYGQAMRHNTQFFVEYFALDLVMNSDGTCQGVIALNMEDGTLHRFKAASTILATGGYGRAYFSATSAHTCTGDGNAMVARAGIPLEDLEFVQFHPTGIYGAGCLITEGSRGEGGILRNSEGERFMERYAPTAKDLASRDVVSRAMTMEIREGRGVGPLKDHIYLHLNHLPPDVLKERLPGISETAAIFAGVDVTKEPIPVLPTVHYNMGGIPTNHYGEVVTIKGDNPDAVVPGLMAAGETACASVHGANRLGANSLLDIVVFGRACANRVAEIRRPGEKQKPLEKDAGQRTIAWLDKLRNSNGSLPTSQIRLNMQRVMQTNAAVFRTQETLEEGCQLIDKTWESFHDVQVKDRSLIWNSDLIETIELENLLINACITMYSAEARKESRGAHAREDFKVRDDGSWMKHTVGFWENEKVRLDYRPVHLNVLDDEVESFPPKARVY

>Vitis vinifera (XP_003631879.1)

MWRCVSRGLRFRSSDSKRSLTSDRSSYTSSYTIVDHTYDAVVVGAGGAGLRAAIGLSEHGFNTACITKLFPTRSHTVAAQGGINAALGNMTEDDWRWHMYDTVKGSDWLGDQDAIQYMCREAPKAVIELENYGLPFSRTEDGRIYQRAFGGQSLNFGKGGQAYRCACAADRTGHALLHTLYGQAMKHNTQFFVEYFALDLLMDNDGACQGVIALNMEDGTLHRFQAASTILATGGYGRTYFSATSAHTCTGDGNAMVARAGLPLQDLEFVQFHPTGIYGAGCLITEGSRGEGGILRNSEGERFMERYAPTAKDLASRDVVSRSMTMEIREGRGVGPLKDHIYLHLNHLPPEVLKERLPGISETAAIFAGVDVTKEPIPVLPTVHYNMGGIPTNYHGEVLTIKGNDPDAVVPGLMAAGEAACASVHGANRLGANSLLDIVVFGRACANRVAEIHKPGEKQKPLEKDSGEKTIAWLDKLRNSNGSIPTSKIRLNMQRVMQNNAAVFRTQETLEEGCKLIDKAWESFHDVQLKDRSLIWNSDLSETIELENLLINACITMHSAEARKESRGAHAREDFTKRDDENWIRHTLGYWENEKVRLDYRPVHMNPLDDEIESIPPKARVY

>Thellungiella halophila (BAJ33900.1)

MWRCVSRSLRAPYSRTSLSGSRISRLFSTGSTDDYKIVDHTYDAVVVGAGGAGLRAAIGLSEHGFNTACITKLFPTRSHTVAAQGGINAALGNMSEDDWRWHMYDTVKGSDWLGDQDAIQYMCREAPKAVIELENYGLPFSRTEEGKIYQRAFGGQSLDFGKGGQAYRCACAADRTGHALLHTLYGQAMKHNTQFFVEYFALDLLMASDGSCQGVIALNMEDGTLHRFRSAQTILATGGYGRAYFSATSAHTCTGDGNAMVARAGLPLQDLEFVQFHPTGIYGAGCLITEGSRGEGGILRNSEGERFMERYAPTAKDLASRDVVSRSMTMEIREGRGVGPHKDHIYLHLNHLPPEVLKERLPGISETAAIFAGVDVTKEPIPVLPTVHYNMGGIPTNYHGEVVTIKGDDPDAVVPGLMAAGEAACASVHGANRLGANSLLDIVVFGRACANRVAEINKPGEKQRPLEEDAGRKTIEWLNKLRNSRGSLPTSSIRLNMQRIMQNNAAVFRTQETLEEGCQLIDKAWESFEDVQVKDRSLIWNSDLIETIELENLLINAAITMHSAEARKESRGAHAREDFTKREDGEWMKHTLGYWEDEKVRLDYRPVHMDTLDDEIETFPPKARVY

>Zea mays (ACG43057.1)

MWRSCVSRGLSRAKASASRLLSTASSSYTVVDHTYDAVVVGAGGAGLRAAIGLSEHGFNTACITKLFPTRSHTVAAQGGINAALGNMSEDDWRWHMYDTVKGSDWLGDQDSIQYMCREAPKAVIELENYGLPFSRTEEGKIYQRAFGGQSLDFGKGGQAYRCACAADRTGHAMLHTLYGQAMKHNTQFFVEYFALDLLMDNDGKCQGVIALNMEDGTLHRFRASNTILATGGYGRAYFSATSAHTCTGDGNAMVARAGLPLQDLEFVQFHPTGIYGAGCLITEGSRGEGGILRNSEGERFMERYAPTAKDLASRDVVSRSMTMEIREGRGVGPLKDHIYLHLNHLPPEVLKERLPGISETAAIFAGVDVTKEPIPVLPTVHYNMGGIPTNYHGEVVDIKGDNPDAVIPGLMAAGEAACASVHGANRLGANSLLDIVVFGRACANRVADISKPGEKQKPLEKDAGEKTIAWLDKLRNANGSLPTSKIRLNMQRVMQNNAAVFRTQETLEEGCELISKTWESFHDVKLSDRSLIWNSDLIETLELENLLINACITMYSAEARKESRGAHAREDFTTRDDEKWMKHSLGYWENEKVRLAYRPVHMNTLDDEVESFPPKARVY

>Brachypodium distachyon (XP_003558573.1)

MWRSCVSRGLREAKAAARRFSSSSSYTVVDHTYDAVVVGAGGAGLRAAIGLSEHGFNTACITKLFPTRSHTVAAQGGINAALGNMTEDDWRWHMYDTVKGSDWLGDQDSIQYMCREAPKAVIELENYGLPFSRTEDGKIYQRAFGGQSLDFGKGGQAYRCACAADRTGHAMLHTLYGQAMKHNTQFFVEYFALDLIMDNEGTCQGVIALNMEDGTLHRFRSTNTIIATGGYGRAYFSATSAHTCTGDGNAMVARAGLPLQDLEFVQFHPTGIYGAGCLITEGSRGEGGILRNSEGERFMERYAPTAKDLASRDVVSRSMTMEIREGRGVGPLKDHIYLHLNHLPPEVLKERLPGISETAAIFAGVDVTKEPIPVLPTVHYNMGGIPTNYHGEVVHIKGDNPDSVIPGLLAAGEAACASVHGANRLGANSLLDIVVFGRACANRVAEISKPGEKQKPLEKDAGQKTIAWLDKLRNSNGSLPTSKIRLNMQRVMQNNAAVFRTQETLAEGCELITKAQESFHDVKISDRSLIWNSDLIETIELENLLINACITMYSAEARKESRGAHAREDFTTRDDEKWMKHSLGYWEDEKVRLEYRPVHMNTLDDEIETFPPKARVY

>Oryza sativa (NP_001058845.1)

MWRGCVSRGLRSLSKGKGSSSSAPVSAAARLFSTASSSYTVVDHSYDAVVVGAGGAGLRAAIGLSEHGFNTACITKLFPTRSHTVAAQGGINAALGNMTEDDWRWHMYDTVKGSDWLGDQDSIQYMCREAPKAVIELENYGLPFSRTEDGKIYQRAFGGQSLDFGKGGQAYRCACAADRTGHAMLHTLYGQAMKHNTQFFVEYFALDLIMDSEGTCQGVIALNMEDGTLHRFRATNTILATGGYGRAYFSATSAHTCTGDGNAMVARAGLPLQDLEFVQFHPTGIYGAGCLITEGSRGEGGILRNSEGERFMERYAPTAKDLASRDVVSRSMTMEIREGRGVGPLKDHIYLHLNHLPPEVLKERLPGISETAAIFAGVDVTKEPIPVLPTVHYNMGGIPTNYHGEVVTMKGDNPDSVVPGLMAAGEAACASVHGANRLGANSLLDIVVFGRACANRVAETAKPGEKQKPLQKSAGEKTIAWLDKLRNANGSLPTSKIRLNMQRVMQNNAAVFRTQETLEEGCKLITKAWESYHDVKISDRSLIWNSDLIETIELENLLINACITMHSAEARKESRGAHAREDFTKRDDEQWMKHSLGYWENEKVRLAYRPVHMNTLDSEVESFPPKARVY

>Hordeum vulgare (BAK02783.1)

MWRSCVSRGLREAKAAAAAASRRFSTTSSYTVVDHTYDAVVVGAGGAGLRAAIGLSEHGFNTACITKLFPTRSHTVAAQGGINAALGNMSEDDWRWHMYDTVKGSDWLGDQDAIQYMCREAPKAVIELENYGLPFSRTEDGKIYQRAFGGQSLDFGKGGQAYRCACAADRTGHAMLHTLYGQAMKHNTQFFVEYFALDLIMDKEGTCQGVIALNMEDGTLHRFRSTNTILATGGYGRAYFSATSAHTCTGDGNAMVARAGLPLQDLEFVQFHPTGIYGAGCLITEGSRGEGGILRNSEGERFMERYAPTAKDLASRDVVSRSMTMEIREGRGVGPLKDHLYLHLNHLPPEVLKERLPGISETAAIFAGVDVTKEPIPVLPTVHYNMGGIPTNYHGQVVDIKGDNPDTIIPGLMAAGEAACASVHGANRLGANSLLDIVVFGRACANRVAEISKPGETQKPLEKDAGEKTIAWLDKLRNANGSLPTSNIRLNMQRIMQNNAAVFRTQETLTEGCKLISEAQKSFNDVKLSDRSLIWNSDLIETIELENLLINACITMHSAEARQESRGAHAREDFKTRDDDKWMKHSLGYWEDEKVRLEYRPVHMNTLDDEVETFPPKARVY

>Chlamydomonas reinhardtii (XP_001689842.1)

MPVLAKLLGGASKASSLQQLAGLGQRAALAATQQAGFASQSYPVIDHQYDAIVVGAGGAGLRAAVGLSELGFNTACITKLFPTRSHTVAAQGGINAALGNMTEDDWRWHAYDTIKGSDWLGDQDAIHYMCREAPKAVIELENYGLPFSRTEDGKIYQRAFGGQSLDFGKGGQAYRCACAADRTGHAMLHTLYGQAMKHDIQFYVEYFALDLIMDSDGVCRGVMALCMEDGTLHRFQAHQTVLATGGYGRAYFSATSAHTCTGDGNAMAARAGIPLQDLEFVQFHPTGIYGAGCLITEGSRGEGGILRNSEGERFMERYAPTAKDLASRDVVSRSMTMEIREGRGCGPEKDHIYLHLNHLPPELLAERLPGISETAAIFAGVDVTKEPIPVLPTVHYNMGGVPTNYMGEVLAPTPDNPDKVVPGLFAAGEAACASVHGANRLGANSLLDIVVFGRACANRIGQIMKPNTPHKPLPATAGEGAVARLDKLRNAKGNLRTAEIRRNMQKVMQNNAAVFRTQESLEEGCKLIDETMASFQDVKTTDRGLVWNTDLIETLELENLLINAAVTMHSAEKRKESRGAHAREDFTQRDDANWMKHTLGFLPSTNDKVNISYRPVHMKPLSEEMPFIPPKPRVY

>Ostreococcus tauri (XP_003082803.1)

MFASTSRRALRRGCVGPVAALATGASAASTAAVSTASMASASSMASASARASVDARGGRSWTNGARGGGRSRRSRDGFARAYSGTSASSAYEIIDHEYDALVVGAGGAGLRAAIGLGEHGFKTACVTKLFPTRSHTVAAQGGINAALGNMTEDDWRWHAYDTVKGADWLGDQDAIQYMCREAPKAVIELEKYGMPFSRTDDGRIYQRAFGGQSLDFGKGGQAYRCAAAADRTGHAMLHTLYGAALKHDVQFFVEYFALDLIMDKGECVGVMALCLEDGTLHRFRSHQTILATGGYGRAYFSATSAHTCTGDGNAMVARAGLPLQDQEFVQFHPTGIYGAGCLITEGSRGEGGILRNSEGERFMERYAPSAKDLASRDVVSRAMTMEIREGRGVGKEKDHIYLHLNHLPPELLAERLPGISETAAIFAGVDVTKEPIPVIPTVHYNMGGIPTNYKGEVVAPKNGDMDAIVPGLMAAGEAACASVHGANRLGANSLLDIVVFGRACANTVSEKLKPGTPHRQIAADAGSNAVERLDKIRNSKGSAPTAMLRRTMQKVMQDDAAVFRTQESLANGCKRIDDVAAQLDNLKLTDRSLVWNTDLVEALELHNLMPNAQTTMHSAEQRKESRGAHAREDFPDRLDDTWMKHTLAYVENGKVKIDYRPNHHYTLDDEMEVIPPKARVY

>Volvox carteri (XP_002949632.1)

MSVLAKLLTGTAAKAQSTQLGLLGQRSILATAQQTALPELRGYASQTYPVIDHQYDAVVVGAGGAGLRAAVGLSELGFKTACVTKLFPTRSHTVAAQGGINAALGNMTEDDWRWHAYDTVKGSDWLGDQDAIHYMCREAPKAVIELENYGLPFSRTEDGKIYQRAFGGQSLDFGKGGQAYRCACAADRTGHAMLHTLYGMAMKHNIQFFVEYFALDLMMDSDGACRGIMALCMEDGTIHRFQAHQTVLATGGYGRAYFSATSAHTCTGDGGGMVARAGLPLQDLEFVQFHPTGIYGAGCLITEGCRGEGGILRNSEGERFMERYAPTAKDLASRDVVSRSMTIEIREGRGCGPEKDHIYLHLNHLPPELLAERLPGISETAAIFAGVDVTKEPIPVLPTVHYNMGGIPTNYMGEVLAPTKENPDKVVPGLFAAGEAACASVHGANRLGANSLLDIVVFGRACANRVGEIMKPNTPHKPLPASAGEHAIARLDKLRNAKGNLRTAEIRRNMQKVMQNNAAVFRTQETLAEGCKLIDECAASFSDVKVTDRGLVWNTDLVETLELENLLLNAAITMHGAEQRKESRGAHAREDFTERDDAKWLKHTLGYMPSVENKVSISYRPVHMQPLSEEMPYIPPKARVY

>Escherichia coli (AAC73817.1)

MKLPVREFDAVVIGAGGAGMRAALQISQSGQTCALLSKVFPTRSHTVSAQGGITVALGNTHEDNWEWHMYDTVKGSDYIGDQDAIEYMCKTGPEAILELEHMGLPFSRLDDGRIYQRPFGGQSKNFGGEQAARTAAAADRTGHALLHTLYQQNLKNHTTIFSEWYALDLVKNQDGAVVGCTALCIETGEVVYFKARATVLATGGAGRIYQSTTNAHINTGDGVGMAIRAGVPVQDMEMWQFHPTGIAGAGVLVTEGCRGEGGYLLNKHGERFMERYAPNAKDLAGRDVVARSIMIEIREGRGCDGPWGPHAKLKLDHLGKEVLESRLPGILELSRTFAHVDPVKEPIPVIPTCHYMMGGIPTKVTGQALTVNEKGEDVVVPGLFAVGEIACVSVHGANRLGGNSLLDLVVFGRAAGLHLQESIAEQGALRDASESDVEASLDRLNRWNNNRNGEDPVAIRKALQECMQHNFSVFREGDAMAKGLEQLKVIRERLKNARLDDTSSEFNTQRVECLELDNLMETAYATAVSANFRTESRGAHSRFDFPDRDDENWLCHSLYLPESESMTRRSVNMEPKLRPAFPPKIRTY

>Sphingomonas (WP_009822262.1)

MTEAYKIIDHTYDTVVVGAGGSGLRATMGSAEAGLKTACITKLFPTRSHTVAAQGGIAASLGNNSPDHWTWHMYDTVKGSDWLGDQDAIEYMVREAPAAVIELEHAGVPFSRNENGTIYQRPFGGHMQNMGEGPPVQRTCAAADRTGHAMLHALYQQSLKYDADFYIEYFAIDLIMENGECRGVIAICMEDGSIHRFRAHAVVLATGGYGRAYFSATSAHSCTGDGGGMVLRAGLPLQDLEFVQFHPTGIYGAGVLITEGARGEGGYLTNSEGERFMERYAPSAKDLASRDVVSRSMAMEMREGRGVGPNKDHIFLHLDHIDPKVLAERLPGITESGKIFAGVDLTRQPLPVTPTVHYNMGGIPCNYHGQVVTKIGDDPEVVVPGLYAVGEAACVSVHGANRLGSNSLIDLVVFGRATGLHLKETLKPNGSHKPLPKDAADLALSRLDTYRNAKGGSPTAAIRLEMQHTMQKHAAVFRDSELLAEGVQKMAQVNKRMEDVSVADRSLIWNTDLIETLELDNLMSSAVCTMVSAENRKESRGAHAHEDFPNRDDENWMKHTISWFDGWGGQGGKVSLDYRPVHDYTLTDEADYIKPKARVY

>Maricaulis maris (YP_758065.1)

MAEYKWIDHTYDVVVVGAGGSGLRAALGASQAGLKTACITKVFPTRSHTVAAQGGISASLGNMGEDDWRWHMYDTVKGSDWLGDQDAIEYLCREAPAAVYELEHWGVPFSRTEDGKIYQRAFGGMTKNYGEGPVQRTCAAADRTGHAILHTLYGQSVRNETEFFIEYFALDLIMDDDGVCRGITAWKLDDGTLHRFRAQTVVLATGGYGRAYFSATSAHTCTGDGNAMVLRAGLPLQDMEFVQFHPTGIYGAGCLITEGARGEGGYLTNSNGERFMERYAPSAKDLASRDVVSRSMTMEIREGRGVGANGDHIHLHLDHLDPDILGERLPGISESARVFAGVDVTKEPIPVLPTVHYNMGGIPTNYHGEVLTKVGGDADTVVPGLMAVGEAACVSVHGANRLGSNSLIDLVVFGRAAGLRLGETTKAGASQPEIKSTDGQLSLDRLDKYRNAAGGTPTAKLRLEMQRSMQNNCAVFRTGDVLAEGVEAIKQVYTGMADIGVTDRTMVWNTDLMETLEMDNLLSQAAVTVNGAANREESRGAHAREDFSDRDDENWMKHTLAWCDEAGNVKIDYRPVHTYTMSNDIEYIKPKARVY

>Candidatus Pelagibacter (WP_008544378.1)

MSTYKIIDHEYDVVVLGAGGSGLRAAVGLSEAGLKTACISKVFPTRSHTSAAQGGISAALGNMGEDDWRWHMYDTVKGADWLGDQDSIEYLCKEAPQAVIELEKYGVPFSRTEEGKIYQRPFGGMTKNYGNGIVQRTCAAADRTGHAILHTLYGQALKHNTEFFIEYFALDLLMKDGECKGLIAWNLNDGTIHRFRAHTVIIATGGYGKVYYSATSAHTCTGDGNAMVLRAGLPLQDMEFVQFHPTGIYGHGTLITEGARGEGGYLTNSKGERFMERYAPSAKDLASRDVVSRSMSIEINEGRGVGKDQDHVHLNLSHLDKEIIESRLPGITDAARLFANVDVTKEPIPVVPTVHYNMGGIPTNYKGEVMTVNGSEKTVPGLMAIGEAACVSVHGANRLGSNSLIDLVVFGRAAAKRAAELIKPGTPHEEIGETETQKCLDRFDKLRNANGENSTADLRLAMQKTMQSKCAVFRTEKNLKEGVDEIRKTYDGMDSISVKDRSLVFNTDLVETLEFDNLIRQAVATVDSAYHRKESRGAHARDDYPKRDDEKFMQHTLAWCDGKNTKISYREVHKSTLTNEVQYFPPQERVY

>Saccharomyces cerevisiae (EGA85885.1)

MLSLXKSALSKLTLLRNTRTFTSSALVRQTQGSVNGSASRSADGKYHIIDHEYDCVVIGAGGAGLRAAFGLAEAGYKTACISKLFPTRSHTVAAQGGINAALGNMHKDNWKWHMYDTVKGSDWLGDQDSIHYMTREAPKSIIELEHYGVPFSRTENGKIYQRAFGGQTKEYGKGAQAYRTCAVADRTGHALLHTLYGQALRHDTHFFIEYFALDLLTHNGEVVGVIAYNQEDGTIHRFRAHKTIIATGGYGRAYFSCTSAHTCTGDGNAMVSRAGFPLQDLEFVQFHPSGIYGSGCLITEGARGEGGFLVNSEGERFMERYAPTAKDLACRDVVSRAITMEIREGRGVGKKKDHMYLQLSHLPPEVLKERLPGISETAAIFAGVDVTKEPIPIIPTVHYNMGGIPTKWNGEALTIDEETGEDKVIPGLMACGEAACVSVHGANRLGANSLLDLVVFGRAVAHTVADTLQPGLPHKPLPSDLGKESIANLDKLRNANGSRSTAEIRMNMKQTMQKDVSVFRTQSSLDEGVRNITAVEKTFDDVKTTDRSMIWNSDLVETLELQNLLTCASQTAVSAANRKESRGAHAREDYPNRDDEHWMKHTLSWQKDVAAPVTLKYRRVIDHTLDEKECPSVPPTVRAY

+3

>Caenorhabditis elegans (NP_509446.1)

MLRAASNGLRNTVAARSVSLSAANHSDAKRSDIAQYKVVDHAYDAVVVGAGGAGLRAAMGLAEGGLKTAVITKLFPTRSHTVAAQGGINAALGNMNPDNWRWHFYDTVKGSDWLGDQDAIHYMTREAERAVIELENYGMPFSRTTDGKIYQRAFGGQSNDFGRGGQAHRTCCVADRTGHSLLHTLYGASLQYNCNYFVEYFALDLIMENGVCVGVIAMDLEDGTIHRFRSKNTVLATGGYGRAFFSCTSAHTCTGDGTALTARAGINNSDMEFVQFHPTGIYGAGCLITEGSRGEGGYLVNSAGERFMERYAPNAKDLASRDVVSRSMTVEIMEGRGVGPDKDHIYLQLHHLPAEQLQQRLPGISETAMIFAGVDVTKEPIPVIPTVHYNMGGVPTNYKGQVLNYTPKKGDEVVPGLYAAGECGAHSVHGANRLGANSLLDLVIFGRACAIDILKNTSAGVGVPELPKNAGEASVANIDKLRHNKGDISTAELRLTMQKSMQNHAAVFRRGDILKEGVKVLSKLYKDQAHLNVADKGLVWNSDLIETLELQNLLINATQTIVAAENREESRGAHARDDFPDRLDELDYSKPLEGQTKKELKDHWRKHSIIRSNIETGEVSLDYRPVIDTTLDKSETDWVPPKVRSY

>Mus musculus (BAE26754.1)

MAGVGAVSRLLRGRRLALTGAWPGTLQKQTCGFHFSVGENKKASAKVSDAISTQYPVVDHEFDAVVVGVGGAGLRAAFGLSEAGFNTACLTKLFPTRSHTVAAQGGINAALGNMEEDNWRWHFYDTVKGSDWLGDQDAIHYMTEQAPASVVELENYGMPFSRTEDGKIYQRAFGGQSLKFGKGGQAHRCCCVADRTGHSLLHTLYGRSLRYDTSYFVEYFALDLLMENGECRGVIALCIEDGSIHQIRAKNTVIATGGYGRTYFSCTSAHTSTGDGTAMVTRAGLPCQDLEFVQFHPTGIYGAGCLITEGCRGEGGILINSQGERFMERYAPVAKDLASRDVVSRSMTLEIREGRGCGPEKDHVYLQLHHLPPEQLATRLPGISETAMIFAGVDVTKEPIPVLPTVHYNMGGIPTNYKGQVLKHVNGRDQIVPGLYACGEAACASVHGANRLGANSLLDLVVFGRACALSIAESCRPGDKVPSIKANAGEESVMNLDKLRFADGSIRTSELRLNMQKSMQNHAAVFRVGSVLQEGCEKISQLYGDLKHLKTFDRGMVWNTDLVETLELQNLMLCALQTIYGAEARKESRGAHAREDYKVRVDEYDYSKPIQGQQKKPFGEHWRKHTLSYVDIKTGKVTLEYRPVIDKTLNEADCATVPPAIRSY

>Canis lupus (XP_535807.3)

MSGVRAVSRLLGARRLALTRAWPAAWQTGTRSFHFTIDGNKRSSAKVSDSISTQYPVVDHEFDAVVVGAGGAGLRAAFGLSEAGFNTACVTKLFPTRSHTVAAQGGINAALGNMEEDNWRWHFYDTVKGSDWLGDQDAIHYMTEQAPASVVELENYGMPFSRTEDGRIYQRAFGGQSLKFGKGGQAHRCCCVADRTGHSLLHTLYGRSLRYDTSYFVEYFALDLLMENGECRGVIALCIEDGSIHRIRAKNTVVATGGYGRTYFSCTSAHTSTGDGTAMVTRAGLPCQDLEFVQFHPTGIYGAGCLITEGCRGEGGILINSQGERFMERYAPVAKDLASRDVVSRSMTLEIREGRGCGPEKDHVYLQLHHLPPEQLAIRLPGISETAMIFAGVDVTKEPIPVLPTVHYNMGGIPTNYKGQVLRHVNGQDQIVPGLYACGEAACASVHGANRLGANSLLDLVVFGRACALSIAESCRPGDKIPPIKPNAGEESVMNLDKLRFANGSVRTSELRLNMQKSMQSHAAVFRVGSVLQEGCEKISQLYGDLKHLKTFDRGMVWNTDLVETLELQNLMLCALQTIYGAEARKESRGAHAREDYKVRIDEYDYSKPIQGQQKKPFEEHWRKHTLSYVDIKTGKVSLEYRPVIDRTLNEADCATVPPAIRSY

>Homo sapiens (AAA20683.1)

MSGVRGLSRLLSARRLALAKAWPTVLQTGTRGFHFTVDGNKRASAKVSDSISAQYPVVDHEFDAVVVGAGGAGLRAAFGLSEAGFNTACVTKLFPTRSHTVAAQGGINAALGNMEEDNWRWHFYDTVKGSDWLGDQDAIHYMTEQAPAAVVELENYGMPFSRTEDGKIYQRAFGGQSLKFGKGGQAHRCCCVADRTGHSLLHTLYGRSLRYDTSYFVEYFALDLLMENGECRGVIALCIEDGSIHRIRAKNTVVATGGYGRTYFSCTSAHTSTGDGTAMITRAGLPCQDLEFVQFHPTGIYGAGCLITEGCRGEGGILINSQGERFMERYAPVAKDLASRDVVSRSMTLEIREGRGCGPEKDHVYLQLHHLPPEQLATRLPGISETAMIFAGVDVTKEPIPVLPTVHYNMGGIPTNYKGQVLRHVNGQDQIVPGLYACGEAACASVHGANRLGANSLLDLVVFGRACALSIEESCRPGDKVPPIKPNAGEESVMNLDKLRFADGSIRTSELRLSMQKSMQNHAAVFRVGSVLQEGCGKISKLYGDLKHLKTFDRGMVWNTDLVETLELQNLMLCALQTIYGAEARKESRGAHAREDYKVRIDEYDYSKPIQGQQKKPFEEHWRKHTLSFVDVGTGKVTLEYRPVIDKTLNEADCATIPPAIRSY

>Gallus gallus (NP_001264327.1)

MAAVVAASRSLAKCWLRPAVRAWPAACQTHARNFHFTVDGKKNASTKVSDSISTQYPVVDHEFDAVVVGAGGAGLRAAFGLSEAGFNTACVTKLFPTRSHTVAAQGGINAALGNMEDDNWRWHFYDTVKGSDWLGDQDAIHYMTEQAPAAVIELENYGMPFSRTEEGKIYQRAFGGQSLQFGKGGQAHRCCCVADRTGHSLLHTLYGRSLRYDTSYFVEYFALDLLMENGECRGVIALCIEDGTIHRFRAKNTVIATGGYGRTYFSCTSAHTSTGDGTAMVTRAGLPCQDLEFVQFHPTGIYGAGCLITEGCRGEGGILINSQGERFMERYAPVAKDLASRDVVSRSMTIEIREGRGCGPEKDHVYLQLHHLPPQQLATRLPGISETAMIFAGVDVTKEPIPVLPTVHYNMGGIPTNYKGQVITHVNGEDKVVPGLYACGEAASASVHGANRLGANSLLDLVVFGRACALTIAETCKPGEPVPSIKPNAGEESVANLDKLRFADGTIRTSEARLNMQKTMQSHAAVFRTGSILQEGCEKLSQIYRDLAHLKTFDRGIVWNTDLVETLELQNLMLCALQTIYGAEARKESRGAHAREDYKLRIDEFDYSKPLQGQQKRPFEEHWRKHTLSYVDVKSGKVTLKYRPVIDRTLNEEDCSSVPPAIRSY

>Bos taurus (AAI05358.1)

MSALRSSQPVPRGPPGVAAVSRLWRARRLALTCTKWPAAWQTGTRSFHFTVDGNKRSSAKVSDAISAQYPVVDHEFDAVVVGAGGAGLRAAFGLSEAGFNTACVTKLFPTRSHTVAAQGGINAALGNMEEDNWRWHFYDTVKGSDWLGDQDAIHYMTEQAPASVVELENYGMPFSRTEDGKIYQRAFGGQSLKFGKGGQAHRCCCVADRTGHSLLHTLYGRSLRYDTSYFVEYFALDLLMESGECRGVIALCIEDGSIHRIRARNTVIATGGYGRTYFSCTSAHTSTGDGTAMVTRAGLPCQDLEFVQFHPTGIYGAGCLITEGCRGEGGILINSQGERFMERYAPVAKDLASRDVVSRSMTLEIREGRGCGPEKDHVYLQLHHLPPAQLAMRLPGISETAMIFAGVDVTKEPIPVLPTVHYNMGGIPTNYKGQVLRHVNGQDQVVPGLYACGEAACASVHGANRLGANSLLDLVVFGRACALSIAESCRPGDKVPSIKPNAGEESVMNLDKLRFANGSIRTSELRLNMQKSMQSHAAVFRVGSVLQEGCEKISSLYGDLRHLKTFDRGMVWNTDLVETLELQNLMLCALQTIYGAEARKESRGAHAREDFKERVDEYDYSKPIQGQQKKPFEQHWRKHTLSYVDIKTGKVTLEYRPVIDRTLNETDCATVPPAIRSY

>Thermosynechococcus elongatus (NP_682167.1)

MLNKRTMQDFGVVIVGGGLAGCRAALEICRLAPETPIALVSKTHPIRSHSVAAQGGIAATLKNVDTEDSWESHAFDTVKGSDFLADQDAVAILAQEAPSVVIDLEHLGVLFSRLPDGRIAQRPFGGHTHPRTCYAADKTGHAILHELYCNLLKYNVTFFSEWYVLRLIVEDQQAKGVVAYHIETGQLDILRAPAILFATGGYGRVFNTTSNDFASTGDGLGMTARAGLPLEDMEFVQFHPTGLYPAGVLISEAVRGEGAYLLNAEGERFMARYAPSRMELAPRDITSRAIAIEIREGRGIHSDGSAGGPFVYLDVRHLGREKIMNRIPFCWEEAHRLAGVDAVVEPIPVRPTVHYSMGGIPVNLNGQVRANATEMVTGFYAAGECACVSVHGANRLGSNSLLECVVYGRRTGAAIAKDLPSLPRPQLDPQFYLQAAAREIQHLFDQGGDLRIAQLRRQVQDCMTQYCGVFRTADFMERGLAELQRLKAAYGRVRLDDRQRYWNTELIEAFELANLLIVAEVILRSALSRQESRGAHFREDYPQRDDVNYLRHTLATYDSDGIRIDYLPVTITLFPPQERNY

>Synechocystis (NP_440839.1)

MLEQDVVIVGGGLAGCRAALEIKRLAPDTKVAIVAKTHPIRSHSVAAQGGIAASLKNVDAEDSWEAHAFDTVKGSDYLADQDAVEILTKEAPEVIIELEHLGVLFSRLPDGKIAQRAFGGHSHNRTCYAADKTGHAILHELVNNLRRNKVEIYDEWYVMKLIYEEGEAKGLVMYEIATGRIEIVRAKAVMVATGGYGRVYNTTSNDYASTGDGLAMAAIAGIPLEDMEFVQFHPTGLYPVGVLISEAVRGEGAYLINSEGRRFMEDYAPSRMELAPRDITSRAITLEIRAGRGVNADGSAGGPYVYLDLRHMGREKIMSRIPFCWEEAHRLVGIDAVEQPMPVRPTVHYCMGGIPVNTDGRVRKNANELTEGFFAAGECACVSVHGGNRLGSNSLLECVVYGRRTGRSIAEYVQGRSLPEIDEAVYKTEAQTRIDQLLNQQGTVRINTLRQAFQDCMTSHCGVFRSESFMAEGLEQVQNLKAQYGQIFLDDKQPQWNTEVIEALELQSIMAVGELILTSAIQRQESRGSHAREDFPSRDDEQFLRHTLASFDGEQIKVEYMPVVINRFEPKERKY

>Synechococcus (YP_001735801.1)

MLQHDVIIVGGGLAGCRAALEIKKKNPNFDVGLVAKTHPIRSHSVAAQGGIAASLKNVDPEDNWEAHAFDTVKGSDYLADQDAVEILTKEAPDVIIELEHLGVLFSRLEDGRIAQRAFGGHSHKRTCYAADKTGHAILHELVNNLRRNGVNIYDEWYVMNLIFEENQAKGIVMYHIETGHLEIVQTKAVMFATGGYGRVFNTTSNDFASTGDGLAMTAAVGLPLEDMEFVQFHPTGLFPVGVLISEAVRGEGAYLRNSDGDRFMEKYAPKQMELAPRDITSRAITLEIRAGRGIHPDGSAGGNFVHLDLTHMGKEKIMERVPFCWEEAHRLVGVDAINEPMPVRPTAHYSMGGIPVNTDGRVRRNGTELTEGFFAAGECACVSVHGANRLGSNSLLECVVYGQRVGGKIADYIGDRPFPEINEQQYLDEAKNRIGQLLNQSGNLRIHNLRQQFQDAMSDHCGVFRTEATMAAGLEQIQHLKDQYNHLFLDDKDIHWNTELIEALELQSILKVGEAILTSAYHRKESRGAHSREDFPTRDDQTYLAHTLSFCNDTGVTIEYMPVVINRFEPKERKY

>Anabaena cylindrica (YP_007157014.1)

MLEHDVIIVGGGLAGCRAALEIARTDPSLNVAVVAKTHPIRSHSVAAQGGMAASLKNVDDEDSWEAHAFDTVKGSDYLADQDAVAILTQEAPNVVIDLEHMGVLFSRLADGRIAQRAFGGHSHNRTCYAADKTGHAILHELVNNLRRYGVQIYEEWYVMRLILEDGQAKGLVMFHLLDGQIEVVRAKAVMFATGGYGRVYNTTSNDYASTGDGLAMTAIAGLPLEDMEFVQFHPTGLYPVGVLISEAVRGEGAYLINAEGDRFMANYAPSRMELAPRDITSRAITYEIRAGRGVNLDGSPGGPFVYLDLRHMGKEKIMSRVPFCWEEAHRLVGVDAVTQPMPVRPTIHYCMGGIPVNTDGQVRSSGDSLVDGFYAAGETSCVSVHGANRLGSNSLLECVVYGKRTGASIARYVQNRKFPNVNEQPYITEAKQQIQSLLEQPGKYRINQIRQEFQDTMTQYCGVFRTEELMQEGLEKLEKLQQQYPQIYLDDKGSCWNTELVEALELQSLMVVGQTILASALNRQESRGAHFREDFPNRDDSQFLQHTMAYYSPAGIDIQYRPVMIDMFEPKERKY

>Microcystis aeruginosa (WP_002767958.1)

MKEHDVVIVGGGLAGCRAALEIKRLNPTIDVAVVAKTHPIRSHSVAAQGGIAAALQNVDPNDNPKAHAYDTVKGSDFLADQDAVDILTQEAPEVIIELEHLGVLFSRLEDGRIAQRAFGGHSHNRACYAADKTGHAMLHELVSNLRRNQVKIYDEWYVLRLILEEGTAKGVVMYQIATGQLEILRAKAIMFGTGGYGRVYNTTSNDFACTGDGLALSAKAGIPLEDMEFVQFHPTGLYPVGVLISEAVRGEGAYLINSEGRRFMEDYAPSRMELAPRDITSRAITLEIRAGRGVNLDGSAGGPFVYLDLRHLGREKIMSRIPFCWEEAHRLVGVDAVEEPMPVRPTVHYCMGGIPVNTDGRVRLSADTLSEGFFSAGECSCVSVHGANRLGSNSLLECVVYGRRTGKSIAKYVEKRAFPVFNPDIYLQDAKEEITALLTKKGTIRIGQLRQQFQDCMTQHCGVFRSEATMREGIKQIGELKRQYEQIYLDDRGDCWNTELIEAWELQSLMVVGEIILTSALNRQESRGAHSREDFPQRDDQNFLQHTLAYYSPSGIDIDYMPVVIQDFTPVERKY

IRON-SULFUR

>Arabidopsis thaliana (NM_113653.3)

MASGLIGRLVGTKPSKLATAARLIPARWTSTGAEAETKASSGGGRGSNLKTFQIYRWNPDNPGKPELQNYQIDLKDCGPMVLDALIKIKNEMDPSLTFRRSCREGICGSCAMNIDGCNGLACLTKIQDEASETTITPLPHMFVIKDLVVDMTNFYNQYKSIEPWLKRKTPASVPAKEILQSKKDRAKLDGMYECILCACCSTSCPSYWWNPESYLGPAALLHANRWISDSRDEYTKERLEAIDDEFKLYRCHTILNCARACPKGLNPGKQITHIKQLQR

>Capsella rubella (EOA24529.1)

MASGLIGRLVGTKPSRLTTAARLMPARWTSTGAEAETKASSGGGGAGKDSKLKTFQIYRWNPDNPGKPELQDYQIDLKDCGPMVLDALIKIKNEMDPSLTFRRSCREGICGSCAMNIDGCNGLACLTKIQDGASETTITPLPHMFVIKDLVVDMTNFYNQYKSIEPWLKRKTPPSVPGKEILQSKKDRAKLDGMYECILCACCSTSCPSYWWNPESYLGPAALLHANRWISDSRDEYTKERLEAIDDEFKLYRCHTILNCARACPKGLNPGKQITHIKQLQR

>Thellungiella halophila (BAJ34353.1)

MASGLIGRLVGTKPSRLTTAARLIPARCTASGSEAEPKASSGGGGGSKLKTFQIYRWNPDNPGKPQLQDYQIDLKDCGPMVLDALIKIKNEMDPSLTFRRSCREGICGSCAMNIDGCNGLACLTKIQDGASETTITPLPHMFVIKDLVVDMTNFYNQYKSIEPWLKRKNPPSEPGKEILQSKKDRAKLDGMYECILCACCSTSCPSYWWNPESYLGPAALLHANRWISDSRDEYTKERLEAIDDEFKLYRCHTILNCARACPKGLNPGKQIAHIKQLQR

>Cucumis melo (ADN34136.1)

MATGILRRVLSRVSASSPSRFVRIRAHASEAEAQQVEQKATASSNLKTFAIYRWNPDSPSKPELQEYKIDLKECGPMVLDALIKIKNEIDPSLTFRRSCREGICGSCAMNIDGCNGLACLTKIPSGDSSTITPLPHMFVIKDLVVDMTNFYNQYKSIEPWLKRKSEPPVPGKEILQSKKDRAKLDGMYECILCACCSTSCPSYWWNPESYLGPAALLHANRWISDSRDEYTKERLEAINDEFKLYRCHTILNCARACPKGLNPGKQIQHIKSLQLLG

>Fragaria vesca (XP_004304690.1)

MATGLLRRAFTGISSSTSPAARRVLLRAFASEPQIDASTPKARETSKLKTFSIYRWSPDSPTKPELKEYEIDLKDCGPMVLDALIKIKNEMDPSLTFRRSCREGICGSCAMNIDGCNGLACLTKIESGSATTITPLPHMYVIKDLVVDMTNFYNQYKSIEPWLKRKNPAEEPGKEILQTKKDRAKLDGMYECILCACCSTSCPSYWWNPEAYLGPAALLHANRWIVDSRDEYTKERLDAINDEYKLYRCHTILNCARACPKGLNPGKQIQHIKQLQPAGNSDSLSV

>Glycine max (XP_003553891.1)

MATGLLKRAAQRLPSSPGFKLALLRAHASEAQAQQVEPKARGTPTLKTFQIYRWSPENPSKPELKDYQINLKECGPMVLDALIKIKNEIDPSLTFRRSCREGICGSCAMNIDGCNGLACLTKIPSEGAATTITPLPHMFVIKDLVVDMTNFYNQYKSIEPWLKRKNPPEPGKEILQSKKEREKLDGMYECILCACCSTSCPSYWWNPESYLGPAALLHANRWISDSRDEYTKERLDAINDEFKLYRCHTILNCARACPKGLNPGKQISHIKSLQPKA

>Solanum lycopersicum (XP_004231896.1)

MATGLIRRAIYRVQSSSPAARLLVARAHASDTKSQQVXNLKSFQIYRWNPDNPGKPELQEYKIDLKECGPMVLDALIKIKNEVDPSPTFRRSCREGICGSCAMNIDGCNGLACLTKISSDSVSTITPLPHMFVIKDLVVDMTNFYNQYKSIEPWLKRKTPAPTPGKEIPQSKSDKAKLDGMYECILCACCSTSCPSYWWNPESYLGPAALLHANRWIMDSRDEHTLERLDAVNDEFKLYRCHTILNCSRACPKGFNPGKXQNIKRLEMAP

>Zea mays (NP_001136531.1)

MAAAALLRRSPAARALLSPALSSRLVASKPHSSSPTQPPPEAKGASSTKTFSIYRWDPDSPSTKPHLRDYQVDLSDCGPMVLDALLKIKNEQDPSLTFRRSCREGICGSCAMNIDGDNGLACLTKISSASSASTVSPLPHMFVIKDLVVDMTNFYNQYKSVEPWLKRKDPPPQLGKEVPQTKADRAKLDGMYECILCACCSTSCPSYWWNPEEYLGPAALLHANRWIQDSRDQFTKERLDAINDEFKLYRCHTIKNCTHACPKGLNPAKQIDTIKKLQLDA

>Brachypodium distachyon (XP_003573312.1)

MAAAALLRRSQTAARALLSPAPLSSRLVASKPHSSSPAPPPPSASTKTFSIYRWDPDSPSTKPHLKDYKVDLSDCGPMVLDALLKIKNEQDPSLTFRRSCREGICGSCAMNIDGDNGLACLTKISSESKGASTISPLPHMFVVKDLVVDMTNFYNQYKSVEPWLKRKDPPSPEGKEIPQTKADRAKLDGMYECILCACCSTSCPSYWWNPEEYLGPAALLHANRWIQDSRDQYTKERLDSINDEFKLYRCHTIKNCTHACPKGLNPAKQIDTIKKLQLGA

>Triticum aestivum (CAD62367.1)

MAAAALLRRSPAARALLSPALSSRLVASKPHSSSPAPPPPSSKPASTKTFSIYRWDPDSPSTKPHLKDYKVDLSDCGPMVLDALLKIKNEQDPSLTFRRSCREGICGTCAMNIDGDNGLACLTKISSEAAGASTISPLPHMFVVKDLVVDMTNFYNQYKSVEPWLKRKDPPAAGGKEIYQSKADRAKLDGMYECILCACCSTSCPSYWWNPEEYLGPAALLHANRWIQDSRDEFTKERLDSINDEFKLYRCHTIKNCTHACPKGLNPAKQIDTIKKLQLGA

>Oryza sativa (NP_001060876.1)

MAAAALLRRSPAARALLSPALSSRLVASKPHSSSPAPPPPPSKAGANTKTFSIYRWDPDSPSTKPHLKDYKVDLSDCGPMVLDVLLKIKNEQDPSLTFRRSCREGICGSCAMNIDGDNGLACLTKISSASSASTISPLPHMFVIKDLVVDMTNFYNQYKSVEPWLKRKDAPPQPGKEIPQTKADRAKLDGMYECILCACCSTSCPSYWWNPEEYLGPAALLHANRWIQDSRDQFTKERLDSINDEFKLYRCHTIKNCTHACPKGLNPAKHIDTIKKLQLEA

>Ostreococcus lucimarinus (XP_001421210.1)

MRASASASASTSATTTTTTTTTTNVQQFRVYRWDPDSGDEPKYKTYDVDTNDCGPMMLDVLFKIKDEQDQTLSFRRSCREGICGSCAMNINGRNGLACLTKVEKTEKGAQTIAPLPHMFVVRDLVVDMANFYAQYKSIEPFLKTKSDAPANGAERLQTKEDRAKLDGLYECILCACCSTSCPSYWWNSDKYLGPAVLLQAYRWIIDSRDEFTEERLANVNDAFKLYRCHTIMNCSKVCPKGLNPALKIAKIKQMVA

>Micromonas (XP_002506801.1)

MFAAALRRGAGAVARDRPATASIFDISRVVTTGASNPAHAAAAATTTAAESAKPPANVQEFQIYRWDPESGGKPRYQTYKVDTNACGPMMLDVLFKIKDEQDNSLAFRRSCREGICGSCAMNINGTNTLACLSKVDKATSGAQKIAPLPHMFVVRDLVVDMANFYAQYKSVEPYLKIKSGDLKKGVEHIQTKEERAKLDGLYECILCACCSTSCPSYWWNQDQYLGPAVLMQAYRWIIDSRDEYTEERLDKVNDAMKLFRCHTIMNCSKVCPKGLNPAKAIAKVKTMVH

>Chlamydomonas reinhardtii (XP_001696290.1)

MLPSLLTNARRGAQAALQPGFLSAFISTTSESLNAAATATASKPAPSRPPLAKPPLYKEFQIYRWNPDSDEKPKYASYQVDINNCGPMMLDVLLKIKDEQDQTLSLRRSCREGICGSCAMNIDGSNTLACLCKVNRDPGHVGKVAPLPHMFVVKDLVVDMANFYAQYKSIKPYLQKKEAAKGQEFYQSKESRAKLDGLYECILCACCSTSCPSYWWNSDKYLGPAVLLAAYRWIIDSRDDMTSERMKEVDDAYKLYRCKTIMNCATVCPKGLNPGKAINKIKQSLAKGSPV

>Escherichia coli (AAC73818.1)

MRLEFSIYRYNPDVDDAPRMQDYTLEADEGRDMMLLDALIQLKEKDPSLSFRRSCREGVCGSDGLNMNGKNGLACITPISALNQPGKKIVIRPLPGLPVIRDLVVDMGQFYAQYEKIKPYLLNNGQNPPAREHLQMPEQREKLDGLYECILCACCSTSCPSFWWNPDKFIGPAGLLAAYRFLIDSRDTETDSRLDGLSDAFSVFRCHSIMNCVSVCPKGLNPTRAIGHIKSMLLQRNA

>Novosphingobium aromaticivorans (YP_497593.1)

MATFSLPKNSKITGKARHHAAATNGKVRKFKVYRYDPDSGENPRYDTFEIDLDQCGPMVLDALIKMKGEQDPTLTFRRSCREGICGSCAMNMNGRNGLACTTAIEDLSGDIRITPLPHMEVIKDLVPDFTHFYAQYASIRPWLQTVSPTPSGKERLQSPEQREKLDGLYECILCACCSTSCPSYWWNSDKFLGPAILLQAYRWLADSRDEMTGERLDELEDPFRLYRCHTIMNCANVCPKGLSPARAIAEIKKMQAERLV

>Erythrobacter (WP_006832237.1)

MATFTLPKNSKITGKSRVHKAEGAARVKKFKVYRYDPDTGENPRYDTFEIDLDECGPMVLDALFKIKNEVDPTLTFRRSCREGICGSCSMNMNGKNGLACTTAIDDLKGEIRITPLPHMEVIKDLVPDFTHFYAQYASIRPWLQTVSTTPSGKERLQSPEQREKLDGLYECILCACCSTSCPSYWWNSDKFLGPAILLQAYRWLADSRDEMTGERLDQLEDPFRLYRCHTIMNCANVCPKGLSPAKAIAETKKMMAERAI

>Novosphingobium (WP_008993907.1)

MATFTLPANSKIRKQGKVHKAEAGATKIKKFTVYRYDPDSGENPRYDTFEIDLDKCGPMVLDAIIKIKNEMDSTLTFRRSCREGICGSCSMNMNGKNGLACTTAIEDLKGDIRITPLPSMDVIKDLVPDFTHFYAQYASIRPWLQTVSTTPSGKERLQSPEQREKLDGLYECILCACCSTSCPSYWWNSDKFLGPAILLQAYRWLADSRDEMTGERLDELEDPFRLYRCHTIMNCANVCPKGLSPARAIAEIKKMQAERQV

>Saccharomyces cerevisiae (EGA73998.1)

MLNVLLRRKAFCLVTKKGMATATTAAATHTPRLKTFKVYRWNPDEPSAKPHLQSYQVDLNDCGPMVLDALLKIKDEQDSTLTFRRSCREGICGSCAMNIGGRNTLACICKIDQNESKQLKIYPLPHMFIVKDLVPDLTNFYQQYKSIQPYLQRSSFPKDGTEVLQSIEDRKKLDGLYECILCACCSTSCPSYWWNQEQYLGPAVLLQAYRWLIDSRDQATKTRKAMLNNSMSLYRCHTIMNCTRTCPKGLNPGLAIAEIKKSLAFA

+3

>Caenorhabditis elegans (NP_495992.1)

MLARSARLLHSAELAANAIRAASGAPATAAAAEASFPSTDDVAAKTKKTGNRIKTFEIYRFNPEAPGAKPTVQKFDVDLDQCGTMILDALIKIKNEVDPTLTFRRSCREGICGSCAMNIGGQNTLACICKIDSDTSKSTKIYPLPHMFVVKDLVPDMNLFYAQYASIQPWIQKKTPLTLGEKQMHQSVAERDRLDGLYECILCACCSTSCPSYWWNADKYLGPAVLMQAYRWVIDSRDDYATERLHRMHDSFSAFKCHTIMNCTKTCPKHLNPAKAIGEIKSLLTGFTSKPAAEPSAF

>Canis lupus (NP_001239146.1)

MAAVVGVSLKRRFPAAALGGACLQACRGAQTAAATAPRIKKFAIYRWDPDKTGDKPHMQTYEIDLNKCGPMVLDALIKIKNEIDSTLTFRRSCREGICGSCAMNINGGNTLACTRRIDTNLSKVSKIYPLPHMYVIKDLVPDLSNFYAQYKSIEPYLKKKDESQEGKQQYLQSIEDREKLDGLYECILCACCSTSCPSYWWNGDKYLGPAVLMQAYRWMIDSRDDFTEERLAKLQDPFSLYRCHTIMNCTRTCPKGLNPGKAIAEIKKMMATYKGKKASV

>Equus caballus (NP_001157295.1)

MAAAVGVSLRRRFPATALGGACLQACRGAQTAAATAPRIKKFAIYRWDPDKTGDKPHMQTYEIDLNKCGPMVLDALIKIKNEIDSTLTFRRSCREGICGSCAMNINGGNTLACTRRIDTNLSKVSKIYPLPHMYVIKDLVPDLSNFYAQYKSIEPYLKKKDESQGGKQQYLQSIEEREKLDGLYECILCACCSTSCPSYWWNGDKYLGPAVLMQAYRWMIDSRDDFTEERLAKLQDPFSLYRCHTIMNCTKTCPKGLNPGKAIAEIKKMMATYKEKKASA

>Bos taurus (NP_001035573.1)

MAAVVALSLRRRFPAAALGGARLQACRGAQTAAAAAPRIKKFAIYRWDPDKTGDKPHMQTYEIDLNNCGPMVLDALIKIKNEIDSTLTFRRSCREGICGSCAMNINGGNTLACTRRIDTNLSKVSKIYPLPHMYVIKDLVPDLSNFYAQYKSIEPYLKKKDESQGGKEQYLQSIEDREKLDGLYECILCACCSTSCPSYWWNGDKYLGPAVLMQAYRWMIDSRDDFTEERLAKLQDPFSLYRCHTIMNCTQTCPKGLNPGKAIAEIKKMMATYKEKQASA

>Gallus gallus (NP_001264968.1)

MAAAVVGVSLRRGVPARFLRAGLRPICRGAQTAAAATSRIKKFSIYRWDPDKPGDKPRMQTYEVDLNKCGPMVLDALIKIKNELDSTLTFRRSCREGICGSCAMNIAGGNTLACTKKIDPDLSKTTKIYPLPHMYVVKDLVPDLSNFYAQYKSIEPYLKKKDESKQGKEQYLQSIEDRQKLDGLYECILCACCSTSCPSYWWNGDKYLGPAVLMQAYRWMIDSRDDYTEERLAQLQDPFSLYRCHTIMNCTRTCPKGLNPGKAIAEIKKMMATYKEKAAAA

>Homo sapiens (NP_002991.2)

MAAVVALSLRRRLPATTLGGACLQASRGAQTAAATAPRIKKFAIYRWDPDKAGDKPHMQTYEVDLNKCGPMVLDALIKIKNEVDSTLTFRRSCREGICGSCAMNINGGNTLACTRRIDTNLNKVSKIYPLPHMYVIKDLVPDLSNFYAQYKSIEPYLKKKDESQEGKQQYLQSIEEREKLDGLYECILCACCSTSCPSYWWNGDKYLGPAVLMQAYRWMIDSRDDFTEERLAKLQDPFSLYRCHTIMNCTRTCPKGLNPGKAIAEIKKMMATYKEKKASV

>Thermosynechococcus elongatus (NP_682544.1)

MTIQLRIRRQGPDKNAYWQTFELEIDPSLTILDALIQIKESQDGSLSCRKNCRNTICGSCAMTINGRSALACQQSILAELANSPVPNQIAIAPLGNLPVLKDLVVDMSDFWQKLSAVNPYVSTAARQVPEREFLQSPSDRAKLNASGNCILCGACYGACNAVEVNPAFVGPHALAKAARLVADTRDSDTDQRLDQYNTATSGVWGCTRCFNCNTVCPVGVQPLDRISEIKQAILARSGEATHNQDRPLRHRQVLLELVKEGGWVDERQFGLRVVGNGFRDVGGVMSLVPLGWRLLRRGKFPLRFEKSAGQAQIKAVVTALQQKQPYSKIEAKKKGMKC

>Synechococcus (YP_007061931.1)

MELNVQILRQAPDAPPYSQTFRLDISPGATVLDCLNQIKWSQDGTLAFRKNCRNTICGSCAMRINGRAALACQNSVANELKWSLQPQTITIAPLGNLPIVRDLIVDMQPFWQNLDDVNPYVSAAARNLGEREFRQTPDQRAKLNEMGNCIMCGACYSDCNGKEVNPEFVGPHALAKAYRMLADNRDQAQAERLEQYNSSAGVWGCTRCFNCNTVCPMDVAPLDQISKIKGEILEQAQGLPAPRPIRHRQVLVDLVKRGGWVDERKFGLEVVGNKFRDVRGVLSLLPLGWRMVKRGKFPMRFEKSAGQAQVQGLIESVQSLEKSQSDQGVRSHE

>Acaryochloris marina (YP_001517973.1)

MQVLFKISRQQQQSAVKVQTYSLDVDPSTTILDCLNLIKWQQDGSLAFRKNCRNTICGSCSMRINHRAALACQQNVKAEVARSYSAWNKQNGADSDRENPDSIPVIEIAPMGNMPVIKDLVVEMDQFWQKLDQVEPYVSSASRQVPEREFLQSPEDRSKLDQMGNCILCGACYSDCNALPANGSFVGPHALAKAHRLLADTRDQATEHRLEQYNQDIQGVWGCTRCFNCNTVCPMGVAPLDQISKIKSQVLANPTLPDSRPLRHRRVLVDLVQEGGWIDERKFGLKVVGNNFKDLPGLFSLVPLAGQMLRRQKLPIKFDASTGTDEVRGLIEAVKKRQSKTASQSESTR

>Oscillatoria (WP_007356679.1)

MQIRFKITRQTQNSAPEFQTYTIDAEPGNTVLDCLNRIKWEQDGTLAFRKNCRNTICGSCSMRINGRSALACKENIGSEVKRLQEIAKANSAQTPTDSIPEITIAPMGNMPVIKDLVVDMTSFWNNLEAVEPYVSTEARKIPEREFLQTPEEREKLNQTGNCILCGACYSECNAREVNPEFVGPHALAKAYRMVADNRDSDTENRLEKYNQGTVGVWGCTRCYYCNSVCPMDVAPMDQIGKIKQDILDRKDAQASRSIRHRKVLIDLVKQGGWIDERKFGVQVVGNYFRDIKGLLSLGPLGLRMIARGKFPLSFEPSEGRETVRSLIESVQNLED

>Lyngbya (WP_009785496.1)

MEVQFKIIRQTQDSSPRIQTYKLEVEPGNTILECLNRIKWELDGTLAFRKNCRNTICGSCGMRINGRSALACKENITSELARLEQIAASSSAPTASNGIPEITIAPMGNMPVIKDLVVDMKRFWGNLEKVEPYVSTQSRDIPEREFLQTPEERAKLNQMGNCILCGACYSECNALEVNPEFAGPHALAKAQRMIADNRDDHTEDRLEHYNQGTEGVWGCTRCFNCNTACPMEVAPMDQITKIKHEILERKNAGDSTAIRHRKVLVDLVKQGGWVDERKFGILVIGNYFRDVKALLGLGPLGFKMLTRGKFPLSFEPSEGTKTVRSLIESVQRLEQQK
