# Supplementary material for: Evolution and Functional Implications of the Tricarboxylic Acid Cycle as Revealed by Phylogenetic Analysis
Source: Genome Biol Evol. 2014 Oct 1;6(10):2830–48. doi: 10.1093/gbe/evu221 (PMC4224347; doi:10.1093/gbe/evu221)
Supplement: Supplementary Data [file supp_evu221_Supplemental_DataSet_4.doc]

FUMARASE

MITOCHONDRIAL

>Arabidopsis thaliana (NM_130319)

MSIYVASRRLSGGTTVTALRYATSLRSYSTSFREERDTFGPIQVPSDKLWGAQTQRSLQNFEIGGERERMPEPIVRAFGVLKKCAAKVNMEYGLDPTIGKAIMQAAQEVAEGKLNDHFPLVVWQTGSGTQSNMNANEVIANRAAEILGRKRGEKCVHPNDHVNRSQSSNDTFPTVMHIAAATEINSRLIPSLKTLHSTLESKSFEFKDIVKIGRTHTQDATPLTLGQEFGGYATQVKYGLNRVTCTLPRLYQLAQGGTAVGTGLNTKKGFDVKIAAAVAEETNLPFVTAENKFEALAAHDACVETSGSLNTIATSLMKIANDIRFLGSGPRCGLGELVLPENEPGSSIMPGKVNPTQCEALTMVCAQVMGNHVAVTVGGSNGHFELNVFKPVIASALLHSVRLIADASASFEKNCVRGIEANRERISKLLHESLMLVTSLNPKIGYDNAAAVAKKAHKEGCTLKEAALNLGVLTAEEFDTLVVPEKMIGPSD

>Arabidopsis thaliana (NM_180835)

MAALTMQFEGEKKNVSEVADVTLKQEDEQQERRSYSTPFREERDTFGPIQVPSDKLWGAQTQRSLQNFEIGGDRERMPEPIVRAFGVLKKCAAKVNMEYGLDPMIGEAIMEAAQEVAEGKLNDHFPLVVWQTGSGTQSNMNANEVIANRAAEILGHKRGEKIVHPNDHVNRSQSSNDTFPTVMHIAAATEITSRLIPSLKNLHSSLESKSFEFKDIVKIGRTHTQDATPLTLGQEFGGYATQVEYGLNRVACTLPRIYQLAQGGTAVGTGLNTKKGFDVKIAAAVAEETNLPFVTAENKFEALAAHDACVETSGSLNTIATSLMKIANDIRFLGSGPRCGLGELSLPENEPGSSIMPGKVNPTQCEALTMVCAQVMGNHVAVTIGGSNGHFELNVFKPVIASALLHSIRLIADASASFEKNCVRGIEANRERISKLLHESLMLVTSLNPKIGYDNAAAVAKRAHKEGCTLKVNNKLLTFSSLNKSEFKPIFSKRKHVHVCYNIFVVLFWI

>Arabidopsis lyrata (XM_002864039.1)

MAALIMQFEGEKKNLSEVADVTLKQDQDEQQRRSYSTSFREERDTFGPIQVPSDKLWGAQTQRSLQNFKIGGDRERMPEPIVRAFGVLKKCAAKVNMEYGLDPMIGEAIMEAAQEVAEGKLNDHFPLVVWQTGSGTQSNMNANEVIANRAAEILGHKRGEKIVHPNDHVNRSQSSNDTFPTVMHIAVATEITSRLIPSLKNLHSSLESKSFEFKDIVKIGRTHTQDATPLTLGQEFGGYATQVKYGLSRVACTLPRIYQLAQGGTAVGTGLNTKKGFDVKIAAAVAEETNLPFVTAENKFEALAAHDACVETSGSLNTIATSLMKIANDIRFLGSGPRCGLGELSLPENEPGSSIMPGKVNPTQCEALTMVCAQAMGNHVAVTIGGSNGHFELNVFKPVIASALLHSIRLIADASASFDKNCVRGSEANRERISKLLHESLMLVTSLNPKIGYDNAAAVAKKAHKEGCTLKHAAMKLGVLTSEEFDTLVVPEKMIGPSD

>Vitis vinifera (XM_002272997)

MMMYAASRRLCGGSAGSPLLGLRYMCLRSLSTSFREEKDTFGPILVPSDKLWGAQTQRSLQNFDIGGEREQMPEPIIRAFGILKKCAAKVNMEYGLDPSIGKAIMQAAQEVSEGKLNDHFPLVIWQTGSGTQSNMNANEVIANRAAEILGHQRGEKYVHPNDHVNRSQSSNDTFPTVMHIAAATEINSRLIPSLKHLETALHSKSIEFKDIIKIGRTHTQDATPLTLGQEFSGYYTQVKYGIDRVTCTLPRMYQLAQGGTAVGTGLNTKKGFDVKIARAVAEETNLPFVTAENKFEALAAHDAFVETSGSVNTVATSLMKIANDIRLLGSGPRCGLGELILPENEPGSSIMPGKVNPTQCEALTMVCAQVMGNHVAITVGGSNGHFELNVFKPLIASGLLRSIRLLGDASASFEKNCVRGIQANKERILKLLHESLMLVTSLNPKIGYDNAAAVAKKAHKEGRTLKEAALNLGVLTSEEFDQLVVPEKMIGPSD

>Vitis vinifera (XM_002283132)

MAMLRAYRCGSYRSLMPQFMEMLRYDCLRAFSTSFREEKDTLGTVLVPDDKLWGAQTQRSLQNFEIGGESERMPEPIIRAFGILKKCAAKVNMEYGLDPSIGKAIMQAAQEVAEGKLNDHFPLVIWQTGSGTQSNMNANEVIANRAAEILGHKRGEKFVHPNDHVNRSQSSNDTFPTVMHIAAGVEINSRFIPKLKQLHTSLHSKSLEFKDIIKIGRTHTQDATPLTLGQEFSGYSTQVKYGIDRVMCTLPRMYQYLAQGGMAVGTGLNTKKGFDVKIAATVAEETNLPFITSENKFEALAAHDAFVETSGALNTICASLMKIGNDIRLLGSGPRCGLGELTLPDNEPGSSIMPGKVNPTQCEALTMVCAQVMGNHVAITVGGSNGHFELNVYKPLIASGLLRSLRLLGDASASFEKNCIRGIQANQERISKLLHESLMLVTSLNPKIGYDNAAAVAKKAHKEGTTLKHAALNLGVLTSEEFEKLVVPEKMIGPSD

>Glycine max (XM_003518904)

MALYVLSRRLSAGSNSTTLLALRFVSSTRSYSSSFREERDTFGAIQVPSEKLWGAQTQRSLQNFDIGGPRERMPEPIIRAFGVLKKCAAKVNMEYGLDPAVGKAIMQAAQEVAEGKLNDHFPLVVWQTGSGTQSNMNANEVIANRAAEILWHKRGGKLVHPNDHVNRSQSSNDTFPTVMHIAAAMEINSRLIPSLKTLHGTLNSKSIEFKDIVKIGRTHTQDATPLTLGQEFSGYTTQVKYGIDRVIGTLPHMYQLAQGGTAVGTGLNTKKGFDAKIAAAVAEETNLPFVTAENKFEALAAHDAFVETSGALNTIAASLMKIANDIRLLGSGPRCGLGELILPENEPGSSIMPGKVNPTQCEALTMVCAQVIGNHVAVTVGGSNGHFELNVFKPMIASCLLHSLRLLGDSSASFEKNCVRGIQANRERISKLLHESLMLVTSLNPKIGYDKAAAVAKTAHKEGCTLKEAALKLGVLSSEDFDKLVVPEKMLGPSD

>Glycine max (XM_003518909)

MALSFREERDTFGAIQVPSEKLWGAQTQRSLQNFDIGGPRERMPEPIIRAFGVLKKCAAKVNMEYGLDPNVGEAIMQAAQEVAEGKLNDHFPLVVWQTGSGTQSNMNANEVIANRAAEILGHKRGEKFVHPNDHVNRSQSSNDTFPTVMHIAAATEINSRLIPSLKTLHDTLNSKSVEFKDIVKIGRTHTQDATPLTLGQEFSGYTTQVKYGIDRVIDTLPRMYQLAQGGTAVGTGLNTKKGFDAKIAAAVAEETNLPFVTAENKFEALAAHDAFVETSGALNTVVASLMKIANDIRLLGSGPRCGLGELILPENEPGSSIMPGKVNPTQCEALTMVCAQVMGNHVAITVGGSNGHFELNVFKPMIANCLLHSLRLLGDSSASFEKNCLRGIQANRERISKLLHESLMLVTSLNPKIGYDKAATVAKTAHKEGTTLKEAALKLGVLCSEDFDKLVVPEKMLGPSD

>Medicago truncatula (XM_003591424)

MALYVLSRRLSTKSTSPTLFTFRYFSSTTTFREERDTFGPIQVPSDKLWGAQTQRSLQNFDIGGPRERMPEPIIRSFGILKKCAAKVNMEYGLDPTIGKAIMKAAQEVADGKHNDHFPLVVWQTGSGTQSNMNANEVIANRASEILGHKRGEKFVHPNDHVNMSQSSNDTFPTVMHIAAGTEINSRLIPSLKTLHATLDAKSIEFKDIVKIGRTHTQDATPLTLGQEFSGYTTQLKYSLDRVITILPRMYQLAQGGTAVGTGLNTKKGFDVKVSLAVATETNLPFVTAENKFEALAAHDSFVETSGALNTISASLMKIANDIRLLGSGPRCGLGELILPENEPGSSIMPGKVNPTQCEALTMVCAQVMGNHVAITVGGSNGHFELNVFKPVIASNLLQSVRLLGDSCASFEKNCVRGIQANRERISKLLHESLMLVTSLNPKIGYDKAAAVAKKAHKEGSTLKEAALKLGVLTSEEFDSLVVPEKMLGPSD

>Populus trichocarpa (XM_002302698)

MAMYIVSRRLSTGSTTWRYGNWLRSYSTAFREERDTFGPISVPADKLWGAQTQRSLQNFDIGGDRERMPEPIIRAFGILKKCAAKVNMQYGLDPSIGKAIMQAAHEVAEGKLSDHFPLVVWQTGSGTQSNMNANEVIANRAAEILGHKRGEKFVHPNDHVNRSQSSNDTFPTVMHIAAATEINSRLIPKLKTLHLTLHSKSVEFKDIVKIGRTHTQDATPLTLGQEFSGYTTQVKYGIDRVMCTLPHMYQLAQGGTAVGTGLNTKKGFDVKIASAVAEETNLPFVTAENKFEALAAHDAFVETSGALNTVATSLMKIANDIRLLGSGPRCGLGELILPENEPGSSIMPGKVNPTQCEALTMVCAQVMGNHVAITVGGSNGHFELNVFKPMIASGLLHSIRLLGDASASFEKNCVRGIQANRERISKLLHESLMLVTSLNPKIGYDNAAAVAKLAHKEGSTLKEAALKLGMLTSEEFDTLVVPEKMIGPTD

>Solanun tuberosum (X91615)

MAMLNAARRLSAGSALSDSLRYTSSWRFFSTSFREERDTFGPILVPSDKLWGAQTQRSLQNFEIGGDRERMPEPIIRAFGIIKKCAAKVNMDYGLDQSIGKAIMQAAEEVAEGKLNDHFPLVVWQTGSGTQSNMNANEVIANRAAEILGHNRGDKHVHPNDHVNKSQSSNDTFSTVMHIAAAMELNKRLVPNLETVAYSLNSKSVEFKDIIKIGRTHTQDATPLTLGQEFSGYATQVKYGIDRVLCTLQRMYQLAQGGTAVGTGLNTKKGFDIKIAAAVAEETNFPFVTAENKFEALAAHDAFAETSGALNTLAASLMKIGNDIRFLGSGPRCGLGELSLPENEPGSSIMPGKVNPTQCEALTMVCAQVMGNHVAVTVGASNGHFELNVFKPMIANALLHSVRLLGDASASFEKNCVRDIQANRDRIAKLLHESLMLVTCLNPKIGYDNAAAVAKKAHKEGTSLKEAALNLGVLTSEEFDQLVVPEKMIGPTD

>Solanun lycopersicum (XP_004247395.1)

MAMLNAARRLSAGSALSDSLRYTSSSWRFFSTSFREERDTFGPILVPSDKLWGAQTQRSLQNFEIGGDRERMPEPIIRAFGIIKKCAAKVNMDYGLDQSIGKAIMQAAEEVAEGKLNDHFPLVVWQTGSGTQSNMNANEVIANRAAEILGYNRGDKHVHPNDHVNKSQSSNDTFPTVMHIAAAMELNKRLVPNLKQLHTSLNSKSVEFKDIIKIGRTHTQDATPLTLGQEFSGYATQVKYGIDRVLGTLPRMYQLAQGGTAVGTGLNTKKGFDIKIAAAVAEETNLPFVTAENKFEALAAHDAFAETSGALNTVAASLMKIGNDIRFLGSGPRCGLGELSLPENEPGSSIMPGKVNPTQCEALTMVCAQVMGNHVAVTVGASNGHFELNVFKPMIANALLHSVRLLGDASASFEKNCVRDIQANRDRIAKLLHESLMLVTCLNPKIGYDNAAAVAKKAHKEGTSLKEAALNLAVLTSEEFDQLVVPEKMIGPTD

>Zea mays (NM_001152824)

MAMALRRLAGVSGSPSASSAAATVLLRSALTRPISTGFREERDTFGPIQVPNDKLWGAQTQRSLQNFDIGGERERMPVPIIRAFGVLKKCAAKVNMDYGLDPTIGKAIIQAAEEVAEGKLDDHFPLVIWQTGSGTQSNMNANEVIANRASEILGHKRGEKFVHPNDHVNRSQSSNDTFPTVMHIATAVEIHSRFIPSLQQLHDSLHSKTIEFKDIIKIGRTHTQDATPLTLGQEFSGYTTQVKYGIDRINCTLPRMYQLAQGGTAVGTGLNTKKGFDAKIAAAVAEETNLPFVTAENKFEALAAHDAFVESSGAVNTISASLMKIANDIRLLGSGPRCGLGELILPENEPGSSIMPGKVNPTQCEALTMVCAQVMGNHVGVTIGGANGHFELNVFKPMIAAGLLRSLRLLGDASVSFEKNCVRGIQANHKRISQLLHESLMLVTSLNPKIGYDNAAAVAKKAHKEGTTLKEAALDLGVLTEQEFHELVVPEKMIGPSD

>Brachypodium distachyon (XM_003557905)

MAMVLRRLAGASGSPSAAALLFRPALTRPISTGFREERDTFGPIRVPSDKLWGAQTQRSLQNFDIGGERERMPVPIIRAFGVLKKCAAKVNMEYGLDPTIGKAIMQAAEEVAEGKLDDHFPLVIWQTGSGTQSNMNANEVIANRAAEILGHKRGDKFVHPNDHVNRSQSSNDTFPTVMHIAAAVEINSRFIPSLEQLHKSLHSKSDEFKDIIKIGRTHTQDATPLTLGQEFSGYATQVKYGIDRIACTLPRMYQLAQGGTAVGTGLNTKKGFDCKIAAAVAEETDLPFVTAENKFEALAAHDAFVESSGAVNTISASLMKIANDIRLLGSGPRCGLGELSLPENEPGSSIMPGKVNPTQCEALTMVCAQVMGNHVGVTIGGSNGHFELNVFKPMIAAGLLRSLRLLGDASVSFEKNCVRGIEANHKRISQLLHESLMLVTSLNPKIGYDNAAAVAKKAHKEGTTLKEAALSLGVLTEKEFHELVVPEKMIGPSD

>Hordeum vulgare (AK251785)

MAMVLRRLAGASGSPSAAALLLRPALTRPISTDFREERDTFGPIRVPNDKLWGAQTQRSLQNFDIGGERERMPVPIIRAFGVLKKCAAKVNMEYGLDPTIGKAIMQAAEEVAEGKLDDHFPLVIWQTGSGTQSNMNANEVIANRAAEILGHKRGDKFVHPNDHVNRSQSSNDTFPTVMHIAAAVEINSRFIPSLEQLHKSLHSKSDEFKDIIKIGRTHTQDATPLTLGQEFSGYATQVKYGIDRIACTLPRMYQLAQGGTAVGTGLNTKKGFDGKIAAAVAEETELPFVTAENKFEALAAHDAFVESSGAVNTISASLMKIANDIRLLGSGPRCGLGELILPENEPGSSIMPGKVNPTQCEALTMVCAQVMGNHVGVTIGGSNGHFELNVYKPMIAAGLLRSLRLLGDASVSFEKNCVRGIEANHKRISQLLHESLMLVTSLNPKIGYDNAAAVAKKAHKEGTTLKEAALSLRVLTEKEFHELVVPEKMIGPSD

>Oryza sativa (CT835200)

MQAAQAVAEGQLDDHFPLVIWQTGSGTQSNMNANEVIANRAAEILGHKRGEKFVHPNDHVNRSQSSNDTFPTVMHIAAATEINSRFVPSLQQLHKSLDSKSVEFQDIIKIGRTHTQDATPLTLGQEFSGYATQVKYGIDRIVCTLPRMYQLAQGGTAVGTGLNTKKGFDVKIAAAVAEEMELPFVTAENKFEALAAHDAFVESSGAVNTISASLMKIANDIRLLGSGPRCGLGELILPENEPGSSIMPGKVNLTQCEALTMVCAQVMGNHVGVTVGGRNGHFELNVFKPMIAAGLLRSLRLLGDASVSFEKNCVRGIQANHKRISQLLHESLMLVTSLNPKIGYDNAAAVAKKAHKEGSTLKEAALDLGVLTESEFHELVVPEKMIGPSD

>Chlamydomonas reinhardtii (XM_001689899)

MLGDAPVLPAGRTLQRRADDTFSMMAWELLRGSACSSSLATSSSSRAVLLLTRLLRTTAGANAISSVMRARRPQDTMGSVEVPANKYWGAQTQRSLLNFRIGVSRMPEPLIRALGLVKRATAEVNTAAGLLPRHVGQAVMAAASEVAEGRLSEHFPLVIWQTGSGTQTNMNANEVIARRCPQQQPQQAVELRAVHPNDHVNRGQSSNDTFPTAMHVAVALQVHRHLLPALAGLQERLAAKADEFAGIIKIGRTHTQDATPLTLGQEFSGYAAQVKYGMERVQQAMPRVYELAQGGTAVGTGLNTFQGFAEAVAAVLARDTGLPLVTAANKFEALAAHDALVHLSGGLNTLTVSLMKIGNDVRLLGSGPRCGLGELRLPANEPGSSIMPGKVNPTQVEALTMVCAQVMGNHMAVTVAGAGGQFELNTYKPLIAAALLRSVGLLADAAWSFSEHLVAGLEADGPRIAEHVRRSLMLVTVLNPIIGYEAAAAVAKRAHAEGISLREAALALGVVGSADEFDRLVRPELMLRPSTLSSGAADGQQGGSST

>Volvox carteri (XM_002952102)

MNLGGHKPITCAVTRPTQHPLTQHPPTHPQPLRTRHKPTFATSTNDQRPTTNPIQDTMGPVEVPADRYWGAQTQRALEHFQFGDPHTERVPEALIRALGLVKRAAAEVNTQAGLLRPELGRAVMAAAGEVAEGRLGAHFPLLTWQTGSGTQTNMNANEVIARRAAEILAATSPNGATAVHPNDHVNLGQSSNDTFPTAMHVAVALQVHHRLLPALQGLQEALAAKSHEFARIIKIGRTHTQDATPLTLGQEFSGYATQVQYGIERVRHAMGHVYMLAQGGTAVGTGLNTFRGFAEAVAEALARDTGLPFVTAPNKFEALAAHDAAVHLSGALNTLADGYTVHLQIGNDIRLLGSGPRCGIGELLLPANEPGSSIMPGKVVNPTQVEALIMVCALVMGNHTAVTVAGSAAGGQLELNTCKPLIAAAVLRSTGLLADAVRSFTVHLVSGLRADEARIARLVLGALMLVTALNPVIGYDAAARVAKKAHSEGITLREAALDLGLVSGEEFDRVVQPSKMLAPYDLPAFVGSGGGDGDAAAAAAINTRSFTEDGARPDGSFGS

>Ostreococcus tauri (XM_003081524)

MTRHTPPRRIVQSHPARHRRARRRRRAVARARSRRDTDAMPFADRLVELMRTPREGEARALVSHGVSLGRHGDVAGAGTEGETRRESDTMGVVDVPMHKLWGAQTQRSLENFKIGGEKMPIAIVRSLAIVKYAAATVNEREGRLLGSCEDHWMNTFPLVRVPNRIGTQTNMNLNEVICNSPIHPNDHVNMSQSSNDTFPTAMSIATAHEVQERLIPALKMLQEALHVKVLAWGDIVKIGRTHLQDAVPITLAQEFSGYEQQVKNSLLRAKGALVHLLELAIGGTAVGTGLNAPPRFGENMAAEISMLTGLPFVSAPNKFEALAAHDAQSTLSGMLKTIAISLLKIANDIRLLGSGPRAGLGELCLPENEPGSSIMPGKVNPTQCESLMQVCAQVIGNDLAVTIGGSASSHFELNVAKPLIAHNNLNSIALLSDSLESFTKNCVVGIEPNMERIDALMRSSLMLVTSLVPKIGYDKAAKISKKAHAEGLTLREAGVQLGLLTNEQFDEWIRPDEMTRPEGLQSKL

>Micromonas pusilla (XM_003055560)

MGVVRVPANRYWGAQTQRSLQNFKIGGEKMPMEILRALAIVKHAAATVNQDLGKLDAPRAAAIRAAALEVIEGKLDENFPLVVWQTGSGTQTNMNVNEVISNRAIEIMGGVVGSKSPVHPNDHVNMSQSSNDTFPTAMSVATAYEVQEGLMPALRVLRDDLDAKSEAWSGIVKIGRTHLMDAVPITLGQEFGGYAQHVRNSLLRAKASMVHLTELAIGGTAVGTGINSHPEYAERMARQISKLTGLPFVSAPNKFESLAAHDAQTALSGMLKTMALSLLKISNDVRMLGSGPRAGLGELSLPANEPGSSIMPGKVNPTQCEAMMMVCAQVVGNDMGVAMGGAAGSHFELNVAKPLIAYNNLQSIKLLSEASVSFAENCVRGIEPNIERIDELMRSSLMLVTALNPHIGYDNAAKIAKKAHAEGSTLKEAGVRLGLLTSEQFDEWVRPELMTAPEVPPTSKL

>Mus musculus (NM_010209)

MYRALRLLARSRRLLRVPSAGAAVSGEATTLPRCAPNVARMASQNSFRVEFDTFGELKVPTDKYYGAQTVRSTMNFKIGGATERMPIPVIQAFGILKRAAAEVNQEYGLDPKIASAIMKAADEVAEGKLNDHFPLVVWQTGSGTQTNMNVNEVISNRAIEMLGGELGSKKPVHPNDHVNKSQSSNDTFPTAMHIAAAVEVHKVLLPGLQKLHDALSAKSKEFAQVIKIGRTHTQDAVPLTLGQEFSGYVQQVQYAMVRIKAAMPRIYELAAGGTAVGTGLNTRIGFAEKVAAKVAALTGLPFVTAPNKFEALAAHDALVELSGAMNTAACSLMKIANDIRFLGSGPRSGLGELILPENEPGSSIMPGKVNPTQCEAMTMVAAQVMGNHVAVTVGGSNGHFELNVFKPMMIKNVLHSARLLGDASVSFTDNCVVGIQANTERINKLMNESLMLVTALNPHIGYDKAAKIAKTAHKNGSTLKETAIELGYLTAEQFDEWVKPKDMLGPK

>Rattus norvegicus (NM_017005)

MNRAFCLLARSRRFPRVPSAGAVLSGEAATLPRCAPNVVRMASQNSFRIEYDTFGELKVPTDKYYGAQTVRSTMNFKIGGATERMPIPVIKAFGILKRAAAEVNQEYGLDPKIASAIMKAADEVAEGKLNDHFPLVVWQTGSGTQTNMNVNEVISNRAIEMLGGELGSKKPVHPNDHVNKSQSSNDTFPTAMHIAAALEVHQVLLPGLQKLHDALSAKSKEFAQVIKIGRTHTQDAVPLTLGQEFSGYVQQVQYAMERIKAAMPRIYELAAGGTAVGTGLNTRIGFAEKVAAKVAALTGLPFVTAPNKFEALAAHDALVELSGAMNTTACSLMKIANDIRFLGSGPRSGLGELILPENEPGSSIMPGKVNPTQCEAMTMVAAQVMGNHVAVTVGGSNGHFELNVFKPMMIKNVLHSARLLGDASVSFTENCVVGIQANTERINKLMNESLMLVTALNPHIGYDKAAKIAKTAHKNGSTLKETAIELGYLTAEQFDEWVKPKDMLGPK

>Bos taurus (NP_001069271.1)

MDRALRLLARSRLLSRNPGSAPKPGLGPGGPAVLLLRPPNAARMASQNSFRIEYDTFGELKVPNDKYYGAQTVRSTMNFKIGGMTERMPIPVIKAFGILKRAAAEVNQDYGLDPKIANAIMKAADEVAEGKLNDHFPLVVWQTGSGTQTNMNVNEVISNRAIEILGGELGSKKPVHPNDHVNKSQSSNDTFPTAMHIAAAIEVHEVLLPGLQKLHDALEAKSKEFDQIIKIGRTHTQDAVPLTLGQEFSGYVQQVKYATTRIKAAMPRIYELAAGGTAVGTGLNTRIGFAEKVAAKVAVLTGLPFVTAPNKFEALAAHDALVELSGAMNTTACSLMKIANDIRFLGSGPRSGLGELILPENEPGSSIMPGKVNPTQCEAMTMVAAQVMGNHVAVTVGGSNGHFELNVFKPMMIKNVLHSARLLGDASVSFTENCVVGIQANTERISKLMNESLMLVTALNPHIGYDKAAKIAKTAHKNGSTLKATAIELGYLTAEQFDEWVKPKDMLGPK

>Homo sapiens (NP_000134.2)

MYRALRLLARSRPLVRAPAAALASAPGLGGAAVPSFWPPNAARMASQNSFRIEYDTFGELKVPNDKYYGAQTVRSTMNFKIGGVTERMPTPVIKAFGILKRAAAEVNQDYGLDPKIANAIMKAADEVAEGKLNDHFPLVVWQTGSGTQTNMNVNEVISNRAIEMLGGELGSKIPVHPNDHVNKSQSSNDTFPTAMHIAAAIEVHEVLLPGLQKLHDALDAKSKEFAQIIKIGRTHTQDAVPLTLGQEFSGYVQQVKYAMTRIKAAMPRIYELAAGGTAVGTGLNTRIGFAEKVAAKVAALTGLPFVTAPNKFEALAAHDALVELSGAMNTTACSLMKIANDIRFLGSGPRSGLGELILPENEPGSSIMPGKVNPTQCEAMTMVAAQVMGNHVAVTVGGSNGHFELNVFKPMMIKNVLHSARLLGDASVSFTENCVVGIQANTERINKLMNESLMLVTALNPHIGYDKAAKIAKTAHKNGSTLKETAIELGYLTAEQFDEWVKPKDMLGPK

>Gorilla gorilla (XP_004028710.1)

MYRALRLLARSRRLVRAPAAALAAAPGLGGAAVPSFWPPNAARMASQNSFRIEYDTFGELKVPNDKYYGAQTVRSTMNFKIGGVTERMPTPVIKAFGILKRAAAEVNQDYGLDPKIANAIMKAADEVAEGKLNDHFPLVVWQTGSGTQTNMNVNEVISNRAIEMLGGELGSKIPVHPNDHVNKSQSSNDTFPTAMHIAAAIEVHEVLLPGLQKLHDALDAKSKEFAQIIKIGRTHTQDAVPLTLGQEFSGYVQQVKYAMTRIKAAMPRIYELAAGGTAVGTGLNTRIGFAEKVAAKVAALTGLPFVTAPNKFEALAAHDALVELSGAMNTTACSLMKIANDIRFLGSGPRSGLGELILPENEPGSSIMPGKVNPTQCEAMTMVAAQVMGNHVAVTVGGSNGHFELNVFKPMMIKNVLHSARLLGDASVSFTENCVVGIQANTERINKLMNESLMLVTALNPHIGYDKAAKIAKTAHKNGSTLKETAIELGYLTAEQFDEWVKPKDMLGPK

>Canis lupus (XP_537215.1)

MYRAVRLLARSRRLVRAPGSASAPGLGEAAVLLLRPPDAARMASQNSFRIEFDTFGELKVPNDKYYGAQTVRSTMNFKIGGVTERMPIPVIKAFGILKRAAAEVNQDYGLDSKIAEAIMKAADEVAEGKLNDHFPLVVWQTGSGTQTNMNVNEVISNRAIEMLGGELGSKKPVHPNDHVNKSQSSNDTFPTAMHIAAAIEVHEVLLPGLQKLHDALNAKSKEFAQIIKIGRTHTQDAVPLTLGQEFSGYVQQVKYAMTRIKAAMPRIYELAAGGTAVGTGLNTRIGFAEKVAAKVAALTGLPFVTAPNKFEALAAHDALVELSGAMNTTACSLMKIANDIRFLGSGPRSGLGELILPENEPGSSIMPGKVNPTQCEALTMVAAQVMGNHVAVTVGGSNGHFELNVFKPMMIKNVLHSARLLGDAAVSFTENCVMGIQANTERISKLMSESLMLVTALNPHIGYDKAAKIAKTAHKNGSTLKATAIELGYLTAEQFDEWVKPKDMLGPK

>Drosophila melanogaster (NP_572339.1)

MVLPLLQRSTLRGVQQMTKPWAAIGSLRLASQEFRVESDTFGELKVPADKYYGAQTMRSQINFPIGGATERMPKPVVQAMGILKKAAAEVNKEFGLDSKVSEAISKAADDVISGKLYDDHFPLVIWQTGSGTQSNMNVNEVISNRAIELLGGKLGSKTPVHPNDHVNKSQSSNDTFPTAIHISVALELNNNLKPAIKTLHDALRAKSEEFKDIIKIGRTHTMDAVPLTLGQEFSGYAQQLAYAQERIDACLPRVYELALGGTAVGTGLNTRKGFAEKCAAKIAELTSLPFVTAPNKFEALAARDAMVEVHGVLNTIAVSLMKIANDIRFLGSGPRCGLGELSLPENEPGSSIMPGKVNPTQCESLTMLSAQVMGNQVAVTIGGSNGHFELNVFKPLIVSNVLRSIRLLSDGSRTFTANCVNGIQANRENIAKIMNESLMLVTALNPHIGYDKAAKIAKTAHKNGTTLKEEAINLGYLTEQQFNDWVRPEQMLGPK

>Saccharomyces cerevisiae (NM_001184076.1)

MLRFTNCSCKTFVKSSYKLNIRRMNSSFRTETDAFGEIHVPADKYWGAQTQRSFQNFKIGGARERMPLPLVHAFGVLKKSAAIVNESLGGLDPKISKAIQQAADEVASGKLDDHFPLVVFQTGSGTQSNMNANEVISNRAIEILGGKIGSKQVHPNNHCNQSQSSNDTFPTVMHIAASLQIQNELIPELTNLKNALEAKSKEFDHIVKIGRTHLQDATPLTLGQEFSGYVQQVENGIQRVAHSLKTLSFLAQGGTAVGTGLNTKPGFDVKIAEQISKETGLKFQTAPNKFEALAAHDAIVECSGALNTLACSLFKIAQDIRYLGSGPRCGYHELMLPENEPGSSIMPGKVNPTQNEALTQVCVQVMGNNAAITFAGSQGQFELNVFKPVMIANLLNSIRLITDAAYSFRVHCVEGIKANEPRIHELLTKSLMLVTALNPKIGYDAASKVAKNAHKKGITLKESALELGVLTEKEFDEWVVPEHMLGPK

>Schizosaccharomyces pombe (NP_588397.3)

MASVAHISTAKAIFRAGGLPCRRLITPTLTGLPLKTHRMNSTTPTYHLIPKGGKHGEFRQESDTFGPIQVPAEKYWGAQTQRSLQNFRIGGEKERLPLPLVRAFGVLKRAAASVNREFGLDPKLADAIEQAAQEVIDGRLDDNFPLVVFQTGSGTQSNMNSNEVIANRAIEILGGTLGSKKPVHPNDHVNMSQSSNDTFPTVMHIASVLQIHTHLLPAMKHLHRALKGKEEEFKNIIKIGRTHMQDATPLSLGQEFSGYVTQVGYGIERINNALPRLCLLAQGGTAVGTGLNTFEGFDVKVAEKVSKLTNIEFKTAPNKFEALAAHDAIVEMSGALNVIACSLMKIANDIRQLGSGPRCGLGELILPANEPGSSIMPGKVNPTQCEALTMVCAQVMGNHATITVAGASGHCELNVFKPLLAKNILSSIRLLGDACESFTDHCVVGIEPNYEGIARHLRDSLMLVTALNPHIGYDNCAKIAKTALKNKSTLKHEFVTLGFGTPEQFDEWVRPELMISAKKV

>Candida albicans (EEQ44776.1)

MLRLSRNFKAVAPLRTFTTSTINFQNTRIESDAFGEIEVPTDKYYGAQTARSKSNFKIGGEAARMPVPVVRAFGILKKSAAKVNEELGALDPKLSAAIQQAATEVAEGKLDDHFPLVVFQTGSGTQSNMNANEVISNRAIEILGGELGSKKPVHPNDHCNMSQSSNDTFPTVMHIAAVTEINNSLIPELTKLRDSLQAKAEEFKDIIKIGRTHLQDATPLTLGQEFSGYVQQLTNGIERVEKSLPNLLYLAQGGTAVGTGLNTKKGWDSKVAEEVSRLTGFPFKTAPNKFEALAAHDAIVEASGALNTVAASLFKIANDIRYLGSGPRCGYGELSLPSNEPGSSIMPGKVNPTQNEALTMVATQVFGNNAAITFAGASGQFELNVFKPVMIANLLSSIRLIADGAASFRVHCVDGIEANTDKIDKLLHESLMLVTALNPKIGYDAASKVAKNAHKKGITLKESCLELGALSSEEFDQWVRPENMIGPKD

>Aspergillus niger (XP_001395836.2)

MLTSAHTSRAAVRSMASLTHAASRASASPAVARTAVAFTPASFSCRRLLSSNSRPVQHFPRLQTLTSTSSKRAFGTTVKMSSATRIETDAFGEIEVPADKYWGAQTQRSLGNFDINQPQDRMPEPVVKAFGILKGAAAEVNMKFGLDPKIGEAIKQAAAEVAEGKLMDHFPLVVWQTGSGTQSNMNSNEVISNRAIEILGGEKGSKKPVHPNDHVNMSASSNDSFPTAMHIAAVVELENTLLPSLRSLRDALQVKVEKFDKIIKIGRTHLQDATPLTLGQEFSGYVAQLDRNIERVETSIPHLRYLAQGGTAVGTGLNTFKGFDEAIAAEVTKLTGTEFKTAPNKFEVLAAHDSIVEASGALNTLACSLFKIAQDIRYLGSGPRCGLGELVLPENEPGSSIMPGKVNPTQCESLTMVCSQVMGNHVAATVGGMNGQFELNVFKPLMIRNLLHSVRILADGMASFEKNLVHGLEANEPRINSLLHESLMLVTCLNPVIGYDMASKVAKNAHKKGLTLKQSAMELKALSEEDFDKYVRPELMLSPKEKK

BACTERIA

>Escherichia coli (AAC74683.1)

MNTVRSEKDSMGAIDVPADKLWGAQTQRSLEHFRISTEKMPTSLIHALALTKRAAAKVNEDLGLLSEEKASAIRQAADEVLAGQHDDEFPLAIWQTGSGTQSNMNMNEVLANRASELLGGVRGMERKVHPNDDVNKSQSSNDVFPTAMHVAALLALRKQLIPQLKTLTQTLNEKSRAFADIVKIGRTHLQDATPLTLGQEISGWVAMLEHNLKHIEYSLPHVAELALGGTAVGTGLNTHPEYARRVADELAVITCAPFVTAPNKFEALATCDALVQAHGALKGLAASLMKIANDVRWLASGPRCGIGEISIPENEPGSSIMPGKVNPTQCEALTMLCCQVMGNDVAINMGGASGNFELNVFRPMVIHNFLQSVRLLADGMESFNKHCAVGIEPNRERINQLLNESLMLVTALNTHIGYDKAAEIAKKAHKEGLTLKAAALALGYLSEAEFDSWVRPEQMVGSMKAGR

>Pelagibacterium halotolerans (YP_004900787.1)

MSTRTETDSMGPIEVPNEKYYGAQTARSLMNFDIGGEKMPLEIVHAFGILKKAAAVTNTKLGLMDEATRDLVVAAADEVIAGKLDDHFPLVVWQTGSGTQSNMNVNEVISNRAIEMAGGAMGSKKPVHPNDHVNMSQSSNDTYPTAMHIAAVTIVEDKLFAKVKLLRDTLAKKSEEFMDVVKIGRTHLQDATPLTLGQEISGWVAQIDLALKAIEATLPQLRELALGGTAVGTGLNTHPDYARMVAEEISSLSGHQFVTAPNKFAVLAGHDAFVGASGALKQLAVAFMKIANDVRWLASGPRSGLGEITIPENEPGSSIMPGKVNPTQSEAMTMVVAQVMGNDATIGFAASQGNFELNVYKPVIAFNFIQSVRLLADAAQSFNDNCAIGIEPDRKKINEHLNNSLMLVTALNRKIGYDNAAKIAKTAHKNGTTLREEAIALGLLTGEEFDAEVRPEQMVGPISVKK

>Magnetospirillum magneticum (YP_423013.1)

MNATRPEKDSFGIIQVPEPHIWGAQTQRSLEYFAISGERMPMELISALARVKGGCARANAELGLLAAGKADAIIAAAQEVLSGRHGEEFPLSVWQTGSGTQTNMNMNEVLANRASEILGGERGLNRCVHANDDVNLGQSSNDVFPTAMHVAAAVTVVTGLLPSLAHLRKTLVGLTEEFAAIVKIGRTHLQDATPLTLGQEFSGYAAQLDHAEAVLLATLPSLYPLAIGGTAVGTGLNTHPEFGARVAAQLAADLGLPFVSATNKFAALAAHDGMVAMHGAVKTLAAALMKIANDIRWLASGPRCGLGEISLPENEPGSSIMPGKVNPTQCEALTMACCQVMGNDVAITMGGASGNFELNVFKPLIAHNFLQSVRLMADGMASFERHCVRGITANRQRIDQLLEQSLMLVTALAPHIGYDRAAEIAKSAHAGGATLRQAALATGYVTAEQFDVWVDPTIMV

>Microvirga (WP_009764047.1)

MSPSTRIETDTFGPIDVPADKLWGAQTQRSLQNFRIGTDRMPLPLVHALAIVKQAAALVNKDLGKLEPRLADAIASAAADVVQGKYDDEFPLVVYQTGSGTQSNMNMNEVLSNLAIERLGGERGSKKPVHPNDHVNMGQSSNDSFPTAMHIAVAREIHDRLVPALKHLHKALNDKAEAYKDIVKIGRTHLQDATPVTLGQEFSGYAAQVQLGIARIEQTLPGIYALAQGGTAVGTGLNAHPEFADRFASKVVELTALPFTSANNKFEALASNDALVYTHGALASLAAGLFKIANDIRLMGSGPRSGIGEISLPENEPGSSIMPGKVNPTQAEAMTMLCCQVAGNQTTVTFAGSQGHFELNVFKPVIVNAVLQSIRLIADGAISFTDNCVVGIEPNRERLNELMQRSLMLVTALAPSIGYDKAAAIAKSAHKNGTTLKEEALKAGVAEDHFHAVVRPETMLQPGE

>Methylobacterium extorquens (YP_001640127.1)

MSPHENPSVETRTESDTFGPIEVPAHRYWGAQTQRSIQNFKIGTERQPAPLVHALGIVKQAAALVNKDLGGLDPKIADAIAESAAEVVAGQHDDEFPLVVWQTGSGTQSNMNANEVIASLANERLGGKRGGKSPVHPNDHCNRGQSSNDTFPTAMHIAVAREVQERLLPALSHLHTALDAKAKEFESIVKIGRTHLQDATPVSLGQEFSGYAAQVALGGARIAATLPGVLALAQGGTAVGTGLNAHPEFAERFAAKVAELTGLPFTSAENKFEALATHDALVFLQGALTALASGLFKIANDIRLLGSGPRSGLGELSLPENEPGSSIMPGKVNPTQCEALTMVCAQVVGNGTTVSFAGSQGHFELNVFKPVIANAVLQSVRILADASVSFTDNCVVGIKANTDRISDLMSRSLMLVTALAPSIGYDKAAEIAKTAHKNGTTLKEEALRLGYVTEEEFERVVRPETMLAPSAE

>Pseudovibrio (WP_008547491.1)

MSAQDSDSMRQESDSFGVLDVPVGKYWGAQTARSLINFPIGREHLPPALIHALGAVKLAAAKVNMEEGRLPEDLGKAICQAATEVISGKLDAHFPLVIWQTGSGTQSNMNTNEVIANRAIELLGGQIGSKSPVHPNDHVNMGQSSNDTFPTAMHIAAACEINKVLLPALVHLHDTLEAKVAAFSDIVKIGRTHTQDATPLTLGQEFSGYVAQLEYSKERIEQSLVGLYRLAQGGTAVGTGLNSTPEFASAFATHVSEVTGLPFVTATNKFEALATHDAAVFAHGALNTLACSLMKIANDIRFLASGPRSGLGEISLPANEPGSSIMPGKVNPTQCEAVTMVCAQVMGNQTAVSVAGATGHFELNVFKPVIANSLLQSIELLADCMVSFADRCVVGIEPNLDRIEELMERSLMLVTALAPTIGYDKATEIAKSAHKNGTTLKEEALRLGYVTAEEFDRVVRPEKMVRPG

>Rhodomicrobium vannielii (YP_004012301.1)

MTEDVSPSATRTETDTFGPIDVPANHYWGAQTQRSLGNFKIGGERMPIPLVHALGIVKKAAAKTNLALGNLEQPLHDAIVAAAEEVIAGKLDAEFPLVVWQTGSGTQSNMNANEVISNRAIEMLGGEKGSKKPVHPNDHVNRSQSSNDTFPTAMRIAAAVEVNKALLPALDHLASAIEAKAEEFKDIIKIGRTHLQDATPVTLGQEFSGYAAQLRFGIERVKATLPRLYLLAQGGTAVGTGLNTPIGFAEKFADEVEKETGLPFVTAPNKFEALASNDAEVELSGALNVLATSLFKIANDIRLMGSGPRSGIAEISLPENEPGSSIMPGKVNPTQCEALTMVCAQVMGNNVAITVAGSQGHFELNVYKPVIIYNLLQSIRLISDAAVSFTDHCVVGIVANRARIEDLLERSLMLVTALAPKIGYDKAAKIAKTAHKHGTTLREEALALGYVSEEDFNALMKPELMIKPGV

>Oceanibaculum indicum (WP_008945038.1)

MTESADRDAYRDESDTMGTVKVPADRLWGAQTQRSLQNFRIGGERMPAPLIHAFGIQKQAAALANMALGVLEKKLGDAIVTAAGEVADGTLADEFPLVVWQTGSGTQTNMNANEVIANRANELLGGQRGTKSPVHPNDHVNRSQSSNDSFPTVMHIAAASEIVNRLLPALRHLHAALEAKSRAFADIVKIGRTHLQDATPLTLGQEFSGYATQVAYGITRIEGTLPRLYELAQGGTAVGTGLNAPEGFDTAFAAEAARLTGLPFVTAPNKFEALAAHDALVEASGALNTLAVSLMKIANDIRLLGSGPRSGLAELRLPENEPGSSIMPGKVNPTQAEALTMVCAQVMGNHTTITVAGMSGHLELNVFKPVIAYNALQSIRLLADAALSFTDNCLAGIEADGTRISEHVERSLMLVTALAPHIGYDKAASVAKKAHSDGKTLRETALALGYVDADSFDRWVDPHQMIAPTRKGE

CYANOBACTETRIA

>Anabaena variabilis (YP_324057.1)

MTTNTDFRIERDSMGDRQIASDVYYGIQTLRAIENFPISGLKPLPTYVDAGLLIKKATAIVNGELGCIPQDISQAIVQATDEILAGKLRDQFVVDVYQAGAGTSHHMNINEVLANRALEILGEEKGNYKRVSPNDHVNYGQSTNDVIPTAIRIGGLLALTKTLQPALEKAIAALEHKAVEFQDIVKSGRTHLQDAVPVRLGDNFAAWAHILSEHQNRIYTASGDLMVLGLGGSAAGTGLNTHPQYRARVVEVLAELLNLPLQPAPHLMAAMQSMSPFVNVSGAIRNLAQDLAKISHDLRLMDSGPKTGLKEIQLPPVQPGSSIMPGKYNPVMAEMTSMVCFQVMGYDQAIALAAQAGQLELNVMMPLIAYDLIHSIEILGSTIAALTERCIQGITANKERCLAYAEGSLALVTALNTHIGYLNAADVAKESLNTGKSLRQIVLEKGLMSEAELATVLNLEEMSSILPLQAE

>Nostoc punctiforme (YP_001866048.1)

MTEHTDSQFRIERDSMGDRQIASSFYYGIQTLRAIENFPISGIKPLATYVDAGLIIKKATAIVNGELNCIPEDISQAIVQATDEILAGKFRDQFVVDVYQAGAGTSHHMNVNEVLANRALEILGEEKGNYKRVSPNDHVNYGQSTNDVIPTAIRIGGLLALSKTLHPAIEGAIASLENKAVEFQDIVKSGRTHLQDAVPVRLGENFRAWAHILTEHQNRIYTASGDLMVLGLGGSAAGTGLNTHPLYRARVVEVLSELIDTPLEPAPHLMAAMQSMAPFVNVSGALRNLAQDLVKISHDLRLMDSGPKTGLKEIQLPPVQPGSSIMPGKYNPVMAEMTSMVCFQVMGYDSAITLAAQAGQLELNVMMPLIAYNLIHSIEILGNTIAALTERCIEGITANQERCLAYAEGSLALVTALNTHIGYLNAAAVAKESLETGKSLRQIVLERGLMSETDLATVLNLEHMSGILPLKTE

>Prochlorococcus marinus (NP_876011.1)

MAQSFRIENDSMGTIKVPDQALWGAQTQRSLINFAIGHNKMPMKLIYSIVQIKASAAIVNCRLGVLDKQRKNFILNACNEISNGMHDEQFPLSVWQTGSGTQTNMNVNEVISNIASHLNGNKLGSHEPLHPNDHVNRSQSTNDVFPAAIQIATVQEILENLLPELDQLIETFDKKIIKWNRIIKTGRTHLQDAVPLTLGQEASAWKEQLIASRNRLNKSLNELYPLPLGGTAIGTGLNAPAKFDKEIALEIAKSTRSPFVSAQNKFAIMASHDALVHTMSQLKLLAVSLFKIVNDLRLLSCGPRGGLGELRLPENEPGSSIMPGKVNPTQCEAMAMVCTQIMALDSAVTMAGSGGHLQMNSYKPLIAFNLLESIDLLSSACKSSRILMIEGIEPNLEKIQNSLQNSLMLITSLTPIIGYEKASKIAQCAHEKDITLKEATKLLGYLNEDDFDRIVNPQSMTGMEN

>Prochlorococcus marinus (NP_895312.1)

MMADLMRIEHDSMGTIEVPAGVLWGAQTQRSLLNFAISTDRMPVELIHALALIKQAAASVNCRLGVLDEVQRDQIIKAASAVASGLHDDQFPLRVWQTGSGTHTNMNVNEVISNLASQANDEPLGSHRPVHPNDHVNRSQSTNDAFPTAIHIAAVQGITNNLLPELEQLIAAFARKSDAWSDIIKIGRTHLQDAVPLTLGQEASAWRDQIASAHSRIQSSLIELYPLPLGGTAVGTGLNAPARFGQETAAQLASITGLPFSSAKNKFAVMASHDGLVNAMAQLRMLAVALFKISNDLRLLACGPRAGLAELHLPENEPGSSIMPGKVNPSQCEAMAMVCLQVIGLDSAVTMAGGSGHLQMNVYKPLIGFNLLHSIELLHDACRKYRLAMVQGIEPNRIKIQHDLEQSLMLVTALAPEIGYDKASEIAHLAHEKGFSLREAALKLGYVSKEDFDRIVNPALMTSARL

>Cyanothece (YP_002378241.1)

MDNKFRIETDSLGEVQVPADKLWGAQTQRSLEYFNIGDDFMPPEMITAYVILKKASAIVNHRGGRLGDEQKELICQVCDEILAGQHQDQFPLKVWMTGSGTQFNMNINEVISNRCCQLAETPIGSKKPVHPNDHVNCAMSTNDSFPAAMCIAAAQGVKERLIPSIQQLRDSLNIKAKAWADIIKIGRTHMQDATPLTLGQEFSGYVGLLEDNLDRLEKCLDGVYQLALGGTAVGTGINAAPGFDREVAQEIAQLSGLPFVTAPNKFTVQGSHDALVMLSGALKTLATSLYKMANDIRLLSCGPRCGFNELHIPENEPGSSIMPGKVNPTQCEALAMLAVQVMANDMAVTFGGGGGYLEMNVYKPLMIFNILKSIRLMEDGCHNFRKFLVEGTQPNTKQIEYFLQRSLMLVTALSPVIGYDKASYVAHYALEKDLTLKQAALELNYISEEEYDRIVDPAKMVHPYVAEN

>Synechococcus (WP_007098867.1)

MVAATRVETDSMGPVEVPAEALWGAQTQRSLHNFAIAADRMPAELIHALARIKQAAAITNARLGVLDAERRDYIVAAATAVAEGLHDGQFPLRVWQTGSGTQTNMNLNEVISNLAAQAAGEPLGSHKPVHPNDHVNRSQSTNDAFPAAIHVAAAQGISHRLLPELQRLKNAFGAKADAWTDIVKIGRTHLQDAVPLTLGQEASAWRDQIGTAAQRIDASLAEVLPLPLGGTAVGTGLNAPDGFARLAAEELTRLTGLPFSSAPNKFAVMAGHDGLVNAMGQLRLLAVSLLKIANDIRLLACGPRAGLAELHLPANEPGSSIMPGKVNPTQCEAMAMVCTQVIGLDAAVAMAGSGGHLQMNVYKPLIGFNLLETITLLTDACRCFRVAMVEGMEPNRSRIEHDVEQSLMLVTPLAPVIGYDKASAIAKHAHEQGSSLKEAALELGYVSAEEFDQVVDPAAMAVAQD

>Synechocystis (NP_442130.1)

MVNSHRLETDSMGSLEVQADRLWGAQTQRSLMFFDIGSDVMPPDLIRAFAILKKAAAITNQDLGKLPADKAELIITAADEIIAGQWLDHFPLRIWQTGSGTQTNMNVNEVIANRAIAICGGELGSKNPIHPNDHVNMSQSSNDTFPTAMHIAAVAGLQTKLIPSLQALRDSLNEKAECFAGITKIGRTHLMDAVPLTLGQEFSGYVAQLDQGLTQINYCLPGLLELALGGTAVGTGLNSHPQFAKKVAEEIAQLTGYTFISAPNKFAALAGHEAIAFASGVLKSIAASLMKIANDLRWMGSGPRCGLGELALPANEPGSSIMPGKVNPTQCEAMTMVCVQVMGNDATIGFAASQGNFELNVFKPVIIHNFLHSLHLLSDACASFRQHLVVGLQVNESKVKDFLDTSLMLVTALNPHIGYDNAALVAKTAFAQGITLKQAAVDLGLLTPAQFDAWVVPEQMIAPIAD

>Thermosynechococcus elongatus (NP_682324.1)

MTTDTRLEYDSLGAVEVPADCYWGAQTARSLKHFAIGSQRMPLAVIHAMARLKKAAAIANRDLGVLDPEKAKWIIQAADEVIAGQWDDQFPLAIWQTGSGTQTNMNVNEVIANRAIELAGGVKGSKSPIHPNDHVNCSQSSNDTFPTAMHVATVLALQERLLPTLRHLLTVLQEKAAAFAEIIKIGRTHLMDAVPLTLGQEFSGYASQIAAAQAHIEYALQHLYPLAIGATAVGTGLNAPAGFGDRVAAELAQMTGYPFRKAENPFAALAAHDPLVMLSGALKTLAAALMKIANDIRWLGSGPRCGLGELRLPANEPGSSIMPGKVNPTQCEALTMVCVQVMGNDAAVGIAGSQGNFELNVYKPLIIYNVLQSIALLSDAAQSFTDHCLVGVEPNRQQIQAYVERSLMLVTALNPHIGYDKAAAVAKKAYSEGKTLKEAAVELGYLTAEEFDRWVRLELMLGEKGTS

_____________________________________________________________

MALATE DEHYDROGENASE

MITOCHONDRIAL

>Arabidopsis thaliana (AAG40021.1)

MFRSMLVRSSASAKQAVIRRSFSSGSVPERKVAILGAAGGIGQPLALLMKLNPLVSSLSLYDIANTPGVAADVGHINTRSEVVGYMGDDNLAKALEGADLVIIPAGVPRKPGMTRDDLFNINAGIVKNLCTAIAKYCPHALINMISNPVNSTVPIAAEIFKKAGMYDEKKLFGVTTLDVVRARTFYAGKANVPVAEVNVPVIGGHAGVTILPLFSQATPQANLSSDILTALTKRTQDGGTEVVEAKAGKGSATLSMAYAGALFADACLKGLNGVPDVIECSYVQSTITELPFFASKVRLGKNGVEEVLDLGPLSDFEKEGLEALKPELKSSIEKGVKFANQ

>Capsella rubella (EOA38168.1)

MFRSMLVRSSASAKQAVIRRSFSSGSVPERKVAILGAAGGIGQPLALLMKLNPLVSSLSLYDIANTPGVAADVGHINTRSEVVGYMGDDNLAKALEGADLVIIPAGVPRKPGMTRDDLFNINAGIVKNLCTAIAKYCPHALINMISNPVNSTVPIAAEIFKKAGMYDEKKLFGVTTLDVVRAKTFYAGKANVPVAEVNVPVIGGHAGVTILPLFSQATPQANLSGDVLTALTKRTQDGGTEVVEAKAGKGSATLSMAYAGALFADACLKGLNGVPDVVECSYVQSTITELPFFASKVRLGKNGVEEVLDLGPLSDFEKEGLEALKPELKSSIEKGVKFANQ

>Glycine max (XP_003527211.1)

MMKPSMLRSLHSAATRGASHLSRRGYASEPVPERKVAVLGAAGGIGQPLSLLMKLNPLVSSLSLYDIAGTPGVAADVSHINTGSEVVGYQGDEELGKALEGADVVIIPAGVPRKPGMTRDDLFNINAGIVKTLCTAIAKYCPHALVNMISNPVNSTVPIAAEVFKKAGTYDEKRLFGVTTLDVVRAKTFYAGKANVPVAGVNVPVVGGHAGITILPLFSQATPKANLDDDVIKALTKRTQDGGTEVVEAKAGKGSATLSMAYAGALFADACLKGLNGVPDVVECSFVQSTVTELPYFASKVRLGKNGVEEVLGLGPLSDFEQQGLESLKPELKSSIEKGIKFANQ

>Populus trichocarpa (XP_002316794.1)

MMLRSIKSLATSPSSHILRRGYASEAVPDRKVAVLGAAGGIGQPLALLMKLNPLVSSLALYDIANTPGVAADVSHINTRSEVSGYSGEAELGKALEGADVVIIPAGVPRKPGMTRDDLFNINAGIVKGLCQAIAKYCPHALVNMISNPVNSTVPIAAEVFKKAGTYDPKRLFGVTTLDVVRAKTFYAGKAKVPVAEVNVPVVGGHAGITILPLFSQATPKANLSDAEITALTKRTQDGGTEVVEAKAGKGSATLSMAYAGAIFADACLKGLNGAPDVVECSYVQSTITELPFFASKVRLGKNGVEEVLGLGPLSDYEKEGLEKLKPELQSSIEKGIKFANQ

>Solanum tuberosum (CAD33240.1)

MRTSMLKSIVRRSSTAGESYVSRRGFASGSAPERKVAVLGAAGGIGQPLSLLMKLNPLVSSLSLYDIAGTPGVAADVSHINTRSEVVGFAGEEQLGKALEGADIVIIPAGVPRKPGMTRDDLFNINAGIVKSLCTAIAKYCPNALVNMISNPVNSTVPIAAEVFKKAGTYDEKKLFGVTMLDVVRAKTFYAGKAKVNVAEVNLPVVGGHAGITILPLFSQATPKANLSNEEIVALTKRTQDGGTEVVEAKAGKGSATLSMAYAGAIFADACLKGLNGVPDVVECAFVQSNVTELPFFASKVRLGKNGVEEVLGLGPLNEYEKQGLEALKPELLSSIEKGIKFAKEN

>Vitis vinifera (NP_001268095.1)

MKASLFRSAETALRRVSSPSASSHLLRRSYCVESKPERKVAVLGAAGGIGQPLALLMKLNPLVSSLSLYDIAGTPGVAADVSHINTRSQVAGYMGDDQLGQALEGADLVIIPAGVPRKPGMTRDDLFNINAGIVKSLCTAIAKYCPNALVNMISNPVNSTVPIAAEVFKKAGTYDEKKLFGVTTLDVVRAKTFYAGKAKVPVAEANVPVVGGHAGITILPLFSQATPKSNNLSDEDIVALTKRTQDGGTEVVEAKAGKGSATLSMAYAGAVFADACLKVLNGVPDVVECSFVQSTIVPDLPYFASKVKLGKNGVEEVLGLGPLSDYEKQGLESLKHELKASIEKGIKFATQS

>Brachypodium distachyon (XP_003569456.1)

MRPSVMRSAAQLLRRRSYSSASGQPERKVAILGAAGGIGQPLALLMKLNPLVSSLSLYDIAATPGVAADISHINSPALVKGFMGDEQLAEALEGADLVIIPAGVPRKPGMTRDDLFNINAGIVKNLCTAIAKYCPNALINMISNPVNSTVPIAAEVFKKAGTYDEKRLFGVTTLDVVRAKTFYAGKANVPVTGVNVPVVGGHAGITILPLFSQATPASNALSAEEIKALTKRTQDGGTEVVEAKAGKGSATLSMAYAGAVFGDACLKGLNGVPDIVECSYVQSTITELPFFASKVRLGKNGVEEVLGLGQLSDFEKEGLESLKGELKSSIEKGIKFANAN

>Zea mays (NP_001140825.1)

MRPSLMRSTSQLLRRRSYSSASGQPERKVAILGAAGGIGQPLSLLMKLNPLVSSLSLYDIAGTPGVAADVSHINSPALVKGFMGDEQLGEALEGSDVVIIPAGVPRKPGMTRDDLFNINAGIVKNLSTAIAKYCPNALVNMISNPVNSTVPIAAEVFKKAGTYDEKKLFGVTTLDVVRAKTFYAGKANLPVTDVNVPVVGGHAGITILPLFSQATPATNALSDEDIKALTKRTQDGGTEVVEAKAGKGSATLSMAYAGAVFADACLKGLNGVPDIVECSFVQSTVTELPFFASKVRLGKNGVEEVLGLGELSDFEKEGLEKLKSELKSSIEKGIKFANDN

>Oryza sativa (NP_001043717.1)

MRPSLMRSASQVLRRRRGYSSASGQPERKVAILGAAGGIGQPLSLLMKLNPLVSSLSLYDIAGTPGVAADVSHINAPAQVKGFMGDDQLGEALEGSDIVIIPAGVPRKPGMTRDDLFNINAGIVKNLCTAIAKYCPNALVNMISNPVNSTVPIAAEVFKKAGTYDEKKLFGVTTLDVVRAKTFYAGKANVPVTDVNVPVVGGHAGITILPLFSQATPATNALSDEDIKALTKRTQDGGTEVVEAKAGKGSATLSMAYAGAVFANACLKGLNGVPDVVECSFVQSTVTELPFFASKVKLGKNGVEEVLGLGQLSDFEKEGLENLKGELKASIEKGIKFANA

>Chlamydomonas reinhardtii (XP_001703167.1)

MSLQSSIRADSNCTLPNNPVCVLLPVDFIVAAMASSTSSAMAKWAAQAARGFAAAAPSSGKGRKVAVLGAAGGIGQPLSMLMKMNSQVSSLSLYDIAGTPGVAADVSHINTKAQVKGFDKDGLAEALRGCDLVIIPAGVPRKPGMTRDDLFKINAGIVRDLVTAVGQHCPGAVLNIISNPVNSTVPIAAEQLKKMGVYDKRKVMGVTTLDVVRAKTFYAEKNGLDVASVDVPVVGGHAGVTILPLFSQATPKATMSAEVLDALTKRTQDGGTEVVQAKAGKGSATLSMAYAAALFADSCLRGLNGAPVVECTYVESTVTDAPYFASKVKLSTEGVDKIHDLGPLSDYEKAGLKAMMPELLASIEKGVQFVKGA

>Volvox carterii (XP_002953466.1)

MASQSSASLLKWAQAASRGFAAAAASGPGRKVAVLGAAGGIGQPLSMLMKMNAQVSQLSLYDVIGTPGVAADVSHINTKAQAKGFDKDGLAEALRGCDLVIIPAGVPRKPGMTRDDLFKINAGIVRDLVEAVGKHCPGALLNIISNPVNSTVPIAAEQLKKMGVYDKRKVMGVTTLDVVRAKTFYAEKNGLDVASVDVPVVGGHAGVTILPLFSQATPKVNMPHDVLDALTKRTQDGGTEVVQAKAGKGSATLSMAYAAALFADSCLRGLNGTPAVECTYVESSITDAPYFASKVKLSTEGVDKVFELGALSDYEKEGLKAMMPELLASIEKGVEFIKSG

>Ostreococcus lucimarinus (XP_001418230.1)

MFARALRRASNDAIARTRARGYKVAVLGAAGGIGQPCGLLMKMNPLVTELALYDIAGTPGVAADVSHVNTAAQTKGYAGDGELGAALKDADVVIIPAGVPRKPGMTRDDLFAINGGIVKGLVEAIADNCPNAMINMISNPVNSTVPIAAEVLKAKGKYDARKLFGVTTLDVVRAKTFYAEKAGLETAKVDVPVVGGHAGITILPLFSQATPKASNLSEADIDALTKRTQDGGTEVVAAKAGKGSATLSMAYAGALFADACLRAKNGEANIVECTYVESKITDAAFFASKVTLGRDGVDTIHGLGSLTAYEQANLDAMIPQLQGEIKKGIDFVKGA

>Micromonas pusilla (XP_003062229.1)

MTQTRGYKVAVLGAAGGIGQPCGLLMKMNPLVTELSLYDIAGTPGVAADVSHINTAAQVKGYAGDAELGAALKDADLVIIPAGVPRKPGMTREDLFKINAGIVAGLTEACATHCPNAMINMISNPVNSTVPIAAEVLKKRGAYDPKKLFGVTTLDVVRAKTFYAEKNGLETAKVDVPVVGGHAGITILPLLSQATPKAEMDAATIEALTKRTQDGGTEVVAAKAGKGSATLSMAYAGAVFADSCLRAKNGEAGVVECTYVESNVTDARFFASKVTLGKEGVETIHGLGELTPYEKAGLDGMMAELNDSINKGVEFAKAL

CYTOPLASTC

>Arabidopsis thaliana (AAM14159.1)

MAKEPVRVLVTGAAGQIGYALVPMIARGIMLGADQPVILHMLDIPFAAEALNGVKMELVDAAFPLLKGVVATTDAVEACTGVNVAVMVGGFPRKEGMERKDVMSKNVSIYKSQASALEKHAAPNCKVLVVANPANTNALILKEFAPSIPEKNITCLTRLDHNRALGQVSERLSVPVSDVKNVIIWGNHSSTQYPDVNHATVKTSVGEKPVRELVKNDEWLNGEFISTVQQRGAAIIKARKLSSALSAASSACDHIRDWVVGTPEGTFVSMGVYSDGSYNVPAGLIYSFPVTCRNGEWTIVQGLPIDDASRKKMDLTAEELKEEKDLAYSCLS

>Solanum lycopersicum (XP_004247734.1)

MAKDPVRVLVTGAAGQIGYALVPMIARGVMLGADQPVILHMLDIPPAAEALNGVKMELVDAAFPLLKGVVATTDAVEACTGVNVAVMVGGFPRKEGMERKDVMSKNVSIYKSQASALEKHAAPNCKVLVVANPANTNALILKEFAPSIPEKNITCLTRLDHNRALGQISERLSVQVSDVKNVIIWGNHSSSQYPDVNHATVSTPAGDKPVRELVADDAWLNGEFISTVQQRGAAIIKARKLSSALSAASSACDHIRDWVLGTPEGTFVSMGVYSDGSYNVPAGLIYSFPVTCKNGEWSIVQGLPIDEFSRKKLDLTAEELSEEKALAYSCLA

>Zea mays (NP_001105603.1)

MAKEPMRVLVTGAAGQIGYALVPMIARGVMLGADQPVILHMLDIPPAAEALNGVKMELVDAAFPLLKGVVATTDVVEACTGVNVAVMVGGFPRKEGMERKDVMSKNVSIYKSQASALEAHAAPNCKVLVVANPANTNALILKEFAPSIPEKNVTCLTRLDHNRALGQISERLNVQVSDVKNVIIWGNHSSSQYPDVNHATVKTSTGEKPVRELVSDDEWLNGEFITTVQQRGAAIIKARKFSSALSAASSACDHIRDWVLGTPEGTFVSMGVYSDGSYGVPSGLIYSFPVTCSGGEWKIVQGLPIDEFSRKKMDATAQELTEEKTLAYSCLE

>Triticum aestivum (ACQ57333.1)

MAAKEPMRVLVTGAAGQIGYALVPMIARGVMLGADQPVILHMLDIEFAAEALKGVKMELIDAAFPLLKGVVATTDVVEACTGVNVAVMVGGFPRKEGMERKDVMTKNVSIYKAQASALEAHAAPNCKVLVVANPANTNALILKEFAPSIPEKNISCLTRLDHNRALGQISERLGVQVSDVKNAIIWGNHSSSQYPDVNHATVKTPSGEKPVRELVQDDEWLNGEFIATVQQRGAAIIKARKLSSALSAASSACDHIRDWVLGTPEGTFVSMGVYSDGSYGVPAGLIYSFPVTCSGGEWTIVQGLPIDEFSRKKMDATAQELSEEKALAYSCLA

>Brachypodium distachyon (XP_003574062.1)

MAKDPMRVLVTGAAGQIGYALVPMIARGIMLGADQPVILHMLDIPQAAEALNGVKMELVDAAFPLLKGIVATTDVVEACTGVNVAVMVGGFPRKEGMERKDVMSKNVSIYKSQASALEAHAAPNCKVLVVANPANTNALILKEFAPSIPEKNISCLTRLDHNRALGQISERLNVQVTDVKNVIIWGNHSSSQYPDVNHATVNTPSGEKPVRELVKDDEWLNAGFIATVQQRGAAIIKARKFSSALSAASSACDHIRDWVLGTPEGTYVSMGVYSDGSYGVPAGLIYSFPVTCSSGEWTIVQGLPIDEFSRKKMDATAQELSEEKLLAYSCLE

>Medicago (O48905.1)

MAKDPVRVLVTGAAGQIGYALVPMIARGVMLGPDQPVILHMLDIAPAAESLNGVKMELVDAAFPLLKGVVATTDVVEACTGVNIAVMVGGFPRKEGMERKDVMSKNVSIYKSQASALEKHAAANCKVLVVANPANTNALILKEFAPSIPERNISCLTRLDHNRALGQISERLNVQVSDVKNVIIWGNHSSTQYPDVNHATVNTPAGEKPVRQLVSDDAWLNGEFISTVQQRGAAIIKARKLSSALSAASAACDHIRDWVLGTPQGTFVSMGVYSDGSYNVPSGLIYSFPVTCANGEWKIVQGLSIDEFSRKKLDLTAEELTEEKNLAHSCLS

>Micromonas (XP_002500781.1)

MVSPVTVMITGAAGQIGYALAPQVCRGAMLGPDTPIILHLLDIAPAETALKGVAMELVDAAYPLVKKIVATVNLEEACKGVDVAVMVGGFPRGPGMERKDVMAKNVAIYKGQASALQEHASPDVKVVVVANPANTNALILSNYAPKIPKENVTCLTRLDHNRALGQVSERTGVPVSDVKNCIIWGNHSSTQYPDVNHATAGGKSVRELVNDDEYLNGEFVTTVQQRGAAIIAARKFSSALSAASSVCDHVRDWVHGTPEGTWTSMGVISPGGDAGYGVEAGLMYSFPVTCAGGKWSIVQGLAIDDRSRSLMDATAAELKEEKTMAEELIAAM

>Chlamydomonas reinhardtii (XP_001694886.1)

MLGADQPIILHLLDVEPAKNALEGLRMELVDGAYPLLEGVLTFTDVAAACKDVDVAVMVGGYPRKAGEERKDVMAKNVSIYQQQASALEANASKDVKVVVVANPANTNALILAENAPSIPRENITCLTRLDHNRALGQVAERTGSHVGIVKNVIIWGNHSSTQYPDVNHGTVGGKPIRSAVNDDTWLNGDFITTVQQRGAAIIKARGLSSALSAANAVCNHVRDWVRGTPGGSWTSMGVVSDGSYGVQRGLVYSYPVTCAGGKWKVVQGLPIDGPSRERLRVTEAELVEERDLALQCLAEKK

>Volvox carteri (XP_002950493.1)

MELIDGAYPLLEGVFAFTDVAAACKDVDVAVMVGGYPRKAGEERKDVMAKNVSIYKSQASALAENASKDVKVVVVANPANTNALILAENAPSIPRENITCLTRLDHNRALGQVSERTKTHVTAVKNVIIWGNHSSTQYPDVNHGIVAGKPIRAVVGDDAWLNGEFISTVQQRGAAIIKARGLSSALSAANAVCNHVRDWVCGTPYGSWVSMGVVSDGSYGVQPGLVYSYPVTCSGGKWHVVQGLPIDDASKERMRTTEAELVEERDLALQCLAEKSAAAQ

>Ostreococcus tauri (XP_003079632.1)

MTRPRPRPRRGAGRPSRVHRACDRPRHRLARAFVMTRRPVNVVITGAAGQIGYALAPMVCAGAATGREKAIALRLLDVPFAEKALRGVMMELEDAAFELVESVSAHVDPEEAFVDADVCIMVGGFPRKAGMERKDVMGKNVAIYREQARALATKAKPGVKIVVVANPANTNANILRKFAPEIPAANVTCMTRLDHNRALAKLGGKSGRATRDVKNVIIWGNHSSTQYPDVNHATIEGKTAREVIGDDAYLDGEFVDAVRRRGAEIIEARQLSSALSAASSVCDHVYDWLNGTKEGEWTSMGVVSDGSYGVPEGLVYSFPVTCTGGKWQIVQGLTIDERSRKLMDESATELTEEFELAEACLAESA

PEROXISSOME

>Arabidopsis thaliana (NP_179863.1)

MDPNQRIARISAHLNPPNLHNQIADGSGLNRVACRAKGGSPGFKVAILGAAGGIGQPLAMLMKMNPLVSVLHLYDVANAPGVTADISHMDTSAVVRGFLGQPQLEEALTGMDLVIIPAGVPRKPGMTRDDLFNINAGIVRTLSEAIAKCCPKAIVNIISNPVNSTVPIAAEVFKKAGTFDPKKLMGVTMLDVVRANTFVAEVMSLDPREVEVPVVGGHAGVTILPLLSQVKPPCSFTQKEIEYLTDRIQNGGTEVVEAKAGAGSATLSMAYAAVEFADACLRGLRGDANIVECAYVASHVTELPFFASKVRLGRCGIDEVYGLGPLNEYERMGLEKAKKELSVSIHKGVTFAKK

>Prunus persica (EMJ01799.1)

MEPSVEANGRIARISAHLFPPNLQMEDGSALRRVDCRAKGGAPGFKVAILGAAGGIGQPLAMLMKINPLVSVLHLYDVVNAPGVTADISHMDTGAVVRGFLGQPQLESALTGIDLVIIPAGVPRKPGMTRDDLFNINAGIVRTLCEGIAKACPKAIVNLISNPVNSTVPIAAEVFKKAGTYDPKRLLGVTMLDVVRANTFVAEVLGLDPREVDVPVVGGHAGVTILPLLSQVKPPCSFTKEETEYLTNRIQNGGTEVVEAKAGAGSATLSMAYAAVKFADACLRGLRGDAGVVECAFVASEVTELPFFATKVRLGRNGADEIYQLGPLNEYERVGLEKAKRELAASIQKGVSFIKK

>Ricinus communis (XP_002522037.1)

MDSSAEAAQRIARISAHLHPPNFQMEGSSALKRADCRAKGGSPGFKVAILGAAGGIGQPLAMLMKMNPLVSVLHLYDVVNAPGVTADISHMDTGAVVRGFLGQPQLENALTGMDLVVIPAGVPRKPGMTRDDLFNINAGIVRTLCEGIAKCCPRAIVNLISNPVNSTVPIAAEVFKKAGTYDPKRLLGVTMLDVVRANTFVAEVLGLDPREVDVPVVGGHAGVTILPLLSQVKPPCSFTSEETEYLTKRIQDGGTEVVQAKAGAGSATLSMAYAAVKFADACLRGLRGDAGIVECSFVASQVTELPFFATKVRLGRNGAEEVYQLGPLNEYERIGLEKAKKELEASIQKGISFIKK

>Vitis vinifera (XP_002263670.2)

MHPTSDANQRIARISAHLQPSNFQMGESSGLSRENCRAKGGAPGFKVAILGAAGGIGQPLAMLMKMNPLVSVLHLYDVVNTPGVTSDISHMDTGAVVRGFLGQQQLEDALTGMDLVIIPAGVPRKPGMTRDDLFNINAGIVKTLCEGIAKCCPNAIVNLISNPVNSTVPIAAEVFKKAGTFDPKRLLGVTMLDVVRANTFVAEVLGLDPREVDVPVVGGHAGVTILPLLSQVKPPCSFTPEEIDYLTARIQNGGTEVVEAKAGAGSATLSMAYAAVKFADTCLRGLRGDAGVIQCAYVFSQVTELPFFASKVRLGRTGAEEIYPLGPLNEYERAGLEKAKKELASSIQKGISFIRK

>Cucumis sativus (XP_004143423.1)

MQPIPDVNQRIARISAHLHPPKYQMEESSVLRRANCRAKGGAPGFKVAILGAAGGIGQPLAMLMKMNPLVSVLHLYDVVNAPGVTADISHMDTGAVVRGFLGQQQLEAALTGMDLVVIPAGVPRKPGMTRDDLFKINAGIVKTLCEGIAKCCPTAIVNLISNPVNSTVPIAAEVFKKAGTYDPKRLLGVTMLDVVRANTFVAEVLGLDPRDVNVPVVGGHAGVTILPLLSQVKPPSSFTQEEINYLTDRIQNGGTEVVEAKAGAGSATLSMAYAAVKFADACLRGLRGDAGVVECAFVSSQVTELPFFATKVRLGRNGIDEVYSLGPLNEYERIGLEKAKKELAGSIEKGVSFIRG

>Brachypodium distachyon (XP_003576179.1)

MEKGGAGNAAARRMARLASHLHPSTSQMEEVSILRGYNCRAKGAAPGFKVAVLGASGGIGQPLSLLMKMNPLVSVLHLYDVVNMPGVTADISHMNTGAVVRGFLGQPQLENALTGMDLVIIPAGIPRKPGMTRDDLFNINAGIVRTLCEGIAKCCPNAIVNVISNPVNSTVPIAAEVFKKAGTYDPKRLLGVTTLDVVRANTFVGEVLGLDPRDVNVPVVGGHAGITILPLLSQVNPSCSFTSEEISYLTSRIQNGGTEVVEAKAGAGSATLSMAYAAAKFADACLRGLHGDAGIVECSYVASQVTELPFFASKVRLGRCGVEEILPLGPLNEFERAGLEKAKKELSESIQKGVSFINK

>Ostreococcus lucimarinus (XP_001416526.1)

MTVTKSQYSVAVLGAAGGIGQSLSLLLKMNPLISDLRLYDLANTPGVAADLSHTNTTCQVRGFMGADQLKDALKGADLVVIPAGVPRKPGMTRDDLFAINAGIVRDLCVACTEACPNALINIISNPVNSTVPIASEVFKKAGCYDPKKIFGVTTLDIVRSNTFVAEAKGLDINDVDVPVIGGHAGITILPLLSQTYPKCDFTAEEADKLTVRIQNAGTEVVEAKAGAGSATLSMAYAAARMAEACLRGLSGEPDVYECSYVASNITELPYFATKVRLGPSGADEVMPIGDITEYEADWLAKLKVELTGSIQKGVDFANQ

>Micromonas pusilla (XP_003055830.1)

MAAAAMPVAAPVLSARASFRGSSKSSVRRSTAKAAATTTRRRVATATTSEYKVAVLGAAGGIGQSLSLLLKMNPMIAQLNLYDIQGTPGVAADLSHTNTQTKVTGYAGADSLADALKGCDLVIIPAGVPRKPGMTRDDLFEINAGIVKTLCEAVAANCPGALVNIISNPVNSTVPIAAEVFKKAGTYDPKKVFGVTTLDVVRSNTFVAEAKGLDVNDVDIPVVGGHAGITILPLLSQSYPATKFDADELEAMTVRIQNAGTEVVEAKAGAGSATLSMAYAAARMAEACLRGLSGEADVYECSYVASSVTELPFFATKVRLGPGGAEEVLPVGDLTEYEKGWLEKLIPELKGSIDKGIAFANK

>Volvox carteri (XP_002954828.1)

MAALNRIQKIASHLDPPKLFKFKVAVLGAAGGIGQPLSMLLKMSPYISELSLYDVANTPGVAADVSHMSTAARVKGYLGPDQLPAALAGCHLVIIPAGVPRKPGMTRDDLFNINAGIVRTLAEAVAAHCPTAWVAIISNPVNSTVPIAAEVLQRAGVLNPARLFGVTTLDVVRAEAFIAEIVGADPRDVSVPVVGGHAGITILPLLSQARPALPASMTAEQRKALMVRIQDAGTEVVQAKAGAGSATLSMAYAAARFADSCLRAMSGEGPVNEYAYVRSSAVPGLPYFSSPLRLGRGGVEEIFPLGAVDAMEQENFEAMKAELLGSIKKGEEFAARGPAAAK

>Chlamydomonas reinhardtii (XP_001702586.1)

MADPLNRIQKIASHLDPAKPRKFKVALLGAAGGIGQPLSLLLKMSPYVSDLALYDVANTPGVAADVSHMSTAARVRGYLGPDQLGAALTGAALVIIPAGVPRKPGMTRDDLFNINAGIVRGLAQGIAQHCPAAWVAIISNPVNSTVPIAAEVLQKAGVFNPAKLFGVTTLDVVRAEAFIGELTGTDPRDVHVPVVGGHAGVTILPLLSQARPPLPASMSAEARKALMVRIQDAGTEVVQAKAGAGSATLSMAYAAARFADSCLRAMSGEGPVSEYAYIRHPPRLSSGSGSSVAVDLPYFSSPVRLGRLGVEEVLPLGPMDALEADNFAAMKAELLGSIKKGVEFAAKGPAPAK

CHLOROPLASTIC

>Arabidopsis thaliana (NP_190336.1)

MATATSASLFSTVSSSYSKASSIPHSRLQSVKFNSVPSFTGLKSTSLISGSDSSSLAKTLRGSVTKAQTSDKKPYGFKINASYKVAVLGAAGGIGQPLSLLIKMSPLVSTLHLYDIANVKGVAADLSHCNTPSQVRDFTGPSELADCLKDVNVVVIPAGVPRKPGMTRDDLFNINANIVKTLVEAVAENCPNAFIHIISNPVNSTVPIAAEVLKKKGVYDPKKLFGVTTLDVVRANTFVSQKKNLKLIDVDVPVIGGHAGITILPLLSKTKPSVNFTDEEIQELTVRIQNAGTEVVDAKAGAGSATLSMAYAAARFVESSLRALDGDGDVYECSFVESTLTDLPFFASRVKIGKNGLEAVIESDLQGLTEYEQKALEALKVELKASIDKGVAFANKPAAAAAN

>Brassica rapa (ACI25097.1)

MAAASSISIGSTVPRASSSSSSSSLPQSRAQAVNFNYSLPRFTALRSSTLLSGLDSSSFAKSLRGSVTKPQSTDTKPYGLNINASYKVAVLGAAGGIGQPLSLLIKMSPLVSTLHLYDIANVKGVAADLSHCNTPSQVRDFTGPAELADCLKDVNVVVIPAGVPRKPGMTRDDLFNINAGIVKTLVEAVADNCPNAFIHIISNPVNSTVPIAAEVLRKKGVYDPKKLFGVTTLDVVRANTFVSQKKNLKLIDVDVPVIGGHAGITILPLLSKTKPSVSFTDEEIEKLTVRIQNAGTEVVDAKAGAGSATLSMAYAAARFVESSLRALDGDGDVYECSFVDSTLTDLPFFASRIKIGRNGVEAVIESDLQGLTEYEHKALEALKPELKASIEKGVAFANKPAN

>Solanum lycopersicum (XP_004235981.1)

MAATSATTLSVGSTTSLGCKGSSISQSKAFGVKFNSKNNIRSFSGLKAATTVSCESESSFIGKESLAALKQSITPKAQKGNRGYVSCVQPQASYKVAILGASGGIGQPLALLVKMSPLVSELNLYDIANVKGVAADLSHCNTPSQVSDFTGASELANCLKGVNVVVIPAGVPRKPGMTRDDLFNINANIVKTLVEAVADNCPDAFIHIISNPVNSTVPIAAEVLKRKGVYDPKKLFGVTTLDVVRANTFVAQKKNLRLIDVDVPVVGGHAGITILPLLSKTKPSTTFTDEEVQELTVRIQNAGTEVVEAKAGAGSATLSMAYAAARFVESSLRALDGDSDVYECAFVQSDISELPFFASRIKIGKNGVEALISSDLQGLSEYEQKALDALKPELKSSIEKGTGFVQKEPVAA

>Glycine max (XP_003524782.1)

MAAAPAATFTIGTTGSLGQRGNSLPQLKSSGLKFNSQNHLKSFCGLKAMSSVRCESESSFLVNKTGAALRASFASKAQKENDQNFNYNSQPQASYKVAVLGAAGGIGQPLALLIKMSPLVSDLHLYDIANVKGVAADISHCNTPSQVRDFTGASELANCLKDVNVVVIPAGVPRKPGMTRDDLFNINAGIVRDLVSAVADNSPDAFIQIISNPVNSTVPIAAEVLKQKGVYDPKKLFGVTTLDVVRANTFVAQRKNLKLIDVDVPVVGGHAGITILPLLSKTRPSASFTDEEIEELTVRIQNAGTEVVEAKAGAGSATLSMAYAAARFVESSLRALDGDGDVYECSYVESDLTDLPFFASRVKLGRKGVEALIPSDLQGLTDYEQKALESLKPELMASIEKGIAFAQKQAVAA

>Vitis vinifera (XP_002284909.1)

MAATSLTTITIGSSASIGSKAIPLSRSKCFGVSFNSQNYLKSFSGLKAASSISCESEASFLGKESSAALRNSFAPKAGKQNLRHQYYLQPQASSSKVAILGAAGGIGQPLALLIKMSPLVSALHLYDIANVKGVAADLSHCNTPAQVLDFTGTSELANSLKGVDVVVIPAGVPRKPGMTRDDLFNINANIVKTLVEAVADNCPDAFIHIISNPVNSTVPIAVEVLRRKGVYDPKKVFGVTTLDVVRANTFVAQKKNLRLIDVDVPVVGGHAGITILPLLSRTKPSVSFTDEEVEELTVRIQNAGTEVVEAKAGAGSATLSMAYAAARFVESSLRALDGDTDVYECAYVQSELTELPFFASRVKIGKKGIEAVISSDLQGLTEYEEKALEALKPELKASIEKGVAFAQKQTVTA

>Brachypodium distachyon (XP_003574556.1)

MASAITISSVSAQAALISKPRNHGITSYSGLKASSSSISFETGSSFLGKNASLRASVTPRIVPKAKSGAQISPEASYKVAVLGAAGGIGQPLGLLIKMSPLVSELRLYDIANVKGVAADLSHCNTPSQVLDFTGPGELADCLKGADVVVIPAGVPRKPGMTRDDLFNINAGIVKSLIEAVADNCPEAFIHIISNPVNSTVPIAAEILKQKGVYNPKKLFGVSTLDVVRANTFVAQKKNLKLIDVDVPVVGGHAGITILPLLSKTRPSVTFTEEETEELTKRIQNAGTEVVEAKAGAGSATLSMAYAAARFVESSLRALAGDPDVYECTYVQSELTELPFFASRVKLGKNGVESIISSDLEGVTEYEAKALEALKPELKGSIEKGIEFVHKQQGATASV

>Coccomyxa subellipsoidea (EIE24005.1)

MSSRSRSSVVVEAKAGARKVALLGAAGGIGQPLALLLKMQPLIAELSLYDIANTVGVAADLSHCNTTVKVTGHTGQESLAAALEGADLVVIPAGVPRKPGMTRDDLFNINAGIVKTLAEAIAKHSPTAVIAIISNPVNSTVPITAEVLKKAGVYDPRKVLGVTTLDVVRANTFVAEAKGLAVQDVDVPVVGGHAGITILPLLSQTNPAVSFTEDEAAKLTDRIQNAGTEVVEAKAGAGSATLSMAYAAARFSESVLRGLEGEADVYEAAFVESKVTELPFFASKVRLGPNGVEEVLPLGKLTPFEEKGVADLIPVLKKNIDTGVEFANK

>Micromonas (XP_002500632.1)

MTAKAEYKVAVLGAAGGIGQSLSLLLKMNPLISDLALYDIANTPGVAADLSHTNTTCSVKGYAGEEQLADALKGCDLVIIPAGVPRKPGMTRDDLFSINAGIVKNLCEACAKNCPKAILNIISNPVNSTVPIASEVYKKAGVYDPKKIFGVTTLDVVRSNTFISEAKGLDVNDVDVPVVGGHAGITILPLLSQTYPSTKFTAEELEALTVRIQNAGTEVVEAKAGAGSATLSMAYAAARMAEACLRGLSGEAEVYECSYVASSVTDLPYFATKVKLGPNGAEEVLPVGDITDYEKGWLEKLIPELKASIDKGIEFANKD

ANIMALS

>Caenorhabditis elegans (NP_504656.1) cytosol

MSAPLRVLVTGAAGQIGYSIVIRIADGTVFGKEQPVELVLLDVPQCSNILEGVVFELQDCALPTLFSVVAVTDEKSAFTGIDYAFLVGAMPRREGMERKDLLAANVKIFKSQGKALAEYAKPTTKVIVVGNPANTNAFIAAKYAAGKIPAKNFSAMTRLDHNRALAQLALKTGTTIGNVKNVIIWGNHSGTQFPDVTHATVNKNGTETDAYAAVGDNAFLQGPFIATVQKRGGVIIEKRKLSSAMSAAKAACDHIHDWHFGTKAGQFVSMAVPSDGSYGIPQGLIFSFPVTIEGGEWKIVQGLSFDDFAKGKIAATTKELEEERDDALKACDDANI

>Caenorhabditis elegans (NP_498457.1) mitochondria

MSLPAKTLVQAAANSGLRAVSVRHSSQAPKVALLGAAGGIGQPLGLLLKQDPLVAHLALYDVVNTPGVAADLSHIDSNAKVTAHTGPKELYAAVENADVIVIPAGVPRKPGMTRDDLFNTNAGIVRDLAAVIAKASPKALIAIITNPVNSTVPIASEVLKKAGVYDPKRVFGVTTLDVVRSQAFVSELKGHDASKTVVPVVGGHAGITIIPLLSQVKPSTKFSEEEISKLTPRIQDAGTEVVNAKAGAGSATLSMALAGARFANALVRGIKGEKNVQCAYVASDAVKGVEYFSTPVELGPNGVEKILGVGKVSAYEQKLIDASVPELNKNIAKGVAFVKGN

>Homo sapiens (NP_005909.2) cytosol

MLSALARPASAALRRSFSTSAQNNAKVAVLGASGGIGQPLSLLLKNSPLVSRLTLYDIAHTPGVAADLSHIETKAAVKGYLGPEQLPDCLKGCDVVVIPAGVPRKPGMTRDDLFNTNATIVATLTAACAQHCPEAMICVIANPVNSTIPITAEVFKKHGVYNPNKIFGVTTLDIVRANTFVAELKGLDPARVNVPVIGGHAGKTIIPLISQCTPKVDFPQDQLTALTGRIQEAGTEVVKAKAGAGSATLSMAYAGARFVFSLVDAMNGKEGVVECSFVKSQETECTYFSTPLLLGKKGIEKNLGIGKVSSFEEKMISDAIPELKASIKKGEDFVKTLK

>Homo sapiens (NP_005908.1) mitochondria

MSEPIRVLVTGAAGQIAYSLLYSIGNGSVFGKDQPIILVLLDITPMMGVLDGVLMELQDCALPLLKDVIATDKEDVAFKDLDVAILVGSMPRREGMERKDLLKANVKIFKSQGAALDKYAKKSVKVIVVGNPANTNCLTASKSAPSIPKENFSCLTRLDHNRAKAQIALKLGVTANDVKNVIIWGNHSSTQYPDVNHAKVKLQGKEVGVYEALKDDSWLKGEFVTTVQQRGAAVIKARKLSSAMSAAKAICDHVRDIWFGTPEGEFVSMGVISDGNSYGVPDDLLYSFPVVIKNKTWKFVEGLPINDFSREKMDLTAKELTEEKESAFEFLSSA

>Bos taurus (XP_005225065.1) mitochondria

MLSALARPAGAALRRSFSTSAQNNAKVAVLGASGGIGQPLSLLLKNSPLVSRLTLYDIAHTPGVAADLSHIETRATVKGYLGPEQLPDCLKGCDVVVIPAGVPRKPGMTRDDLFNTNATIVATLTAACAQHCPEAMICIISNPVNSTIPITAEVFKKHGVYNPNKIFGVTTLDIVRANAFVAELKDLDPARVNVPVIGGHAGKTIIPLISQCTPKVEFPQDQLTTLTGRIQEAGTEVVKAKAGAGSATLSMAYAGARFVFSLVDAMNGKEGVVECSFVKSQETDCPYFSTPLLLGKKGIEKNLGIGKVSPFEEKMIAEAIPELKASIKKGEEFVKNMK

>Bos taurus (NP_001029800.1) cytosol

MSEPIRVLVTGAAGQIAYSLLYSIGNGSVFGKDQPIILVLLDITPMMGVLDGVLMELQDCALPLLKDVIATDKEEIAFKDLDVAILVGSMPRRDGMERKDLLKANVKIFKCQGAALDKYAKKSVKVIVVGNPANTNCLTASKSAPSIPKENFSCLTRLDHNRAKAQIALKLGVTSDDVKNVIIWGNHSSTQYPDVNHAKVKLQGKEVGVYEALKDDSWLKGEFITTVQQRGAAVIKARKLSSAMSAAKAICDHVRDIWFGTPEGEFVSMGIISDGNSYGIPDDLLYSFPVTIKDKTWKVVEGLPINDFSREKMDLTAKELAEEKETAFEFLASA

>Rattus norvegicus (NP_112413.2) mitochondria

MLSALARPVGAALRRSFSTSAQNNAKVAVLGASGGIGQPLSLLLKNSPLVSRLTLYDIAHTPGVAADLSHIETRANVKGYLGPEQLPDCLKGCDVVVIPAGVPRKPGMTRDDLFNTNATIVATLTAACAQHCPEAMICIISNPVNSTIPITAEVFKKHGVYNPNKIFGVTTLDIVRANTFVAELKGLDPARVNVPVIGGHAGKTIIPLISQCTPKVDFPQDQLATLTGRIQEAGTEVVKAKAGAGSATLSMAYAGARFVFSLVDAMNGKEGVIECSFVQSKETECTYFSTPLLLGKKGLEKNLGIGKITPFEEKMIAEAIPELKASIKKGEDFVKNMK

>Rattus norvegicus (NP_150238.1) cytosol

MSEPIRVLVTGAAGQIAYSLLYSIGNGSVFGKDQPIILVLLDITPMMGVLDGVLMELQDCALPLLQDVIATDKEEVAFKDLDVAVLVGSMPRREGMERKDLLKANVKIFKSQGAALEKYAKKSVKVIVVGNPANTNCLTASKSAPSIPKENFSCLTRLDHNRAKSQIALKLGVTADDVKNVIIWGNHSSTQYPDVNHAKVKLQGKEVGVYEALKDDSWLKGEFITTVQQRGAAVIKARKLSSAMSAAKAISDHIRDIWFGTPEGEFVSMGVISDGNSYGVPDDLLYSFPVVIKNKTWKFVEGLPINDFSREKMDLTAKELTEEKETAFEFLSSA

>Felis catus (NP_001265782.1) mitochondria

MLSALARPAGAALRRSFSTSAQNNAKVAVLGASGGIGQPLSLLLKNSPLVSRLTLYDIAHTPGVAADLSHIETRAAVKGYLGPEQLPDCLKGCDVVVIPAGVPRKPGMTRDDLFNTNASIVATLTAACAQHCPEAMICIISNPVNSTIPITAEVFKKHGVYNPNKIFGVTTLDIVRANTFIAELKGLDPARVNVPVIGGHAGKTIIPLISQCTPKVDLPQDQLTAVTGRIQEAGTEVVKAKAGAGSATLSMAYAGARFVFSLVDAINGKEGVVECSFVKSQETDCPYFSTPLLLGKKGIEKNLGIGKISPFEEKMIAEALPELKASIKKGEEFVKNMK

>Felis catus (NP_001009329.1) cytosol

MSEPIRVLVTGAAGQIAYSLLYSIGNGSVFGKDQPIILVLLDITPMMGVLDGVLMELQDCALPLLKDVIATDKEDVAFKDLDVAILVGSMPRRDGMERKDLLKANVKIFKCQGAALEKYAKKSVKVIVVGNPANTNCLTACKSAPSIPKENFSCLTRLDHNRAKAQIALKLGVTSDDVKNVIIWGNHSSTQYPDVSHAKVKLHGKEVGVYDALKDDSWLKGEFITTVQQRGAAVIKARKLSSAMSAAKAICDHVRDIWFGTPEGEFVSMGIISDGNPYGVPDDLLYSFPVTIKNKTWKVVEGLTINDFSREKMDLTAKELAEEKETAFEFLSSA

YEAST

>Saccharomyces cerevisiae (NP_012838.1) mitochondria

MLSRVAKRAFSSTVANPYKVTVLGAGGGIGQPLSLLLKLNHKVTDLRLYDLKGAKGVATDLSHIPTNSVVKGFTPEEPDGLNNALKDTDMVLIPAGVPRKPGMTRDDLFAINASIVRDLAAATAESAPNAAILVISNPVNSTVPIVAQVLKNKGVYNPKKLFGVTTLDSIRAARFISEVENTDPTQERVNVIGGHSGITIIPLISQTNHKLMSDDKRHELIHRIQFGGDEVVKAKNGAGSATLSMAHAGAKFANAVLSGFKGERDVIEPSFVDSPLFKSEGIEFFASPVTLGPDGIEKIHPIGELSSEEEEMLQKCKETLKKNIEKGVNFVASK

>Saccharomyces cerevisiae (NP_014515.2) cytosol

MPHSVTPSIEQDSLKIAILGAAGGIGQSLSLLLKAQLQYQLKESNRSVTHIHLALYDVNQEAINGVTADLSHIDTPISVSSHSPAGGIENCLHNASIVVIPAGVPRKPGMTRDDLFNVNAGIISQLGDSIAECCDLSKVFVLVISNPVNSLVPVMVSNILKNHPQSRNSGIERRIMGVTKLDIVRASTFLREINIESGLTPRVNSMPDVPVIGGHSGETIIPLFSQSNFLSRLNEDQLKYLIHRVQYGGDEVVKAKNGKGSATLSMAHAGYKCVVQFVSLLLGNIEQIHGTYYVPLKDANNFPIAPGADQLLPLVDGADYFAIPLTITTKGVSYVDYDIVNRMNDMERNQMLPICVSQLKKNIDKGLEFVASRSASS

>Saccharomyces cerevisiae (NP_010205.1) peroxissome

MVKVAILGASGGVGQPLSLLLKLSPYVSELALYDIRAAEGIGKDLSHINTNSSCVGYDKDSIENTLSNAQVVLIPAGVPRKPGLTRDDLFKMNAGIVKSLVTAVGKFAPNARILVISNPVNSLVPIAVETLKKMGKFKPGNVMGVTNLDLVRAETFLVDYLMLKNPKIGQEQDKTTMHRKVTVIGGHSGETIIPIITDKSLVFQLDKQYEHFIHRVQFGGDEIVKAKQGAGSATLSMAFAGAKFAEEVLRSFHNEKPETESLSAFVYLPGLKNGKKAQQLVGDNSIEYFSLPIVLRNGSVVSIDTSVLEKLSPREEQLVNTAVKELRKNIEKGKSFILDSSKL

>Candida albicans (XP_722820.1) mitochondria

MFSKVATRSFSSSASNAYKVAVLGAGGGIGQPLSLLLKLNHKVTDLALYDIRGAPGVAADVSHVPTNSTVKGYNPDQIEEALTGSDVIVIPAGVPRKPGMTRDDLFNTNASIVRDLAKAAADYAPNAAVCIISNPVNSTVPIVAEVFKSKGNYNPNKLFGVTTLDVLRAARFVSEVAGTNPVNENVPVVGGHSGVTIVPLLSQTKHKDLSGETRDALVHRIQFGGDEVVQAKDGAGSATLSMAQAGARFAGAVLDGLAGEKDVIECTFVDSPLFKDEGVDFFSTKVTLGVDGVKTVHPIGEISDYEEAQVKEAKDTLIKNIKKGVDFVAQNP

>Candida albicans (XP_717126.1) peroxissome MVKVTVAGAAGGIGQPLSLLLKLNPNVDELALFDIVNAKGVAADLSHINTPAVVTGHQPANKEDKTAITEALQGTDLVIIPAGVPRKPGMTRADLFNINASIIRDLVANIARVAPTAAILIISNPVNATVPIAAEVLKKLGVFNPRKLFGVTTLDSVRAETFLGELTNTDPTKLKGKISVIGGHSGDTIVPLINYDAGVGVLSDSDYKNFVHRVQFGGDEVVKAKNGAGSATLSMAYAGYRFADYVISSLTGGATPAGRIPDSSYIYLPGVSGGKEFSAKYVDGVDFFSVPVVLSQGEIRSFVNPFEELTVTKEEKKLVEVALKGLKGSITQGTEFVNASKL

>Candida albicans (XP_718638.1) cytosol

MVKVAILGAAGGIGQPLSLLTKLNPNVDELALFDVVNVPGVGADLSHINSDSKTQSYLPKDKEDKTALAAALKGSDLVIIPAGVPRKPGMTRDDLFNINASIVQGLAEGIAANSPKAFVLVISNPVNSTVPIVAETLQAKGVYDPARLFGVTTLDIVRANTFISQLFLDQTKPSDFNINVVGGHSGETIVPLYSLGNSKQYYDILSEEQKKELIKRVQFGGDEVVQAKNGAGSATLSMAYAGYRLAESILAAVNGKTDIVECTFLNLDSSIKGASEARKLVKDLDFFSLPVQLGKNGITEVKYDILNQISDDEKKLLEVAIEQLQKNIEKGVSFAKK

BACTERIA

>Escherichia coli (AAC75270.1)

MKKVTAMLFSMAVGLNAVSMAAKAKASEEQETDVLLIGGGIMSATLGTYLRELEPEWSMTMVERLEGVAQESSNGWNNAGTGHSALMELNYTPQNADGSISIEKAVAINEAFQISRQFWAHQVERGVLRTPRSFINTVPHMSFVWGEDNVNFLRARYAALQQSSLFRGMRYSEDHAQIKEWAPLVMEGRDPQQKVAATRTEIGTDVNYGEITRQLIASLQKKSNFSLQLSSEVRALKRNDDNTWTVTVADLKNGTAQNIRAKFVFIGAGGAALKLLQESGIPEAKDYAGFPVGGQFLVSENPDVVNHHLAKVYGKASVGAPPMSVPHIDTRVLDGKRVVLFGPFATFSTKFLKNGSLWDLMSSTTTSNVMPMMHVGLDNFDLVKYLVSQVMLSEEDRFEALKEYYPQAKKEDWRLWQAGQRVQIIKRDAEKGGVLRLGTEVVSDQQGTIAALLGASPGASTAAPIMLNLLEKVFGDRVSSPQWQATLKAIVPSYGRKLNGDVAATERELQYTSEVLGLNYDKPQAADSTPKPQLKPQPVQKEVADIAL

>Methylobacterium (WP_017484916.1)

MNSDDKLHIRSANVQHSPISISRRQVLGGAVAGLAATALPGSPLLAAGEARKVDVLLIGGGIMSATLGVWLRELEPDWSLEMVERLDGVALESSNGWNNAGTGHSALAELNYTPEDSKGNIQISKAVEINEAFQITRQFLAWQVRQGVLKNPRSFINSTPHMSFVWGDDNITYLRKRYEALKASPLFAGMEFSTDPEQVKNWVPLMMEGRDPKQKVAATWSPLGTDCEWGEVTRQYIASLKSGPKFDLRLSTEVESFERNGDGTWRVTSKNLKDGSRSTVDARFVFIGAGGGALHLLQASGIPEADDYAGFPVGGSFLVTENQDVALRHLAKAYGKASVGSPPMSVPHLDTRVLDGKRVILFGPFATFSTKFLKEGSYFDLLTSTTTSNVWPMVRVGVEQYPLIEYLAGQVMLSDEDRYQALREYFPDAKKDEWRLVQAGQRVQIIKRDPEKGGVLKLGTEVVAAKDGSIAALLGASPGASTAAPIMLNVLEKVFSQKVASPEWQQKIRQIVPSYGTKLNGDPQRVYQELAYTSEHLQLTPPPQIGTPAPAPAPAETGTVKPVPDMAP

>Agrobacterium tumefaciens (WP_003494302.1)

MAVTPIDQDKSQLTDLSKETSLSNAFPMNRRQVLGGALAGIAAATLPVTPLLANTAAKKVDVLLIGGGIMSATLGVWLRELEPTWSMQMLERLDGVALESSNGWNNAGTGHSALAELNYTPEDDNGNIKISQAVNINESFQISRQFWAWQVRNGVLKNPRSFINHTPHMSFVWGDENVAYLEKRYQALKASPLFAGMEYSTDPEQIKKWVPLMMEGRDPSQKIGATWSPLGTDMEFGEITRQFVSHLQGDQNFDLQVNSEVSDIQRNADGSWRVTYANTKTDAEQVVDAKFVFIGAGGGALHLLQMSGIPEGDDYAGFPVGGSFLINENPDVTMQHLAKAYGKASVGSPPMSVPHLDTRVLGGKRVILFGPFATFSTKFLKEGSYFDLVSSVTTSNAWPMVRVGIDEYPLVEYLAGQLMMSDDDRFAALKEYFPNAKQGEWRLWQAGQRVQIIKRDAEKGGVLRLGTEVVAAKDGSIAGLLGASPGASTAAPIMLSVLEKVFKDKVATPEWQAKIRQIVPSYGTKLNDDPEKVQQEWAYTAEHLQLPTPPQIDLEALKGAGAAPAGAPVKKVPDIAL

>Sphingobium yanoikuyae (WP_010338787.1)

MTSSSSPAAPRRRPLFKKILLTLAILLLVLAIAATAFLFRPMASPAGKAAANDQPIDVLVIGGGIMGVTLATYLQEMEPDWRIDIYERMDKVAQESSNGWNNAGTGHSGFAELNYTPEKDDGSIATEKAVDIAEQFEISRQFWSHEVRQGRLPKPTDFINATPHMSFVWGDDNIAYLHKRRDALVKNPLFYGMQYSQDPAQIRRWAPLVMEGRDPKQKVAATFMPLGTDVNFGVITTQLTNALTRNRNFTLQLGHEVRGLHQNADKTWNVTVHDLKKNSDAVVKAKFVFIGAGGASLKLLQMSGIPESKNYAGFPVGGQFLAFEGPAATSRHNVKVYGKAEAGSPPMSVPHLDARKLDGKSVTLFGPFALQSTKFLKNGSSLDLFASIFKNNVGGMMKVGAENLDLVRYLAQQATLTDAQRQAELVKYYPNAKRGDWKLITAGQRVQVIKYDPKKGTVLQFGTEIVTDKDASIAALLGASPGASTSPAIMLEVMRRAFPQQMATSWKPKVAAMVPSYGQKLNASPDLTNRIRRMTSQTLGLPYVEVPAGLNGAMPDAGAKLPAKRNLNSEQQAL

>Acetobacter tropicalis (WP_006557936.1)

MMSSTTTDTSSSVDVVLIGGGVMSATLGAFLRQLQPDWSISIFERLDNVAEESSDAWNNAGTGHSALCELNYTPQQADGSVDISKAVAVNESFQISRQFWAYLVEQGIIATPRDFITPVPHMSFVWGDANVNFLKKRYEALSAHPLFEGMEYSEDPAQMAQWMPLAMKNRPAGQKLAVTRSLNGTDVNFGALTRLLFGYLASTPACTLHTKHEVQDIKQGKDGRWRVQVQDLRLNTQRTVSAKFVFIGGGGGALPLLQKTGIPESRGVGGFPVSGQFLRCKNQDIIAQHHAKVYGKASVGAPPMSVPHLDTRMINGQRALLFGPYAGFSTRFLKNGSLLDLPRSIKSNNIGAILAVAKDNWPLTKYLIQQVLQSNSERINALRDFVPEARSEDWELVTAGQRVQIIKKDARKGGVLQFGTEVIASQDGSVAALLGASPGASTAAPIMLTVLKKCFADKLPEWDAKLKEIIPSFGQKLADNPELCTQLRDKTTTVLNLKE

CYANOBACTERIA

>Synechococcus (YP_729866.1)

MDRYDVVLVGAGIMSATLATLLHELDPELQLLVVERLEAPALESSAAGNNAGTGHAANCELNYTPLQADGTISTEKPLAINASFESSLEFWATLSERGRLDPSCFIHRVPHISFVWGEGDVAYLRQRYEQLKSLPAFAAMDWSRDEAELASWIPLVMAGRDPQIAVAATRIERGTDVDFGALSRSLFVPLQASGALDLVFGTSVSDLNRQAEGWELQLRGPSGRRVVMTPFVFLGAGGGALPLLQRSGIPEATAYAGFPVSGQWLVCNDPDLSEHHFAKVYGKAKVGAPPMSVPHLDSRWIDGRRSLLFGPYAGFSSKFLKQGSLLDLPRSVRSSNLLPMLQVGVNNIPLVRYLVNQLRQSSEERMEALKAFLPTARTEDWTLSVAGQRVQIIKRTSAGGRLQLGTEVVSAADGSLAALLGASPGASTSVEIMLEILQRCFPDRLASETWQQRLQALLPSYGQDLNANGELLQRSRDRSDALLGLQIAR

>Cyanobium gracile (YP_007047712.1)

MTLTTVDVTLVGAGIMGATLGTLLRQLQPDLSLLLVEGLPREAQESSSAWNNAGTGHAGNCELNYTPMAADGSISLAKALDINEAFDLSRQFWTHLVRDGALGSPGAFIQGVPHMSFVLGEERRAYLRERHRRMAAHHCYAGMAYSEDHAQVAAWAPLLIEGRDPAQAIGATHIAAGTDVNFGALTSQLLDHLRASGAFEARFSTAVQSLRRRPEGGWRLETRDRTTGETRLIDTGTVFLGAGGGALTLLQKSGIPEGRGYGGFPVSGLWLRCGDPALASRHHAKVYGMAAAGSPPMSVPHLDTRLVDGRQWILFGPYAGFSSKFLKSGSLCDWPRSLRAGNLLPLLAVARDNLPLTEYLIGQVFASAEQRFAALRDFYPDARPSDWTLAVAGQRVQIIKADARRGGVLQFGTEVVHAADTSLVAVLGASPGASAAVAIALEILHSCFRETLPDGWQATLKRILPSYGASIRDDADLCRRIRSESAEVLGLAAPA

>Prochlorococcus marinus (NP_874812.1)

MVLSDSLASDTRFDAVLIGAGIMSSTLAVLLHELDPEMRILIVERLEAPALESSSALNNAGTGHAANCEFNYTPCHPDGSINIQKALVINSAFERSLEFWASMTELGKLSPKTFLNLVPHISFVNCEEDVLFLQQRYKKLSAINAFRDMEWSADKEELSEWIPLIMNNRNPDQKVAATRVKRGTDINFGALTLAYLETLQESGAVELKLSTEVVDISRLQEETWEISLANTAGTYCVQAPFLFLGAGGGALSLLQNSGIEEGKKYGGFPVSGQWLVCNDSKLVKMHHAKVYGKSAIGAPPMSVPHLDTRWIGTERFLLFGPFAGFNTKFLKNGSNWDFLRSIQFSNFAPMIQTGLRNFDLIKYLIGQLKLDHDDRIAVLKDFFPEARSSNWTLSLAGQRVQIIKKTSKGGVLKLGTEVVTSSDGSLAALLGASPGASTAISIMLEVLQRCWGKKMSTDIWQKRLRDLLPSFGQDINHDKSLLDKLRNRSDSLLGLR
